# Ultra-Deep Sequencing Reveals the microRNA Expression Pattern of the Human Stomach

**DOI:** 10.1371/journal.pone.0013205

**Published:** 2010-10-08

**Authors:** Ândrea Ribeiro-dos-Santos, André S. Khayat, Artur Silva, Dayse O. Alencar, Jessé Lobato, Larissa Luz, Daniel G. Pinheiro, Leonardo Varuzza, Monica Assumpção, Paulo Assumpção, Sidney Santos, Dalila L. Zanette, Wilson A. Silva, Rommel Burbano, Sylvain Darnet

**Affiliations:** 1 Instituto de Ciências Biológicas, Universidade Federal do Pará, Belém, Brazil; 2 Departamento de Genética, Faculdade Medicina Ribeirão Preto, Universidade de São Paulo, Ribeirão Preto, Brazil; 3 Life Technologies, São Paulo, Brazil; 4 Hospital Universitário João Barros Barreto, Universidade Federal do Pará, Belém, Brazil; 5 Centro Regional de Hemoterapia, Faculdade Medicina Ribeirão Preto, Universidade de São Paulo, Ribeirão Preto, Brazil; 6 Instituto Nacional de Ciência e Tecnologia em Células-Troncos e Terapia Celular (CNPq/MCT), Ribeirão Preto, Brazil; Duke-National University of Singapore Graduate Medical School, Singapore

## Abstract

**Background:**

While microRNAs (miRNAs) play important roles in tissue differentiation and in maintaining basal physiology, little is known about the miRNA expression levels in stomach tissue. Alterations in the miRNA profile can lead to cell deregulation, which can induce neoplasia.

**Methodology/Principal Findings:**

A small RNA library of stomach tissue was sequenced using high-throughput SOLiD sequencing technology. We obtained 261,274 quality reads with perfect matches to the human miRnome, and 42% of known miRNAs were identified. Digital Gene Expression profiling (DGE) was performed based on read abundance and showed that fifteen miRNAs were highly expressed in gastric tissue. Subsequently, the expression of these miRNAs was validated in 10 healthy individuals by RT-PCR showed a significant correlation of 83.97% (P<0.05). Six miRNAs showed a low variable pattern of expression (miR-29b, miR-29c, miR-19b, miR-31, miR-148a, miR-451) and could be considered part of the expression pattern of the healthy gastric tissue.

**Conclusions/Significance:**

This study aimed to validate normal miRNA profiles of human gastric tissue to establish a reference profile for healthy individuals. Determining the regulatory processes acting in the stomach will be important in the fight against gastric cancer, which is the second-leading cause of cancer mortality worldwide.

## Introduction

Recently, Friedman *et al.*, 2009 demonstrated that the majority of human genes are under the control of miRNAs. The >45,000 miRNA target sites within human 3′UTRs are conserved, and >60% of the human protein-coding genes have been under selective pressure to maintain pairings to miRNAs [1]. miRNAs are small, non-coding sequences of 17–25 bp that regulate gene expression by binding to the 3′ end of target mRNAs, resulting in the inhibition of translation of the mRNAs [2], [3]. Approximately 14,000 miRNAs have been identified in animals, plants and fungi [4], [5], [6].

Mechanisms that involve miRNAs as negative regulators of gene expression are similar in animals and plants; they regulate fundamental cellular processes [6]. In humans, approximately 3% of all genes are predicted to encode miRNA precursors, and over >60% of protein-coding genes could be regulated by miRNAs [1]. MiRNAs have essential functions in many cellular processes such as growth and development, cellular proliferation, differentiation and apoptosis. Consequently, alterations in miRNA expression contribute to human diseases such as cancers [7], [8].

Alterations in miRNA expression patterns have been observed in many types of human cancer, such as breast, colon, lung, prostate, leukemia and stomach cancers. miRNA expression alterations lead to a loss or gain of function and are associated with the development of human neoplasm via diverse mechanisms [5].

Here, we present a genetic study of the miRNA of the human stomach, because despite the importance of the organ, little genetic data are available on their presence and regulation in humans. This work is the first full miRnome sequencing of normal stomach tissue using next-generation sequencing technology.

## Results

In the present study, profiles were obtained by ultra-deep sequencing using the SOLiD platform (Life Technologies, CA, US). This is the first study to use this procedure to describe the miRnome of normal gastric tissue. miRNAs were isolated from normal gastric cardia mucosa of a single healthy patient. The profiles were validated using Real Time PCR to determine the expression of the 15 most highly expressed miRNAs in another 10 healthy patients.

### ULTRA-DEEP SEQUENCING

**Figure 1:**
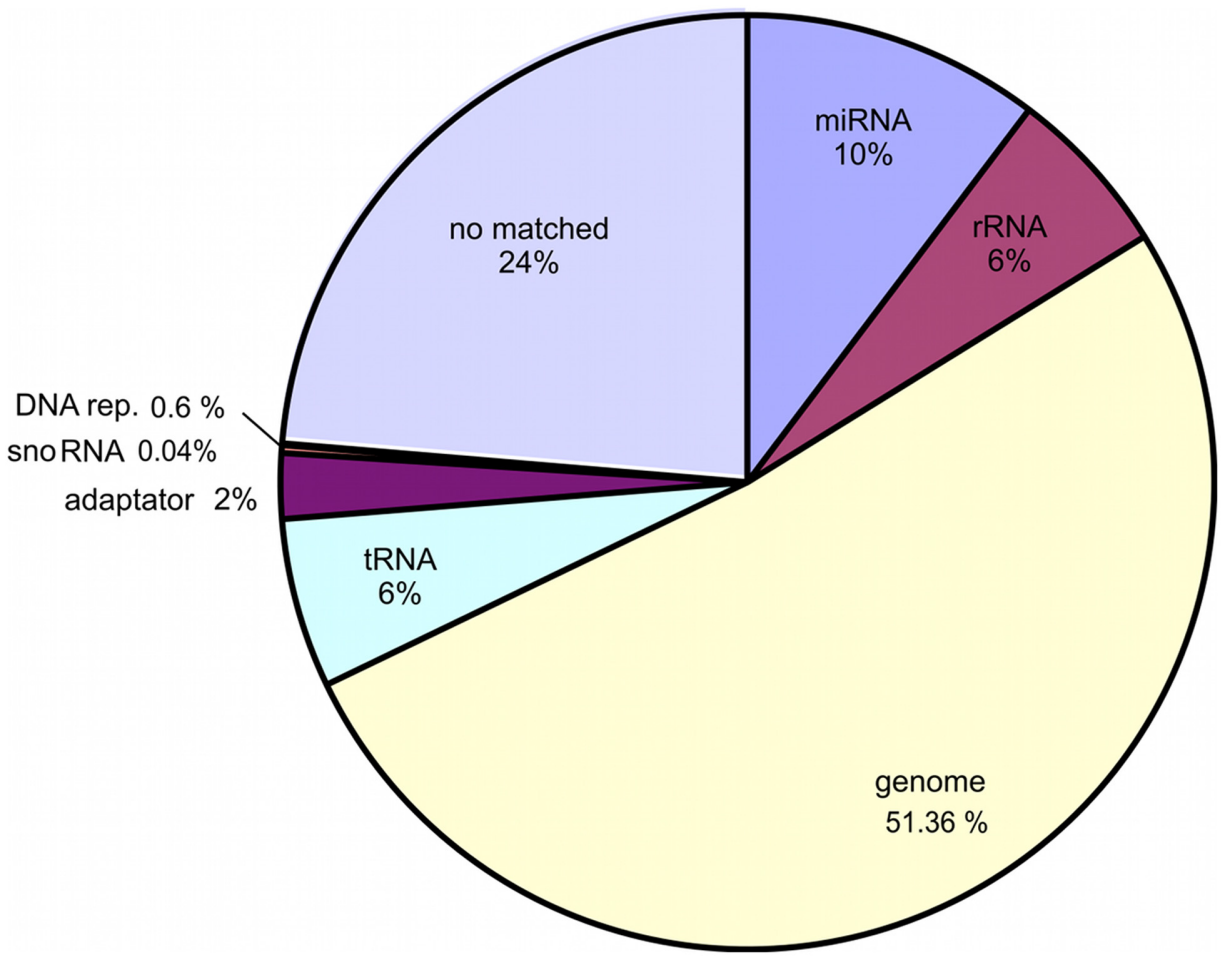
Distribution of small RNAs sequenced from human gastric cardia tissue using deep-sequencing.

Ultra-deep sequencing yielded a total of 104 million raw reads and 5 million reads for the gastric cardia miRNA library. For further analysis, 3.2 million reads were selected according to sequence quality (at least QV≥10 in the 10 first bases) [8]. After mapping these to the human genome (release 19), the total mapped read count was 2,534,490 million reads (75% of total quality reads). Approximately 38% of 2.5 million reads (963,460 reads) were repetitive DNA sequences, tRNA, rRNA or other small molecules; 10% (261,274) were accepted reads, perfectly aligned with known mature miRNAs (MirBase version 15, release 04/2010) [9]; and the rest of the reads (52%) were matched to genome sequences (Figure 1).

**Figure 2:**
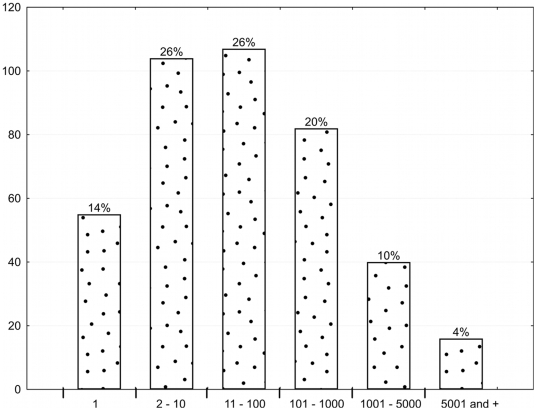
Distribution of microRNA by read count number in human gastric cardia. The read count is based on quantity of read detected during the deep-sequencing of small RNA library of gastric cardia, using SOLiD system. For microRNA detection was used the miRBase release 15.0.

For the expression analysis of miRNAs, only reads with matches to mature miRNAs sequences (261,274 reads) were included. In gastric cardia, we identified 404 of 970 known mature miRNA sequences (42%). To analyze the expression of miRNAs, we defined five ranges: 1 to 10 read counts; 11 to 100; 101 to 1,000; 1,001 to 5,000; and a read count of over 5,000 (Figure 2) (additional details are provided in **Table S1**). The first range (1–10) comprised 40% of the miRNAs observed; the second range (11 to 100) comprised 26%; the third range (101 to 1,000) comprised 20%; and the fourth and fifth ranges (>1000 read count) comprised 14%.

With this classification, 347 mature miRNAs were expressed between the first and third ranges and 57 between the fourth and fifth ranges. For the characterization of the miRnome, we selected a set of the 15 miRNAs that were expressed at the highest levels (≥1,000 reads).

**Figure 3:**
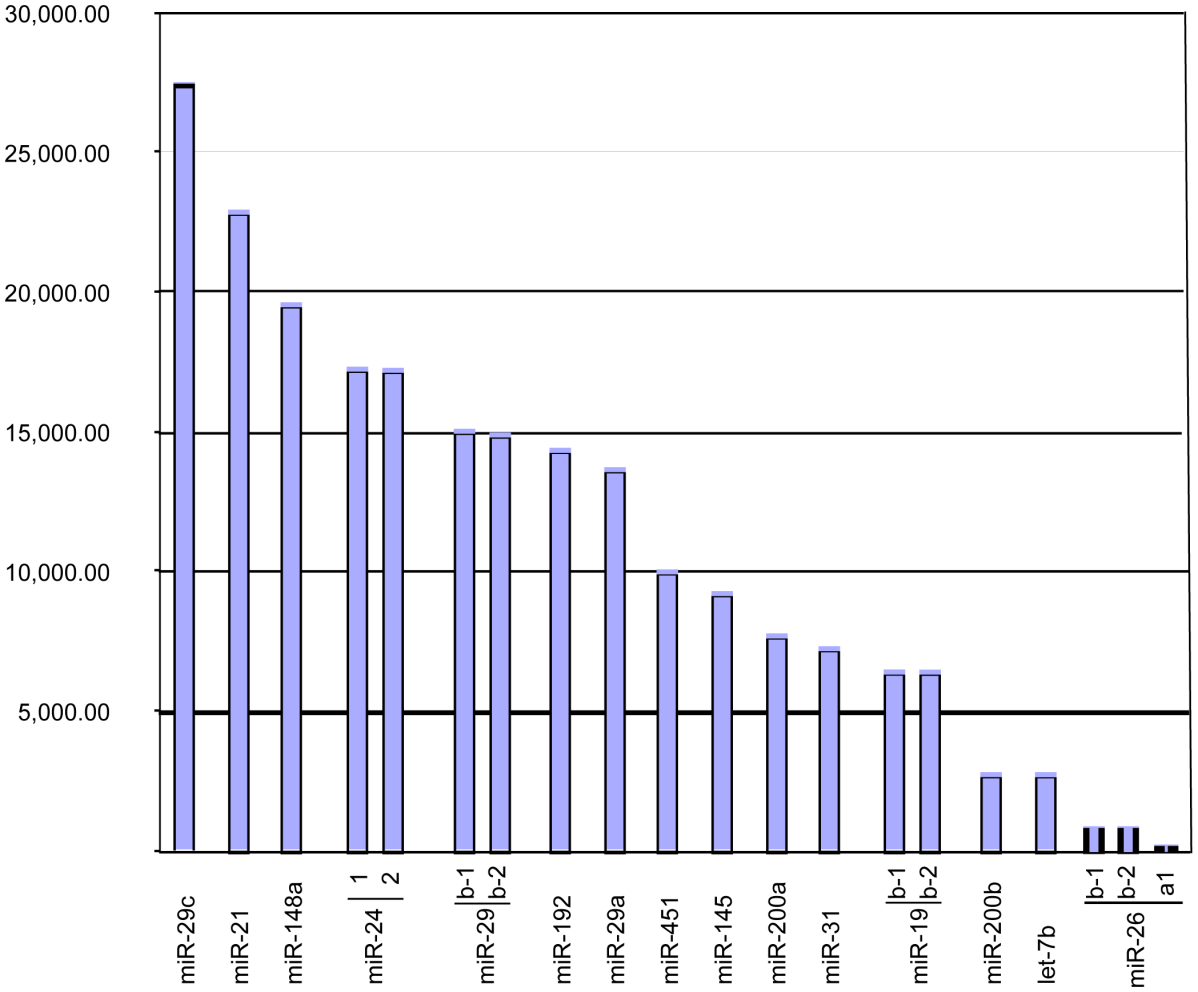
Most expressed microRNAs in human gastric cardia. The read count is based on quantity of read detected during the deep-sequencing of small RNA library of gastric cardia, using SOLiD system.

**Figure 4:**
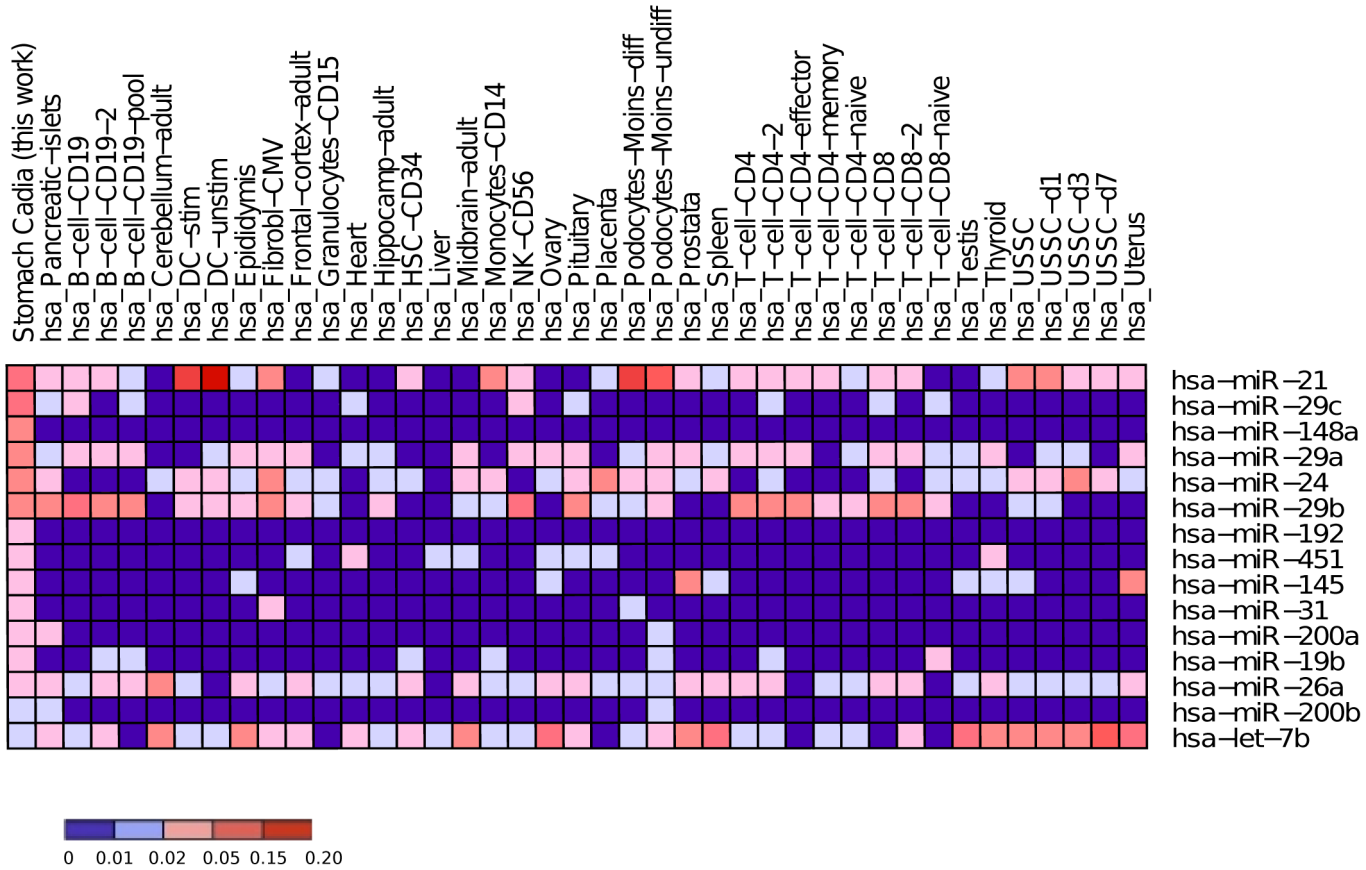
Heatmap of normalized expression of the 15 most expressed mature miRNAs in human gastric tissue and their comparison with other normal tissues published in the mammalian microRNA expression atlas (Landgraf, P. et al. (2007). Cell 129: 1401–1414). The heatmap was generated using gene pattern software with normalized expression of microRNA (read count number for specific microRNA/total of count number of microRNA). Color scale indicates the percent of total count reads number 0 to 0.2 (0 to 20%). Human tissue description and abbreviation: hsa_B-cell-CD19 (B cells from peripheral blood); hsa_B-cell-CD19-pool (B cells from peripheral blood (pool from 4 healthy donors)); hsa_B-cell-CD19-2 (B cells from peripheral blood); hsa_DC-unstim (myeloid dendritic cells not stimulated); hsa_DC-stim (myeloid dendritic cells stimulated with endotoxin); hsa_DC-unstim (myeloid dendritic cells not stimulated); hsa_DC-stim (myeloid dendritic cells stimulated with endotoxin); hsa_Fibrobl-CMV(Foreskin fibroblasts; Primary fibroblasts lytically infected with Cytomegalovirus); hsa_Frontal-cortex-adult; (Brain_normal adult; sample from Brodman area 9 (superior frontal gyrus) of a 20 year old healthy male, 6 hours postmortem); hsa_Granulocytes-CD1; (Granulocytes; Granulocyte cells from peripheral blood (pool from 4 healthy donors)); hsa_HSC-CD34 (pluripotent hematopoetic stem cell CD34+ - sorted cells); hsa_NK-CD56;(NK cells from peripheral blood (pool from 4 healthy donors)); hsa_Podocytes-Moins-undiff (Podocytes_undifferentiated); hsa_Podocytes-Moins-diff (Podocytes_differentiated); hsa_T-cell-CD4 (T helper cells, peripheral blood (pool from 4 healthy donors)); hsa_T-cell-CD4-2 (T helper cells); hsa_T-cell-CD4-naive(CD4+ CD45 RA+(CD45RO−) native cells); hsa_T-cell-CD4-effector (CD4+, CD45RO+, CD27−, CCR7−. Effector cells); hsa_T-cell-CD4-memory (CD4+CD45RO+(CD45RA−) memory cells); hsa_T-cell-CD8-2 (cytotoxic T-cells); hsa_T-cell-CD8 (cytotoxic T-cells, peripheral blood(pool from 4 healthy donors); hsa_T-cell-CD8-naive (CD8+, CD45RA+, CD27+, CCR7+); hsa_USSC (unrestricted somatic stem cells from umbilical cord); hsa_USSC-d1(unrestricted somatic stem cells from umbilical cord induced 1 day to osteoblasts); hsa_USSC-d3 (unrestricted somatic stem cells from umbilical cord induced 3 day to osteoblasts); hsa_USSC-d7 (unrestricted somatic stem cells from umbilical cord induced 7 day to osteoblasts).

Table 1 and Figure 3 list the 15 mature miRNAs identified in the human gastric cardia. The heatmap of Figure 4 summarizes the expression of these 15 miRNAs in the gastric cardia and across normal human tissues, DGE data, available in the microRNA.org databank [10], [11]. Mature miRNA expression could be classified into two groups: i) cardia-tissues: miRNAs rarely expressed in other tissues but expressed in gastric cardia, including miR-148a, miR-192, miR-200a and miR-200b; ii) quasi-ubiquitous: miRNAs expressed in many tissues and conditions, including miR-29c, miR-21, miR-24, miR-29b, miR-29a, miR-451, miR-31, miR-145, miR-26a, miR-19b and let-7b.

**Table 1:** Characteristics of the 15 most highly expressed microRNAs in human gastric cardia and number of possible targets predicted.

miRNA	isoform	arm	Read count	Target number	Highly expressed in other tissues
miR-29	c	3p	27511	2609	no
miR-21		5p	22945	2013	yes
miR-148	a	3p	19629	2830	no
miR-24	1	3p	17322	2729	no
	2	3p	17282		
miR-29	b2	3p	15099	2948	yes
	b1	3p	14965		
miR-192		5p	14417	1029	no
miR-29	a	3p	13723	2525	no
miR-451		5p	10064	3166	no
miR-145		5p	9286	603	no
miR-31		5p	7314	2192	no
miR-200	a	3p	7770	3070	no
miR-19	b2	3p	6477	3951	no
	b1	3p	6474		
miR-200	b	3p	2803	3421	no
let-7	b	5p	2800	2976	yes
miR-26	a1	5p	1064	2694	yes
	a2	5p	1061		
	b	5p	337		

### REAL TIME PCR

The ultra-deep sequencing results were validated using the singleplex Real Time PCR (RT-PCR) method on the 15 miRNAs selected to determine their expression in the gastric cardia region from 10 healthy individuals. Of the test subjects, 60% were males, the mean age was 39.1 (±12.8) years and 50% of the studied subjects tested positive for *H. pylori*, according to international criteria established for their identification [12], [13] (Table 2) (additional details are provided in **Table S2**).

**Table 2:** Putative target genes of the fourteen miRNAs analized by qRT-PCR.

miRNAs	*TargetScan's putatives targets*
hsa-miR-19	***UBN2*** *; * ***TNRC6B*** *; * ***EPS15*** *; * ***BRWD1*** *; * ***NUFIP2*** *; * ***PTEN*** *; * ***PTPRD*** *; * ***DDX6*** *; * ***IGF1*** *; * ***KIAA0355*** *; * ***CNOT6*** *; * ***GMFB*** *; * ***PLXNA4*** *; * ***ATXN1*** *; * ***DICER1*** *; * ***SCML2*** *; * ***PIK3R3*** *; * ***KLF12*** *; * ***PDIK1L*** *; * ***CREB5*** *.*
hsa-miR-29a	***ANKRD52*** *; * ***UBN2*** *; * ***TNRC6B*** *; * ***EPS15*** *; * ***NFAT5*** *; * ***BACH2*** *; * ***BRWD1*** *; * ***NUFIP2*** *; * ***PTEN*** *; * ***CDK6*** *; * ***PTPRD*** *; * ***DDX6*** *; * ***IGF1*** *; * ***KIAA2018*** *; * ***KIAA0355*** *; * ***CNOT6*** *; * ***GMFB*** *; * ***SH3PXD2A*** *; * ***KLF4*** *; * ***SLC16A2*** *.*
hsa-miR-29c	***ANKRD52*** *; * ***UBN2*** *; * ***TNRC6B*** *; * ***EPS15*** *; * ***NFAT5*** *; * ***BACH2*** *; * ***BRWD1*** *; * ***NUFIP2*** *; * ***PTEN*** *; * ***CDK6*** *; * ***PTPRD*** *; * ***DDX6*** *; * ***IGF1*** *; * ***KIAA2018*** *; * ***KIAA0355*** *; * ***CNOT6*** *; * ***GMFB*** *; * ***SH3PXD2A*** *; * ***KLF4*** *; * ***SLC16A2*** *.*
hsa-miR-29b	***ANKRD52*** *; * ***UBN2*** *; * ***TNRC6B*** *; * ***EPS15*** *; * ***NFAT5*** *; * ***BACH2*** *; * ***BRWD1*** *; * ***NUFIP2*** *; * ***PTEN*** *; * ***CDK6*** *; * ***PTPRD*** *; * ***DDX6*** *; * ***IGF1*** *; * ***KIAA2018*** *; * ***KIAA0355*** *; * ***CNOT6*** *; * ***GMFB*** *; * ***SH3PXD2A*** *; * ***KLF4*** *; * ***SLC16A2*** *.*
hsa-miR-200b	***ANKRD52*** *; * ***UBN2*** *; * ***EPS15*** *; * ***BACH2*** *; * ***BRWD1*** *; * ***NUFIP2*** *; * ***PTEN*** *; * ***DDX6*** *; * ***KIAA2018*** *; * ***KIAA0355*** *; * ***CNOT6*** *; * ***SH3PXD2A*** *; * ***KLF4*** *; * ***SLC16A2*** *; * ***PLXNA4*** *; * ***ATXN1*** *; * ***KLF12*** *; * ***SNTB2*** *; * ***PDIK1L*** *; * ***CREB5*** *.*
hsa-miR-26a	***ANKRD52*** *; * ***UBN2*** *; * ***TNRC6B*** *; * ***EPS15*** *; * ***BRWD1*** *; * ***PTEN*** *; * ***CDK6*** *; * ***PTPRD*** *; * ***IGF1*** *; * ***KIAA2018*** *; * ***GMFB*** *; * ***SH3PXD2A*** *; * ***KLF4*** *; * ***C11orf41*** *; * ***PIK3R3*** *; * ***PDIK1L*** *; * ***TP53INP1*** *; * ***CCND2*** *; * ***PTP4A1*** *; * ***MIB1*** *.*
hsa-miR-148a	***ANKRD52*** *; * ***UBN2*** *; * ***TNRC6B*** *; * ***EPS15*** *; * ***NFAT5*** *; * ***BACH2*** *; * ***PTEN*** *; * ***CDK6*** *; * ***PTPRD*** *; * ***DDX6*** *; * ***IGF1*** *; * ***CNOT6*** *; * ***GMFB*** *; * ***SH3PXD2A*** *; * ***KLF4*** *; * ***ATXN1*** *; * ***DICER1*** *; * ***SCML2*** *; * ***PIK3R3*** *; * ***SP1*** *.*
hsa-miR-200a	***ANKRD52*** *; * ***TNRC6B*** *; * ***BACH2*** *; * ***BRWD1*** *; * ***NUFIP2*** *; * ***PTEN*** *; * ***CDK6*** *; * ***PTPRD*** *; * ***PLXNA4*** *; * ***ATXN1*** *; * ***C11orf41*** *; * ***KLF12*** *; * ***PLAG1*** *; * ***TP53INP1*** *; * ***ELF2*** *; * ***CCND2*** *; * ***PTP4A1*** *; * ***MIB1*** *; * ***PHF21A*** *; TET3.*
hsa-miR-145	***ANKRD52*** *; * ***UBN2*** *; * ***EPS15*** *; * ***BACH2*** *; * ***NUFIP2*** *; * ***CDK6*** *; * ***DDX6*** *; * ***KIAA0355*** *; * ***GMFB*** *; * ***KLF4*** *; * ***SLC16A2*** *; * ***SNTB2*** *; * ***PDIK1L*** *; * ***CREB5*** *; * ***NFIB*** *; * ***SP1*** *; * ***ELF2*** *; * ***CCND2*** *; * ***PHF21A*** *; * ***ADAM19*** *.*
hsa-miR-24	***ANKRD52*** *; * ***UBN2*** *; * ***NFAT5*** *; * ***PTPRD*** *; * ***KIAA2018*** *; * ***KIAA0355*** *; * ***CNOT6*** *; * ***SH3PXD2A*** *; * ***SLC16A2*** *; * ***C11orf41*** *; * ***SCML2*** *; * ***PIK3R3*** *; * ***SP1*** *; * ***PLAG1*** *; * ***TP53INP1*** *; * ***ADAM19*** *; PHLPPL; AMMECR1; CALCR; CSNK1G1.*
hsa-miR-31	***ANKRD52*** *; * ***TNRC6B*** *; * ***NFAT5*** *; * ***BACH2*** *; * ***NUFIP2*** *; * ***KIAA2018*** *; * ***SLC16A2*** *; * ***PLXNA4*** *; * ***C11orf41*** *; * ***DICER1*** *; * ***SNTB2*** *; ELAVL2; CCNJ; CALCR; BAHD1; RHOBTB1; ZBTB34; RAPH1; SLC1A2; JAZF1.*
hsa-miR-21	***UBN2*** *; * ***TNRC6B*** *; * ***NFAT5*** *; * ***BRWD1*** *; * ***CDK6*** *; * ***SCML2*** *; * ***KLF12*** *; * ***SNTB2*** *; * ***NFIB*** *; * ***PLAG1*** *; * ***ELF2*** *; CPEB3; GLIS2; TIAM1; BAHD1; TET1; PAG1; STK38L; ZNF217; BCL11A.*
hsa-miR-192	***NFAT5*** *; * ***DDX6*** *; * ***IGF1*** *; * ***DICER1*** *; * ***CREB5*** *; C6orf168; CTNNBIP1; ZBTB34; COL5A1; PPP1R3D; NCOA3; XPO4; SLC5A3; SH3RF3; CDON; DIAPH2; DBT; FAM167A; CCNT2; IKZF2.*
hsa-miR-451	*GATAD2B; C11orf30; TSC1; VAPA; OSR1; FBXO33; CAB39; YWHAZ; PSMB8; SAMD4B; GK; AEBP2; CDKN2D; TTN.*

Putative targets of the miRNAs were predict by TargeScan 5.1. Genes marked in bold type are targets of at least two miRNAs listed above.

### COMPARARISON WITH DGE AND RT-PCR

**Figure 5:**
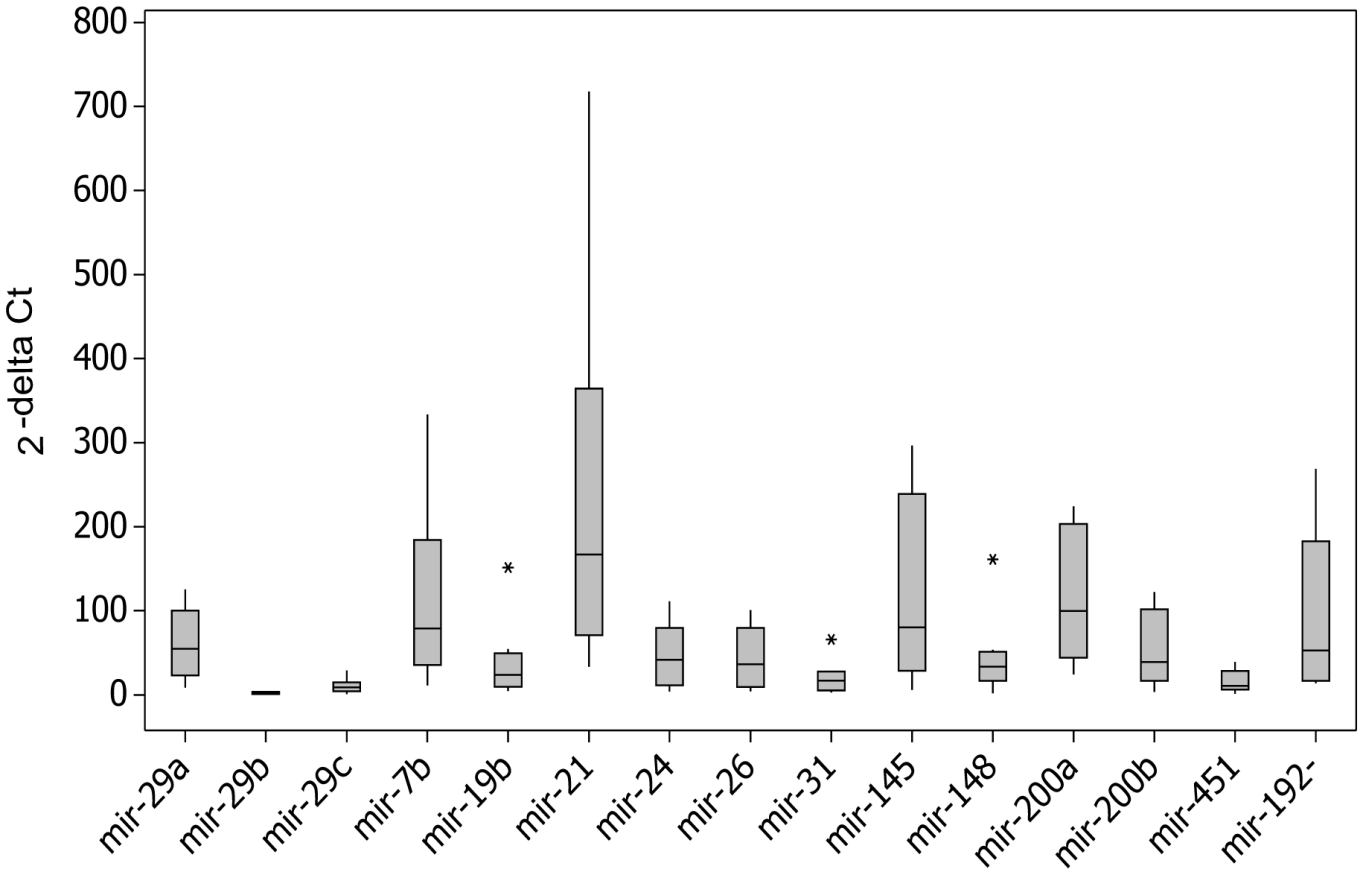
Quantification by Real Time PCR of high expressed microRNAs in human gastric cardia. The quantification is based on Ct and was normalized by endogenous expression control. The 2^−ΔCt^ for each miR is the mean of ten determination originated from gastric cardia tissue of ten different individuals.

Both singleplex (RT-PCR) and multiplex (SOLiD platform) technologies showed high expression (expression over 1,000 reads in the DGE and 7-fold above the endogenous control in the RT-PCR) of the 15 miRNAs (Figures 4 and 5).

The same RNA samples had been analyzed by two different platforms: DGE and RT-PCR - Life Technologies. The results experiments were compared and were observed linear regression between 2^−ΔCt^ and square root of read count number. Pearson correlation is high 83.9% with statistical significant test (P<0.05) and validated SOLID results.

Additionally, DGE results were compared to the quantification of the expression range of the miRNAs in 10 healthy subjects, by Real Time PCR. The regression for average of RT-PCR assays *versus* square root of read count number. Pearson correlation is high 68.4% with statistical significant test (P<0.05).

Could observe miRNAs with high interindividual variation, for exempla miR-21, and another with low interindividual variation, e.g. expression pattern slightly variable (miR-29b, miR-29c, miR-19b, miR-31, miR-148a, miR-451).

## Discussion

miRNAs regulate the majority of human genes; however, only a few miRNAs have had their targets and specific functions identified [14]. In our study, the stomach sample was obtained from a single individual without stomach neoplasia or other pre-neoplasia conditions such as atrophy, metaplasia or dysplasia. Pre-cancerous lesions such as gastritis lead to genomic hypo-methylation in the stomach that could modify the expression pattern of miRNAs [15]. The sample was obtained from the normal tissue from a patient without any pathologies, which helped in avoiding the risk of collecting a seemingly normal tissue sample with micro-invasions of early-stage tumorigenic cells as could occur in patients with any of the above pathologies.

This study is the first ultra-high-throughput sequencing of miRNAs in the physiologically normal human stomach. Only 5.06% of miRNAs identified in gastric tissue had already been detected in other tissues and cataloged in bioinformatics databanks such as microRNA.org [10]. We expect this group of miRNAs to be regulators of housekeeping genes, which are abundant in human tissues. Another 7.84% of miRNAs had no matches in the miRNA expression databases and could represent miRNAs that are specific to the digestive system or stomach.

Similar studies have been performed with other normal tissues, such as the mouth, pharynx, esophagus, anus and intestine. We compared these data with our miRNA expression data to define the expression pattern of the stomach tissue. We found high expression levels in 15 miRNAs, 13 of which had already been identified as highly expressed in other tissues.

The expression of mir-148a and mir-192 had been identified in other normal and cancerous human tissues, but was not over-expressed. Mir-192 had already been detected in gastrointestinal tissues such as the colon, ileum, duodenum, small intestine, stomach, pancreas and liver [16]. Basal expression of mir-148a has been observed in connective tissue and endocrine tissue [17]. Recently, mir-148a was found to be repressed in umbilical cord blood cells [18] and silenced by hypermethylation in colon tumors [19]. We observed high expression of the mir-200 cluster (a and b) in gastric cardia as observed in the islets of Langerhans [11]. In a microarray experiment, mir-200a and mir-200b were detected at low levels in gastrointestinal tissues but at high levels in the colon, stomach and pancreas [16]. A recently published microRNA expression atlas showed that this miRNA is characteristic to endocrine tissue [17]. Recent findings show that the low expression of the mir-200 cluster is correlated with ovarian cancer [19], [20]. Therefore, the mir-200 cluster may be important in maintaining the integrity of digestive tissues such as gastric cardia because such high expression up-regulated the expression of e-cadherin, the protein responsible for the organization of the architecture of the epithelial tissue. Additionally, the results strongly suggested an important role of the miR-200 family in the repression of the epithelial-mesenchymal transition (EMT) and the progression of cancer [21].

Table 1 and 2 show the number of possible targets for each highly expressed mature miRNA. Table 2 shows some targets of miRNAs predicted by the TargetScan against families of conserved miRNAs. With the exception of miR-451, all shared at least two other gene targets. The genes *ANKD52* and *UBN2*, are targets of ten of the fourteen miRNAs analyzed, while the gene *TNRC6B* is of nine miRNAs, and the genes *EPS15*, *NFAT5*, *BACH2*, *BRWD1*, *NUFIP2*, *PTEN*, *CDK6*, and *PTPRD DD6*, are targets of eight miRNA. This result suggests that these miRNAs are strong candidates to be silenced in cardia region. The experimental validation of these genes, followed by an analysis of the function of each one can reveal the physiological role of these miRNAs in normal gastric tissue.

Many miRNAs may regulate the translation of proteins that act in tissue proliferation and tissue patterning such as mir-200a (which may target integrin) and mir-145 (which may interact with *ERBB4* mRNA). Many predicted mRNA targets were found to be common to multiple highly expressed miRNAs. For example, the *HTR4* (5-hydroxytryptamine [serotonin] receptor) and *AFF2* mRNAs (*AF4/FMR2* family, member 2) were predicted to be targets of 13 of the 15 most highly expressed miRNAs. Another five mRNAs, including *IGF-1* (insulin-like growth factor 1 [somatomedin C]), were common predicted targets of 12 miRNAs. Eight miRNAs had 222 predicted targets in common; for example, mir-29b was predicted to target 196 of these. Five miRNAs (19b, 29a, 29b, 29c and 148a) shared 70 predicted targets, some of which (*CDK6*, *PTEN*, *IGF1*, *FRS2*, *PDGFRA*, *PIK3R1* and *MXD1*) regulate cellular proliferation and tumor suppression.

Several observations link miRNAs to cancer. First, many miRNAs are involved in cellular proliferation and apoptosis. Second, many miRNA loci are located in fragile sites of the human genome, regions that are frequently amplified or deleted in human neoplasias and cause large differences in miRNA expression compared with normal tissues [21], [22], [23], [24].

The Real Time PCR technique (which uses relative quantification) confirmed 15 miRNAs identified as showing the highest expression by the SOLiD platform (which is based on absolute numbers) (Figures 4 and 5). Therefore, these miRNAs can be considered as being over-expressed [25], [26], [27], [28]. The correlation between DGE and RT-PCR assays was clear and statistic significant. And DGE experiment could be considered representative of tissues gastric samples isolated from 10 health subjects and define part of the expression pattern of the healthy gastric tissue.

The results from both methodologies indicate that miRNA-21 was the most highly expressed in gastric cardia tissue. This miRNA is also distributed in other human tissues (e.g., dendritic cells, T-cells, pancreas) and could be involved in regulating the expression of housekeeping genes (*ATPAF1*, *KIF3A*, *CYBRD1*).

The SOLiD platform showed that miR-192 and 148a are specific to gastric tissue. In addition, Real Time PCR confirmed that these miRNAs are over-expressed. Thus, overall, these miRNAs are likely to regulate the expression of genes related to gastric tissue homeostasis. Therefore, the low expression levels of these miRNAs could be related to the development of a stomach neoplasia. The potential use of miRNA-192 and miRNA-148a as risk markers in gastric cancer could be best investigated through the analysis of the miRnomes of different histological types of gastric cancer.

Understanding the regulatory processes that act in the human stomach will be important in the fight against gastric cancer, which is the second-leading cause of cancer mortality worldwide.

## Materials and Methods

### BIOLOGICAL MATERIAL

The gastric cardia is a microscopic zone that is normally found in the most proximal portion of the stomach, close to the esophageal opening (cardiac orifice or cardia) and contains the cardiac glands. Our fresh tissue sample for ultra deep sequencing was obtained from a gastroscopic biopsy (∼4 mm^3^). The patient was 33 years old, with no evidence of cancer and a normal gastroesophageal junction. Macroscopic observation of the tissue showed no evidence of lesions, and histological examination confirmed normal and healthy conditions.

To confirm the results of ultra-deep sequencing, cardia samples, near cardiac orifice, from 10 addition, healthy individuals were also obtained by endoscopic biopsies (∼4 mm^3^) and, after histological examination excluding abnormalities, were analyzed by Real Time PCR. *H. pylori* infection was diagnosed based on the typical appearance of the bacterium along the mucus layer covering the gastric mucous membrane Antral biopsies were taken for histology evaluation due to higher density of the bacteria in gastric antrum and to the excellent sensitivity of this diagnostic method, according to international criteria established for their identification [12], [13] (additional details are provided in **Table S2**).

### ETHICS STATEMENT

Written informed consent was obtained from all patients, and the study was approved by the Comitê de Ética em Pesquisa (CEP) of Hospital Universitário João Barros Barreto (HUJBB) - Federal University of Pará (UFPA) (Protocol number 14052004/HUJBB).

### miRNA LIBRARY

The total small RNA was obtained from the sample tissue using the mirVana Isolation Kit (Ambion Inc., US). The concentration and quality were determined using a Nanodrop 1000 spectrophotometer, and purification and size selection were performed using 6% polyacrylamide gel electrophoresis. Using the SOLiD Small RNA Expression Kit (Ambion Inc., US), 200 ng of small RNA of 150–200 bp were used as a template to obtain the miRNA library. All miRNAs of the library were tagged with unique and specific amplification primers, known as the barcode system (Life Technologies, CA, US). Then, 50 pg of the library was pooled with seven other miRNA libraries at the same concentration. A fraction of the library pool (0.1 pg) was amplified and fixed on magnetic beads using emulsion PCR. The ePCR product was deposited on a single slide and subjected to the multiplex SOLiD sequencing reaction.

### SOLiD ULTRA-DEEP SEQUENCING AND DATA ANALYSIS

The SOLiD (version 2.0) sequencing system (Life Technologies) was used to generate reads that were 35 bp long. The second step was to decode the barcode, matching each bead sequence with the identity of the sample. All small RNA sequences of gastric cardia are available in the NCBI Sequences Read Archive (SRA012099). Sequence analysis was performed using the SOLiD System Small RNA Analysis Tool (Life Technologies) and MiRanalyzer [8]. First, we filtered out all sequences that matched RNA contaminants such as tRNA, rRNA, DNA repeats and adaptor molecules. After excluding contaminant reads, we aligned all sequences against miRNA precursor sequences (MirBase ver. 12) and then only included the reads that matched mature miRNA sequences [9]. To compare these expression data with those of other human tissues, miRNA expression data were imported from the microRNA.org database, and the expression of each mature miRNA was normalized by total read count [10]. Graphical analysis was performed using Genepattern^10^. The miRNA–biological process relationships were predicted using miRNApath (http://lgmb.fmrp.usp.br/mirnapath/tools.php).

### miRNA REAL TIME PCR (VALIDATION)

Biopsy samples of gastric cardia tissues were collected from 10 healthy patients. After collection, the samples were immediately processed and stored at −80°C until RNA extraction. Total RNA was extracted by homogenizing 40 milligrams of frozen tissue, followed by RNA isolation by TRIzol reagent (Life Technologies) according to the manufacturer's instructions. The concentration and quality of miRNAs were determined using a Nanodrop 1000 spectrophotometer (ND-1000; Nanodrop Technologies, Wilmington, DE). Total RNA was reverse transcribed using a TaqMan@MicroRNA Reverse Transcription kit (Life Technologies).

The analysis of miRNAs levels was performed on a 7500 Real-Time PCR System (Life Technologies) with TaqMan miRNA assays according to the manufacturer's instructions (Life Technologies) using primers designed with Primer Express (Life Technologies). The mean expression level of three human endogenous controls (Z30, RNU19 and RNU6B - calibrators) was used as an internal control in all miRNA experiments to allow for the comparison of expression results.

The high expression levels of miRNAs identified by ultra-deep sequencing (in descending order: miR-29c, miR-21, miR-148a, miR-29a, miR-24, miR-29b, miR-192, miR-451, miR-145, miR-31, miR-200a, miR-19b, miR-200b, let-7b and miR-26a) were validated with the TaqMan miRNA assays (Life Technologies). These analyses were used to measure the expression levels of mature miRNAs and the inter-individual variation in the 10 samples of healthy cardia tissue. The expression data of each mature miRNA were normalized to the mean expression level of the three human endogenous controls (Z30, RNU19 and RNU6B). The relative miRNA expression levels were then calculated by the comparative threshold cycle (Ct) method (2^−ΔCt^). Correlation test was performed using Pearson method (SPSS v.12).

## Supporting Information

Table S1 Identity and abundance data for all known miRNAs in SOLiD sequence dataset in human stomach and miRamda database.
(0.44 MB PDF)

Table S2 Description and RT-PCR results of 10 samples from healthy individuals obtained by endoscopic biopsies. Samples size is about ∼4 mm3. For all samples were performed a histological examination and H. pylori detection.
(0.03 MB XLS)
