# Supplementary material for: Ultra-Deep Sequencing Reveals the microRNA Expression Pattern of the Human Stomach
Source: PLoS One. 2010 Oct 8;5(10):e13205. doi: 10.1371/journal.pone.0013205 (PMC2951895; doi:10.1371/journal.pone.0013205)
Supplement: Table S1 — Identity and abundance data for all known miRNAs in SOLiD sequence dataset in human stomach and miRamda database. (0.44 MB PDF) [file pone.0013205.s001.pdf]

matureform	miR_Ac	miR_Ac	miR_Al	miR_Al	miR_Al	miR_Al	miR_Al	miR_Al	miR_Al	miR_Al	miR_Al	miR_As	miR_As	miR_As	miR_B-	miR_B-	miR_B-	miR_B-	miR_B-
miR-let-7a	0.03	0.03	0.03	0.03	0.03	0	0	0.02	0	0.03	0.01	0.02	0.05	0.04	0.04	0.01	0.04	0.01	0.04
miR-let-7b	0.01	0.01	0.01	0	0	0.03	0	0.03	0.01	0.02	0.01	0.05	0.05	0.05	0	0	0.01	0.01	0.02
miR-let-7c	0.01	0	0	0	0	0.02	0	0.02	0	0	0	0	0.01	0.02	0.01	0.01	0	0	0
miR-let-7d	0	0	0.01	0.01	0	0	0	0	0	0.01	0	0	0	0	0.01	0	0.01	0	0
miR-let-7e	0	0	0	0	0	0	0	0	0	0	0	0	0.01	0.01	0.01	0	0	0	0
miR-let-7f	0	0	0.02	0.02	0.01	0	0	0	0	0.01	0	0	0.01	0	0.03	0.01	0.01	0	0.01
miR-let-7g	0	0	0.01	0	0.01	0	0	0	0	0	0	0	0	0	0	0	0.01	0	0
miR-let-7i	0	0	0	0	0	0	0	0	0	0	0	0	0	0	0.02	0.01	0	0	0.01
miR-1	0	0	0	0	0	0	0	0	0	0	0	0	0	0	0	0	0	0	0
miR-100	0	0	0	0	0	0.01	0	0	0	0	0	0.01	0	0.01	0	0	0	0	0
miR-100871	0	0	0	0	0	0	0	0	0	0	0	0	0	0	0	0	0	0	0
miR-100871	0	0	0	0	0	0	0	0	0	0	0	0	0	0	0	0	0	0	0
miR-101	0	0	0	0	0.01	0	0	0	0	0.01	0	0	0	0.01	0	0.01	0	0	0
miR-103	0	0	0	0.01	0.01	0	0	0	0	0	0	0	0	0	0	0	0	0	0
miR-105	0	0	0	0	0	0	0	0	0	0	0	0	0	0	0	0	0	0	0
miR-106a	0.01	0.01	0	0	0	0	0	0	0	0	0	0	0	0	0	0	0	0	0
miR-106b	0.01	0	0	0.01	0.02	0	0	0	0.01	0	0	0	0	0	0	0	0	0.01	0.01
miR-107	0	0	0	0	0	0	0	0	0	0	0	0	0	0	0	0	0	0	0
miR-10a	0	0	0	0	0	0	0	0	0	0	0	0	0	0	0	0	0	0	0
miR-10b	0	0	0	0	0	0	0	0	0	0	0	0	0	0	0	0	0	0	0
miR-122	0	0	0	0	0	0.05	0	0.03	0	0	0	0	0.01	0.01	0	0	0	0	0
miR-124	0	0	0.01	0	0	0	0	0	0	0	0	0	0.22	0.01	0	0	0	0	0
miR-125a	0	0	0	0	0	0	0	0	0	0	0	0	0	0	0	0	0	0	0
miR-125a	0	0	0	0	0	0	0	0	0	0	0	0	0	0	0	0	0	0	0
miR-125b	0	0	0	0	0	0.04	0	0.01	0	0	0	0.06	0.03	0.04	0	0	0	0	0
miR-126	0	0	0	0.08	0	0.01	0.04	0.02	0.03	0.02	0.07	0.1	0.03	0.09	0.06	0.26	0.02	0.04	0.09
miR-127	0	0	0	0	0	0	0	0	0	0	0	0	0	0	0	0	0	0	0
miR-127	0	0	0	0	0	0	0	0	0	0	0	0	0	0	0	0	0	0	0
miR-128a	0	0	0	0	0	0	0	0	0	0	0	0	0	0	0	0	0	0	0
miR-128b	0	0	0	0	0	0	0	0	0	0	0	0	0	0	0	0	0.01	0	0
miR-129	0	0	0	0	0	0	0	0	0	0	0	0	0	0	0	0	0	0	0
miR-129	0	0	0	0	0	0	0	0	0	0	0	0	0	0	0	0	0	0	0
miR-129	0	0	0	0	0	0	0	0	0	0	0	0	0	0	0	0	0	0	0
miR-130a	0.05	0.06	0	0.02	0	0.02	0.01	0.01	0	0	0	0.02	0.01	0.01	0.02	0	0	0	0.01
miR-130b	0.01	0.01	0	0	0	0	0	0	0	0	0	0	0	0	0.01	0	0	0	0

miR-132	0	0	0	0	0	0	0	0	0	0	0	0	0	0	0	0	0	0	0
miR-133a	0	0	0	0	0	0	0	0	0	0	0	0	0	0	0	0	0	0	0
miR-133b	0	0	0	0	0	0	0	0	0	0	0	0	0	0	0	0	0	0	0
miR-134	0	0	0	0	0	0	0	0	0	0	0	0	0	0	0	0	0	0	0
miR-135a	0	0	0	0	0	0	0	0	0	0	0	0	0	0	0	0	0	0	0
miR-135b	0	0	0	0	0	0	0	0	0	0	0	0.01	0	0	0	0	0	0	0
miR-136	0	0	0	0	0	0	0	0	0	0	0	0.01	0.01	0.01	0	0	0	0	0
miR-137	0.06	0.06	0	0	0	0	0	0	0	0	0	0	0	0	0	0	0	0	0
miR-138	0	0	0	0	0	0	0	0	0	0	0	0	0	0.01	0	0	0	0	0
miR-139	0	0	0	0	0	0	0	0	0	0	0	0	0	0	0	0	0	0	0
miR-139	0	0	0	0	0	0	0	0	0	0	0	0	0	0	0	0	0	0	0
miR-140	0	0	0	0	0	0	0	0	0	0	0	0	0	0	0	0	0	0	0
miR-140	0	0	0.01	0	0.01	0	0.01	0.01	0	0.01	0.01	0.01	0.01	0.01	0	0	0.01	0	0.01
miR-141	0	0	0	0	0	0	0	0.01	0	0	0	0	0	0	0	0	0	0	0
miR-142	0	0	0.13	0.19	0.15	0	0.12	0.04	0.01	0.08	0.08	0	0.01	0.01	0.07	0.16	0.1	0.11	0.1
miR-142	0	0	0.3	0.14	0.09	0.01	0.17	0.15	0.04	0.34	0.18	0.02	0.01	0	0.18	0.1	0.19	0.16	0.31
miR-143	0	0	0.01	0	0	0.01	0	0.04	0	0	0	0.02	0.01	0.02	0	0	0	0	0
miR-144	0	0	0	0	0	0	0.16	0	0.28	0.02	0.04	0.01	0.02	0.02	0	0.01	0.02	0.09	0
miR-145	0	0	0	0	0	0	0	0	0	0	0	0	0	0	0	0	0	0	0
miR-146a	0	0	0	0.01	0.01	0	0	0	0	0	0	0	0	0	0	0.02	0	0	0.01
miR-146b	0	0	0	0	0	0	0	0	0	0	0	0	0	0	0	0	0	0	0
miR-146b	0	0	0	0	0	0	0	0	0	0	0	0	0	0	0	0	0	0	0
miR-147a	0	0	0	0	0	0	0	0	0	0	0	0	0	0	0	0	0	0	0
miR-147b	0	0	0	0	0	0	0	0	0	0	0	0	0	0	0	0	0	0	0
miR-148a	0	0	0	0	0	0	0	0	0	0	0	0	0	0	0	0	0	0	0
miR-148b	0	0	0	0	0	0	0	0	0	0	0	0	0	0	0	0	0	0	0
miR-149	0	0	0	0	0	0	0	0	0	0	0	0	0	0	0	0	0	0	0
miR-150	0	0	0	0	0	0	0	0	0	0	0	0	0	0	0.01	0	0	0	0.01
miR-151	0	0	0	0.01	0	0	0	0	0	0	0	0	0	0	0	0	0	0	0
miR-151	0.01	0.01	0	0.01	0	0	0	0	0	0	0	0	0	0	0	0	0	0	0
miR-152	0	0	0	0	0	0	0	0	0	0	0	0	0	0	0	0	0	0	0
miR-153	0	0	0	0	0	0	0	0	0	0	0	0.02	0	0	0	0	0	0	0
miR-154	0	0	0	0	0	0	0	0	0	0	0	0	0	0	0	0	0	0	0
miR-155	0	0	0	0	0	0	0	0	0	0	0	0	0	0	0	0	0	0	0
miR-15a	0.01	0.01	0.05	0.07	0.06	0.1	0.08	0.08	0.09	0.05	0.12	0.03	0.01	0.03	0.04	0.02	0.08	0.11	0.03
miR-15b	0.01	0.01	0.01	0.01	0.02	0	0	0.01	0	0	0	0	0	0	0.01	0.01	0	0	0

miR-16	0.09	0.07	0.12	0.1	0.14	0.22	0.16	0.14	0.34	0.1	0.19	0.05	0.03	0.03	0.17	0.09	0.1	0.21	0.08
miR-17	0.02	0.02	0.01	0.01	0.01	0	0.01	0	0.01	0	0.01	0	0	0	0.02	0.03	0.01	0.02	0
miR-181a	0	0	0.02	0	0	0	0	0.01	0	0.06	0.01	0.02	0.01	0.02	0.01	0.02	0.13	0	0.02
miR-181b	0	0	0	0.01	0	0	0	0	0	0.01	0	0	0	0	0	0	0.01	0	0
miR-181c	0	0	0	0	0	0	0	0	0	0	0	0	0	0	0	0	0	0	0
miR-181d	0	0	0	0	0	0	0	0	0	0	0	0	0	0	0	0	0	0	0
miR-182	0	0	0	0	0	0	0	0	0	0	0	0	0	0	0	0	0	0	0
miR-183	0	0	0	0	0	0	0	0	0	0	0	0	0	0	0	0	0	0	0
miR-184	0	0	0	0	0	0	0	0	0	0	0	0	0	0	0	0	0	0	0
miR-185	0	0	0	0	0	0	0.01	0	0	0	0	0	0	0	0	0	0	0.01	0
miR-186	0	0	0	0	0	0	0	0	0	0	0	0	0	0	0.01	0.01	0	0	0.01
miR-187	0	0	0	0	0	0	0	0	0	0	0	0	0	0	0	0	0	0	0
miR-188	0	0	0	0	0	0	0	0	0	0	0	0	0	0	0	0	0	0	0
miR-188	0	0	0	0	0	0	0	0	0	0	0	0	0	0	0	0	0	0	0
miR-18a	0.01	0.01	0	0	0.01	0	0.01	0	0	0	0	0	0	0	0.01	0	0	0	0
miR-18b	0	0.01	0	0	0	0	0	0	0	0	0	0	0	0	0	0	0	0	0
miR-190	0	0	0	0	0	0	0	0	0	0	0	0	0	0	0	0	0	0	0
miR-190b	0	0	0	0	0	0	0	0	0	0	0	0	0	0	0	0	0	0	0
miR-191	0.01	0.01	0.03	0.02	0.04	0.01	0	0.03	0	0.03	0.01	0	0	0	0.01	0	0.05	0.01	0.02
miR-192	0	0	0	0	0	0	0	0	0	0	0	0	0	0	0	0	0	0	0
miR-193a	0	0	0	0	0	0	0	0	0	0	0	0	0	0	0	0	0	0	0
miR-193a	0	0	0	0	0	0	0	0	0	0	0	0	0	0	0	0	0	0	0
miR-193b	0	0	0	0	0	0	0	0	0	0	0	0	0	0	0	0	0	0	0
miR-194	0	0	0	0	0	0	0	0	0	0	0	0	0	0	0	0	0	0	0
miR-195	0	0	0	0	0	0.01	0	0.01	0	0	0	0.01	0	0	0	0	0	0	0
miR-196a	0.01	0.01	0	0	0	0	0	0	0	0	0	0	0	0	0	0	0	0	0
miR-196b	0	0	0	0	0	0	0	0	0	0	0	0	0	0	0.01	0	0.01	0	0
miR-197	0	0	0	0	0	0	0	0	0	0	0	0	0	0	0	0	0	0	0
miR-198	0	0	0	0	0	0	0	0	0	0	0	0	0	0	0	0	0	0	0
miR-199a	0	0	0	0	0	0	0	0	0	0	0	0	0	0	0	0	0	0	0
miR-199a	0	0	0	0	0	0	0	0	0	0	0	0	0	0	0	0	0	0	0
miR-199b	0	0	0	0	0	0	0	0	0	0	0	0	0	0	0	0	0	0	0
miR-199b	0	0	0	0	0	0	0	0	0	0	0	0	0	0	0	0	0	0	0
miR-19a	0.01	0	0	0.01	0.01	0	0	0	0	0	0	0	0	0	0.01	0.01	0	0	0
miR-19b	0.01	0.01	0.01	0	0.04	0	0	0	0	0	0	0	0	0	0.02	0.02	0.01	0	0.01
miR-200a	0	0	0	0	0	0	0	0	0	0	0	0	0	0	0	0	0	0	0

miR-200b	0	0	0	0	0	0	0	0	0	0	0	0	0	0	0	0	0	0	0
miR-200c	0	0	0	0	0	0	0	0	0	0	0	0	0	0	0	0	0	0	0
miR-202	0	0	0	0	0	0	0	0	0	0	0	0	0	0	0	0	0	0	0
miR-203	0	0	0	0	0	0	0	0	0	0	0	0	0	0	0	0	0	0	0
miR-204	0	0	0	0	0	0	0	0	0	0	0	0	0	0	0	0	0	0	0
miR-205	0	0	0	0	0	0	0	0	0	0	0	0	0	0	0	0	0	0	0
miR-206	0	0	0	0	0	0	0	0	0	0	0	0	0	0	0	0	0	0	0
miR-208	0	0	0	0	0	0	0	0	0	0	0	0	0	0	0	0	0	0	0
miR-208b	0	0	0	0	0	0	0	0	0	0	0	0	0	0	0	0	0	0	0
miR-20a	0.01	0.01	0.01	0.03	0.02	0	0.01	0	0.01	0.01	0	0	0	0	0.01	0.01	0.01	0	0.01
miR-20b	0.02	0.03	0	0	0	0	0	0	0	0	0	0	0	0	0	0	0	0	0
miR-21	0.19	0.16	0	0	0.01	0.02	0.01	0.02	0	0.05	0.01	0.08	0.01	0.02	0.01	0.01	0.01	0.02	0.01
miR-210	0	0	0	0	0	0	0	0	0	0	0	0	0	0	0	0	0	0	0
miR-211	0	0	0	0	0	0	0	0	0	0	0	0	0	0	0	0	0	0	0
miR-212	0	0	0	0	0	0	0	0	0	0	0	0	0	0	0	0	0	0	0
miR-214	0	0.01	0	0	0	0	0	0	0	0	0	0	0	0	0	0	0	0	0
miR-215	0	0	0	0	0	0	0	0	0	0	0	0	0	0	0	0	0	0	0
miR-216a	0	0	0	0	0	0	0	0	0	0	0	0	0	0	0	0	0	0	0
miR-216b	0	0	0	0	0	0	0	0	0	0	0	0	0	0	0	0	0	0	0
miR-217	0	0	0	0	0	0	0	0	0	0	0	0	0	0	0	0	0	0	0
miR-218	0	0	0	0	0	0	0	0	0	0	0	0	0	0	0	0	0	0	0
miR-219	0	0	0	0	0	0	0	0	0	0	0	0	0	0	0	0	0	0	0
miR-219	0	0	0	0	0	0	0	0	0	0	0	0	0	0	0	0	0	0	0
miR-22	0.01	0	0	0	0	0.02	0	0.01	0.01	0	0.01	0.01	0.01	0.03	0	0	0	0	0.01
miR-220	0	0	0	0	0	0	0	0	0	0	0	0	0	0	0	0	0	0	0
miR-220b	0	0	0	0	0	0	0	0	0	0	0	0	0	0	0	0	0	0	0
miR-220c	0	0	0	0	0	0	0	0	0	0	0	0	0	0	0	0	0	0	0
miR-221	0.01	0.01	0	0	0	0	0	0	0	0	0	0	0	0	0	0	0	0	0
miR-222	0	0	0	0	0	0	0	0	0	0	0	0	0	0	0	0	0	0	0
miR-223	0	0	0.02	0.01	0	0	0	0.01	0	0	0.01	0	0	0	0	0.01	0	0	0
miR-224	0	0	0	0	0	0	0	0	0	0	0	0	0	0	0	0	0	0	0
miR-23a	0.01	0.02	0.01	0	0.01	0	0	0.01	0	0	0	0	0	0	0	0	0	0	0
miR-23b	0	0	0	0	0	0.01	0	0.01	0	0	0	0	0	0	0	0	0	0	0
miR-24	0.02	0.04	0.01	0.01	0.01	0.02	0	0.02	0	0	0.01	0	0.01	0	0.02	0.01	0.01	0.01	0
miR-25	0	0.01	0.01	0	0	0.01	0	0	0.01	0	0	0	0	0	0.02	0	0	0	0
miR-26a	0.01	0.01	0	0.01	0.01	0	0.01	0.01	0	0	0.01	0.01	0	0	0.01	0	0	0	0

miR-26b	0.02	0.01	0	0	0.01	0	0	0	0	0	0	0	0	0	0	0.01	0	0	0.01
miR-27a	0.01	0.02	0.03	0.03	0.01	0.02	0.01	0.05	0	0	0.03	0.01	0.01	0.01	0	0.01	0.01	0.02	0
miR-27b	0	0	0	0	0	0.02	0	0.02	0	0	0	0	0	0	0	0	0	0	0
miR-28	0	0	0	0	0	0	0	0	0	0	0	0	0	0	0	0	0	0	0
miR-28	0	0	0	0	0	0	0	0	0	0	0.01	0.01	0	0.01	0	0	0	0	0.01
miR-296	0	0	0	0	0	0	0	0	0	0	0	0	0	0	0	0	0	0	0
miR-296	0	0	0	0	0	0	0	0	0	0	0	0	0	0	0	0	0	0	0
miR-298	0	0	0	0	0	0	0	0	0	0	0	0	0	0	0	0	0	0	0
miR-299	0	0	0	0	0	0	0	0	0	0	0	0	0	0	0	0	0	0	0
miR-299	0	0	0	0	0	0	0	0	0	0	0	0	0	0	0	0	0	0	0
miR-29a	0.01	0.01	0	0	0	0	0	0.01	0	0	0	0.03	0.01	0.01	0	0	0	0	0.01
miR-29b	0.01	0.01	0.02	0.01	0.01	0.02	0	0.01	0	0.01	0.01	0.01	0.03	0.04	0.02	0	0.01	0	0.02
miR-29c	0	0	0	0	0	0	0	0	0	0	0	0	0	0	0	0	0	0	0
miR-300	0	0	0	0	0	0	0	0	0	0	0	0	0	0	0	0	0	0	0
miR-301a	0.02	0.02	0	0	0	0	0	0	0	0	0	0	0	0	0	0	0	0	0
miR-301b	0	0.01	0	0	0	0	0	0	0	0	0	0	0	0	0.01	0	0	0	0
miR-302a	0	0	0	0	0	0	0	0	0	0	0	0	0	0	0	0	0	0	0
miR-302b	0	0	0	0	0	0	0	0	0	0	0	0	0	0	0	0	0	0	0
miR-302c	0	0	0	0	0	0	0	0	0	0	0	0	0	0	0	0	0	0	0
miR-302d	0	0	0	0	0	0	0	0	0	0	0	0	0	0	0	0	0	0	0
miR-30a	0.05	0.05	0	0	0	0.01	0	0	0	0	0	0.01	0.01	0.01	0	0	0	0	0
miR-30b	0	0	0	0.01	0	0	0	0	0	0	0	0	0	0	0	0	0	0	0
miR-30c	0.04	0.02	0	0	0.02	0	0	0	0	0	0	0	0	0	0.01	0	0	0	0
miR-30d	0	0	0.01	0.02	0	0.01	0.01	0	0.01	0	0.01	0.02	0.01	0.01	0.01	0.01	0.01	0	0.01
miR-30e	0	0	0	0	0.05	0.01	0.01	0.01	0	0.03	0.01	0.02	0.01	0.01	0	0.02	0.02	0	0.01
miR-31	0	0	0	0	0	0	0	0	0	0	0	0	0	0	0	0	0	0	0
miR-32	0	0	0.01	0.02	0.01	0	0.01	0.01	0	0.01	0	0.01	0.01	0.01	0	0.01	0.01	0.01	0.01
miR-320	0.01	0	0	0	0	0	0	0	0	0	0	0	0	0.01	0	0	0	0	0
miR-323	0	0	0	0	0	0	0	0	0	0	0	0	0	0	0	0	0	0	0
miR-323	0	0	0	0	0	0	0	0	0	0	0	0	0	0	0	0	0	0	0
miR-324	0	0	0	0	0	0	0	0	0	0	0	0	0	0	0	0	0	0	0
miR-324	0	0.01	0	0	0	0	0	0	0	0	0	0	0	0	0	0	0	0	0
miR-325	0	0	0	0	0	0	0	0	0	0	0	0	0	0	0	0	0	0	0
miR-325	0	0	0	0	0	0	0	0	0	0	0	0	0	0	0	0	0	0	0
miR-326	0	0	0	0.01	0	0	0	0	0	0	0	0	0	0	0	0	0	0	0
miR-328	0	0	0	0	0	0	0	0	0	0	0	0	0	0	0	0	0	0	0

miR-329	0	0	0	0	0	0	0	0	0	0	0	0	0	0	0	0	0	0	0
miR-330	0	0	0	0	0	0	0	0	0	0	0	0	0	0	0	0	0	0	0
miR-330	0	0	0	0	0	0	0	0	0	0	0	0	0	0	0	0	0	0	0
miR-331	0	0	0	0	0	0	0	0	0	0	0	0	0	0	0	0	0	0	0
miR-331	0	0	0	0	0	0	0	0	0	0	0	0	0	0	0	0	0	0	0
miR-335	0	0	0	0	0	0	0	0	0	0	0	0	0	0	0	0	0	0	0
miR-337	0	0	0	0	0	0	0	0	0	0	0	0	0	0	0	0	0	0	0
miR-337	0	0	0	0	0	0	0	0	0	0	0	0	0	0	0	0	0	0	0
miR-338	0	0	0	0	0	0	0	0	0	0	0	0	0	0	0	0	0	0	0
miR-338	0	0	0	0	0	0	0	0	0	0	0	0	0	0	0	0	0	0	0
miR-339	0	0	0	0	0	0	0	0	0	0	0	0	0	0	0	0	0	0	0
miR-339	0	0	0	0	0	0	0	0	0	0	0	0	0	0	0	0	0	0	0
miR-33a	0	0	0	0	0	0	0	0	0	0	0	0	0	0	0	0	0	0	0
miR-33b	0	0	0	0	0	0	0	0	0	0	0	0	0	0	0	0	0	0	0
miR-340	0	0	0	0.01	0	0	0	0	0	0	0	0	0	0	0	0	0	0	0
miR-342	0	0	0	0	0	0	0	0	0	0	0	0	0	0	0	0	0	0	0
miR-342	0	0	0	0	0	0	0	0	0	0	0	0	0	0	0	0	0	0	0
miR-345	0	0	0	0	0	0	0	0	0	0	0	0	0	0	0	0	0	0	0
miR-346	0	0	0	0	0	0	0	0	0	0	0	0	0	0	0	0	0	0	0
miR-34a	0	0	0	0	0	0	0	0	0	0	0	0.01	0	0	0	0	0	0	0
miR-34b	0	0	0	0	0	0	0	0	0	0	0	0	0	0	0	0	0	0	0
miR-34c	0	0	0	0	0	0	0	0	0	0	0	0	0	0	0	0	0	0	0
miR-34c	0	0	0	0	0	0	0	0	0	0	0	0	0	0	0	0	0	0	0
miR-361	0	0	0	0	0	0	0	0	0	0	0	0	0	0	0	0	0	0	0
miR-361	0	0	0	0	0	0	0	0	0	0	0	0	0	0	0	0	0	0	0
miR-362	0	0	0	0	0	0	0	0	0	0	0	0	0	0	0	0	0	0	0
miR-362	0	0	0	0	0	0	0	0	0	0	0	0	0	0	0	0	0	0	0
miR-363	0.04	0.02	0	0	0	0	0	0	0	0	0	0	0	0	0.01	0	0	0	0
miR-365	0	0	0	0	0	0	0	0	0	0	0	0	0	0	0	0	0	0	0
miR-367	0	0	0	0	0	0	0	0	0	0	0	0	0	0	0	0	0	0	0
miR-368	0	0	0	0	0	0	0	0	0	0	0	0	0	0	0	0	0	0	0
miR-369	0	0	0	0	0	0	0	0	0	0	0	0	0	0	0	0	0	0	0
miR-369	0	0	0	0	0	0	0	0	0	0	0	0	0	0	0	0	0	0	0
miR-370	0	0	0	0	0	0	0	0	0	0	0	0	0	0	0	0	0	0	0
miR-371	0	0	0	0	0	0	0	0	0	0	0	0	0	0	0	0	0	0	0
miR-371	0	0	0	0	0	0	0	0	0	0	0	0	0	0	0	0	0	0	0

miR-372	0	0	0	0	0	0	0	0	0	0	0	0	0	0	0	0	0	0	0
miR-373	0	0	0	0	0	0	0	0	0	0	0	0	0	0	0	0	0	0	0
miR-374a	0	0	0	0	0	0	0	0	0	0	0	0	0	0	0	0	0	0	0
miR-374b	0	0	0	0.01	0	0	0	0	0	0	0	0	0	0	0	0	0	0	0
miR-375	0	0	0	0	0	0	0	0	0	0	0	0	0	0	0	0	0	0	0
miR-376a	0	0	0	0	0	0	0	0	0	0	0	0	0	0	0	0	0	0	0
miR-376b	0	0	0	0	0	0	0	0	0	0	0	0	0	0	0	0	0	0	0
miR-377	0	0	0	0	0	0	0	0	0	0	0	0	0	0	0	0	0	0	0
miR-378	0.01	0.01	0.01	0	0	0	0	0	0	0	0	0	0	0	0	0	0	0	0
miR-379	0	0	0	0	0	0	0	0	0	0	0	0	0	0	0	0	0	0	0
miR-380	0	0	0	0	0	0	0	0	0	0	0	0	0	0	0	0	0	0	0
miR-381	0	0	0	0	0	0	0	0	0	0	0	0	0	0	0	0	0	0	0
miR-382	0	0	0	0	0	0	0	0	0	0	0	0	0	0	0	0	0	0	0
miR-383	0	0	0	0	0	0	0	0	0	0	0	0	0	0	0	0	0	0	0
miR-384	0	0	0	0	0	0	0	0	0	0	0	0	0	0	0	0	0	0	0
miR-384	0	0	0	0	0	0	0	0	0	0	0	0	0	0	0	0	0	0	0
miR-409	0	0	0	0	0	0	0	0	0	0	0	0	0	0	0	0	0	0	0
miR-409	0	0	0	0	0	0	0	0	0	0	0	0	0	0	0	0	0	0	0
miR-410	0	0	0	0	0	0	0	0	0	0	0	0	0	0	0	0	0	0	0
miR-411	0	0	0	0	0	0	0	0	0	0	0	0	0	0	0	0	0	0	0
miR-412	0	0	0	0	0	0	0	0	0	0	0	0	0	0	0	0	0	0	0
miR-421	0	0	0	0	0	0	0	0	0	0	0	0	0	0	0	0	0	0	0
miR-422a	0	0	0	0	0	0	0	0	0	0	0	0	0	0	0	0	0	0	0
miR-423	0	0	0.01	0	0	0	0	0	0	0	0	0	0	0.01	0	0	0	0	0
miR-423	0	0	0	0	0	0	0	0	0	0	0	0	0	0	0	0	0	0	0
miR-424	0	0	0.01	0.01	0	0	0.01	0	0	0	0.02	0	0	0	0.01	0	0	0.03	0
miR-425	0	0	0.01	0.02	0.01	0	0.01	0	0	0	0.01	0	0	0	0.02	0.01	0.01	0.01	0.01
miR-429	0	0	0	0	0	0	0	0	0	0	0	0	0	0	0	0	0	0	0
miR-431	0	0	0	0	0	0	0	0	0	0	0	0	0	0	0	0	0	0	0
miR-432	0	0	0	0	0	0	0	0	0	0	0	0	0	0	0	0	0	0	0
miR-433	0	0	0	0	0	0	0	0	0	0	0	0	0	0	0	0	0	0	0
miR-448	0	0	0	0	0	0	0	0	0	0	0	0	0	0	0	0	0	0	0
miR-449a	0	0	0	0	0	0	0	0	0	0	0	0	0	0	0	0	0	0	0
miR-449b	0	0	0	0	0	0	0	0	0	0	0	0	0	0	0	0	0	0	0
miR-450a	0	0	0	0	0	0	0	0	0	0	0	0	0	0	0	0	0	0	0
miR-450b	0	0	0	0	0	0	0	0	0	0	0	0	0	0	0	0	0	0	0

miR-450b	0	0	0	0	0	0	0	0	0	0	0	0	0	0	0	0	0	0	0
miR-451	0	0	0	0	0	0	0.01	0	0.09	0	0.01	0.01	0.02	0.03	0	0	0	0.02	0
miR-452	0	0	0	0	0	0	0	0	0	0	0	0	0	0	0	0	0	0	0
miR-453	0	0	0	0	0	0	0	0	0	0	0	0	0	0	0	0	0	0	0
miR-454	0	0	0	0	0	0	0	0	0	0	0	0	0	0	0	0	0	0	0
miR-455	0	0	0	0	0	0	0	0	0	0	0	0	0	0	0	0	0	0	0
miR-455	0	0	0	0	0	0	0	0	0	0	0	0	0	0	0	0	0	0	0
miR-483	0	0	0	0	0	0	0	0	0	0	0	0	0	0	0	0	0	0	0
miR-483	0	0	0	0	0	0	0	0	0	0	0	0	0	0	0	0	0	0	0
miR-484	0	0	0	0	0	0	0	0	0	0	0	0	0	0	0	0	0	0	0
miR-485	0	0	0	0	0	0	0	0	0	0	0	0	0	0	0	0	0	0	0
miR-485	0	0	0	0	0	0	0	0	0	0	0	0	0	0	0	0	0	0	0
miR-486	0	0	0	0	0	0	0	0	0	0	0	0	0	0	0	0	0	0	0
miR-486	0	0	0	0	0	0	0	0	0	0	0	0	0	0	0	0	0	0	0
miR-487a	0	0	0	0	0	0	0	0	0	0	0	0	0	0	0	0	0	0	0
miR-487b	0	0	0	0	0	0	0	0	0	0	0	0	0	0	0	0	0	0	0
miR-488	0	0	0	0	0	0	0	0	0	0	0	0.01	0.01	0.01	0	0	0	0	0
miR-489	0	0	0	0	0	0	0	0	0	0	0	0	0	0	0	0	0	0	0
miR-490	0	0	0	0	0	0	0	0	0	0	0	0	0	0	0	0	0	0	0
miR-491	0	0	0	0	0	0	0	0	0	0	0	0	0	0	0	0	0	0	0
miR-491	0	0	0	0	0	0	0	0	0	0	0	0	0	0	0	0	0	0	0
miR-492	0	0	0	0	0	0	0	0	0	0	0	0	0	0	0	0	0	0	0
miR-493	0	0	0	0	0	0	0	0	0	0	0	0	0	0	0	0	0	0	0
miR-494	0	0	0	0	0	0	0	0	0	0	0	0	0	0	0	0	0	0	0
miR-495	0	0	0	0	0	0	0	0	0	0	0	0	0	0	0	0	0	0	0
miR-496	0	0	0	0	0	0	0	0	0	0	0	0	0	0	0	0	0	0	0
miR-497	0	0	0	0	0	0	0	0	0	0	0	0	0	0	0	0	0	0	0
miR-498	0	0	0	0	0	0	0	0	0	0	0	0	0	0	0	0	0	0	0
miR-499	0	0	0	0	0	0	0	0	0	0	0	0	0	0	0	0	0	0	0
miR-499	0	0	0	0	0	0	0	0	0	0	0	0	0	0	0	0	0	0	0
miR-500	0	0	0	0	0	0	0	0	0	0	0	0	0	0	0	0	0	0	0
miR-501	0	0	0	0	0	0	0	0	0	0	0	0	0	0	0	0	0	0	0
miR-501	0	0	0	0	0	0	0	0	0	0	0	0	0	0	0	0	0	0	0
miR-502	0	0	0	0	0	0	0	0	0	0	0	0	0	0	0	0	0	0	0
miR-503	0	0	0	0	0	0	0	0	0	0	0	0	0	0	0	0	0	0	0
miR-504	0	0	0	0	0	0	0	0	0	0	0	0	0	0	0	0	0	0	0

miR-505	0	0	0	0	0	0	0.01	0	0	0	0	0	0	0	0	0	0	0	0
miR-506	0	0	0	0	0	0	0	0	0	0	0	0	0	0	0	0	0	0	0
miR-507	0	0	0	0	0	0	0	0	0	0	0	0	0	0	0	0	0	0	0
miR-508	0	0	0	0	0	0	0	0	0	0	0	0	0	0	0	0	0	0	0
miR-508	0	0	0	0	0	0	0	0	0	0	0	0	0	0	0	0	0	0	0
miR-509	0	0	0	0	0	0	0	0	0	0	0	0	0	0	0	0	0	0	0
miR-509	0	0	0	0	0	0	0	0	0	0	0	0	0	0	0	0	0	0	0
miR-510	0	0	0	0	0	0	0	0	0	0	0	0	0	0	0	0	0	0	0
miR-511	0	0	0	0	0	0	0	0	0	0	0	0	0	0	0	0	0	0	0
miR-512	0	0	0	0	0	0	0	0	0	0	0	0	0	0	0	0	0	0	0
miR-513	0	0	0	0	0	0	0	0	0	0	0	0	0	0	0	0	0	0	0
miR-513	0	0	0	0	0	0	0	0	0	0	0	0	0	0	0	0	0	0	0
miR-514	0	0	0	0	0	0	0	0	0	0	0	0	0	0	0	0	0	0	0
miR-515	0	0	0	0	0	0	0	0	0	0	0	0	0	0	0	0	0	0	0
miR-515	0	0	0	0	0	0	0	0	0	0	0	0	0	0	0	0	0	0	0
miR-516a	0	0	0	0	0	0	0	0	0	0	0	0	0	0	0	0	0	0	0
miR-516b	0	0	0	0	0	0	0	0	0	0	0	0	0	0	0	0	0	0	0
miR-517a	0	0	0	0	0	0	0	0	0	0	0	0	0	0	0	0	0	0	0
miR-517c	0	0	0	0	0	0	0	0	0	0	0	0	0	0	0	0	0	0	0
miR-518a	0	0	0	0	0	0	0	0	0	0	0	0	0	0	0	0	0	0	0
miR-518a	0	0	0	0	0	0	0	0	0	0	0	0	0	0	0	0	0	0	0
miR-518b	0	0	0	0	0	0	0	0	0	0	0	0	0	0	0	0	0	0	0
miR-518c	0	0	0	0	0	0	0	0	0	0	0	0	0	0	0	0	0	0	0
miR-518d	0	0	0	0	0	0	0	0	0	0	0	0	0	0	0	0	0	0	0
miR-518e	0	0	0	0	0	0	0	0	0	0	0	0	0	0	0	0	0	0	0
miR-518f	0	0	0	0	0	0	0	0	0	0	0	0	0	0	0	0	0	0	0
miR-519a	0	0	0	0	0	0	0	0	0	0	0	0	0	0	0	0	0	0	0
miR-519b	0	0	0	0	0	0	0	0	0	0	0	0	0	0	0	0	0	0	0
miR-519b	0	0	0	0	0	0	0	0	0	0	0	0	0	0	0	0	0	0	0
miR-519c	0	0	0	0	0	0	0	0	0	0	0	0	0	0	0	0	0	0	0
miR-519d	0	0	0	0	0	0	0	0	0	0	0	0	0	0	0	0	0	0	0
miR-519e	0	0	0	0	0	0	0	0	0	0	0	0	0	0	0	0	0	0	0
miR-520a	0	0	0	0	0	0	0	0	0	0	0	0	0	0	0	0	0	0	0
miR-520c	0	0	0	0	0	0	0	0	0	0	0	0	0	0	0	0	0	0	0
miR-520c	0	0	0	0	0	0	0	0	0	0	0	0	0	0	0	0	0	0	0
miR-520c	0	0	0	0	0	0	0	0	0	0	0	0	0	0	0	0	0	0	0

miR-520d	0	0	0	0	0	0	0	0	0	0	0	0	0	0	0	0	0	0	0
miR-520d	0	0	0	0	0	0	0	0	0	0	0	0	0	0	0	0	0	0	0
miR-520e	0	0	0	0	0	0	0	0	0	0	0	0	0	0	0	0	0	0	0
miR-520f	0	0	0	0	0	0	0	0	0	0	0	0	0	0	0	0	0	0	0
miR-520g	0	0	0	0	0	0	0	0	0	0	0	0	0	0	0	0	0	0	0
miR-520h	0	0	0	0	0	0	0	0	0	0	0	0	0	0	0	0	0	0	0
miR-521	0	0	0	0	0	0	0	0	0	0	0	0	0	0	0	0	0	0	0
miR-522	0	0	0	0	0	0	0	0	0	0	0	0	0	0	0	0	0	0	0
miR-523	0	0	0	0	0	0	0	0	0	0	0	0	0	0	0	0	0	0	0
miR-524	0	0	0	0	0	0	0	0	0	0	0	0	0	0	0	0	0	0	0
miR-525	0	0	0	0	0	0	0	0	0	0	0	0	0	0	0	0	0	0	0
miR-525	0	0	0	0	0	0	0	0	0	0	0	0	0	0	0	0	0	0	0
miR-526a	0	0	0	0	0	0	0	0	0	0	0	0	0	0	0	0	0	0	0
miR-526b	0	0	0	0	0	0	0	0	0	0	0	0	0	0	0	0	0	0	0
miR-527	0	0	0	0	0	0	0	0	0	0	0	0	0	0	0	0	0	0	0
miR-532	0	0	0	0	0	0	0	0	0	0	0	0	0	0	0	0	0	0	0
miR-532	0	0	0	0	0	0	0	0	0	0	0	0	0	0	0	0	0	0	0
miR-539	0	0	0	0	0	0	0	0	0	0	0	0	0	0	0	0	0	0	0
miR-541	0	0	0	0	0	0	0	0	0	0	0	0	0	0	0	0	0	0	0
miR-542	0	0	0	0	0	0	0	0	0	0	0	0	0	0	0	0	0	0	0
miR-542	0	0	0	0	0	0	0	0	0	0	0	0	0	0	0	0	0	0	0
miR-543	0	0	0	0	0	0	0	0	0	0	0	0	0	0	0	0	0	0	0
miR-544	0	0	0	0	0	0	0	0	0	0	0	0	0	0	0	0	0	0	0
miR-545	0	0	0	0	0	0	0	0	0	0	0	0	0	0	0	0	0	0	0
miR-548a	0	0	0	0	0	0	0	0	0	0	0	0	0	0	0	0	0	0	0
miR-548a	0	0	0	0	0	0	0	0	0	0	0	0	0	0	0	0	0	0	0
miR-548a	0	0	0	0	0	0	0	0	0	0	0	0	0	0	0	0	0	0	0
miR-548b	0	0	0	0	0	0	0	0	0	0	0	0	0	0	0	0	0	0	0
miR-548b	0	0	0	0	0	0	0	0	0	0	0	0	0	0	0	0	0	0	0
miR-548c	0	0	0	0	0	0	0	0	0	0	0	0	0	0	0	0	0	0	0
miR-548d	0	0	0	0	0	0	0	0	0	0	0	0	0	0	0	0	0	0	0
miR-548d	0	0	0	0	0	0	0	0	0	0	0	0	0	0	0	0	0	0	0
miR-549	0	0	0	0	0	0	0	0	0	0	0	0	0	0	0	0	0	0	0
miR-550	0	0	0	0	0	0	0	0	0	0	0	0	0	0	0	0	0	0	0
miR-551a	0	0	0	0	0	0	0	0	0	0	0	0	0	0	0	0	0	0	0
miR-551b	0	0	0	0	0	0	0	0	0	0	0	0	0	0	0	0	0	0	0

miR-552	0	0	0	0	0	0	0	0	0	0	0	0	0	0	0	0	0	0	0
miR-553	0	0	0	0	0	0	0	0	0	0	0	0	0	0	0	0	0	0	0
miR-554	0	0	0	0	0	0	0	0	0	0	0	0	0	0	0	0	0	0	0
miR-555	0	0	0	0	0	0	0	0	0	0	0	0	0	0	0	0	0	0	0
miR-556	0	0	0	0	0	0	0	0	0	0	0	0	0	0	0	0	0	0	0
miR-556	0	0	0	0	0	0	0	0	0	0	0	0	0	0	0	0	0	0	0
miR-557	0	0	0	0	0	0	0	0	0	0	0	0	0	0	0	0	0	0	0
miR-558	0	0	0	0	0	0	0	0	0	0	0	0	0	0	0	0	0	0	0
miR-559	0	0	0	0	0	0	0	0	0	0	0	0	0	0	0	0	0	0	0
miR-561	0	0	0	0	0	0	0	0	0	0	0	0	0	0	0	0	0	0	0
miR-562	0	0	0	0	0	0	0	0	0	0	0	0	0	0	0	0	0	0	0
miR-563	0	0	0	0	0	0	0	0	0	0	0	0	0	0	0	0	0	0	0
miR-564	0	0	0	0	0	0	0	0	0	0	0	0	0	0	0	0	0	0	0
miR-566	0	0	0	0	0	0	0	0	0	0	0	0	0	0	0	0	0	0	0
miR-567	0	0	0	0	0	0	0	0	0	0	0	0	0	0	0	0	0	0	0
miR-568	0	0	0	0	0	0	0	0	0	0	0	0	0	0	0	0	0	0	0
miR-569	0	0	0	0	0	0	0	0	0	0	0	0	0	0	0	0	0	0	0
miR-570	0	0	0	0	0	0	0	0	0	0	0	0	0	0	0	0	0	0	0
miR-571	0	0	0	0	0	0	0	0	0	0	0	0	0	0	0	0	0	0	0
miR-572	0	0	0	0	0	0	0	0	0	0	0	0	0	0	0	0	0	0	0
miR-573	0	0	0	0	0	0	0	0	0	0	0	0	0	0	0	0	0	0	0
miR-574	0	0	0	0	0	0	0	0	0	0	0	0	0	0	0	0	0	0	0
miR-574	0	0	0	0	0	0	0	0	0	0	0	0	0	0	0	0	0	0	0
miR-575	0	0	0	0	0	0	0	0	0	0	0	0	0	0	0	0	0	0	0
miR-576	0	0	0	0	0	0	0	0	0	0	0	0	0	0	0	0	0	0	0
miR-576	0	0	0	0	0	0	0	0	0	0	0	0	0	0	0	0	0	0	0
miR-577	0	0	0	0	0	0	0	0	0	0	0	0	0	0	0	0	0	0	0
miR-578	0	0	0	0	0	0	0	0	0	0	0	0	0	0	0	0	0	0	0
miR-579	0	0	0	0	0	0	0	0	0	0	0	0	0	0	0	0	0	0	0
miR-580	0	0	0	0	0	0	0	0	0	0	0	0	0	0	0	0	0	0	0
miR-581	0	0	0	0	0	0	0	0	0	0	0	0	0	0	0	0	0	0	0
miR-582	0	0	0	0	0	0	0	0	0	0	0	0	0	0	0	0	0	0	0
miR-582	0	0	0	0	0	0	0	0	0	0	0	0	0	0	0	0	0	0	0
miR-583	0	0	0	0	0	0	0	0	0	0	0	0	0	0	0	0	0	0	0
miR-584	0	0	0	0	0	0	0	0	0	0	0	0	0	0	0	0	0	0	0
miR-585	0	0	0	0	0	0	0	0	0	0	0	0	0	0	0	0	0	0	0

miR-586	0	0	0	0	0	0	0	0	0	0	0	0	0	0	0	0	0	0	0
miR-587	0	0	0	0	0	0	0	0	0	0	0	0	0	0	0	0	0	0	0
miR-588	0	0	0	0	0	0	0	0	0	0	0	0	0	0	0	0	0	0	0
miR-589	0	0	0	0	0	0	0	0	0	0	0	0	0	0	0	0	0	0	0
miR-590	0	0	0	0	0	0	0	0	0	0	0	0	0	0	0.01	0	0	0	0
miR-590	0	0	0	0	0	0	0	0	0	0	0	0	0	0	0	0	0	0	0
miR-591	0	0	0	0	0	0	0	0	0	0	0	0	0	0	0	0	0	0	0
miR-592	0	0	0	0	0	0	0	0	0	0	0	0	0	0	0	0	0	0	0
miR-593	0	0	0	0	0	0	0	0	0	0	0	0	0	0	0	0	0	0	0
miR-595	0	0	0	0	0	0	0	0	0	0	0	0	0	0	0	0	0	0	0
miR-596	0	0	0	0	0	0	0	0	0	0	0	0	0	0	0	0	0	0	0
miR-597	0	0	0	0	0	0	0	0	0	0	0	0	0	0	0	0	0	0	0
miR-598	0	0	0	0	0	0	0	0	0	0	0	0	0	0	0	0	0	0	0
miR-600	0	0	0	0	0	0	0	0	0	0	0	0	0	0	0	0	0	0	0
miR-601	0	0	0	0	0	0	0	0	0	0	0	0	0	0	0	0	0	0	0
miR-602	0	0	0	0	0	0	0	0	0	0	0	0	0	0	0	0	0	0	0
miR-603	0	0	0	0	0	0	0	0	0	0	0	0	0	0	0	0	0	0	0
miR-604	0	0	0	0	0	0	0	0	0	0	0	0	0	0	0	0	0	0	0
miR-605	0	0	0	0	0	0	0	0	0	0	0	0	0	0	0	0	0	0	0
miR-606	0	0	0	0	0	0	0	0	0	0	0	0	0	0	0	0	0	0	0
miR-607	0	0	0	0	0	0	0	0	0	0	0	0	0	0	0	0	0	0	0
miR-608	0	0	0	0	0	0	0	0	0	0	0	0	0	0	0	0	0	0	0
miR-609	0	0	0	0	0	0	0	0	0	0	0	0	0	0	0	0	0	0	0
miR-610	0	0	0	0	0	0	0	0	0	0	0	0	0	0	0	0	0	0	0
miR-611	0	0	0	0	0	0	0	0	0	0	0	0	0	0	0	0	0	0	0
miR-612	0	0	0	0	0	0	0	0	0	0	0	0	0	0	0	0	0	0	0
miR-613	0	0	0	0	0	0	0	0	0	0	0	0	0	0	0	0	0	0	0
miR-614	0	0	0	0	0	0	0	0	0	0	0	0	0	0	0	0	0	0	0
miR-615	0	0	0	0	0	0	0	0	0	0	0	0	0	0	0	0	0	0	0
miR-615	0	0	0	0	0	0	0	0	0	0	0	0	0	0	0	0	0	0	0
miR-616	0	0	0	0	0	0	0	0	0	0	0	0	0	0	0	0	0	0	0
miR-617	0	0	0	0	0	0	0	0	0	0	0	0	0	0	0	0	0	0	0
miR-618	0	0	0	0	0	0	0	0	0	0	0	0	0	0	0	0	0	0	0
miR-619	0	0	0	0	0	0	0	0	0	0	0	0	0	0	0	0	0	0	0
miR-620	0	0	0	0	0	0	0	0	0	0	0	0	0	0	0	0	0	0	0
miR-621	0	0	0	0	0	0	0	0	0	0	0	0	0	0	0	0	0	0	0

miR-622	0	0	0	0	0	0	0	0	0	0	0	0	0	0	0	0	0	0	0
miR-623	0	0	0	0	0	0	0	0	0	0	0	0	0	0	0	0	0	0	0
miR-624	0	0	0	0	0	0	0	0	0	0	0	0	0	0	0	0	0	0	0
miR-625	0	0	0	0	0	0	0	0	0	0	0	0	0	0	0	0	0	0	0
miR-626	0	0	0	0	0	0	0	0	0	0	0	0	0	0	0	0	0	0	0
miR-627	0	0	0	0	0	0	0	0	0	0	0	0	0	0	0	0	0	0	0
miR-628	0	0	0	0	0	0	0	0	0	0	0	0	0	0	0	0	0	0	0
miR-628	0	0	0	0	0	0	0	0	0	0	0	0	0	0	0	0	0	0	0
miR-629	0	0	0	0	0	0	0	0	0	0	0	0	0	0	0	0	0	0	0
miR-630	0	0	0	0	0	0	0	0	0	0	0	0	0	0	0	0	0	0	0
miR-631	0	0	0	0	0	0	0	0	0	0	0	0	0	0	0	0	0	0	0
miR-632	0	0	0	0	0	0	0	0	0	0	0	0	0	0	0	0	0	0	0
miR-633	0	0	0	0	0	0	0	0	0	0	0	0	0	0	0	0	0	0	0
miR-634	0	0	0	0	0	0	0	0	0	0	0	0	0	0	0	0	0	0	0
miR-635	0	0	0	0	0	0	0	0	0	0	0	0	0	0	0	0	0	0	0
miR-636	0	0	0	0	0	0	0	0	0	0	0	0	0	0	0	0	0	0	0
miR-637	0	0	0	0	0	0	0	0	0	0	0	0	0	0	0	0	0	0	0
miR-638	0	0	0	0	0	0	0	0	0	0	0	0	0	0	0	0	0	0	0
miR-639	0	0	0	0	0	0	0	0	0	0	0	0	0	0	0	0	0	0	0
miR-640	0	0	0	0	0	0	0	0	0	0	0	0	0	0	0	0	0	0	0
miR-641	0	0	0	0	0	0	0	0	0	0	0	0	0	0	0	0	0	0	0
miR-642	0	0	0	0	0	0	0	0	0	0	0	0	0	0	0	0	0	0	0
miR-643	0	0	0	0	0	0	0	0	0	0	0	0	0	0	0	0	0	0	0
miR-644	0	0	0	0	0	0	0	0	0	0	0	0	0	0	0	0	0	0	0
miR-645	0	0	0	0	0	0	0	0	0	0	0	0	0	0	0	0	0	0	0
miR-646	0	0	0	0	0	0	0	0	0	0	0	0	0	0	0	0	0	0	0
miR-647	0	0	0	0	0	0	0	0	0	0	0	0	0	0	0	0	0	0	0
miR-648	0	0	0	0	0	0	0	0	0	0	0	0	0	0	0	0	0	0	0
miR-649	0	0	0	0	0	0	0	0	0	0	0	0	0	0	0	0	0	0	0
miR-650	0	0	0	0	0	0	0	0	0	0	0	0	0	0	0	0	0	0	0
miR-651	0	0	0	0	0	0	0	0	0	0	0	0	0	0	0	0	0	0	0
miR-652	0	0	0	0	0	0	0	0	0	0	0	0	0	0	0	0	0	0	0
miR-653	0	0	0	0	0	0	0	0	0	0	0	0	0	0	0	0	0	0	0
miR-654	0	0	0	0	0	0	0	0	0	0	0	0	0	0	0	0	0	0	0
miR-654	0	0	0	0	0	0	0	0	0	0	0	0	0	0	0	0	0	0	0
miR-655	0	0	0	0	0	0	0	0	0	0	0	0	0	0	0	0	0	0	0

miR-656	0	0	0	0	0	0	0	0	0	0	0	0	0	0	0	0	0	0	0
miR-657	0	0	0	0	0	0	0	0	0	0	0	0	0	0	0	0	0	0	0
miR-658	0	0	0	0	0	0	0	0	0	0	0	0	0	0	0	0	0	0	0
miR-659	0	0	0	0	0	0	0	0	0	0	0	0	0	0	0	0	0	0	0
miR-660	0	0	0	0	0	0	0	0	0	0	0	0	0	0	0	0	0	0	0
miR-661	0	0	0	0	0	0	0	0	0	0	0	0	0	0	0	0	0	0	0
miR-662	0	0	0	0	0	0	0	0	0	0	0	0	0	0	0	0	0	0	0
miR-665	0	0	0	0	0	0	0	0	0	0	0	0	0	0	0	0	0	0	0
miR-671	0	0	0	0	0	0	0	0	0	0	0	0	0	0	0	0	0	0	0
miR-671	0	0	0	0	0	0	0	0	0	0	0	0	0	0	0	0	0	0	0
miR-672	0	0	0	0	0	0	0	0	0	0	0	0	0	0	0	0	0	0	0
miR-674	0	0	0	0	0	0	0	0	0	0	0	0	0	0	0	0	0	0	0
miR-674	0	0	0	0	0	0	0	0	0	0	0	0	0	0	0	0	0	0	0
miR-7	0	0	0	0	0	0	0	0	0	0	0	0	0	0	0	0	0	0	0
miR-708	0	0	0	0	0	0	0	0	0	0	0	0	0	0.01	0	0	0	0	0
miR-744	0	0	0	0	0	0	0	0	0	0	0	0	0	0	0.01	0	0	0	0
miR-758	0	0	0	0	0	0	0	0	0	0	0	0	0	0	0	0	0	0	0
miR-760	0	0	0	0	0	0	0	0	0	0	0	0	0	0	0	0	0	0	0
miR-770	0	0	0	0	0	0	0	0	0	0	0	0	0	0	0	0	0	0	0
miR-770	0	0	0	0	0	0	0	0	0	0	0	0	0	0	0	0	0	0	0
miR-802	0	0	0	0	0	0	0	0	0	0	0	0	0	0	0	0	0	0	0
miR-871	0	0	0	0	0	0	0	0	0	0	0	0	0	0	0	0	0	0	0
miR-872	0	0	0	0	0	0	0	0	0	0	0	0	0	0	0	0	0	0	0
miR-873	0	0	0	0	0	0	0	0	0	0	0	0	0	0	0	0	0	0	0
miR-874	0	0	0	0	0	0	0	0	0	0	0	0	0	0	0	0	0	0	0
miR-875	0	0	0	0	0	0	0	0	0	0	0	0	0	0	0	0	0	0	0
miR-875	0	0	0	0	0	0	0	0	0	0	0	0	0	0	0	0	0	0	0
miR-876	0	0	0	0	0	0	0	0	0	0	0	0	0	0	0	0	0	0	0
miR-876	0	0	0	0	0	0	0	0	0	0	0	0	0	0	0	0	0	0	0
miR-877	0	0	0	0	0	0	0	0	0	0	0	0	0	0	0	0	0	0	0
miR-885	0	0	0	0	0	0	0	0	0	0	0	0	0	0	0	0	0	0	0
miR-885	0	0	0	0	0	0	0	0	0	0	0	0	0	0	0	0	0	0	0
miR-887	0	0	0	0	0	0	0	0	0	0	0	0	0	0	0	0	0	0	0
miR-888	0	0	0	0	0	0	0	0	0	0	0	0	0	0	0	0	0	0	0
miR-889	0	0	0	0	0	0	0	0	0	0	0	0	0	0	0	0	0	0	0
miR-890	0	0	0	0	0	0	0	0	0	0	0	0	0	0	0	0	0	0	0

miR-891a	0	0	0	0	0	0	0	0	0	0	0	0	0	0	0	0	0	0	0
miR-891b	0	0	0	0	0	0	0	0	0	0	0	0	0	0	0	0	0	0	0
miR-892a	0	0	0	0	0	0	0	0	0	0	0	0	0	0	0	0	0	0	0
miR-892b	0	0	0	0	0	0	0	0	0	0	0	0	0	0	0	0	0	0	0
miR-9	0	0.01	0	0	0.03	0	0	0	0	0	0	0.09	0.17	0.17	0.01	0	0	0	0
miR-92a	0.02	0.02	0.01	0.01	0.02	0	0.01	0	0	0	0.01	0	0	0	0.03	0.01	0.01	0.01	0
miR-92b	0	0	0	0	0	0	0	0	0	0	0	0.01	0	0	0	0	0	0	0
miR-93	0.01	0.02	0.01	0	0	0.01	0	0.01	0	0	0.01	0	0	0	0.01	0.01	0	0	0
miR-95	0	0	0	0	0	0	0	0	0	0	0	0	0	0	0	0	0	0	0
miR-96	0	0	0	0	0	0	0	0	0	0	0	0	0	0	0	0	0	0	0
miR-98	0	0	0	0	0	0	0	0	0	0	0	0	0	0	0.01	0	0	0	0
miR-99a	0	0	0	0	0	0.01	0	0	0	0	0	0.03	0.02	0.02	0.01	0.01	0	0	0
miR-99b	0	0	0	0	0	0.15	0	0.06	0	0	0	0.02	0.01	0.02	0	0	0	0	0
	0.97																		

miR_B-	miR_B-	miR_B-	miR_B-	miR_B-	miR_B-	miR_B-	miR_B-	miR_B-	miR_B-	miR_B-	miR_B-	miR_B-	miR_B-	miR_B-	miR_B-	miR_B-	miR_B-	miR_B-	miR_B-	miR_B-
0.01	0.05	0.04	0.02	0.02	0.03	0.01	0.05	0	0.05	0.09	0.05	0.04	0.03	0.04	0.02	0.02	0.01	0.01	0.01	0.02
0.01	0.02	0.01	0	0.02	0	0	0.01	0.01	0.02	0.02	0.02	0.01	0.01	0.02	0	0	0	0	0	0
0	0	0.01	0	0	0	0	0	0	0	0	0	0	0	0	0	0	0	0	0	0
0	0.01	0	0.01	0	0	0	0.01	0	0.01	0.02	0.01	0	0	0	0	0	0	0	0	0
0	0	0.01	0	0	0	0	0	0	0	0	0	0	0	0	0	0	0	0	0	0
0.01	0.02	0.01	0.01	0	0.02	0.01	0.02	0	0.05	0.03	0.01	0.01	0.01	0.03	0.02	0.04	0.08	0.02	0.01	0.02
0	0.01	0	0	0	0	0.01	0.01	0	0.01	0.01	0.01	0.01	0.01	0.02	0.01	0	0.01	0.01	0	0.01
0	0	0	0	0	0.01	0	0.01	0	0.01	0	0	0	0.01	0.01	0	0	0.01	0	0	0
0	0	0	0	0	0	0	0	0	0	0	0	0	0	0	0	0	0	0	0	0
0	0	0	0	0	0	0	0	0	0	0	0	0	0	0	0	0	0	0	0	0
0	0	0	0	0	0	0	0	0	0	0	0	0	0	0	0	0	0	0	0	0
0	0	0	0	0	0	0	0	0	0	0	0	0	0	0	0	0	0	0	0	0
0	0	0	0	0	0	0	0	0	0	0	0.01	0	0.02	0	0	0	0	0.01	0.01	0.02
0	0	0	0	0	0	0	0	0	0	0	0	0	0	0	0	0	0	0	0	0
0	0	0	0	0	0	0	0	0	0	0	0	0	0	0	0	0	0	0	0	0
0	0	0	0	0	0	0	0	0	0	0	0	0	0	0	0	0	0	0	0	0
0.01	0	0	0.01	0	0	0	0.01	0	0.02	0	0	0	0	0	0	0	0.01	0	0	0
0	0	0	0	0	0	0	0	0	0	0	0	0	0	0	0	0	0	0	0	0
0	0	0	0	0	0	0	0	0	0	0	0	0	0	0	0	0	0	0	0	0
0	0	0	0	0	0	0	0	0	0	0	0	0	0	0	0	0	0	0	0	0
0	0	0	0	0	0	0	0	0	0	0	0	0	0	0	0	0	0	0	0	0
0	0	0	0	0	0	0	0	0	0	0	0	0	0	0	0	0	0	0	0	0
0	0	0	0	0	0	0	0	0	0	0	0	0	0	0	0	0	0	0	0	0
0	0	0.01	0	0	0	0	0	0	0	0	0	0	0	0	0	0	0	0	0	0
0	0	0	0	0.01	0	0	0	0	0	0	0	0	0	0	0	0	0	0	0	0
0.02	0.08	0.03	0.09	0.05	0.01	0.05	0.01	0.04	0.03	0	0.05	0.09	0	0.05	0	0	0	0	0	0
0	0	0	0	0	0	0	0	0	0	0	0	0	0	0	0	0	0	0	0	0
0	0	0	0	0	0	0	0	0	0	0	0	0	0	0	0	0	0	0	0	0
0	0.01	0	0	0	0.01	0	0.02	0	0	0.01	0	0	0	0	0	0	0	0	0	0
0	0.01	0	0	0	0.01	0	0.02	0	0	0	0	0	0	0	0	0	0	0	0	0
0	0	0	0	0	0	0	0	0	0	0	0	0	0	0	0	0	0	0	0	0
0	0	0	0	0	0	0	0	0	0	0	0	0	0	0	0	0	0	0	0	0
0	0	0	0	0	0	0	0	0	0	0	0	0	0	0	0	0	0	0	0	0
0.01	0	0.01	0.02	0.01	0	0	0	0	0	0.01	0.01	0	0	0	0	0	0	0	0	0
0	0	0	0	0	0	0	0	0	0	0	0	0	0	0	0	0	0	0	0	0

0	0	0	0	0	0	0	0	0	0	0	0	0	0	0	0	0	0	0	0	0
0	0	0	0	0	0	0	0	0	0	0	0	0	0	0	0	0	0	0	0	0
0	0	0	0	0	0	0	0	0	0	0	0	0	0	0	0	0	0	0	0	0
0	0	0	0	0	0	0	0	0	0	0	0	0	0	0	0	0	0	0	0	0
0	0	0	0	0	0	0	0	0	0	0	0	0	0	0	0	0	0	0	0	0
0	0	0	0	0	0	0	0	0	0	0	0	0	0	0	0	0	0	0	0	0
0	0	0	0	0	0	0	0	0	0	0	0	0	0	0	0	0	0	0	0	0
0	0	0	0	0	0	0	0	0	0	0	0	0	0	0	0	0	0	0	0	0
0	0	0	0	0	0	0	0	0	0	0	0	0	0	0	0	0	0	0	0	0
0	0	0	0	0	0	0	0	0	0	0	0	0	0	0	0	0	0	0	0	0
0	0	0	0	0	0	0	0	0	0	0	0	0	0	0	0	0	0	0	0	0
0	0	0	0	0.01	0	0.01	0	0	0	0	0	0	0.01	0	0	0	0	0	0	0
0.01	0.01	0.01	0.01	0.02	0.01	0.01	0	0.02	0	0	0.01	0.01	0.01	0.01	0.01	0.01	0.01	0.01	0.01	0.01
0	0	0	0	0	0	0	0	0	0	0.01	0	0	0	0	0	0	0	0	0	0
0.08	0.1	0.01	0.08	0.04	0.13	0.08	0.13	0.09	0.12	0.07	0.07	0.1	0.1	0.13	0.16	0.24	0.18	0.22	0.19	0.26
0.2	0.17	0.06	0.36	0.09	0.27	0.17	0.18	0.17	0.09	0.08	0.21	0.2	0.39	0.13	0.35	0.13	0.07	0.13	0.15	0.14
0	0	0	0	0	0	0.01	0	0.01	0.01	0	0	0	0	0	0	0	0	0	0	0
0.13	0.01	0.07	0.01	0.16	0.01	0.07	0.01	0.12	0.06	0.01	0.15	0	0	0.01	0	0	0	0	0	0
0	0	0	0	0	0	0	0	0	0	0	0	0	0	0	0	0	0	0	0	0
0	0.02	0	0.01	0.01	0.02	0	0.02	0	0	0.01	0	0.01	0	0	0	0.03	0.04	0	0	0
0	0	0	0	0	0	0	0	0	0	0	0	0	0	0	0	0	0	0	0	0
0	0	0	0	0	0	0	0	0	0	0	0	0	0	0	0	0	0	0	0	0
0	0	0	0	0	0	0	0	0	0	0	0	0	0	0	0	0	0	0	0	0
0	0	0	0	0	0	0	0	0	0	0	0	0	0	0	0	0	0	0	0	0
0	0	0	0	0	0	0	0	0	0	0	0	0	0	0	0	0	0	0	0	0
0	0	0	0	0	0	0	0	0	0	0	0	0	0	0	0	0	0	0	0	0
0	0	0	0	0	0	0	0	0	0	0	0	0	0	0	0	0	0	0	0	0
0.01	0.01	0	0	0	0	0.02	0	0	0.02	0	0.02	0	0.05	0.02	0.02	0	0	0.05	0.08	0.08
0	0	0	0	0	0	0	0	0	0	0	0	0	0	0	0	0	0	0	0	0
0	0.01	0.02	0.01	0.01	0	0	0	0	0	0	0	0	0	0	0	0	0	0	0	0
0	0	0	0	0	0	0	0	0	0	0	0	0	0	0	0	0	0	0	0	0
0	0	0	0	0	0	0	0	0	0	0	0	0	0	0	0	0	0	0	0	0
0	0	0	0	0	0	0	0	0	0	0	0	0	0	0	0	0	0	0	0	0
0	0.01	0	0	0.01	0	0	0	0	0	0.05	0	0	0	0.01	0	0.22	0.11	0.01	0.01	0.01
0.06	0.03	0.04	0.03	0.02	0.04	0.07	0.04	0.06	0.07	0.04	0.06	0.03	0.02	0.03	0.03	0.02	0.02	0.07	0.03	0.01
0	0	0.01	0	0	0	0	0	0	0.01	0	0	0	0	0	0	0	0	0	0	0

0.19	0.09	0.03	0.04	0.11	0.11	0.18	0.11	0.22	0.17	0.1	0.1	0.1	0.06	0.11	0.1	0.04	0.05	0.09	0.1	0.05
0.01	0	0.02	0.01	0	0.01	0.01	0.01	0.01	0	0.02	0.01	0.01	0	0	0	0	0	0	0	0
0.01	0.07	0	0.03	0	0.06	0	0.05	0	0	0.02	0.02	0.09	0.01	0.02	0	0	0.01	0	0	0
0	0.01	0	0	0	0.01	0	0.02	0	0	0	0	0.01	0	0	0	0	0.01	0	0	0
0	0	0	0	0	0	0	0	0	0	0	0	0	0	0	0	0	0	0	0	0
0	0	0.01	0	0	0	0	0	0	0	0	0	0	0	0	0	0	0	0	0	0
0	0	0	0	0	0	0	0	0	0	0	0	0	0	0	0	0	0	0	0	0
0	0	0	0	0	0	0	0	0	0	0	0	0	0	0	0	0	0	0	0	0
0	0	0	0	0	0	0	0	0	0	0	0	0	0	0	0	0	0	0	0	0
0	0	0	0	0.01	0	0	0	0	0	0.01	0	0	0	0	0	0	0	0	0	0
0	0	0	0	0.01	0	0	0	0	0	0	0	0	0	0.01	0	0	0	0	0	0
0	0	0	0	0	0	0	0	0	0	0	0	0	0	0	0	0	0	0	0	0
0	0	0	0	0	0	0	0	0	0	0	0	0	0	0	0	0	0	0	0	0
0	0	0	0	0	0	0	0	0	0	0	0	0	0	0	0	0	0	0	0	0
0	0	0.01	0	0.01	0	0	0	0	0	0.03	0	0	0	0.01	0	0	0	0	0	0
0	0	0	0	0	0	0	0	0	0	0	0	0	0	0	0	0	0	0	0	0
0	0	0	0	0	0	0	0	0	0	0	0	0	0	0	0	0	0	0	0	0
0	0	0	0	0	0	0	0	0	0	0	0	0	0	0	0	0	0	0	0	0
0.01	0.01	0	0.02	0	0.01	0	0.02	0	0.03	0.04	0.01	0.01	0	0.01	0.01	0	0.01	0	0	0
0	0	0.01	0	0	0	0	0	0	0	0.01	0	0	0	0	0	0	0	0	0	0
0	0	0	0	0	0	0	0	0	0	0	0	0	0	0	0	0	0	0	0	0
0	0	0	0	0	0	0	0	0	0	0	0	0	0	0	0	0	0	0	0	0
0	0	0	0	0	0	0	0	0	0	0	0	0	0	0	0	0	0	0	0	0
0	0	0.03	0	0.02	0	0	0	0	0	0.01	0	0	0	0	0	0	0	0	0	0
0	0	0	0	0	0	0	0	0	0	0	0	0	0	0	0	0	0	0	0	0
0	0	0	0	0	0	0	0	0	0	0	0	0	0	0	0	0	0	0	0	0
0	0	0	0	0	0	0	0	0	0	0	0	0	0	0	0	0	0	0	0	0
0	0	0	0	0	0	0	0	0	0	0	0	0	0	0	0	0	0	0	0	0
0	0	0	0	0	0	0	0	0	0	0	0	0	0	0	0	0	0	0	0	0
0	0	0	0	0	0	0	0	0	0	0	0	0	0	0	0	0	0	0	0	0
0	0	0	0	0	0	0	0	0	0	0	0	0	0	0	0	0	0	0	0	0
0	0	0	0	0	0	0	0	0	0	0	0	0	0	0	0	0	0	0	0	0
0	0	0	0	0	0	0	0	0	0	0	0	0	0	0	0	0	0	0	0	0
0	0.01	0	0	0	0	0	0	0	0	0.02	0	0	0	0.01	0	0.01	0.02	0	0	0
0	0.01	0.01	0.01	0.01	0	0.01	0	0	0.02	0.01	0	0	0	0.01	0.01	0.01	0.04	0	0	0
0	0	0	0	0	0	0	0	0	0	0	0	0	0	0	0	0	0	0	0	0

0	0	0	0	0	0	0	0	0	0	0	0	0	0	0	0	0	0	0	0	0
0	0	0	0	0	0	0	0	0	0	0	0	0	0	0	0	0	0	0	0	0
0	0	0	0	0	0	0	0	0	0	0	0	0	0	0	0	0	0	0	0	0
0	0	0	0	0	0	0	0	0	0	0	0	0	0	0	0	0	0	0	0	0
0	0	0	0	0	0	0	0	0	0	0	0	0	0	0	0	0	0	0	0	0
0	0	0	0	0	0	0	0	0	0	0	0	0	0	0	0	0	0	0	0	0
0	0	0	0	0	0	0	0	0	0	0	0	0	0	0	0	0	0	0	0	0
0	0	0	0	0	0	0	0	0	0	0	0	0	0	0	0	0	0	0	0	0
0	0	0	0	0	0	0	0	0	0	0	0	0	0	0	0	0	0	0	0	0
0	0.01	0.02	0	0.01	0.01	0	0	0	0	0.01	0	0	0	0	0	0	0.01	0	0	0
0	0	0	0	0	0.01	0	0	0	0	0	0	0	0	0	0	0	0	0	0	0
0.01	0.01	0.14	0.02	0.09	0.01	0.02	0.01	0.01	0.03	0.07	0.01	0	0.02	0.02	0.01	0.05	0.08	0.07	0.04	0.04
0	0	0	0	0	0	0	0	0	0	0	0	0	0	0	0	0	0	0	0	0
0	0	0	0	0	0	0	0	0	0	0	0	0	0	0	0	0	0	0	0	0
0	0	0	0	0	0	0	0	0	0	0	0	0	0	0	0	0	0	0	0	0
0	0	0	0	0	0	0	0	0	0	0	0	0	0	0	0	0	0	0	0	0
0	0	0	0	0	0	0	0	0	0	0	0	0	0	0	0	0	0	0	0	0
0	0	0	0	0	0	0	0	0	0	0	0	0	0	0	0	0	0	0	0	0
0	0	0	0	0	0	0	0	0	0	0	0	0	0	0	0	0	0	0	0	0
0	0	0	0	0	0	0	0	0	0	0	0	0	0	0	0	0	0	0	0	0
0	0	0	0	0	0	0	0	0	0	0	0	0	0	0	0	0	0	0	0	0
0	0	0	0	0	0	0	0	0	0	0	0	0	0	0	0	0	0	0	0	0
0	0	0	0	0	0	0	0	0	0	0	0	0	0	0	0	0	0	0	0	0
0	0	0.02	0	0.01	0	0.01	0	0.01	0	0	0.01	0	0	0	0	0	0	0	0	0
0	0	0	0	0	0	0	0	0	0	0	0	0	0	0	0	0	0	0	0	0
0	0	0	0	0	0	0	0	0	0	0	0	0	0	0	0	0	0	0	0	0
0	0	0	0	0	0	0	0	0	0	0	0	0	0	0	0	0	0	0	0	0
0	0	0	0	0	0	0	0	0	0	0	0	0	0	0	0	0	0	0	0	0
0	0.01	0	0	0	0	0	0.01	0	0	0	0	0.02	0	0	0	0	0.01	0	0	0
0.01	0	0	0	0.01	0	0.01	0	0.01	0.01	0	0	0	0	0	0	0	0	0	0	0
0	0	0	0	0	0	0	0	0	0	0	0	0	0	0	0	0	0	0	0	0
0	0	0.01	0	0	0	0	0	0	0	0	0	0	0	0	0	0	0.01	0	0	0
0	0	0.01	0	0	0	0	0	0	0	0	0	0	0	0	0	0	0	0	0	0
0	0.01	0.01	0	0	0.01	0	0	0.01	0	0	0	0	0	0	0	0.01	0	0	0	0
0	0	0.02	0.01	0.01	0	0.01	0	0	0	0.01	0	0	0	0	0	0	0.01	0	0	0
0	0.02	0.02	0	0.01	0.01	0.02	0.01	0	0.02	0.01	0.01	0.03	0.01	0.02	0.03	0.01	0.01	0.03	0.06	0.06

0.01	0.01	0	0	0	0	0	0	0	0.01	0.01	0	0.01	0.01	0.01	0.02	0	0.01	0.01	0.03	0.02
0.02	0.01	0.01	0	0.01	0.01	0.03	0	0.03	0.01	0.02	0.01	0.01	0	0.01	0	0.01	0.01	0	0	0
0	0	0	0	0.01	0	0	0	0	0	0	0	0	0	0	0	0	0	0	0	0
0	0	0	0	0.01	0	0	0	0	0	0.01	0	0	0	0	0	0	0	0	0	0
0	0.01	0.01	0.01	0.03	0	0	0	0	0	0	0	0	0	0	0	0	0	0.01	0.01	0.01
0	0	0	0	0	0	0	0	0	0	0	0	0	0	0	0	0	0	0	0	0
0	0	0	0	0	0	0	0	0	0	0	0	0	0	0	0	0	0	0	0	0
0	0	0	0	0	0	0	0	0	0	0	0	0	0	0	0	0	0	0	0	0
0	0	0	0	0	0	0	0	0	0	0	0	0	0	0	0	0	0	0	0	0
0	0	0	0	0	0	0	0	0	0	0	0	0	0	0	0	0	0	0	0	0
0	0	0.01	0.01	0	0	0.01	0	0	0	0.02	0.01	0.01	0.03	0.02	0.02	0	0.01	0.03	0.03	0.03
0.01	0.01	0.03	0.01	0.01	0.01	0.01	0.01	0.01	0	0.04	0.02	0.01	0.1	0.07	0.08	0.02	0.02	0.11	0.09	0.09
0	0	0	0	0	0	0	0	0	0	0.01	0.01	0	0.02	0	0.01	0	0	0.01	0.02	0.01
0	0	0	0	0	0	0	0	0	0	0	0	0	0	0	0	0	0	0	0	0
0	0	0	0	0	0	0	0	0	0	0	0	0	0	0	0	0	0	0	0	0
0	0	0	0	0	0	0	0	0	0	0	0	0	0	0	0	0	0	0	0	0
0	0	0	0	0	0	0	0	0	0	0	0	0	0	0	0	0	0	0	0	0
0	0	0	0	0	0	0	0	0	0	0	0	0	0	0	0	0	0	0	0	0
0	0	0	0	0	0	0	0	0	0	0	0	0	0	0	0	0	0	0	0	0
0	0	0	0	0	0	0	0	0	0	0	0	0	0	0	0	0	0	0	0	0
0	0	0	0	0	0	0	0.02	0	0	0	0	0	0	0	0	0	0	0	0	0
0	0	0	0	0	0	0	0	0	0	0.01	0	0	0	0	0	0	0	0	0	0
0	0	0	0	0	0	0	0	0	0	0	0	0	0	0	0	0	0	0	0	0
0	0.03	0.01	0.01	0	0.01	0.01	0.02	0.01	0	0	0.01	0.02	0.01	0.02	0.01	0.02	0.01	0.01	0.01	0.02
0	0.02	0.01	0.03	0.01	0.01	0.01	0	0.01	0	0.02	0.02	0.02	0.02	0.02	0.03	0.02	0.02	0.04	0.03	0.04
0	0	0	0	0	0	0	0	0	0	0	0	0	0	0	0	0	0	0	0	0
0.01	0.01	0.03	0.01	0.01	0.02	0.01	0.01	0.01	0	0	0	0	0.01	0	0	0	0	0	0	0.01
0	0	0	0	0	0.01	0	0.01	0	0	0	0	0	0	0.01	0	0	0	0	0	0
0	0	0	0	0	0	0	0	0	0	0	0	0	0	0	0	0	0	0	0	0
0	0	0	0	0	0	0	0	0	0	0	0	0	0	0	0	0	0	0	0	0
0	0	0	0	0	0	0	0	0	0	0	0	0	0	0	0	0	0	0	0	0
0	0	0	0	0	0	0	0	0	0	0	0	0	0	0	0	0	0	0	0	0
0	0	0	0	0	0	0	0	0	0	0	0	0	0	0	0	0	0	0	0	0
0	0	0	0	0	0	0	0	0	0	0	0	0	0	0	0	0	0	0	0	0
0	0	0	0	0	0	0	0	0	0	0	0	0	0	0	0	0	0	0	0	0
0	0	0	0	0	0	0	0	0	0	0	0	0	0	0	0	0	0	0	0	0

0	0	0	0	0	0	0	0	0	0	0	0	0	0	0	0	0	0	0	0	0
0	0	0	0	0	0	0	0	0	0	0	0	0	0	0	0	0	0	0	0	0
0	0	0	0	0	0	0	0	0	0	0	0	0	0	0	0	0	0	0	0	0
0	0	0	0	0	0	0	0	0	0	0	0	0	0	0	0	0	0	0	0	0
0	0	0	0	0	0	0	0	0	0	0	0	0	0	0	0	0	0	0	0	0
0	0	0	0	0	0	0	0	0	0	0	0	0	0	0	0	0	0	0	0	0
0	0	0	0	0	0	0	0	0	0	0	0	0	0	0	0	0	0	0	0	0
0	0	0	0	0	0	0	0	0	0	0	0	0	0	0	0	0	0	0	0	0
0	0	0	0	0	0	0	0	0	0	0	0	0	0	0	0	0	0	0	0	0
0	0	0	0	0	0	0	0	0	0	0	0	0	0	0	0	0	0	0	0	0
0	0	0	0	0	0	0	0	0	0	0	0	0	0	0	0	0	0	0	0	0
0	0	0	0	0	0	0	0	0	0	0	0	0	0	0.01	0	0	0	0	0	0
0	0	0	0	0	0	0	0	0	0	0	0	0	0	0	0	0	0	0	0	0
0	0	0	0	0	0	0	0	0	0	0	0	0	0	0	0	0	0	0	0	0
0	0	0	0	0	0	0	0	0	0	0	0	0	0	0	0	0	0	0	0	0
0	0	0	0	0	0	0.01	0	0	0.01	0	0	0	0	0	0	0	0	0	0	0
0	0	0	0	0	0	0	0	0	0	0	0	0	0	0	0	0	0	0	0	0
0	0	0	0	0	0	0	0	0	0	0	0	0	0	0	0	0	0	0	0	0
0	0	0	0	0	0	0	0	0	0	0	0	0	0	0	0	0	0	0	0	0
0	0	0	0	0	0	0	0	0	0	0	0	0	0	0	0	0	0	0	0	0
0	0	0	0	0	0	0	0	0	0	0	0	0	0	0	0	0	0	0	0	0
0	0	0	0	0	0	0	0	0	0	0	0	0	0	0	0	0	0	0	0	0
0	0	0	0	0	0	0	0	0	0	0	0	0	0	0	0	0	0	0	0	0
0	0	0	0	0	0	0	0	0	0	0	0	0	0	0	0	0	0	0	0	0
0	0	0	0	0	0	0	0	0	0	0.01	0	0	0	0	0	0	0	0	0	0
0	0	0	0	0	0	0	0	0	0	0	0	0	0	0	0	0	0	0	0	0
0	0	0	0	0	0	0	0	0	0	0	0	0	0	0	0	0	0	0	0	0
0	0	0	0	0	0	0	0.01	0	0	0	0	0	0	0	0	0	0	0	0	0
0	0	0	0	0	0	0	0	0	0	0	0	0	0	0	0	0	0	0	0	0
0	0	0	0	0	0	0	0	0	0	0	0	0	0	0	0	0	0	0	0	0
0	0	0	0	0	0	0	0	0	0	0	0	0	0	0	0	0	0	0	0	0
0	0	0	0	0	0	0	0	0	0	0	0	0	0	0	0	0	0	0	0	0
0	0	0	0	0	0	0	0	0	0	0	0	0	0	0	0	0	0	0	0	0
0	0	0	0	0	0	0	0	0	0	0	0	0	0	0	0	0	0	0	0	0
0	0	0	0	0	0	0	0	0	0	0	0	0	0	0	0	0	0	0	0	0
0	0	0	0	0	0	0	0	0	0	0	0	0	0	0	0	0	0	0	0	0

0	0	0	0	0	0	0	0	0	0	0	0	0	0	0	0	0	0	0	0	0
0	0	0	0	0	0	0	0	0	0	0	0	0	0	0	0	0	0	0	0	0
0	0	0.01	0	0.01	0	0	0	0	0	0.02	0	0	0	0.01	0	0	0	0	0	0
0	0	0	0.01	0.01	0	0	0.01	0	0	0	0	0	0	0	0	0	0	0	0	0
0	0	0	0	0	0	0	0	0	0	0	0	0	0	0	0	0	0	0	0	0
0	0	0	0	0	0	0	0	0	0	0	0	0	0	0	0	0	0	0	0	0
0	0	0	0	0	0	0	0	0	0	0	0	0	0	0	0	0	0	0	0	0
0	0	0	0	0	0	0	0	0	0	0	0	0	0	0	0	0	0	0	0	0
0	0	0	0	0	0	0	0	0	0	0	0	0	0	0	0	0	0	0	0	0
0	0	0	0	0	0	0	0	0	0	0	0	0	0	0	0	0	0	0	0	0
0	0	0	0	0	0	0	0	0	0	0	0	0	0	0	0	0	0	0	0	0
0	0	0	0	0	0	0	0	0	0	0	0	0	0	0	0	0	0	0	0	0
0	0	0	0	0	0	0	0	0	0	0	0	0	0	0	0	0	0	0	0	0
0	0	0	0	0	0	0	0	0	0	0	0	0	0	0	0	0	0	0	0	0
0	0	0	0	0	0	0	0	0	0	0	0	0	0	0	0	0	0	0	0	0
0	0	0	0	0	0	0	0	0	0	0	0	0	0	0	0	0	0	0	0	0
0	0	0	0	0	0	0	0	0	0	0	0	0	0	0	0	0	0	0	0	0
0	0	0	0	0	0	0	0	0	0	0	0	0	0	0	0	0	0	0	0	0
0	0	0	0	0	0	0	0	0	0	0	0	0	0	0	0	0	0	0	0	0
0	0	0	0	0	0	0	0	0	0	0	0	0	0	0	0	0	0	0	0	0
0	0	0	0	0	0	0	0	0	0	0	0	0	0	0	0	0	0	0	0	0
0	0	0	0	0	0	0	0	0	0	0	0	0	0	0	0	0	0	0	0	0
0	0	0	0	0	0	0	0	0	0	0	0	0	0	0	0	0	0	0	0	0
0	0	0.01	0	0	0.01	0	0	0	0	0	0	0	0	0	0	0	0	0	0	0
0	0.01	0	0.01	0	0.01	0	0.01	0	0	0	0.01	0	0	0	0	0	0	0	0	0
0.01	0	0	0	0	0	0.01	0	0.01	0	0	0.01	0	0	0.01	0	0	0	0	0	0
0.01	0.01	0.02	0.01	0.01	0.01	0.01	0.01	0.01	0	0.01	0	0.01	0	0	0	0	0	0	0	0
0	0	0	0	0	0	0	0	0	0	0	0	0	0	0	0	0	0	0	0	0
0	0	0	0	0	0	0	0	0	0	0	0	0	0	0	0	0	0	0	0	0
0	0	0	0	0	0	0	0	0	0	0	0	0	0	0	0	0	0	0	0	0
0	0	0	0	0	0	0	0	0	0	0	0	0	0	0	0	0	0	0	0	0
0	0	0	0	0	0	0	0	0	0	0	0	0	0	0	0	0	0	0	0	0
0	0	0	0	0	0	0	0	0	0	0	0	0	0	0	0	0	0	0	0	0
0	0	0	0	0	0	0	0	0	0	0	0	0	0	0	0	0	0	0	0	0
0	0	0	0	0	0	0	0	0	0	0	0	0	0	0	0	0	0	0	0	0
0	0	0	0	0	0	0	0	0	0	0	0	0	0	0	0	0	0	0	0	0

0	0	0	0	0	0	0	0	0	0	0	0	0	0	0	0	0	0	0	0	0
0.04	0	0.02	0	0.03	0	0.02	0	0.03	0.01	0	0.01	0	0	0	0	0	0	0	0	0
0	0	0	0	0	0	0	0	0	0	0	0	0	0	0	0	0	0	0	0	0
0	0	0	0	0	0	0	0	0	0	0	0	0	0	0	0	0	0	0	0	0
0	0	0	0	0	0	0	0	0	0	0	0	0	0	0	0	0	0	0	0	0
0	0	0	0	0	0	0	0	0	0	0	0	0	0	0	0	0	0	0	0	0
0	0	0	0	0	0	0	0	0	0	0	0	0	0	0	0	0	0	0	0	0
0	0	0	0	0	0	0	0	0	0	0	0	0	0	0	0	0	0	0	0	0
0	0	0	0	0	0	0	0	0	0	0	0	0	0	0	0	0	0	0	0	0
0	0	0	0	0	0	0	0	0	0	0	0	0	0	0	0	0	0	0	0	0
0	0	0	0	0	0	0	0	0	0	0	0	0	0	0	0	0	0	0	0	0
0	0	0	0	0	0	0	0	0	0	0	0	0	0	0	0	0	0	0	0	0
0	0	0	0	0	0	0	0	0	0	0	0	0	0	0	0	0	0	0	0	0
0	0	0	0	0	0	0	0	0	0	0	0	0	0	0	0	0	0	0	0	0
0	0	0	0	0	0	0	0	0	0	0	0	0	0	0	0	0	0	0	0	0
0	0	0	0	0	0	0	0	0	0	0	0	0	0	0	0	0	0	0	0	0
0	0	0	0	0	0	0	0	0	0	0	0	0	0	0	0	0	0	0	0	0
0	0	0	0	0	0	0	0	0	0	0	0	0	0	0	0	0	0	0	0	0
0	0	0	0	0	0	0	0	0	0	0	0	0	0	0	0	0	0	0	0	0
0	0	0	0	0	0	0	0	0	0	0	0	0	0	0	0	0	0	0	0	0
0	0	0	0	0	0	0	0	0	0	0	0	0	0	0	0	0	0	0	0	0
0	0	0	0	0	0	0	0	0	0	0	0	0	0	0	0	0	0	0	0	0
0	0	0	0	0	0	0	0	0	0	0	0	0	0	0	0	0	0	0	0	0
0	0	0	0	0	0	0	0	0	0	0	0	0	0	0	0	0	0	0	0	0
0	0	0	0	0	0	0	0	0	0	0	0	0	0	0	0	0	0	0	0	0
0	0	0	0	0	0	0	0	0	0	0	0	0	0	0	0	0	0	0	0	0
0	0	0	0	0	0	0	0	0	0	0	0	0	0	0	0	0	0	0	0	0
0	0	0	0	0	0	0	0	0	0	0	0	0	0	0	0	0	0	0	0	0
0	0	0	0	0	0	0	0	0	0	0	0	0	0	0	0	0	0	0	0	0
0	0	0	0	0	0	0	0	0	0	0	0	0	0	0	0	0	0	0	0	0
0	0	0	0	0	0	0	0	0	0	0	0	0	0	0	0	0	0	0	0	0
0	0	0	0	0	0	0	0	0	0	0	0	0	0	0	0	0	0	0	0	0
0	0	0	0	0	0	0	0	0	0	0	0	0	0	0	0	0	0	0	0	0
0	0	0	0	0	0	0	0	0	0	0	0	0	0	0	0	0	0	0	0	0
0	0	0	0	0	0	0	0	0	0	0	0	0	0	0	0	0	0	0	0	0
0	0	0	0	0	0	0	0	0	0	0	0	0	0	0	0	0	0	0	0	0

0	0	0	0	0	0	0	0	0	0	0	0	0	0	0	0	0	0	0	0	0
0	0	0	0	0	0	0	0	0	0	0	0	0	0	0	0	0	0	0	0	0
0	0	0	0	0	0	0	0	0	0	0	0	0	0	0	0	0	0	0	0	0
0	0	0	0	0	0	0	0	0	0	0	0	0	0	0	0	0	0	0	0	0
0	0	0	0	0	0	0	0	0	0	0	0	0	0	0	0	0	0	0	0	0
0	0	0	0	0	0	0	0	0	0	0	0	0	0	0	0	0	0	0	0	0
0	0	0	0	0	0	0	0	0	0	0	0	0	0	0	0	0	0	0	0	0
0	0	0	0	0	0	0	0	0	0	0	0	0	0	0	0	0	0	0	0	0
0	0	0	0	0	0	0	0	0	0	0	0	0	0	0	0	0	0	0	0	0
0	0	0	0	0	0	0	0	0	0	0	0	0	0	0	0	0	0	0	0	0
0	0	0	0	0	0	0	0	0	0	0	0	0	0	0	0	0	0	0	0	0
0	0	0	0	0	0	0	0	0	0	0	0	0	0	0	0	0	0	0	0	0
0	0	0	0	0	0	0	0	0	0	0	0	0	0	0	0	0	0	0	0	0
0	0	0	0	0	0	0	0	0	0	0	0	0	0	0	0	0	0	0	0	0
0	0	0	0	0	0	0	0	0	0	0	0	0	0	0	0	0	0	0	0	0
0	0	0	0	0	0	0	0	0	0	0	0	0	0	0	0	0	0	0	0	0
0	0	0	0	0	0	0	0	0	0	0	0	0	0	0	0	0	0	0	0	0
0	0	0	0	0	0	0	0	0	0	0	0	0	0	0	0	0	0	0	0	0
0	0	0	0	0	0	0	0	0	0	0	0	0	0	0	0	0	0	0	0	0
0	0	0	0	0	0	0	0	0	0	0	0	0	0	0	0	0	0	0	0	0
0	0	0	0	0	0	0	0	0	0	0	0	0	0	0	0	0	0	0	0	0
0	0	0	0	0	0	0	0	0	0	0	0	0	0	0	0	0	0	0	0	0
0	0	0	0	0	0	0	0	0	0	0	0	0	0	0	0	0	0	0	0	0
0	0	0	0	0	0	0	0	0	0	0	0	0	0	0	0	0	0	0	0	0
0	0	0	0	0	0	0	0	0	0	0	0	0	0	0	0	0	0	0	0	0
0	0	0	0	0	0	0	0	0	0	0	0	0	0	0	0	0	0	0	0	0
0	0	0	0	0	0	0	0	0	0	0	0	0	0	0	0	0	0	0	0	0
0	0	0	0	0	0	0	0	0	0	0	0	0	0	0	0	0	0	0	0	0
0	0	0	0	0	0	0	0	0	0	0	0	0	0	0	0	0	0	0	0	0
0	0	0	0	0	0	0	0	0	0	0	0	0	0	0	0	0	0	0	0	0
0	0	0	0	0	0	0	0	0	0	0	0	0	0	0	0	0	0	0	0	0
0	0	0	0	0	0	0	0	0	0	0	0	0	0	0	0	0	0	0	0	0
0	0	0	0	0	0	0	0	0	0	0	0	0	0	0	0	0	0	0	0	0
0	0	0	0	0	0	0	0	0	0	0	0	0	0	0	0	0	0	0	0	0
0	0	0	0	0	0	0	0	0	0	0	0	0	0	0	0	0	0	0	0	0
0	0	0	0	0	0	0	0	0	0	0	0	0	0	0	0	0	0	0	0	0

0	0	0	0	0	0	0	0	0	0	0	0	0	0	0	0	0	0	0	0	0
0	0	0	0	0	0	0	0	0	0	0	0	0	0	0	0	0	0	0	0	0
0	0	0	0	0	0	0	0	0	0	0	0	0	0	0	0	0	0	0	0	0
0	0	0	0	0	0	0	0	0	0	0	0	0	0	0	0	0	0	0	0	0
0	0	0	0	0	0	0	0	0	0	0	0	0	0	0	0	0	0	0	0	0
0	0	0	0	0	0	0	0	0	0	0	0	0	0	0	0	0	0	0	0	0
0	0	0	0	0	0	0	0	0	0	0	0	0	0	0	0	0	0	0	0	0
0	0	0	0	0	0	0	0	0	0	0	0	0	0	0	0	0	0	0	0	0
0	0	0	0	0	0	0	0	0	0	0	0	0	0	0	0	0	0	0	0	0
0	0	0	0	0	0	0	0	0	0	0	0	0	0	0	0	0	0	0	0	0
0	0	0	0	0	0	0	0	0	0	0	0	0	0	0	0	0	0	0	0	0
0	0	0	0	0	0	0	0	0	0	0	0	0	0	0	0	0	0	0	0	0
0	0	0	0	0	0	0	0	0	0	0	0	0	0	0	0	0	0	0	0	0
0	0	0	0	0	0	0	0	0	0	0	0	0	0	0	0	0	0	0	0	0
0	0	0	0	0	0	0	0	0	0	0	0	0	0	0	0	0	0	0	0	0
0	0	0	0	0	0	0	0	0	0	0	0	0	0	0	0	0	0	0	0	0
0	0	0.01	0	0	0	0	0	0	0	0	0	0	0	0	0	0	0	0	0	0
0	0	0	0	0	0	0	0	0	0	0	0	0	0	0	0	0	0	0	0	0
0	0	0	0	0	0	0	0	0	0	0	0	0	0	0	0	0	0	0	0	0
0	0	0	0	0	0	0	0	0	0	0	0	0	0	0	0	0	0	0	0	0
0	0	0	0	0	0	0	0	0	0	0	0	0	0	0	0	0	0	0	0	0
0	0	0	0	0	0	0	0	0	0	0	0	0	0	0	0	0	0	0	0	0
0	0	0	0	0	0	0	0	0	0	0	0	0	0	0	0	0	0	0	0	0
0	0	0	0	0	0	0	0	0	0	0	0	0	0	0	0	0	0	0	0	0
0	0	0	0	0	0	0	0	0	0	0	0	0	0	0	0	0	0	0	0	0
0	0	0	0	0	0	0	0	0	0	0	0	0	0	0	0	0	0	0	0	0
0	0	0	0	0	0	0	0	0	0	0	0	0	0	0	0	0	0	0	0	0
0	0	0	0	0	0	0	0	0	0	0	0	0	0	0	0	0	0	0	0	0
0	0	0	0	0	0	0	0	0	0	0	0	0	0	0	0	0	0	0	0	0
0	0	0	0	0	0	0	0	0	0	0	0	0	0	0	0	0	0	0	0	0
0	0	0	0	0	0	0	0	0	0	0	0	0	0	0	0	0	0	0	0	0
0	0	0	0	0	0	0	0	0	0	0	0	0	0	0	0	0	0	0	0	0
0	0	0	0	0	0	0	0	0	0	0	0	0	0	0	0	0	0	0	0	0
0	0	0	0	0	0	0	0	0	0	0	0	0	0	0	0	0	0	0	0	0
0	0	0	0	0	0	0	0	0	0	0	0	0	0	0	0	0	0	0	0	0
0	0	0	0	0	0	0	0	0	0	0	0	0	0	0	0	0	0	0	0	0

0	0	0	0	0	0	0	0	0	0	0	0	0	0	0	0	0	0	0	0	0
0	0	0	0	0	0	0	0	0	0	0	0	0	0	0	0	0	0	0	0	0
0	0	0	0	0	0	0	0	0	0	0	0	0	0	0	0	0	0	0	0	0
0	0	0	0	0	0	0	0	0	0	0	0	0	0	0	0	0	0	0	0	0
0	0	0	0	0	0	0	0	0	0	0	0	0	0	0	0	0	0	0	0	0
0	0	0	0	0	0	0	0	0	0	0	0	0	0	0	0	0	0	0	0	0
0	0	0	0	0	0	0	0	0	0	0	0	0	0	0	0	0	0	0	0	0
0	0	0	0	0	0	0	0	0	0	0	0	0	0	0	0	0	0	0	0	0
0	0	0	0	0	0	0	0	0	0	0	0	0	0	0	0	0	0	0	0	0
0	0	0	0	0	0	0	0	0	0	0	0	0	0	0	0	0	0	0	0	0
0	0	0	0	0	0	0	0	0	0	0	0	0	0	0	0	0	0	0	0	0
0	0	0	0	0	0	0	0	0	0	0	0	0	0	0	0	0	0	0	0	0
0	0	0	0	0	0	0	0	0	0	0	0	0	0	0	0	0	0	0	0	0
0	0	0	0	0	0	0	0	0	0	0	0	0	0	0	0	0	0	0	0	0
0	0	0	0	0	0	0	0	0	0	0	0	0	0	0	0	0	0	0	0	0
0	0	0	0	0	0	0	0	0	0	0	0	0	0	0	0	0	0	0	0	0
0	0	0	0	0	0	0	0	0	0	0	0	0	0	0	0	0	0	0	0	0
0	0	0	0	0	0	0	0	0	0	0	0	0	0	0	0	0	0	0	0	0
0	0	0	0	0	0	0	0	0	0	0	0	0	0	0	0	0	0	0	0	0
0	0	0	0	0	0	0	0	0	0	0	0	0	0	0	0	0	0	0	0	0
0	0	0	0	0	0	0	0	0	0	0	0	0	0	0	0	0	0	0	0	0
0	0	0	0	0	0	0	0	0	0	0	0	0	0	0	0	0	0	0	0	0
0	0	0	0	0	0	0	0	0	0	0	0	0	0	0	0	0	0	0	0	0
0	0	0	0	0	0	0	0	0	0	0	0	0	0	0	0	0	0	0	0	0
0	0	0	0	0	0	0	0	0	0	0	0	0	0	0	0	0	0	0	0	0
0	0	0	0	0	0	0	0	0	0	0	0	0	0	0	0	0	0	0	0	0
0	0	0	0	0	0	0	0	0	0	0	0	0	0	0	0	0	0	0	0	0
0	0	0	0	0	0	0	0	0	0	0	0	0	0	0	0	0	0	0	0	0
0	0	0	0	0	0	0	0	0	0	0	0	0	0	0	0	0	0	0	0	0
0	0	0	0	0	0	0	0	0	0	0	0	0	0	0	0	0	0	0	0	0
0	0	0	0	0	0	0	0	0	0	0	0	0	0	0	0	0	0	0	0	0
0	0	0	0	0	0	0	0	0	0	0	0	0	0	0	0	0	0	0	0	0
0	0	0	0	0	0	0	0	0	0	0	0	0	0	0	0	0	0	0	0	0
0	0	0	0	0	0	0	0	0	0	0	0	0	0	0	0	0	0	0	0	0
0	0	0	0	0	0	0	0	0	0	0	0	0	0	0	0	0	0	0	0	0
0	0	0	0	0	0	0	0	0	0	0	0	0	0	0	0	0	0	0	0	0

0	0	0	0	0	0	0	0	0	0	0	0	0	0	0	0	0	0	0	0	0
0	0	0	0	0	0	0	0	0	0	0	0	0	0	0	0	0	0	0	0	0
0	0	0	0	0	0	0	0	0	0	0	0	0	0	0	0	0	0	0	0	0
0	0	0	0	0	0	0	0	0	0	0	0	0	0	0	0	0	0	0	0	0
0	0	0	0	0	0	0	0	0	0	0	0	0	0	0	0	0	0	0	0	0
0	0	0	0	0	0	0	0	0	0	0	0	0	0	0	0	0	0	0	0	0
0	0	0	0	0	0	0	0	0	0	0	0	0	0	0	0	0	0	0	0	0
0	0	0	0	0	0	0	0	0	0	0	0	0	0	0	0	0	0	0	0	0
0	0	0	0	0	0	0	0	0	0	0	0	0	0	0	0	0	0	0	0	0
0	0	0	0	0	0	0	0	0	0	0	0	0	0	0	0	0	0	0	0	0
0	0	0	0	0	0	0	0	0	0	0	0	0	0	0	0	0	0	0	0	0
0	0	0	0	0	0	0	0	0	0	0	0	0	0	0	0	0	0	0	0	0
0	0	0	0	0	0	0	0	0	0	0	0	0	0	0	0	0	0	0	0	0
0	0	0	0	0	0	0	0	0	0	0	0	0	0	0	0	0	0	0	0	0
0	0	0	0	0	0	0	0	0	0	0	0	0	0	0	0	0	0	0	0	0
0	0	0	0	0	0	0	0	0	0	0	0	0	0	0	0	0	0	0	0	0
0	0	0	0	0	0	0	0	0	0	0	0	0	0	0	0	0	0	0	0	0
0	0	0	0	0	0	0	0	0	0	0	0	0	0	0	0	0	0	0	0	0
0	0	0	0	0	0	0	0	0	0	0	0	0	0	0	0	0	0	0	0	0
0	0	0	0	0	0	0	0	0	0	0	0	0	0	0	0	0	0	0	0	0
0	0	0	0	0	0	0	0	0	0	0	0	0	0	0	0	0	0	0	0	0
0	0	0	0	0	0	0	0	0	0	0	0	0	0	0	0	0	0	0	0	0
0	0	0	0	0	0	0	0	0	0	0	0	0	0	0	0	0	0	0	0	0
0	0	0	0	0	0	0	0	0	0	0	0	0	0	0	0	0	0	0	0	0
0	0	0	0	0	0	0	0	0	0	0	0	0	0	0	0	0	0	0	0	0
0	0	0	0	0	0	0	0	0	0	0	0	0	0	0	0	0	0	0	0	0
0	0	0	0	0	0	0	0	0	0	0	0	0	0	0	0	0	0	0	0	0
0	0	0	0	0	0	0	0	0	0	0	0	0	0	0	0	0	0	0	0	0
0	0	0	0	0	0	0	0	0	0	0	0	0	0	0	0	0	0	0	0	0
0	0	0	0	0	0	0	0	0	0	0	0	0	0	0	0	0	0	0	0	0
0	0	0	0	0	0	0	0	0	0	0	0	0	0	0	0	0	0	0	0	0
0	0	0	0	0	0	0	0	0	0	0	0	0	0	0	0	0	0	0	0	0
0	0	0	0	0	0	0	0	0	0	0	0	0	0	0	0	0	0	0	0	0
0	0	0	0	0	0	0	0	0	0	0	0	0	0	0	0	0	0	0	0	0
0	0	0	0	0	0	0	0	0	0	0	0	0	0	0	0	0	0	0	0	0
0	0	0	0	0	0	0	0	0	0	0	0	0	0	0	0	0	0	0	0	0

0	0	0	0	0	0	0	0	0	0	0	0	0	0	0	0	0	0	0	0	0
0	0	0	0	0	0	0	0	0	0	0	0	0	0	0	0	0	0	0	0	0
0	0	0	0	0	0	0	0	0	0	0	0	0	0	0	0	0	0	0	0	0
0	0	0	0	0	0	0	0	0	0	0	0	0	0	0	0	0	0	0	0	0
0	0	0	0	0	0	0	0	0	0	0	0	0	0	0	0	0	0	0	0	0
0	0	0	0	0	0	0	0	0	0	0	0	0	0	0	0	0	0	0	0	0
0	0	0	0	0	0	0	0	0	0	0	0	0	0	0	0	0	0	0	0	0
0	0	0	0	0	0	0	0	0	0	0	0	0	0	0	0	0	0	0	0	0
0	0	0	0	0	0	0	0	0	0	0	0	0	0	0	0	0	0	0	0	0
0	0	0	0	0	0	0	0	0	0	0	0	0	0	0	0	0	0	0	0	0
0	0	0	0	0	0	0	0	0	0	0	0	0	0	0	0	0	0	0	0	0
0	0	0	0	0	0	0	0	0	0	0	0	0	0	0	0	0	0	0	0	0
0	0	0	0	0	0	0	0	0	0	0	0	0	0	0	0	0	0	0	0	0
0	0	0	0	0	0	0	0	0	0	0	0	0	0	0	0	0	0	0	0	0
0	0	0	0	0	0	0	0	0	0	0	0	0	0	0	0	0	0	0	0	0
0	0	0	0	0	0	0	0	0	0	0	0	0	0	0	0	0	0	0	0	0
0	0	0	0	0	0	0	0	0	0	0	0	0	0	0	0	0	0	0	0	0
0	0	0	0	0	0	0	0	0	0	0	0	0	0	0	0	0	0	0	0	0
0	0	0	0	0	0	0	0	0	0	0	0	0	0	0	0	0	0	0	0	0
0	0	0	0	0	0	0	0	0	0	0	0	0	0	0	0	0	0	0	0	0
0	0	0	0	0	0	0	0	0	0	0	0	0	0	0	0	0	0	0	0	0
0	0	0	0	0	0	0	0	0	0	0	0	0	0	0	0	0	0	0	0	0
0	0	0	0	0	0	0	0	0	0	0	0	0	0	0	0	0	0	0	0	0
0	0	0	0	0	0	0	0	0	0	0	0	0	0	0	0	0	0	0	0	0
0	0	0	0	0	0	0	0	0	0	0	0	0	0	0	0	0	0	0	0	0
0	0	0	0	0	0	0	0	0	0	0	0	0	0	0	0	0	0	0	0	0
0	0	0	0	0	0	0	0	0	0	0	0	0	0	0	0	0	0	0	0	0
0	0	0	0	0	0	0	0	0	0	0	0	0	0	0	0	0	0	0	0	0
0	0	0	0	0	0	0	0	0	0	0	0	0	0	0	0	0	0	0	0	0
0	0	0	0	0	0	0	0	0	0	0	0	0	0	0	0	0	0	0	0	0
0	0	0	0	0	0	0	0	0	0	0	0	0	0	0	0	0	0	0	0	0
0	0	0	0	0	0	0	0	0	0	0	0	0	0	0	0	0	0	0	0	0
0	0	0	0	0	0	0	0	0	0	0	0	0	0	0	0	0	0	0	0	0
0	0	0	0	0	0	0	0	0	0	0	0	0	0	0	0	0	0	0	0	0
0	0	0	0	0	0	0	0	0	0	0	0	0	0	0	0	0	0	0	0	0
0	0	0	0	0	0	0	0	0	0	0	0	0	0	0	0	0	0	0	0	0

0	0	0	0	0	0	0	0	0	0	0	0	0	0	0	0	0	0	0	0	0
0	0	0	0	0	0	0	0	0	0	0	0	0	0	0	0	0	0	0	0	0
0	0	0	0	0	0	0	0	0	0	0	0	0	0	0	0	0	0	0	0	0
0	0	0	0	0	0	0	0	0	0	0	0	0	0	0	0	0	0	0	0	0
0	0	0	0	0	0	0	0	0	0	0	0	0	0	0	0	0	0	0	0	0
0	0	0	0	0	0	0	0	0	0	0	0	0	0	0	0	0	0	0	0	0
0	0	0	0	0	0	0	0	0	0	0	0	0	0	0	0	0	0	0	0	0
0	0	0	0	0	0	0	0	0	0	0	0	0	0	0	0	0	0	0	0	0
0	0	0	0	0	0	0	0	0	0	0	0	0	0	0	0	0	0	0	0	0
0	0	0	0	0	0	0	0	0	0	0	0	0	0	0	0	0	0	0	0	0
0	0	0	0	0	0	0	0	0	0	0	0	0	0	0	0	0	0	0	0	0
0	0	0	0	0	0	0	0	0	0	0	0	0	0	0	0	0	0	0	0	0
0	0	0	0	0	0	0	0	0	0	0	0	0	0	0	0	0	0	0	0	0
0	0	0	0	0	0	0	0	0	0	0	0	0	0	0	0	0	0.01	0	0	0
0	0	0	0	0	0	0	0	0	0	0.03	0	0	0	0	0	0	0	0	0	0
0	0	0	0	0	0	0	0	0	0	0	0	0	0	0	0	0	0	0	0	0
0	0	0	0	0	0	0	0	0	0	0	0	0	0	0	0	0	0	0	0	0
0	0	0	0	0	0	0	0	0	0	0	0	0	0	0	0	0	0	0	0	0
0	0	0	0	0	0	0	0	0	0	0	0	0	0	0	0	0	0	0	0	0
0	0	0	0	0	0	0	0	0	0	0	0	0	0	0	0	0	0	0	0	0
0	0	0	0	0	0	0	0	0	0	0	0	0	0	0	0	0	0	0	0	0
0	0	0	0	0	0	0	0	0	0	0	0	0	0	0	0	0	0	0	0	0
0	0	0	0	0	0	0	0	0	0	0	0	0	0	0	0	0	0	0	0	0
0	0	0	0	0	0	0	0	0	0	0	0	0	0	0	0	0	0	0	0	0
0	0	0	0	0	0	0	0	0	0	0	0	0	0	0	0	0	0	0	0	0
0	0	0	0	0	0	0	0	0	0	0	0	0	0	0	0	0	0	0	0	0
0	0	0	0	0	0	0	0	0	0	0	0	0	0	0	0	0	0	0	0	0
0	0	0	0	0	0	0	0	0	0	0	0	0	0	0	0	0	0	0	0	0
0	0	0	0	0	0	0	0	0	0	0	0	0	0	0	0	0	0	0	0	0
0	0	0	0	0	0	0	0	0	0	0	0	0	0	0	0	0	0	0	0	0
0	0	0	0	0	0	0	0	0	0	0	0	0	0	0	0	0	0	0	0	0
0	0	0	0	0	0	0	0	0	0	0	0	0	0	0	0	0	0	0	0	0
0	0	0	0	0	0	0	0	0	0	0	0	0	0	0	0	0	0	0	0	0
0	0	0	0	0	0	0	0	0	0	0	0	0	0	0	0	0	0	0	0	0
0	0	0	0	0	0	0	0	0	0	0	0	0	0	0	0	0	0	0	0	0
0	0	0	0	0	0	0	0	0	0	0	0	0	0	0	0	0	0	0	0	0

0	0	0	0	0	0	0	0	0	0	0	0	0	0	0	0	0	0	0	0	0
0	0	0	0	0	0	0	0	0	0	0	0	0	0	0	0	0	0	0	0	0
0	0	0	0	0	0	0	0	0	0	0	0	0	0	0	0	0	0	0	0	0
0	0	0	0	0	0	0	0	0	0	0	0	0	0	0	0	0	0	0	0	0
0	0	0	0	0	0	0	0	0	0	0.01	0	0	0	0	0	0.01	0	0	0	0
0.01	0.01	0.01	0	0.01	0	0.01	0.01	0	0	0.02	0	0	0	0.02	0.01	0.01	0.02	0	0	0
0	0	0	0	0	0	0	0	0	0	0	0	0	0	0	0	0	0	0	0	0
0	0	0.03	0.01	0	0.01	0	0	0	0	0	0	0	0	0	0	0	0	0	0	0
0	0	0	0	0	0	0	0	0	0	0	0	0	0	0	0	0	0	0	0	0
0	0	0	0	0	0	0	0	0	0	0	0	0	0	0	0	0	0	0	0	0
0	0	0	0	0	0	0	0	0	0	0	0	0	0	0	0	0	0	0	0	0
0	0	0	0	0	0	0	0	0	0	0	0	0	0	0	0	0	0	0	0	0
0	0	0.01	0	0.01	0	0	0	0	0	0	0	0	0	0	0	0	0	0	0	0

miR_B-	miR_B-	miR_B-	miR_B-	miR_B-	miR_B-	miR_B-	miR_Bl	miR_Bl	miR_Br	miR_Br	miR_Br	miR_Br	miR_Br	miR_Br	miR_Bu	miR_Bu	miR_Bu	miR_Bu	miR_Bu	miR_Bu
0.02	0.01	0.02	0.03	0.03	0.01	0.02	0	0	0.02	0.01	0.03	0.03	0.01	0.04	0.03	0.03	0.03	0.02	0.02	0.03
0	0	0	0	0	0	0	0	0	0.06	0.05	0.02	0.04	0.01	0	0	0	0	0	0.01	0.02
0	0	0	0	0	0	0	0	0	0.01	0.01	0	0	0	0	0	0	0	0	0	0
0	0	0	0	0	0	0	0	0	0	0	0	0	0	0	0.01	0.01	0	0	0	0.01
0	0	0	0	0	0	0	0	0	0	0	0	0	0	0	0	0	0	0	0	0
0.02	0.03	0.02	0.03	0.02	0.02	0.02	0	0	0.01	0.01	0.01	0	0.01	0	0.01	0.02	0.06	0.06	0.01	0.01
0.01	0.01	0	0.01	0.01	0.01	0.01	0	0	0	0	0	0	0	0	0.01	0.01	0	0	0	0
0	0	0.01	0	0.01	0.01	0.01	0	0	0.02	0.01	0	0	0	0	0	0	0	0	0.01	0.01
0	0	0	0	0	0	0	0	0	0	0	0	0	0	0	0	0	0	0	0	0
0	0	0	0	0	0	0	0	0	0	0	0	0	0	0	0	0	0	0	0	0
0	0	0	0	0	0	0	0	0	0	0	0	0	0	0	0	0	0	0	0	0
0	0	0	0	0	0	0	0.01	0	0	0	0	0	0	0	0	0	0	0	0	0
0.02	0.02	0.02	0.01	0.02	0.01	0.02	0	0	0	0	0	0	0	0.01	0	0	0	0	0	0
0	0	0	0	0	0	0	0	0	0	0	0	0	0	0	0	0	0	0	0	0
0	0	0	0	0	0	0	0	0	0	0	0	0	0	0	0	0	0	0	0	0
0	0	0	0	0	0	0	0	0	0	0	0	0	0	0	0	0	0	0	0	0
0	0	0	0	0	0	0	0.01	0.01	0	0	0	0	0.01	0.01	0.01	0.01	0.01	0	0.01	0.01
0	0	0	0	0	0	0	0	0	0	0	0	0	0	0	0	0	0	0	0	0
0	0	0	0	0	0	0	0	0	0	0	0	0	0	0	0	0	0	0	0	0
0	0	0	0	0	0	0	0	0	0	0	0	0	0	0	0	0	0	0	0	0
0	0	0	0	0	0	0	0	0	0	0	0	0	0	0	0	0	0	0	0	0
0	0	0	0	0	0	0	0	0	0	0	0	0	0	0	0	0	0	0	0	0
0	0	0	0	0	0	0	0	0	0	0.01	0	0	0	0	0	0	0	0	0	0
0	0	0	0	0	0	0	0	0	0	0.01	0	0	0	0	0	0	0	0	0	0
0	0	0	0	0	0	0	0	0	0	0	0	0	0	0	0	0	0	0	0	0
0	0	0	0	0	0	0	0	0	0	0	0	0	0	0	0	0	0	0	0	0
0	0	0	0	0	0	0	0	0	0	0	0	0	0	0	0	0	0	0	0	0
0	0	0	0	0	0	0	0	0	0	0	0	0	0	0	0	0	0	0	0	0
0	0	0	0	0	0	0	0	0	0	0	0	0	0	0	0	0	0	0	0	0
0	0	0	0	0	0	0	0	0	0	0	0	0	0	0	0	0	0	0	0	0
0	0	0	0	0	0	0	0	0	0	0	0	0	0	0	0	0	0	0	0	0
0	0	0	0	0	0	0	0	0	0	0	0	0	0	0	0	0	0	0	0	0
0	0	0	0	0	0	0	0	0	0	0	0	0	0	0	0	0	0	0	0	0
0	0	0	0	0	0	0	0	0	0	0.01	0.02	0.01	0	0.05	0	0	0	0	0	0.01
0	0	0	0	0	0	0	0	0	0	0	0	0	0	0	0	0.01	0	0	0	0.01

0	0	0	0	0	0	0	0	0	0	0	0	0	0	0	0	0	0	0	0	0
0	0	0	0	0	0	0	0	0	0	0	0	0	0	0	0	0	0	0	0	0
0	0	0	0	0	0	0	0	0	0	0	0	0	0	0	0	0	0	0	0	0
0	0	0	0	0	0	0	0	0	0	0	0	0	0	0	0	0	0	0	0	0
0	0	0	0	0	0	0	0	0	0	0	0	0	0	0	0	0	0	0	0	0
0	0	0	0	0	0	0	0	0	0	0	0.01	0	0	0	0	0	0	0	0.01	0
0	0	0	0	0	0	0	0	0	0	0	0	0	0	0	0	0	0	0	0	0
0	0	0	0	0	0	0	0	0	0	0	0	0	0	0	0	0	0	0	0	0
0	0	0	0	0	0	0	0	0	0	0	0	0	0	0	0	0	0	0	0	0
0	0	0	0	0	0	0	0	0	0	0	0	0	0	0	0	0	0	0	0	0
0	0	0	0	0	0	0	0	0	0	0	0	0	0	0	0	0	0	0	0	0
0	0	0	0	0	0	0	0	0	0	0	0	0	0	0	0	0	0	0	0	0.01
0.01	0.02	0.01	0.02	0.01	0.01	0.01	0	0	0	0	0	0	0	0	0	0	0.01	0	0.02	0.02
0	0	0	0	0	0	0	0	0	0.08	0.06	0.07	0.13	0.29	0.06	0	0	0	0	0	0
0.19	0.19	0.27	0.14	0.25	0.22	0.23	0.04	0.15	0	0	0	0	0	0	0.21	0.23	0.11	0.19	0.02	0.02
0.14	0.12	0.16	0.28	0.15	0.13	0.11	0.03	0.06	0	0	0	0	0	0	0.06	0.08	0.05	0.06	0.15	0.14
0	0	0	0	0	0	0	0	0	0	0	0	0	0	0	0	0	0	0	0	0
0	0	0	0	0	0	0	0	0	0	0	0	0	0	0	0	0	0	0	0.01	0
0	0	0	0	0	0	0	0	0	0	0	0	0	0	0	0	0	0	0	0	0
0	0	0.02	0.01	0	0.01	0	0	0	0	0	0	0	0	0	0.09	0.03	0.04	0.03	0.01	0.01
0	0	0	0	0	0	0	0	0	0	0	0	0	0	0	0	0	0	0	0	0
0	0	0	0	0	0	0	0	0	0	0	0	0	0	0	0	0	0	0	0	0
0	0	0	0	0	0	0	0	0	0	0	0	0	0	0	0	0	0	0	0	0
0	0	0	0	0	0	0	0	0	0	0	0	0	0	0	0	0	0	0	0	0
0	0	0	0	0	0	0	0	0	0	0	0	0	0	0	0	0	0	0	0	0
0	0	0	0	0	0	0	0	0	0	0	0	0	0	0	0	0	0	0	0	0
0	0	0	0	0	0	0	0	0	0	0	0	0	0	0	0	0	0	0	0	0
0.07	0.04	0.03	0.05	0.04	0.05	0.07	0	0	0	0	0	0	0	0	0	0	0	0	0	0
0	0	0	0	0	0	0	0	0	0	0	0	0	0	0	0	0	0	0	0	0
0	0	0	0	0	0	0	0	0	0	0	0	0	0	0	0	0	0	0	0.01	0
0	0	0	0	0	0	0	0	0	0	0	0	0	0	0	0.01	0	0	0	0	0
0	0	0	0	0	0	0	0	0	0	0	0	0	0	0	0	0	0	0	0	0
0	0	0	0	0	0	0	0	0	0	0	0	0	0	0	0	0	0	0	0	0
0.01	0.03	0.02	0.01	0.01	0.02	0.01	0	0	0	0	0	0	0	0	0.04	0.02	0	0.05	0	0
0.02	0.03	0.02	0.03	0.01	0.06	0.01	0.03	0.03	0.01	0.01	0.06	0.03	0.03	0.07	0.03	0.02	0.03	0.01	0.02	0.07
0	0	0	0	0	0	0	0	0.01	0	0	0	0	0	0.01	0	0.01	0.01	0.01	0	0

0.07	0.08	0.06	0.05	0.04	0.12	0.07	0.26	0.15	0.09	0.06	0.13	0.1	0.12	0.25	0.13	0.18	0.1	0.1	0.05	0.12
0	0	0	0	0	0	0	0.02	0.01	0	0	0	0.01	0.01	0	0.06	0.04	0.02	0.01	0.02	0
0	0	0	0	0	0	0	0	0.01	0.02	0.01	0.01	0.01	0.01	0.01	0.01	0.01	0.01	0.01	0.01	0.02
0	0	0	0	0	0	0	0	0.01	0.01	0	0	0	0	0	0	0	0	0.01	0	0
0	0	0	0	0	0	0	0	0	0	0	0	0	0	0	0	0	0	0	0	0
0	0	0	0	0	0	0	0	0	0	0	0	0	0	0	0	0	0	0	0	0
0	0	0	0	0	0	0	0	0	0	0.01	0.02	0	0	0	0	0	0	0	0	0
0	0	0	0	0	0	0	0	0	0	0.01	0	0	0	0	0	0	0	0	0	0
0	0	0	0	0	0	0	0	0	0	0	0	0	0	0	0	0	0	0	0	0
0	0	0	0	0	0	0	0	0	0	0	0	0	0	0	0	0	0	0	0	0
0	0	0	0	0	0	0	0.01	0	0	0	0	0	0	0.01	0.01	0	0	0	0	0
0	0	0	0	0	0	0	0	0	0	0	0	0	0	0	0	0	0	0	0	0
0	0	0	0	0	0	0	0	0	0	0	0	0	0	0	0	0	0	0	0	0
0	0	0	0	0	0	0	0	0	0	0	0	0	0	0	0	0	0	0	0	0
0	0	0	0	0	0	0	0.03	0.02	0	0	0	0	0.01	0	0.01	0	0	0	0.01	0.02
0	0	0	0	0	0	0	0	0	0	0	0	0	0	0	0	0	0	0	0	0
0	0	0	0	0	0	0	0	0	0	0	0	0	0	0	0	0	0	0	0	0
0	0	0	0	0	0	0	0	0	0	0	0	0	0	0	0	0	0	0	0	0
0	0	0	0.01	0	0.01	0	0.01	0	0.01	0.02	0	0.01	0	0.01	0.02	0.02	0.01	0.01	0.01	0.01
0	0	0	0	0	0	0	0	0	0	0	0	0	0	0	0	0	0	0	0.01	0
0	0	0	0	0	0	0	0	0	0	0	0	0	0.03	0.01	0	0	0	0	0	0
0	0	0	0	0	0	0	0	0	0	0	0	0	0	0	0	0	0	0	0	0
0	0	0	0	0	0	0	0	0	0	0	0	0	0	0	0	0	0	0.01	0	0
0	0	0	0	0	0	0	0.01	0	0	0	0	0	0	0	0	0	0	0	0.01	0.02
0	0	0	0	0	0	0	0	0	0	0	0	0	0	0	0	0	0	0	0	0
0	0	0	0	0	0	0	0	0	0.05	0	0	0	0	0.01	0	0	0	0	0	0
0	0	0	0	0	0	0	0	0	0	0	0	0	0	0	0	0	0	0	0	0
0	0	0	0	0	0	0	0	0	0	0	0	0	0	0	0	0	0	0	0	0
0	0	0	0	0	0	0	0	0	0	0	0	0	0	0	0	0	0	0	0	0
0	0	0	0	0	0	0	0	0	0	0	0	0	0	0	0	0	0	0	0	0
0	0	0	0	0	0	0	0	0	0	0	0	0	0	0	0	0	0	0	0	0
0	0	0	0	0	0	0	0	0	0	0	0	0	0	0	0	0	0	0	0	0
0	0	0	0	0	0	0	0	0	0	0	0	0	0	0	0	0	0	0	0	0
0	0	0	0	0	0	0	0	0.01	0	0	0	0	0	0.01	0	0	0.06	0.03	0	0
0	0.01	0	0	0	0	0	0.05	0.03	0	0	0	0	0	0	0.01	0.01	0.09	0.05	0	0.01
0	0	0	0	0	0	0	0	0	0.01	0	0	0	0.01	0	0	0	0	0	0	0

0	0	0	0	0	0	0	0	0	0.01	0	0	0	0	0	0	0	0	0	0	0
0	0	0	0	0	0	0	0	0	0.2	0.39	0.02	0.03	0.04	0.03	0	0	0	0	0	0
0	0	0	0	0	0	0	0	0	0	0	0	0	0	0	0	0	0	0	0	0
0	0	0	0	0	0	0	0	0	0	0	0	0	0.01	0.01	0	0	0	0	0	0
0	0	0	0	0	0	0	0	0	0	0	0	0	0	0	0	0	0	0	0	0
0	0	0	0	0	0	0	0	0	0	0	0	0.05	0	0.01	0	0	0	0	0	0
0	0	0	0	0	0	0	0	0	0	0	0	0	0	0	0	0	0	0	0	0
0	0	0	0	0	0	0	0	0	0	0	0	0	0	0	0	0	0	0	0	0
0	0	0	0	0	0	0	0	0	0	0	0	0	0	0	0	0	0	0	0	0
0	0	0	0	0	0	0	0.02	0.01	0	0	0.01	0	0.01	0	0.05	0.03	0.04	0.02	0.01	0.01
0	0	0	0	0	0	0	0	0	0	0	0	0	0	0	0	0	0	0	0	0
0.03	0.05	0.06	0.04	0.02	0.06	0.06	0.24	0.21	0.07	0.01	0.14	0.08	0.08	0.02	0.04	0.01	0.02	0.07	0.21	0.08
0	0	0	0	0	0	0	0.01	0	0	0	0	0	0	0	0.01	0.01	0	0	0	0
0	0	0	0	0	0	0	0	0	0	0	0	0	0	0	0	0	0	0	0	0
0	0	0	0	0	0	0	0	0	0	0	0	0	0	0	0	0	0	0	0	0
0	0	0	0	0	0	0	0	0	0	0	0	0	0	0	0	0	0	0	0	0
0	0	0	0	0	0	0	0	0	0	0	0	0	0	0	0	0	0	0	0	0
0	0	0	0	0	0	0	0	0	0	0	0	0	0	0	0	0	0	0	0	0
0	0	0	0	0	0	0	0	0	0	0	0	0	0	0	0	0	0	0	0	0
0	0	0	0	0	0	0	0	0	0	0	0	0	0	0	0	0	0	0	0	0
0	0	0	0	0	0	0	0	0	0	0	0	0	0	0	0	0	0	0	0	0
0	0	0	0	0	0	0	0	0	0	0	0	0	0	0	0	0	0	0	0	0
0	0	0	0	0	0	0	0	0	0	0	0	0	0	0	0	0	0	0	0	0
0	0	0.01	0	0	0	0	0	0	0	0	0.01	0.03	0	0.01	0	0	0	0	0.02	0.02
0	0	0	0	0	0	0	0	0	0	0	0	0	0	0	0	0	0	0	0	0
0	0	0	0	0	0	0	0	0	0	0	0	0	0	0	0	0	0	0	0	0
0	0	0	0	0	0	0	0	0	0	0	0	0	0	0	0	0	0	0	0	0
0	0	0	0	0	0	0	0	0	0	0	0	0	0	0	0	0	0	0	0	0
0	0	0	0	0	0	0	0	0	0	0	0	0	0	0	0	0	0	0	0	0
0	0	0	0	0	0	0	0	0	0	0	0	0	0	0	0	0	0	0	0	0
0	0	0	0	0	0	0	0	0	0	0	0	0	0	0	0	0	0	0	0	0
0	0	0	0	0	0	0	0	0	0.01	0.03	0.01	0.02	0	0	0	0	0	0	0	0.01
0	0	0	0	0	0	0	0	0	0.02	0.02	0	0	0	0.01	0	0	0	0	0.01	0.01
0	0	0.01	0.01	0	0	0	0	0	0	0	0.03	0.06	0.02	0.03	0	0	0	0	0.01	0.02
0	0	0	0	0	0	0	0.01	0	0	0	0	0	0	0	0.01	0	0.01	0.01	0.01	0
0.06	0.08	0.05	0.05	0.05	0.05	0.05	0	0	0.01	0.02	0	0.01	0	0.01	0.01	0.01	0.02	0	0.03	0.01

0.02	0.03	0.01	0.01	0.02	0.02	0.02	0	0	0	0	0	0.01	0	0	0	0.01	0.01	0.01	0.01	0.01
0.01	0	0.01	0.01	0	0	0	0	0	0.04	0.07	0.06	0.1	0.02	0.02	0	0	0.01	0.02	0.01	0.01
0	0	0	0	0	0	0	0	0	0.01	0.03	0.01	0.01	0.01	0.01	0	0	0	0	0.01	0.01
0	0	0	0	0	0	0	0	0	0	0	0	0	0	0	0	0	0	0	0	0
0.01	0	0.01	0.01	0.01	0	0.01	0	0	0	0	0	0	0	0	0	0	0	0	0	0.01
0	0	0	0	0	0	0	0	0	0	0	0	0	0	0	0	0	0	0	0	0
0	0	0	0	0	0	0	0	0	0	0	0	0	0	0	0	0	0	0	0	0
0	0	0	0	0	0	0	0	0	0	0	0	0	0	0	0	0	0	0	0	0
0	0	0	0	0	0	0	0	0	0	0	0	0	0	0	0	0	0	0	0	0
0	0	0	0	0	0	0	0	0	0	0	0	0	0	0	0	0	0	0	0	0
0.04	0.03	0.02	0.04	0.03	0.02	0.03	0.02	0.03	0	0	0.01	0.03	0	0	0.01	0.01	0	0.02	0.02	0.01
0.13	0.12	0.05	0.04	0.11	0.07	0.12	0.03	0.08	0.01	0	0.05	0.03	0.01	0.06	0.01	0.01	0	0.01	0.03	0.05
0.02	0.01	0	0	0.01	0	0.01	0	0	0	0	0	0	0	0.01	0	0	0	0.01	0	0
0	0	0	0	0	0	0	0	0	0	0	0	0	0	0	0	0	0	0	0	0
0	0	0	0	0	0	0	0	0	0	0	0	0	0	0	0	0.01	0	0	0	0
0	0	0	0	0	0	0	0	0	0	0	0	0	0	0.01	0	0	0	0	0	0
0	0	0	0	0	0	0	0	0	0	0	0	0	0	0	0	0	0	0	0	0
0	0	0	0	0	0	0	0	0	0	0	0	0	0	0	0	0	0	0	0	0
0	0	0	0	0	0	0	0	0	0	0	0	0	0	0	0	0	0	0	0	0
0	0	0	0	0	0	0	0	0	0	0	0	0	0	0	0	0	0	0	0	0
0	0	0	0	0	0	0	0	0	0.01	0	0.04	0.01	0	0	0	0	0	0	0	0
0	0	0	0	0	0	0	0	0	0	0	0	0	0	0	0	0	0	0.01	0	0
0.01	0	0	0	0	0	0	0	0	0	0	0	0	0	0	0.01	0	0	0	0	0
0.01	0.02	0.01	0.01	0.02	0.02	0.01	0.01	0	0	0	0	0.01	0.02	0.01	0.01	0.01	0.01	0.01	0.01	0.02
0.03	0.03	0.04	0.02	0.05	0.02	0.03	0	0	0	0.01	0.01	0.02	0.02	0.01	0.01	0	0	0.01	0	0.02
0	0	0	0	0	0	0	0	0	0	0	0	0.01	0	0	0	0	0	0	0	0
0	0	0.01	0.01	0	0	0.01	0	0.01	0.01	0	0	0.01	0.02	0.04	0	0	0	0.01	0.02	0.02
0	0	0	0	0	0	0	0	0	0.01	0	0	0	0	0	0	0	0	0	0	0.01
0	0	0	0	0	0	0	0	0	0	0	0	0	0	0	0	0	0	0	0	0
0	0	0	0	0	0	0	0	0	0	0	0	0	0	0	0	0	0	0	0	0
0	0	0	0	0	0	0	0	0	0	0	0	0	0	0	0	0	0	0	0	0
0	0	0	0	0	0	0	0	0	0	0	0	0	0	0	0	0	0	0	0	0
0	0	0	0	0	0	0	0	0	0	0	0	0	0	0	0	0	0	0	0	0
0	0	0	0	0	0	0	0	0	0	0	0	0	0	0	0	0	0	0	0	0
0	0	0	0	0	0	0	0	0	0	0	0	0	0	0	0	0	0	0	0	0
0	0	0	0	0	0	0	0	0	0	0	0	0	0	0	0	0	0	0	0	0

0	0	0	0	0	0	0	0	0	0	0	0	0	0	0	0	0	0	0	0	0
0	0	0	0	0	0	0	0	0	0	0	0	0	0	0	0	0	0	0	0	0
0	0	0	0	0	0	0	0	0	0	0	0	0	0	0	0	0	0	0	0	0
0	0	0	0	0	0	0	0	0	0	0	0	0	0	0	0	0	0	0	0	0
0	0	0	0	0	0	0	0	0	0	0	0	0	0	0	0	0	0	0	0	0
0	0	0	0	0	0	0	0	0	0	0	0	0	0	0	0	0	0	0	0	0
0	0	0	0	0	0	0	0	0	0	0	0	0	0	0	0	0	0	0	0	0
0	0	0	0	0	0	0	0	0	0	0	0	0	0	0	0	0	0	0	0	0
0	0	0	0	0	0	0	0	0	0	0	0	0	0	0	0	0	0	0	0	0
0	0	0	0	0	0	0	0	0	0	0	0	0	0	0	0	0	0	0	0	0
0	0	0	0	0	0	0	0	0	0	0	0	0	0.01	0	0	0	0	0	0	0
0	0	0	0	0	0	0	0	0	0	0	0	0	0	0	0	0	0	0	0	0
0	0	0	0	0	0	0	0	0	0	0	0	0	0	0	0	0	0	0	0	0
0	0	0	0	0	0	0	0	0	0	0	0	0	0.01	0	0	0	0	0	0	0
0	0	0	0	0	0	0	0	0	0	0	0	0	0	0	0	0	0	0	0	0
0.01	0	0	0	0	0	0	0	0	0	0	0	0	0	0	0	0	0	0	0	0
0	0	0	0	0	0	0	0	0	0	0	0	0	0	0	0	0	0	0	0	0
0	0	0	0	0	0	0	0	0	0	0	0	0	0	0	0	0	0	0	0	0
0	0	0	0	0	0	0	0	0	0	0	0	0	0	0	0	0	0	0	0	0
0	0	0	0	0	0	0	0	0	0	0	0	0	0	0	0	0	0	0	0	0
0	0	0	0	0	0	0	0	0	0	0	0	0	0	0	0	0	0	0	0	0
0	0	0	0	0	0	0	0	0	0	0	0	0	0	0	0	0	0	0	0	0
0	0	0	0	0	0	0	0	0	0	0	0	0	0	0	0	0	0	0	0	0
0	0	0	0	0	0	0	0	0	0	0	0	0	0	0	0	0	0	0	0	0
0	0	0	0	0	0	0	0	0	0	0	0	0	0	0	0	0	0	0	0	0
0	0	0	0	0	0	0	0	0	0	0	0	0	0	0	0	0	0	0	0	0
0	0	0	0	0	0	0	0	0	0	0	0	0	0	0	0	0	0	0	0	0
0	0	0	0	0	0	0	0	0	0	0	0	0	0	0	0	0	0	0	0	0
0	0	0	0	0	0	0	0	0	0	0	0	0	0	0	0	0	0	0	0	0
0	0	0	0	0	0	0	0	0	0	0	0	0	0	0	0	0	0	0	0	0
0	0	0	0	0	0	0	0	0	0	0	0	0	0	0	0	0	0	0	0	0
0	0	0	0	0	0	0	0	0	0	0	0	0	0	0	0	0	0	0	0	0
0	0	0	0	0	0	0	0	0	0	0	0	0	0	0	0	0	0	0	0	0
0	0	0	0	0	0	0	0	0	0	0	0	0	0	0	0	0	0	0	0	0
0	0	0	0	0	0	0	0	0	0	0	0	0	0	0	0	0	0	0	0	0
0	0	0	0	0	0	0	0	0	0	0	0	0	0	0	0	0	0	0	0	0

0	0	0	0	0	0	0	0	0	0	0	0	0	0	0	0	0	0	0	0	0
0	0	0	0	0	0	0	0	0	0	0	0	0	0	0	0	0	0	0	0	0
0	0	0	0.01	0	0	0	0	0	0	0	0	0	0	0	0	0	0	0	0	0
0	0	0	0	0	0	0	0	0	0	0	0	0	0	0	0	0	0	0	0	0
0	0	0	0	0	0	0	0	0	0.04	0	0	0	0	0	0	0	0	0	0	0
0	0	0	0	0	0	0	0	0	0	0	0	0	0	0	0	0	0	0	0	0
0	0	0	0	0	0	0	0	0	0	0	0	0	0	0	0	0	0	0	0	0
0	0	0	0	0	0	0	0	0	0	0	0	0	0	0	0	0	0	0	0	0
0	0	0	0	0	0	0	0	0	0	0	0	0	0	0	0	0	0	0.01	0	0
0	0	0	0	0	0	0	0	0	0	0	0	0	0	0	0	0	0	0	0	0
0	0	0	0	0	0	0	0	0	0	0	0	0	0	0	0	0	0	0	0	0
0	0	0	0	0	0	0	0	0	0	0	0	0	0	0	0	0	0	0	0	0
0	0	0	0	0	0	0	0	0	0	0	0	0	0	0	0	0	0	0	0	0
0	0	0	0	0	0	0	0	0	0	0	0	0	0	0	0	0	0	0	0	0
0	0	0	0	0	0	0	0	0	0	0	0	0	0	0	0	0	0	0	0	0
0	0	0	0	0	0	0	0	0	0	0	0	0	0	0	0	0	0	0	0	0
0	0	0	0	0	0	0	0	0	0	0	0	0	0	0	0	0	0	0	0	0
0	0	0	0	0	0	0	0	0	0	0	0	0	0	0	0	0	0	0	0	0
0	0	0	0	0	0	0	0	0	0	0	0	0	0	0	0	0	0	0	0	0
0	0	0	0	0	0	0	0	0	0	0	0	0	0	0	0	0	0	0	0	0
0	0	0	0	0	0	0	0	0	0	0	0	0	0	0	0	0	0	0	0	0
0	0	0	0	0	0	0	0	0	0	0	0	0	0	0	0	0	0	0	0	0
0	0	0	0	0	0	0	0	0	0	0	0	0	0	0	0	0	0	0	0	0
0	0	0	0	0	0	0	0	0	0	0	0	0	0	0.01	0	0	0	0	0	0
0	0	0	0	0	0	0	0	0	0	0	0	0	0	0	0	0	0	0	0	0.01
0	0	0	0	0	0	0	0.01	0.01	0.02	0	0	0	0	0	0	0	0	0	0	0
0	0	0	0	0	0	0	0	0	0	0.01	0.01	0	0.01	0.02	0	0	0	0.01	0.01	0
0	0	0	0	0	0	0	0	0	0	0	0	0	0	0	0	0	0	0	0	0
0	0	0	0	0	0	0	0	0	0	0	0	0	0	0	0	0	0	0	0	0
0	0	0	0	0	0	0	0	0	0	0	0	0	0	0	0	0	0	0	0	0
0	0	0	0	0	0	0	0	0	0	0	0	0	0	0	0	0	0	0	0	0
0	0	0	0	0	0	0	0	0	0	0	0	0	0	0	0	0	0	0	0	0
0	0	0	0	0	0	0	0	0	0	0	0	0	0	0	0	0	0	0	0	0
0	0	0	0	0	0	0	0	0	0	0	0	0	0	0	0	0	0	0	0	0
0	0	0	0	0	0	0	0	0	0	0	0	0	0	0	0	0	0	0	0	0
0	0	0	0	0	0	0	0	0	0	0	0	0	0	0	0	0	0	0	0	0

0	0	0	0	0	0	0	0	0	0	0	0	0	0	0	0	0	0	0	0	0
0	0	0	0	0	0	0	0	0	0	0	0	0	0	0	0	0	0	0	0	0
0	0	0	0	0	0	0	0	0	0	0	0	0	0	0	0	0	0	0	0	0
0	0	0	0	0	0	0	0	0	0	0	0	0	0	0	0	0	0	0	0	0
0	0	0	0	0	0	0	0	0	0	0	0	0	0.01	0	0	0	0	0	0	0
0	0	0	0	0	0	0	0	0	0	0	0	0	0	0	0	0	0	0	0	0
0	0	0	0	0	0	0	0	0	0	0	0	0	0	0	0	0	0	0	0	0
0	0	0	0	0	0	0	0	0	0	0	0	0	0	0	0	0	0	0	0	0
0	0	0	0	0	0	0	0	0	0	0	0	0	0	0	0	0	0	0	0	0
0	0	0	0	0	0	0	0	0	0	0	0	0	0	0	0	0	0	0	0	0
0	0	0	0	0	0	0	0	0	0	0	0	0	0	0	0	0	0	0	0	0
0	0	0	0	0	0	0	0	0	0	0	0	0	0	0	0	0	0	0	0	0
0	0	0	0	0	0	0	0	0	0	0	0	0	0	0	0	0	0	0	0	0
0	0	0	0	0	0	0	0	0	0	0	0	0	0	0	0	0	0	0	0	0
0	0	0	0	0	0	0	0	0	0	0	0	0	0	0	0	0	0	0	0	0
0	0	0	0	0	0	0	0	0	0	0	0	0	0	0	0	0	0	0	0	0
0	0	0	0	0	0	0	0	0	0	0	0	0	0	0	0	0	0	0	0	0
0	0	0	0	0	0	0	0	0	0	0	0	0	0	0	0	0	0	0	0	0
0	0	0	0	0	0	0	0	0	0	0	0	0	0	0	0	0	0	0	0	0
0	0	0	0	0	0	0	0	0	0	0	0	0	0	0	0	0	0	0	0	0
0	0	0	0	0	0	0	0	0	0	0	0	0	0	0	0	0	0	0	0	0
0	0	0	0	0	0	0	0	0	0	0	0	0	0	0	0	0	0	0	0	0
0	0	0	0	0	0	0	0	0	0	0	0	0	0	0	0	0	0	0	0	0
0	0	0	0	0	0	0	0	0	0	0	0	0	0	0	0	0	0	0	0	0
0	0	0	0	0	0	0	0	0	0	0	0	0	0	0	0	0	0	0	0	0
0	0	0	0	0	0	0	0	0	0	0	0	0	0	0	0	0	0	0	0	0
0	0	0	0	0	0	0	0	0	0	0	0	0	0	0	0	0	0	0	0	0
0	0	0	0	0	0	0	0	0	0	0	0	0	0	0	0	0	0	0	0	0
0	0	0	0	0	0	0	0	0	0	0	0	0	0	0	0	0	0	0	0	0
0	0	0	0	0	0	0	0	0	0	0	0	0	0	0	0	0	0	0	0	0
0	0	0	0	0	0	0	0	0	0	0	0	0	0	0	0	0	0	0	0	0
0	0	0	0	0	0	0	0	0	0	0	0	0	0	0	0	0	0	0	0	0
0	0	0	0	0	0	0	0	0	0	0	0	0	0	0	0	0	0	0	0	0
0	0	0	0	0	0	0	0	0	0	0	0	0	0	0	0	0	0	0	0	0
0	0	0	0	0	0	0	0	0	0.01	0	0	0	0	0	0	0	0	0	0	0
0	0	0	0	0	0	0	0	0	0	0	0	0	0	0	0	0	0	0	0	0

0	0	0	0	0	0	0	0	0	0	0	0	0	0	0	0	0	0	0	0	0
0	0	0	0	0	0	0	0	0	0	0	0	0	0	0	0	0	0	0	0	0
0	0	0	0	0	0	0	0	0	0	0	0	0	0	0	0	0	0	0	0	0
0	0	0	0	0	0	0	0	0	0	0	0	0	0	0	0	0	0	0	0	0
0	0	0	0	0	0	0	0	0	0	0	0	0	0	0	0	0	0	0	0	0
0	0	0	0	0	0	0	0	0	0	0	0	0	0	0	0	0	0	0	0	0
0	0	0	0	0	0	0	0	0	0	0	0	0	0	0	0	0	0	0	0	0
0	0	0	0	0	0	0	0	0	0	0	0	0	0	0	0	0	0	0	0	0
0	0	0	0	0	0	0	0	0	0	0	0	0	0	0	0	0	0	0	0	0
0	0	0	0	0	0	0	0	0	0	0	0	0	0	0	0	0	0	0	0	0
0	0	0	0	0	0	0	0	0	0	0	0	0	0	0	0	0	0	0	0	0
0	0	0	0	0	0	0	0	0	0	0	0	0	0	0	0	0	0	0	0	0
0	0	0	0	0	0	0	0	0	0	0	0	0	0	0	0	0	0	0	0	0
0	0	0	0	0	0	0	0	0	0	0	0	0	0	0	0	0	0	0	0	0
0	0	0	0	0	0	0	0	0	0	0	0	0	0	0	0	0	0	0	0	0
0	0	0	0	0	0	0	0	0	0	0	0	0	0	0	0	0	0	0	0	0
0	0	0	0	0	0	0	0	0	0	0	0	0	0	0	0	0	0	0	0	0
0	0	0	0	0	0	0	0	0	0	0	0	0	0	0	0	0	0	0	0	0
0	0	0	0	0	0	0	0	0	0	0	0	0	0	0	0	0	0	0	0	0
0	0	0	0	0	0	0	0	0	0	0	0	0	0	0	0	0	0	0	0	0
0	0	0	0	0	0	0	0	0	0	0	0	0	0	0	0	0	0	0	0	0
0	0	0	0	0	0	0	0	0	0	0	0	0	0	0	0	0	0	0	0	0
0	0	0	0	0	0	0	0	0	0	0	0	0	0	0	0	0	0	0	0	0
0	0	0	0	0	0	0	0	0	0	0	0	0	0	0	0	0	0	0	0	0
0	0	0	0	0	0	0	0	0	0	0	0	0	0	0	0	0	0	0	0	0
0	0	0	0	0	0	0	0	0	0	0	0	0	0	0	0	0	0	0	0	0
0	0	0	0	0	0	0	0	0	0	0	0	0	0	0	0	0	0	0	0	0
0	0	0	0	0	0	0	0	0	0	0	0	0	0	0	0	0	0	0	0	0
0	0	0	0	0	0	0	0	0	0	0	0	0	0	0	0	0	0	0	0	0
0	0	0	0	0	0	0	0	0	0	0	0	0	0	0	0	0	0	0	0	0
0	0	0	0	0	0	0	0	0	0	0	0	0	0	0	0	0	0	0	0	0
0	0	0	0	0	0	0	0	0	0	0	0	0	0	0	0	0	0	0	0	0
0	0	0	0	0	0	0	0	0	0	0	0	0	0	0	0	0	0	0	0	0
0	0	0	0	0	0	0	0	0	0	0	0	0	0	0	0	0	0	0	0	0
0	0	0	0	0	0	0	0	0	0	0	0	0	0	0	0	0	0	0	0	0
0	0	0	0	0	0	0	0	0	0	0	0	0	0	0	0	0	0	0	0	0

0	0	0	0	0	0	0	0	0	0	0	0	0	0	0	0	0	0	0	0	0
0	0	0	0	0	0	0	0	0	0	0	0	0	0	0	0	0	0	0	0	0
0	0	0	0	0	0	0	0	0	0	0	0	0	0	0	0	0	0	0	0	0
0	0	0	0	0	0	0	0	0	0	0	0	0	0	0	0	0	0	0	0	0
0	0	0	0	0	0	0	0	0	0	0	0	0	0	0	0	0	0	0	0	0
0	0	0	0	0	0	0	0	0	0	0	0	0	0	0	0	0	0	0	0	0
0	0	0	0	0	0	0	0	0	0	0	0	0	0	0	0	0	0	0	0	0
0	0	0	0	0	0	0	0	0	0	0	0	0	0	0	0	0	0	0	0	0
0	0	0	0	0	0	0	0	0	0	0	0	0	0	0	0	0	0	0	0	0
0	0	0	0	0	0	0	0	0	0	0	0	0	0	0	0	0	0	0	0	0
0	0	0	0	0	0	0	0	0	0	0	0	0	0	0	0	0	0	0	0	0
0	0	0	0	0	0	0	0	0	0	0	0	0	0	0	0	0	0	0	0	0
0	0	0	0	0	0	0	0	0	0	0	0	0	0	0	0	0	0	0	0	0
0	0	0	0	0	0	0	0	0	0	0	0	0	0	0	0	0	0	0	0	0
0	0	0	0	0	0	0	0	0	0	0	0	0	0	0	0	0	0	0	0	0
0	0	0	0	0	0	0	0	0	0	0	0	0	0	0	0	0	0	0	0	0
0	0	0	0	0	0	0	0	0	0	0	0	0	0	0	0	0	0	0	0	0
0	0	0	0	0	0	0	0	0	0	0	0	0	0	0	0	0	0	0	0	0
0	0	0	0	0	0	0	0	0	0	0	0	0	0	0	0	0	0	0	0	0
0	0	0	0	0	0	0	0	0	0	0	0	0	0	0	0	0	0	0	0	0
0	0	0	0	0	0	0	0	0	0	0	0	0	0	0	0	0	0	0	0	0
0	0	0	0	0	0	0	0	0	0	0	0	0	0	0	0	0	0	0	0	0
0	0	0	0	0	0	0	0	0	0	0	0	0	0	0	0	0	0	0	0	0
0	0	0	0	0	0	0	0	0	0	0	0	0	0	0	0	0	0	0	0	0
0	0	0	0	0	0	0	0	0	0	0	0	0	0	0	0	0	0	0	0	0
0	0	0	0	0	0	0	0	0	0	0	0	0	0	0	0	0	0	0	0	0
0	0	0	0	0	0	0	0	0	0	0	0	0	0	0	0	0	0	0	0	0
0	0	0	0	0	0	0	0	0	0	0	0	0	0	0	0	0	0	0	0	0
0	0	0	0	0	0	0	0	0	0	0	0	0	0	0	0	0	0	0	0	0
0	0	0	0	0	0	0	0	0	0	0	0	0	0	0	0	0	0	0	0	0
0	0	0	0	0	0	0	0	0	0	0	0	0	0	0	0	0	0	0	0	0
0	0	0	0	0	0	0	0	0	0	0	0	0	0	0	0	0	0	0	0	0
0	0	0	0	0	0	0	0	0	0	0	0	0	0	0	0	0	0	0	0	0
0	0	0	0	0	0	0	0	0	0	0	0	0	0	0	0	0	0	0	0	0
0	0	0	0	0	0	0	0	0	0	0	0	0	0	0	0	0	0	0	0	0
0	0	0	0	0	0	0	0	0	0	0	0	0	0	0	0	0	0	0	0	0

0	0	0	0	0	0	0	0	0	0	0	0	0	0	0	0	0	0	0	0	0
0	0	0	0	0	0	0	0	0	0	0	0	0	0	0	0	0	0	0	0	0
0	0	0	0	0	0	0	0	0	0	0	0	0	0	0	0	0	0	0	0	0
0	0	0	0	0	0	0	0	0	0	0	0	0	0	0	0	0	0	0	0	0
0	0	0	0	0	0	0	0	0	0	0	0	0	0	0	0	0	0	0	0	0
0	0	0	0	0	0	0	0	0	0	0	0	0	0	0	0	0	0	0	0	0
0	0	0	0	0	0	0	0	0	0	0	0	0	0	0	0	0	0	0	0	0
0	0	0	0	0	0	0	0	0	0	0	0	0	0	0	0	0	0	0	0	0
0	0	0	0	0	0	0	0	0	0	0	0	0	0	0	0	0	0	0	0	0
0	0	0	0	0	0	0	0	0	0	0	0	0	0	0	0	0	0	0	0	0
0	0	0	0	0	0	0	0	0	0	0	0	0	0	0	0	0	0	0	0	0
0	0	0	0	0	0	0	0	0	0	0	0	0	0	0	0	0	0	0	0	0
0	0	0	0	0	0	0	0	0	0	0	0	0	0	0	0	0	0	0	0	0
0	0	0	0	0	0	0	0	0	0	0	0	0	0	0	0	0	0	0	0	0
0	0	0	0	0	0	0	0	0	0	0	0	0	0	0	0	0	0	0	0	0
0	0	0	0	0	0	0	0	0	0	0	0	0	0	0	0	0	0	0	0	0
0	0	0	0	0	0	0	0	0	0	0	0	0	0	0	0	0	0	0	0	0
0	0	0	0	0	0	0	0	0	0	0	0	0	0	0	0	0	0	0	0	0
0	0	0	0	0	0	0	0	0	0	0	0	0	0	0	0	0	0	0	0	0
0	0	0	0	0	0	0	0	0	0	0	0	0	0	0	0	0	0	0	0	0
0	0	0	0	0	0	0	0	0	0	0	0	0	0	0	0	0	0	0	0	0
0	0	0	0	0	0	0	0	0	0	0	0	0	0	0	0	0	0	0	0	0
0	0	0	0	0	0	0	0	0	0	0	0	0	0	0	0	0	0	0	0	0
0	0	0	0	0	0	0	0	0	0	0	0	0	0	0	0	0	0	0	0	0
0	0	0	0	0	0	0	0	0	0	0	0	0	0	0	0	0	0	0	0	0
0	0	0	0	0	0	0	0	0	0	0	0	0	0	0	0	0	0	0	0	0
0	0	0	0	0	0	0	0	0	0	0	0	0	0	0	0	0	0	0	0	0
0	0	0	0	0	0	0	0	0	0	0	0	0	0	0	0	0	0	0	0	0
0	0	0	0	0	0	0	0	0	0	0	0	0	0	0	0	0	0	0	0	0
0	0	0	0	0	0	0	0	0	0	0	0	0	0	0	0	0	0	0	0	0
0	0	0	0	0	0	0	0	0	0	0	0	0	0	0	0	0	0	0	0	0
0	0	0	0	0	0	0	0	0	0	0	0	0	0	0	0	0	0	0	0	0
0	0	0	0	0	0	0	0	0	0	0	0	0	0	0	0	0	0	0	0	0
0	0	0	0	0	0	0	0	0	0	0	0	0	0	0	0	0	0	0	0	0
0	0	0	0	0	0	0	0	0	0	0	0	0	0	0	0	0	0	0	0	0
0	0	0	0	0	0	0	0	0	0	0	0	0	0	0	0	0	0	0	0	0

0	0	0	0	0	0	0	0	0	0	0	0	0	0	0	0	0	0	0	0	0
0	0	0	0	0	0	0	0	0	0	0	0	0	0	0	0	0	0	0	0	0
0	0	0	0	0	0	0	0	0	0	0	0	0	0	0	0	0	0	0	0	0
0	0	0	0	0	0	0	0	0	0	0	0	0	0	0	0	0	0	0	0	0
0	0	0	0	0	0	0	0	0	0	0	0	0	0	0	0	0	0	0	0	0
0	0	0	0	0	0	0	0	0	0	0	0	0	0	0	0	0	0	0	0	0
0	0	0	0	0	0	0	0	0	0	0	0	0	0	0	0	0	0	0	0	0
0	0	0	0	0	0	0	0	0	0	0	0	0	0	0	0	0	0	0	0	0
0	0	0	0	0	0	0	0	0	0	0	0	0	0	0	0	0	0	0	0	0
0	0	0	0	0	0	0	0	0	0	0	0	0	0	0	0	0	0	0	0	0
0	0	0	0	0	0	0	0	0	0	0	0	0	0	0	0	0	0	0	0	0
0	0	0	0	0	0	0	0	0	0	0	0	0	0	0	0	0	0	0	0	0
0	0	0	0	0	0	0	0	0	0	0	0	0	0	0	0	0	0	0	0	0
0	0	0	0	0	0	0	0	0	0	0	0	0	0	0	0	0	0	0	0	0
0	0	0	0	0	0	0	0	0	0	0	0	0	0	0	0	0	0	0	0	0
0	0	0	0	0	0	0	0	0	0	0	0	0	0	0	0	0	0	0	0	0
0	0	0	0	0	0	0	0	0	0	0	0	0	0	0	0	0	0	0	0	0
0	0	0	0	0	0	0	0	0	0	0	0	0	0	0	0	0	0	0	0	0
0	0	0	0	0	0	0	0	0	0	0	0	0	0	0	0	0	0	0	0	0
0	0	0	0	0	0	0	0	0	0	0	0	0	0	0	0	0	0	0	0	0
0	0	0	0	0	0	0	0	0	0	0	0	0	0	0	0	0	0	0	0	0
0	0	0	0	0	0	0	0	0	0	0	0	0	0	0	0	0	0	0	0	0
0	0	0	0	0	0	0	0	0	0	0	0	0	0	0	0	0	0	0	0	0
0	0	0	0	0	0	0	0	0	0	0	0	0	0	0	0	0	0	0	0	0
0	0	0	0	0	0	0	0	0	0	0	0	0	0	0	0	0	0	0	0	0
0	0	0	0	0	0	0	0	0	0	0	0	0	0	0	0	0	0	0	0	0
0	0	0	0	0	0	0	0	0	0	0	0	0	0	0	0	0	0	0	0	0
0	0	0	0	0	0	0	0	0	0	0	0	0	0	0	0	0	0	0	0	0
0	0	0	0	0	0	0	0	0	0	0	0	0	0	0	0	0	0	0	0	0
0	0	0	0	0	0	0	0	0	0	0	0	0	0	0	0	0	0	0	0	0
0	0	0	0	0	0	0	0	0	0	0	0	0	0	0	0	0	0	0	0	0
0	0	0	0	0	0	0	0	0	0	0	0	0	0	0	0	0	0	0	0	0
0	0	0	0	0	0	0	0	0	0	0	0	0	0	0	0	0	0	0	0	0
0	0	0	0	0	0	0	0	0	0	0	0	0	0	0	0	0	0	0	0	0
0	0	0	0	0	0	0	0	0	0	0	0	0	0	0	0	0	0	0	0	0
0	0	0	0	0	0	0	0	0	0	0	0	0	0	0	0	0	0	0	0	0

0	0	0	0	0	0	0	0	0	0	0	0	0	0	0	0	0	0	0	0	0
0	0	0	0	0	0	0	0	0	0	0	0	0	0	0	0	0	0	0	0	0
0	0	0	0	0	0	0	0	0	0	0	0	0	0	0	0	0	0	0	0	0
0	0	0	0	0	0	0	0	0	0	0	0	0	0	0	0	0	0	0	0	0
0	0	0	0	0	0	0	0	0	0	0	0	0	0	0	0	0	0	0	0	0
0	0	0	0	0	0	0	0	0	0	0	0	0	0	0	0	0	0	0	0	0
0	0	0	0	0	0	0	0	0	0	0	0	0	0	0	0	0	0	0	0	0
0	0	0	0	0	0	0	0	0	0	0	0	0	0	0	0	0	0	0	0	0
0	0	0	0	0	0	0	0	0	0	0	0	0	0	0	0	0	0	0	0	0
0	0	0	0	0	0	0	0	0	0	0	0	0	0	0	0	0	0	0	0	0
0	0	0	0	0	0	0	0	0	0	0	0	0	0	0	0	0	0	0	0	0
0	0	0	0	0	0	0	0	0	0	0	0	0	0	0	0	0	0	0	0	0
0	0	0	0	0	0	0	0	0	0	0	0	0	0	0	0	0	0	0	0	0
0	0	0	0	0	0	0	0	0	0	0	0	0	0	0	0	0	0	0	0	0
0	0	0	0	0	0	0	0	0	0	0	0	0	0	0	0	0	0	0	0	0
0	0	0	0	0	0	0	0	0	0	0	0	0	0	0	0	0	0	0	0	0
0	0	0	0	0	0	0	0	0	0	0	0	0	0	0	0	0	0	0	0	0
0	0	0	0	0	0	0	0	0	0	0	0	0	0	0	0	0	0	0	0	0
0	0	0	0	0	0	0	0	0	0	0	0	0	0	0	0	0	0	0	0	0
0	0	0	0	0	0	0	0	0	0	0	0	0	0	0	0	0	0	0	0	0
0	0	0	0	0	0	0	0	0	0	0	0	0	0	0	0	0	0	0	0	0
0	0	0	0	0	0	0	0	0	0	0	0	0	0	0	0	0	0	0	0	0
0	0	0	0	0	0	0	0	0	0	0	0	0	0	0	0	0	0	0	0	0
0	0	0	0	0	0	0	0	0	0	0	0	0	0	0	0	0	0	0	0	0
0	0	0	0	0	0	0	0	0	0	0	0	0	0	0	0	0	0	0	0	0
0	0	0	0	0	0	0	0	0	0	0	0	0	0	0	0	0	0	0	0	0
0	0	0	0	0	0	0	0	0	0	0	0	0	0	0	0	0	0	0	0	0
0	0	0	0	0	0	0	0	0	0	0	0	0	0	0	0	0	0	0	0	0
0	0	0	0	0	0	0	0	0	0	0	0	0	0	0	0	0	0	0	0	0
0	0	0	0	0	0	0	0	0	0	0	0	0	0	0	0	0	0	0	0	0
0	0	0	0	0	0	0	0	0	0	0	0	0	0	0	0	0	0	0	0	0
0	0	0	0	0	0	0	0	0	0	0	0	0	0	0	0	0	0	0	0	0
0	0	0	0	0	0	0	0	0	0	0	0	0	0	0	0	0	0	0	0	0
0	0	0	0	0	0	0	0	0	0	0	0	0	0	0	0	0	0	0	0	0
0	0	0	0	0	0	0	0	0	0	0	0	0	0	0	0	0	0	0	0	0
0	0	0	0	0	0	0	0	0	0	0	0	0	0	0	0	0	0	0	0	0

0	0	0	0	0	0	0	0	0	0	0	0	0	0	0	0	0	0	0	0	0
0	0	0	0	0	0	0	0	0	0	0	0	0	0	0	0	0	0	0	0	0
0	0	0	0	0	0	0	0	0	0	0	0	0	0	0	0	0	0	0	0	0
0	0	0	0	0	0	0	0	0	0	0	0	0	0	0	0	0	0	0	0	0
0	0	0	0	0	0	0	0	0	0	0	0	0	0	0	0	0	0	0	0	0
0	0	0	0	0	0	0	0	0	0	0	0	0	0	0	0	0	0	0	0	0
0	0	0	0	0	0	0	0	0	0	0	0	0	0	0	0	0	0	0	0	0
0	0	0	0	0	0	0	0	0	0	0	0	0	0	0	0	0	0	0	0	0
0	0	0	0	0	0	0	0	0	0	0	0	0	0	0	0	0	0	0	0	0
0	0	0	0	0	0	0	0	0	0	0	0	0	0	0	0	0	0	0	0	0
0	0	0	0	0	0	0	0	0	0	0	0	0	0	0	0	0	0	0	0	0
0	0	0	0	0	0	0	0	0	0	0	0	0	0	0	0	0	0	0	0	0
0	0	0	0	0	0	0	0	0	0	0	0	0	0	0	0	0	0	0	0	0
0	0	0	0	0	0	0	0	0	0	0	0	0	0	0	0	0	0	0	0	0
0	0	0	0	0	0	0	0	0	0	0	0	0	0	0	0	0	0	0	0	0
0	0	0	0	0	0	0	0	0	0	0	0	0	0	0	0	0	0	0	0	0
0	0	0	0	0	0	0	0	0	0	0	0	0	0	0	0	0	0	0	0	0
0	0	0	0	0	0	0	0	0	0	0	0	0	0	0	0	0	0	0	0	0
0	0	0	0	0	0	0	0	0	0	0	0	0	0	0	0	0	0	0	0	0
0	0	0	0	0	0	0	0	0	0	0	0	0	0	0	0	0	0	0	0	0
0	0	0	0	0	0	0	0	0	0	0	0	0	0	0	0	0	0	0	0	0
0	0	0	0	0	0	0	0	0	0	0	0	0	0	0	0	0	0	0	0	0
0	0	0	0	0	0	0	0	0	0	0	0	0	0	0	0	0	0	0	0	0
0	0	0	0	0	0	0	0	0	0	0	0	0	0	0	0	0	0	0	0	0
0	0	0	0	0	0	0	0	0	0	0	0	0	0	0	0	0	0	0	0	0
0	0	0	0	0	0	0	0	0	0	0	0	0	0	0	0	0	0	0	0	0
0	0	0	0	0	0	0	0	0	0	0	0	0	0	0	0	0	0	0	0	0
0	0	0	0	0	0	0	0	0	0	0	0	0	0	0	0	0	0	0	0	0
0	0	0	0	0	0	0	0	0	0	0	0	0	0	0	0	0	0	0	0	0
0	0	0	0	0	0	0	0	0	0	0	0	0	0	0	0	0	0	0	0	0
0	0	0	0	0	0	0	0	0	0	0	0	0	0	0	0	0	0	0	0	0
0	0	0	0	0	0	0	0	0	0	0	0	0	0	0	0	0	0	0	0	0
0	0	0	0	0	0	0	0	0	0	0	0	0	0	0	0	0	0	0	0	0
0	0	0	0	0	0	0	0	0	0	0	0	0	0	0	0	0	0	0	0	0
0	0	0	0	0	0	0	0	0	0	0	0	0	0	0	0	0	0	0	0	0
0	0	0	0	0	0	0	0	0	0	0	0	0	0	0	0	0	0	0	0	0

0	0	0	0	0	0	0	0	0	0	0	0	0	0	0	0	0	0	0	0	0
0	0	0	0	0	0	0	0	0	0	0	0	0	0	0	0	0	0	0	0	0
0	0	0	0	0	0	0	0	0	0	0	0	0	0	0	0	0	0	0	0	0
0	0	0	0	0	0	0	0	0	0	0	0	0	0	0	0	0	0	0	0	0
0	0	0	0	0	0	0	0	0.01	0	0	0.01	0	0	0	0	0	0	0	0	0
0	0	0	0	0	0	0	0	0	0	0	0	0.01	0	0	0.01	0.02	0.14	0.03	0.01	0
0	0	0	0	0	0	0	0	0	0.01	0	0	0	0	0	0	0	0	0	0	0
0	0	0	0	0	0	0	0.03	0.01	0.03	0.01	0	0.01	0.02	0	0.01	0.01	0	0.01	0.01	0
0	0	0	0	0	0	0	0	0	0	0	0	0	0	0	0	0	0	0	0	0
0	0	0	0	0	0	0	0	0	0	0	0.03	0.01	0.01	0.01	0	0.01	0	0	0	0
0	0	0	0	0	0	0	0	0	0	0	0	0	0	0	0	0	0	0	0	0
0	0	0	0	0	0	0	0.01	0	0	0	0.01	0	0.01	0	0	0	0	0	0	0
0	0	0	0	0	0	0	0	0	0.01	0	0.01	0.01	0.01	0.01	0	0	0	0	0.01	0.01

miR_Bι	miR_Bι	miR_Cε	miR_Cε	miR_Cε	miR_Cε	miR_Cε	miR_Cε	miR_Cε	miR_Cε	miR_D(	miR_D(	miR_Dε	miR_Dl	miR_Dl	miR_Dl	miR_Dl	miR_Dl	miR_Dl	miR_Dl	miR_Ep
0.01	0.03	0.08	0.02	0.09	0.22	0.19	0.08	0.05	0.03	0.02	0.02	0.07	0.02	0.03	0.03	0.03	0.02	0	0.02	0.06
0.03	0	0.06	0.03	0.06	0.11	0.13	0.02	0.02	0.01	0.01	0.01	0.02	0.01	0.02	0.01	0.01	0.02	0	0.01	0.07
0.02	0	0.01	0.01	0.02	0.01	0.01	0.02	0.02	0.01	0	0	0	0	0	0	0	0	0	0	0.03
0	0.01	0	0	0.01	0.04	0.06	0.01	0.01	0	0	0	0	0.01	0	0	0	0	0	0	0
0	0	0.01	0	0	0.02	0.02	0	0	0.01	0	0	0.01	0	0	0	0	0	0	0	0
0	0.05	0.01	0.02	0.1	0.02	0.03	0.14	0.11	0.03	0.01	0.02	0.02	0.01	0.01	0.01	0.01	0.02	0	0.02	0.01
0	0.01	0	0	0.01	0	0	0.01	0.01	0	0	0.01	0	0	0	0	0	0	0.01	0	0.01
0	0	0.01	0.01	0.02	0	0	0.02	0.02	0.01	0.01	0.01	0	0	0	0	0	0	0	0	0.01
0	0	0	0	0	0	0	0	0	0	0	0	0	0	0	0	0	0	0	0	0
0	0	0	0	0	0	0	0	0	0	0	0	0.01	0	0	0	0	0	0	0	0
0	0	0	0	0	0	0	0	0	0	0	0	0	0	0	0	0	0	0	0	0
0	0.01	0	0	0	0	0	0	0.01	0	0	0	0	0	0	0	0	0	0	0	0
0	0	0.01	0	0	0	0	0	0	0	0	0	0	0	0	0	0	0.01	0	0	0
0	0	0	0	0	0	0.01	0	0	0	0	0	0	0	0	0	0	0	0	0	0
0	0	0	0	0	0	0	0	0	0	0	0	0	0	0	0	0	0	0	0	0
0	0	0	0	0	0	0	0	0	0	0	0	0	0	0	0	0	0	0	0	0
0	0.01	0	0.01	0.01	0.02	0	0.01	0.01	0.01	0	0	0.02	0	0	0	0	0	0.01	0	0
0	0	0	0	0	0	0	0	0	0	0	0	0	0	0	0	0	0	0	0	0
0	0	0	0	0	0	0	0	0	0	0	0	0	0	0	0	0	0	0	0	0.01
0	0	0	0	0	0	0	0	0	0	0	0	0.03	0	0	0	0	0	0	0	0.01
0.04	0	0	0	0	0	0	0	0	0	0	0	0	0	0	0	0	0	0	0	0
0	0	0.23	0	0	0	0	0	0	0	0	0	0	0	0	0	0	0	0	0	0
0	0	0	0	0	0	0	0	0	0	0	0	0	0	0	0	0	0	0	0	0
0.01	0	0.01	0	0	0	0	0	0	0	0	0	0.01	0	0	0	0	0	0	0	0.01
0.03	0	0.02	0.01	0.01	0	0	0	0.01	0.01	0	0	0.01	0	0	0	0	0	0	0	0.04
0	0	0.01	0	0.02	0	0	0.01	0	0	0	0	0.01	0.03	0.04	0.04	0	0.11	0	0	0.03
0	0	0	0	0	0	0	0	0	0	0	0	0	0	0	0	0	0	0	0	0
0	0	0	0	0	0	0	0	0	0	0	0	0	0	0	0	0	0	0	0	0
0	0	0.01	0	0	0	0	0	0	0	0	0	0	0	0	0	0	0	0	0.01	0
0	0	0.01	0	0	0	0	0	0	0	0	0	0	0	0	0	0	0	0	0.01	0
0	0	0	0	0	0	0	0	0	0	0	0	0	0	0	0	0	0	0	0	0
0	0	0.01	0	0	0	0	0	0	0	0	0	0	0	0	0	0	0	0	0	0
0	0	0	0	0	0	0	0	0	0	0	0	0	0	0	0	0	0	0	0	0
0.02	0	0	0	0	0.03	0	0	0	0	0	0	0.04	0	0	0	0	0.01	0	0	0
0	0	0	0	0	0	0	0	0	0	0	0	0	0	0	0	0	0	0	0	0

0	0	0	0	0	0	0	0	0	0	0	0	0	0	0	0	0	0	0	0	0
0	0	0	0	0	0	0	0	0	0	0	0	0	0	0	0	0	0	0	0	0
0	0	0	0	0	0	0	0	0	0	0	0	0	0	0	0	0	0	0	0	0
0	0	0	0	0	0	0	0	0	0	0	0	0	0	0	0	0	0	0	0	0
0	0	0	0	0	0	0	0	0	0	0	0	0	0	0	0	0	0	0	0	0
0	0	0	0	0	0	0	0	0	0	0	0	0	0	0	0	0	0	0	0	0
0	0	0	0	0	0	0	0	0	0	0	0	0	0	0	0	0	0	0	0	0
0	0	0	0	0	0	0	0	0	0	0	0	0	0	0	0	0	0	0	0	0
0	0	0	0	0	0	0	0	0	0	0	0	0	0	0	0	0	0	0	0	0
0	0	0	0	0	0	0	0	0	0	0	0	0	0	0	0	0	0	0	0	0
0	0	0	0	0	0	0	0	0	0	0	0	0	0	0	0	0	0	0	0	0
0	0	0	0	0	0	0.01	0	0	0	0	0	0	0	0	0	0	0	0	0	0
0.01	0.01	0	0	0.02	0	0	0.01	0.01	0	0.01	0.02	0	0.01	0.01	0.01	0.01	0	0	0.01	0
0	0	0	0	0	0	0	0	0	0	0	0	0	0.01	0	0	0	0	0	0	0.01
0.04	0.06	0	0	0	0	0	0	0	0	0.1	0.09	0	0.12	0.11	0.16	0.17	0.17	0.21	0.14	0
0.09	0.06	0	0.03	0	0	0	0	0	0.02	0.14	0.11	0	0.26	0.23	0.26	0.28	0.17	0.07	0.24	0
0.01	0	0.01	0	0	0	0	0	0	0	0	0	0	0.01	0.01	0.01	0	0.03	0	0	0.15
0	0	0	0	0	0	0	0	0	0	0	0	0	0.02	0	0	0	0.02	0	0	0
0	0	0	0	0	0	0	0	0	0	0	0	0	0	0	0	0	0	0	0	0.01
0	0.04	0	0	0	0	0	0	0	0	0.01	0	0	0	0.01	0.01	0.01	0	0.01	0.02	0
0	0	0	0	0	0	0	0	0	0	0	0	0	0	0	0	0	0	0	0	0
0	0	0	0	0	0	0	0	0	0	0	0	0	0	0	0	0	0	0	0	0
0	0	0	0	0	0	0	0	0	0	0	0	0	0	0	0	0	0	0	0	0
0	0	0	0	0	0	0	0	0	0	0	0	0	0	0	0	0	0	0	0	0
0	0	0	0	0	0	0	0	0	0	0	0	0	0	0	0	0	0	0	0	0
0	0	0	0	0	0	0	0	0	0	0	0	0	0	0	0	0	0	0	0	0
0	0	0	0	0	0	0	0	0	0	0	0	0	0	0	0	0	0	0	0	0
0	0	0	0	0	0	0	0	0	0	0	0	0	0	0	0	0	0	0	0	0
0	0	0	0	0.01	0.01	0	0	0	0	0	0	0	0	0	0	0	0	0	0	0
0	0	0	0	0.01	0	0	0	0	0	0	0	0.01	0	0	0	0	0	0	0	0
0	0	0	0	0	0	0	0	0	0	0	0	0	0	0	0	0	0	0	0	0
0	0	0	0	0	0	0	0	0	0	0	0	0	0	0	0	0	0	0	0	0
0	0	0	0	0	0	0	0	0	0	0	0	0	0	0	0	0	0	0	0	0
0	0.06	0	0	0	0	0	0	0	0	0	0	0	0.01	0.01	0.01	0.01	0.01	0.11	0	0
0.09	0.04	0.02	0.03	0.03	0.01	0.01	0.02	0.01	0.03	0.05	0.03	0.04	0.05	0.06	0.09	0.07	0.05	0.03	0.08	0
0.01	0.01	0	0	0	0.01	0.02	0.01	0	0	0	0	0.01	0	0	0	0	0	0	0	0

0.16	0.1	0.05	0.12	0.1	0.07	0.03	0.07	0.06	0.06	0.07	0.12	0.09	0.11	0.09	0.1	0.18	0.07	0.05	0.14	0.05
0.01	0.01	0	0.03	0.03	0	0	0.01	0.01	0.02	0	0	0.01	0.01	0.01	0.01	0.01	0	0.07	0.01	0
0.01	0.01	0.03	0	0	0	0	0	0	0	0.01	0	0.01	0.01	0.02	0.01	0.02	0.03	0	0.01	0
0	0	0.01	0	0	0	0	0	0	0	0	0	0.02	0	0	0	0	0	0	0	0
0	0	0	0	0	0	0	0	0	0	0	0	0	0	0	0	0	0	0	0	0
0	0	0	0	0	0	0	0	0	0	0	0	0	0	0	0	0	0	0	0	0
0	0	0	0	0	0	0	0	0	0	0	0	0	0	0	0	0	0	0	0	0
0	0	0	0	0.01	0	0	0	0	0	0	0	0	0	0	0	0	0	0	0	0
0	0	0	0	0	0	0	0	0	0	0	0	0	0	0	0	0	0	0	0	0
0	0	0	0	0	0	0	0	0	0	0	0.01	0	0	0	0	0	0	0	0	0
0	0	0	0	0.01	0	0	0.01	0	0	0	0	0.01	0	0	0	0	0	0	0.01	0
0	0	0	0	0	0	0	0	0	0	0	0	0	0	0	0	0	0	0	0	0
0	0	0	0	0	0	0	0	0	0	0	0	0	0	0	0	0	0	0	0	0
0	0	0	0	0	0	0	0	0	0	0	0	0	0	0	0	0	0	0	0	0
0	0.01	0	0	0	0	0	0.01	0.01	0.01	0	0	0.01	0	0	0	0	0	0.02	0.01	0
0	0	0	0	0	0	0	0	0	0	0	0	0	0	0	0	0	0	0	0	0
0	0	0	0	0	0	0	0	0	0	0	0	0	0	0	0	0	0	0	0	0
0	0	0	0	0	0	0	0	0	0	0	0	0	0	0	0	0	0	0	0	0
0.02	0.03	0	0	0.02	0	0.01	0	0.01	0	0.01	0.01	0	0	0.01	0	0	0.01	0	0.01	0
0	0	0	0	0	0	0	0	0	0	0	0	0	0	0	0	0	0	0	0	0
0	0	0	0	0	0	0	0	0	0	0	0	0	0	0	0	0	0	0	0	0
0	0	0	0	0	0	0	0	0	0	0	0	0	0	0	0	0	0	0	0	0
0	0.01	0	0	0	0	0	0	0	0	0	0	0	0	0	0	0	0	0	0	0
0	0	0	0.01	0	0	0	0	0	0	0	0	0	0	0	0	0	0	0	0	0
0.01	0	0.01	0	0	0	0	0	0	0	0	0	0	0	0	0	0	0	0	0	0
0	0	0	0.01	0	0.01	0	0.02	0.01	0.01	0	0	0	0	0	0	0	0	0	0	0
0	0	0	0	0	0	0	0	0	0	0	0	0	0.01	0.01	0	0.01	0.01	0	0	0
0	0	0	0	0	0.01	0	0	0	0	0	0	0	0	0	0	0	0	0	0	0
0	0	0	0	0	0	0	0	0	0	0	0	0	0	0	0	0	0	0	0	0
0	0	0	0	0	0	0	0	0	0	0	0	0	0	0	0	0	0	0	0	0
0	0	0	0	0	0	0	0	0	0	0	0	0	0	0	0	0	0	0	0	0
0	0	0	0	0	0	0	0	0	0	0	0	0	0	0	0	0	0	0	0	0
0	0	0	0	0	0	0	0	0	0	0	0	0	0	0	0	0	0	0	0	0
0	0.01	0	0	0.01	0	0	0.01	0	0	0	0	0.02	0	0	0	0	0	0	0.01	0
0	0.03	0	0	0	0.01	0	0	0.03	0.01	0	0	0.02	0.01	0.01	0	0.01	0.01	0.01	0.01	0
0	0	0	0	0	0	0	0	0	0	0	0	0	0	0	0	0	0	0	0	0

0	0	0	0	0	0	0	0	0	0	0	0	0	0	0	0	0	0	0	0	0
0	0	0	0	0	0	0	0	0	0	0	0	0	0	0	0	0	0	0	0	0.04
0	0	0	0	0	0	0	0	0	0	0	0	0	0	0	0	0	0	0	0	0
0	0	0	0	0	0	0	0	0	0	0	0	0	0	0	0	0	0	0	0	0
0	0	0.01	0	0	0	0	0	0	0	0	0	0	0	0	0	0	0	0	0	0
0	0	0	0	0	0	0	0	0	0	0	0	0	0	0	0	0	0	0	0	0.02
0	0	0	0	0	0	0	0	0	0	0	0	0	0	0	0	0	0	0	0	0
0	0	0	0	0	0	0	0	0	0	0	0	0	0	0	0	0	0	0	0	0
0	0	0	0	0	0	0	0	0	0	0	0	0	0	0	0	0	0	0	0	0
0.01	0.01	0	0.01	0.02	0	0	0.01	0.01	0.01	0	0	0.01	0	0	0	0.01	0	0.08	0	0
0	0	0	0	0	0	0	0	0	0	0	0	0	0	0	0	0	0	0	0	0
0.01	0.05	0	0.14	0.09	0.09	0.09	0.14	0.16	0.3	0.19	0.23	0.02	0.07	0.08	0.04	0.02	0.01	0.06	0.01	0.01
0	0	0	0	0	0	0	0	0	0	0	0	0	0	0	0	0	0	0	0.02	0
0	0	0	0	0	0	0	0	0	0	0	0	0	0	0	0	0	0	0	0	0
0	0	0	0	0	0	0	0	0	0	0	0	0	0	0	0	0	0	0	0	0
0	0	0	0	0	0	0	0	0	0	0	0	0.01	0	0	0	0	0	0	0	0
0	0	0	0	0	0	0	0	0	0	0	0	0	0	0	0	0	0	0	0	0
0	0	0	0	0	0	0	0	0	0	0	0	0	0	0	0	0	0	0	0	0
0	0	0	0	0	0	0	0	0	0	0	0	0	0	0	0	0	0	0	0	0
0	0	0	0	0	0	0	0	0	0	0	0	0	0	0	0	0	0	0	0	0
0	0	0	0	0	0	0	0	0	0	0	0	0	0	0	0	0	0	0	0	0
0	0	0	0	0	0	0	0	0	0	0	0	0	0	0	0	0	0	0	0	0
0	0	0	0	0	0	0	0	0	0	0	0	0	0	0	0	0	0	0	0	0
0.02	0.01	0	0	0	0	0.01	0.01	0.01	0	0.03	0.01	0.02	0.01	0.02	0.01	0	0.01	0	0	0.03
0	0	0	0	0	0	0	0	0	0	0	0	0	0	0	0	0	0	0	0	0
0	0	0	0	0	0	0	0	0	0	0	0	0	0	0	0	0	0	0	0	0
0	0	0	0	0	0	0	0	0	0	0	0	0	0	0	0	0	0	0	0	0
0	0	0	0	0	0	0	0	0	0	0	0	0	0	0	0	0	0	0	0	0
0	0	0	0	0	0	0	0	0	0	0	0	0	0	0	0	0	0	0	0	0
0	0	0	0	0	0	0	0	0	0	0.01	0.01	0	0	0	0	0	0	0	0	0
0	0	0	0	0	0	0	0	0	0	0	0	0	0	0	0	0	0	0	0	0
0.01	0	0	0.03	0.01	0.01	0.03	0.02	0.01	0.02	0.01	0.01	0.03	0	0	0	0	0	0	0	0
0.01	0	0.01	0	0.01	0.01	0.01	0	0	0	0	0	0	0	0	0	0	0	0	0	0.01
0.02	0	0.01	0.02	0.01	0.07	0.05	0.02	0.02	0.05	0.03	0.02	0.04	0.01	0.01	0	0	0.01	0	0	0.01
0.01	0.01	0	0.01	0	0	0	0.01	0	0	0	0	0.01	0	0	0.01	0	0	0	0	0
0	0.01	0.05	0	0	0.01	0.01	0.01	0.01	0.01	0.01	0	0.02	0.01	0.01	0.01	0	0.01	0.01	0.01	0.03

0	0.01	0.01	0	0.01	0	0	0.01	0.01	0	0.01	0.01	0.01	0.01	0	0.01	0	0	0.01	0.01	0.01
0.01	0	0	0.05	0.03	0.03	0.09	0.03	0.04	0.06	0.05	0.03	0.04	0.01	0.01	0.01	0	0	0	0	0.01
0.01	0	0.01	0.01	0.01	0.02	0.02	0.01	0.01	0	0	0	0	0	0	0	0	0	0	0	0.03
0	0	0	0	0	0	0	0	0	0	0	0	0	0	0	0	0	0	0	0	0
0	0	0	0	0	0	0	0	0	0	0	0	0	0	0.01	0.01	0.01	0	0	0	0
0	0	0	0	0	0	0	0	0	0	0	0	0	0	0	0	0	0	0	0	0
0	0	0	0	0	0	0	0	0	0	0	0	0	0	0	0	0	0	0	0	0
0	0	0	0	0	0	0	0	0	0	0	0	0	0	0	0	0	0	0	0	0
0	0	0	0	0	0	0	0	0	0	0	0	0	0	0	0	0	0	0	0	0
0	0	0	0	0	0	0	0	0	0	0	0	0	0	0	0	0	0	0	0	0
0	0.02	0	0.01	0	0	0	0.01	0.01	0.01	0	0.01	0.02	0	0.01	0	0	0	0.01	0.02	0.04
0.01	0.03	0	0.03	0.01	0	0	0.02	0.02	0.01	0.04	0.03	0.05	0.02	0.03	0.01	0.01	0.04	0.01	0.04	0.02
0	0	0	0	0	0	0	0	0	0	0	0	0	0	0	0	0	0	0	0.01	0
0	0	0	0	0	0	0	0	0	0	0	0	0	0	0	0	0	0	0	0	0
0	0	0	0	0	0	0	0	0	0	0	0	0	0	0	0	0	0	0	0	0
0	0	0	0	0	0	0	0	0	0	0	0	0	0	0	0	0	0	0	0	0
0	0	0	0	0	0	0	0	0	0	0	0	0	0	0	0	0	0	0	0	0
0	0	0	0	0	0	0	0	0	0	0	0	0	0	0	0	0	0	0	0	0
0	0	0	0	0	0	0	0	0	0	0	0	0	0	0	0	0	0	0	0	0
0	0	0	0	0	0	0	0	0	0	0	0	0	0	0	0	0	0	0	0	0
0	0	0.01	0.07	0.05	0	0.01	0.06	0.08	0.02	0	0	0	0	0	0	0	0	0	0	0.01
0	0	0	0	0	0	0	0	0	0	0	0.01	0	0	0	0	0	0	0.01	0	0
0	0.01	0	0.01	0	0	0.02	0	0	0	0	0.01	0	0	0	0	0	0	0	0	0
0.01	0.01	0.01	0.01	0.01	0	0	0.01	0.01	0.02	0.01	0.01	0	0.01	0.01	0.01	0.01	0.02	0.03	0.02	0
0.01	0.03	0	0.01	0.01	0	0	0.01	0.01	0	0.01	0.01	0	0.01	0.01	0.02	0.01	0.02	0	0.02	0
0	0	0	0.03	0	0.01	0	0	0.01	0.02	0	0	0	0	0	0	0	0	0	0	0
0.01	0.01	0	0.01	0.02	0.01	0	0.02	0.01	0	0.01	0.01	0.01	0.01	0.02	0.02	0.01	0.01	0.02	0.02	0
0	0	0	0	0	0	0	0.01	0.01	0.01	0	0	0.01	0	0	0	0	0	0.01	0	0
0	0	0	0	0	0	0	0	0	0	0	0	0	0	0	0	0	0	0	0	0
0	0	0	0	0	0	0	0	0	0	0	0	0	0	0	0	0	0	0	0	0
0	0	0	0	0	0	0	0	0	0	0	0	0	0	0	0	0	0	0	0	0
0	0	0	0	0	0	0	0	0	0	0	0	0	0	0	0	0	0	0	0	0
0	0	0	0	0	0	0	0	0	0	0	0	0	0	0	0	0	0	0	0	0
0	0	0	0	0	0	0	0	0	0	0	0	0	0	0	0	0	0	0	0	0
0	0	0	0	0	0	0	0	0	0	0	0	0	0	0	0	0	0	0	0	0
0	0	0	0	0	0	0	0	0	0	0	0	0	0	0	0	0	0	0	0	0

0	0	0	0	0	0	0	0	0	0	0	0	0	0	0	0	0	0	0	0	0
0	0	0	0	0	0	0	0	0	0	0	0	0	0	0	0	0	0	0	0	0
0	0	0	0	0	0	0	0	0	0	0	0	0	0	0	0	0	0	0	0	0
0	0	0	0	0	0	0	0	0	0	0	0	0	0	0	0	0	0	0	0	0
0	0	0	0	0	0	0	0	0	0	0	0	0	0	0	0	0	0	0	0	0
0	0	0	0	0	0	0	0	0	0	0	0	0	0	0	0	0	0	0	0	0
0	0	0	0	0	0	0	0	0	0	0	0	0	0	0	0	0	0	0	0	0
0	0	0	0	0	0	0	0	0	0	0	0	0	0	0	0	0	0	0	0	0
0	0	0	0	0	0	0	0	0	0.01	0	0	0	0	0	0	0	0	0	0	0
0	0	0	0	0	0	0	0	0	0	0	0	0	0	0	0	0	0	0	0	0
0	0	0	0	0	0	0	0	0	0	0	0	0	0	0	0	0	0	0	0	0
0	0	0	0	0	0	0	0	0	0	0	0	0	0	0	0	0	0	0	0	0
0	0	0	0.01	0	0	0	0	0	0	0	0	0	0	0	0	0	0	0	0	0
0	0	0	0	0	0	0	0	0	0	0	0	0	0	0	0	0	0	0	0	0
0	0	0	0	0	0	0	0	0	0	0	0	0	0	0	0	0	0	0	0	0
0	0	0	0	0	0	0	0	0	0	0	0.01	0	0	0	0	0	0	0	0	0
0	0	0	0	0	0	0	0	0	0	0	0	0	0	0	0	0	0	0	0	0
0	0	0	0	0	0	0	0	0	0	0	0	0	0	0	0	0	0	0	0	0
0	0	0	0	0	0	0	0	0	0	0	0	0	0	0	0	0	0	0	0	0
0	0	0	0	0	0	0	0	0	0	0	0	0	0	0	0	0	0	0	0	0
0	0	0	0	0	0	0	0	0	0	0	0	0	0	0	0	0	0	0	0	0
0	0	0	0	0	0	0	0	0	0	0	0	0	0	0	0	0	0	0	0	0
0	0	0	0	0	0	0	0	0	0	0	0	0	0	0	0	0	0	0	0	0
0	0	0	0	0	0	0	0	0	0	0	0	0	0	0	0	0	0	0	0	0
0	0	0	0	0	0	0	0	0	0	0	0	0	0	0	0	0	0	0	0	0
0	0	0	0	0	0	0	0	0	0	0	0	0	0	0	0	0	0	0	0	0
0	0	0	0	0	0	0	0	0	0	0	0	0	0	0	0	0	0	0	0	0
0	0	0	0	0	0	0	0	0	0	0	0	0	0	0	0	0	0	0	0	0
0	0	0	0	0	0	0	0	0	0	0	0	0	0	0	0	0	0	0	0	0
0	0	0	0	0	0	0	0	0	0	0	0	0	0	0	0	0	0	0	0	0
0	0	0	0	0	0	0	0	0	0	0	0	0	0	0	0	0	0	0	0	0
0	0	0	0	0	0	0	0	0	0	0	0	0	0	0	0	0	0	0	0	0
0	0	0	0	0	0	0	0	0	0	0	0	0	0	0	0	0	0	0	0	0
0	0	0	0	0	0	0	0	0	0	0	0	0	0	0	0	0	0	0	0	0
0	0	0	0	0	0	0	0	0	0	0	0	0	0	0	0	0	0	0	0	0
0	0	0	0	0	0	0	0	0	0	0	0	0	0	0	0	0	0	0	0	0

0	0	0	0	0	0	0	0	0	0	0	0	0	0	0	0	0	0	0	0	0
0	0	0	0	0	0	0	0	0	0	0	0	0	0	0	0	0	0	0	0	0
0	0	0	0	0.01	0	0	0.01	0	0	0	0	0.01	0	0	0.01	0	0	0	0.01	0
0	0	0	0	0	0	0	0	0	0	0	0	0.01	0	0	0	0	0	0	0	0
0	0	0	0	0	0	0	0	0	0	0	0	0	0	0	0	0	0	0	0	0
0	0	0	0	0	0	0	0	0	0	0	0	0	0	0	0	0	0	0	0	0
0	0	0	0	0	0	0	0	0	0	0	0	0	0	0	0	0	0	0	0	0
0	0	0	0	0	0	0	0	0	0	0	0	0	0	0	0	0	0	0	0	0
0	0.01	0	0	0	0	0	0	0	0	0	0	0	0	0	0	0	0	0	0	0
0	0	0	0	0	0	0	0	0	0	0	0	0	0	0	0	0	0	0	0	0
0	0	0	0	0	0	0	0	0	0	0	0	0	0	0	0	0	0	0	0	0
0	0	0	0	0	0	0	0	0	0	0	0	0	0	0	0	0	0	0	0	0
0	0	0	0	0	0	0	0	0	0	0	0	0	0	0	0	0	0	0	0	0
0	0	0	0	0	0	0	0	0	0	0	0	0	0	0	0	0	0	0	0	0
0	0	0	0	0	0	0	0	0	0	0	0	0	0	0	0	0	0	0	0	0
0	0	0	0	0	0	0	0	0	0	0	0	0	0	0	0	0	0	0	0	0
0	0	0	0	0	0	0	0	0	0	0	0	0	0	0	0	0	0	0	0	0
0	0	0	0	0	0	0	0	0	0	0	0	0	0	0	0	0	0	0	0	0
0	0	0	0	0	0	0	0	0	0	0	0	0	0	0	0	0	0	0	0	0
0	0	0	0	0	0	0	0	0	0	0	0	0	0	0	0	0	0	0	0	0
0	0	0	0	0	0	0	0	0	0	0	0	0	0	0	0	0	0	0	0	0
0	0	0	0	0	0	0	0	0	0	0	0	0	0	0	0	0	0	0	0	0
0	0	0	0	0	0	0	0	0	0	0	0	0	0	0	0	0	0	0	0	0
0	0	0	0	0	0	0	0	0	0	0.01	0	0	0	0	0	0	0.01	0	0	0
0	0	0	0	0	0	0	0	0	0	0.01	0	0	0	0	0	0	0	0	0	0
0	0	0	0	0.01	0	0	0.01	0.02	0.01	0	0	0	0	0	0	0	0	0	0	0.01
0	0.01	0	0	0	0	0	0	0	0.01	0.01	0	0	0	0	0	0	0	0.01	0	0
0	0	0	0	0	0	0	0	0	0	0	0	0	0	0	0	0	0	0	0	0
0	0	0	0	0	0	0	0	0	0	0	0	0	0	0	0	0	0	0	0	0
0	0	0	0	0	0	0	0	0	0	0	0	0	0	0	0	0	0	0	0	0
0	0	0	0	0	0	0	0	0	0	0	0	0	0	0	0	0	0	0	0	0
0	0	0	0	0	0	0	0	0	0	0	0	0	0	0	0	0	0	0	0	0
0	0	0	0	0	0	0	0	0	0	0	0	0	0	0	0	0	0	0	0	0
0	0	0	0	0	0	0	0	0	0	0	0	0	0	0	0	0	0	0	0	0
0	0	0	0	0	0	0	0	0	0	0	0	0	0	0	0	0	0	0	0	0
0	0	0	0	0	0	0	0	0	0	0	0	0	0	0	0	0	0	0	0	0

0	0	0	0	0	0	0	0	0	0	0	0	0	0	0	0	0	0	0	0	0
0	0	0	0	0	0	0	0	0	0	0	0	0	0.02	0	0	0	0.01	0	0	0
0	0	0	0	0	0	0	0	0	0	0	0	0	0	0	0	0	0	0	0	0
0	0	0	0	0	0	0	0	0	0	0	0	0	0	0	0	0	0	0	0	0
0	0	0	0	0	0	0	0	0	0	0	0	0	0	0	0	0	0	0	0	0
0	0	0	0	0	0	0	0	0	0	0	0	0	0	0	0	0	0	0	0	0
0	0	0	0	0	0	0	0	0	0	0	0	0	0	0	0	0	0	0	0	0
0	0	0	0	0	0	0	0	0	0	0	0	0	0	0	0	0	0	0	0	0
0	0	0	0	0	0	0	0	0	0	0	0	0	0	0	0	0	0	0	0	0
0	0	0	0	0	0	0	0	0	0	0	0	0	0	0	0	0	0	0	0	0
0	0	0	0	0	0	0	0	0	0	0	0	0	0	0	0	0	0	0	0	0
0	0	0	0	0	0	0	0	0	0	0	0	0	0	0	0	0	0	0	0	0
0	0	0	0	0	0	0	0	0	0	0	0	0	0	0	0	0	0	0	0	0
0	0	0	0	0	0	0	0	0	0	0	0	0	0	0	0	0	0	0	0	0
0	0	0	0	0	0	0	0	0	0	0	0	0	0	0	0	0	0	0	0	0
0	0	0	0	0	0	0	0	0	0	0	0	0	0	0	0	0	0	0	0	0
0	0	0.01	0	0	0	0	0	0	0	0	0	0	0	0	0	0	0	0	0	0
0	0	0	0	0	0	0	0	0	0	0	0	0	0	0	0	0	0	0	0	0
0	0	0	0	0	0	0	0	0	0	0	0	0	0	0	0	0	0	0	0	0
0	0	0	0	0	0	0	0	0	0	0	0	0	0	0	0	0	0	0	0	0
0	0	0	0	0	0	0	0	0	0	0	0	0	0	0	0	0	0	0	0	0
0	0	0	0	0	0	0	0	0	0	0	0	0	0	0	0	0	0	0	0	0
0	0	0	0	0	0	0	0	0	0	0	0	0	0	0	0	0	0	0	0	0
0	0	0	0	0	0	0	0	0	0	0	0	0	0	0	0	0	0	0	0	0
0	0	0	0	0	0	0	0	0	0	0	0	0	0	0	0	0	0	0	0	0
0	0	0	0	0	0	0	0	0	0	0	0	0	0	0	0	0	0	0	0	0
0.01	0	0	0	0	0	0	0	0	0	0	0	0	0	0	0	0	0	0	0	0
0	0	0	0	0	0	0	0	0	0	0	0	0	0	0	0	0	0	0	0	0
0	0	0	0	0	0	0	0	0	0	0	0	0	0	0	0	0	0	0	0	0
0	0	0	0	0	0	0	0	0	0	0	0	0	0	0	0	0	0	0	0	0
0	0	0	0	0	0	0	0	0	0	0	0	0	0	0	0	0	0	0	0	0
0	0	0	0	0	0	0	0	0	0	0	0	0	0	0	0	0	0	0	0	0
0	0	0	0	0	0	0	0	0	0	0	0	0	0	0	0	0	0	0	0	0
0	0	0	0	0	0	0	0	0	0	0	0	0	0	0	0	0	0	0	0	0
0	0	0	0	0	0	0	0	0	0	0	0	0	0	0	0	0	0	0	0	0
0	0	0	0	0	0	0	0	0	0	0	0	0	0	0	0	0	0	0	0	0

0	0	0	0	0	0	0	0	0	0	0	0	0	0	0	0	0	0	0	0	0
0	0	0	0	0	0	0	0	0	0	0	0	0	0	0	0	0	0	0	0	0
0	0	0	0	0	0	0	0	0	0	0	0	0	0	0	0	0	0	0	0	0
0	0	0	0	0	0	0	0	0	0	0	0	0	0	0	0	0	0	0	0	0
0	0	0	0	0	0	0	0	0	0	0	0	0	0	0	0	0	0	0	0	0
0	0	0	0	0	0	0	0	0	0	0	0	0	0	0	0	0	0	0	0	0
0	0	0	0	0	0	0	0	0	0	0	0	0	0	0	0	0	0	0	0	0
0	0	0	0	0	0	0	0	0	0	0	0	0	0	0	0	0	0	0	0	0
0	0	0	0	0	0	0	0	0	0	0	0	0	0	0	0	0	0	0	0	0
0	0	0	0	0	0	0	0	0	0	0	0	0	0	0	0	0	0	0	0	0
0	0	0	0	0	0	0	0	0	0	0	0	0	0	0	0	0	0	0	0	0
0	0	0	0	0	0	0	0	0	0	0	0	0	0	0	0	0	0	0	0	0
0	0	0	0	0	0	0	0	0	0	0	0	0	0	0	0	0	0	0	0	0
0	0	0	0	0	0	0	0	0	0	0	0	0	0	0	0	0	0	0	0	0
0	0	0	0	0	0	0	0	0	0	0	0	0	0	0	0	0	0	0	0	0
0	0	0	0	0	0	0	0	0	0	0	0	0	0	0	0	0	0	0	0	0
0	0	0	0	0	0	0	0	0	0	0	0	0	0	0	0	0	0	0	0	0
0	0	0	0	0	0	0	0	0	0	0	0	0	0	0	0	0	0	0	0	0
0	0	0	0	0	0	0	0	0	0	0	0	0	0	0	0	0	0	0	0	0
0	0	0	0	0	0	0	0	0	0	0	0	0	0	0	0	0	0	0	0	0
0	0	0	0	0	0	0	0	0	0	0	0	0	0	0	0	0	0	0	0	0
0	0	0	0	0	0	0	0	0	0	0	0	0	0	0	0	0	0	0	0	0
0	0	0	0	0	0	0	0	0	0	0	0	0	0	0	0	0	0	0	0	0
0	0	0	0	0	0	0	0	0	0	0	0	0	0	0	0	0	0	0	0	0
0	0	0	0	0	0	0	0	0	0	0	0	0	0	0	0	0	0	0	0	0
0	0	0	0	0	0	0	0	0	0	0	0	0	0	0	0	0	0	0	0	0
0	0	0	0	0	0	0	0	0	0	0	0	0	0	0	0	0	0	0	0	0
0	0	0	0	0	0	0	0	0	0	0	0	0	0	0	0	0	0	0	0	0
0	0	0	0	0	0	0	0	0	0	0	0	0	0	0	0	0	0	0	0	0
0	0	0	0	0	0	0	0	0	0	0	0	0	0	0	0	0	0	0	0	0
0	0	0	0	0	0	0	0	0	0	0	0	0	0	0	0	0	0	0	0	0
0	0	0	0	0	0	0	0	0	0	0	0	0	0	0	0	0	0	0	0	0
0	0	0	0	0	0	0	0	0	0	0	0	0	0	0	0	0	0	0	0	0
0	0	0	0	0	0	0	0	0	0	0	0	0	0	0	0	0	0	0	0	0
0	0	0	0	0	0	0	0	0	0	0	0	0	0	0	0	0	0	0	0	0
0	0	0	0	0	0	0	0	0	0	0	0	0	0	0	0	0	0	0	0	0

0	0	0	0	0	0	0	0	0	0	0	0	0	0	0	0	0	0	0	0	0
0	0	0	0	0	0	0	0	0	0	0	0	0	0	0	0	0	0	0	0	0
0	0	0	0	0	0	0	0	0	0	0	0	0	0	0	0	0	0	0	0	0
0	0	0	0	0	0	0	0	0	0	0	0	0	0	0	0	0	0	0	0	0
0	0	0	0	0	0	0	0	0	0	0	0	0	0	0	0	0	0	0	0	0
0	0	0	0	0	0	0	0	0	0	0	0	0	0	0	0	0	0	0	0	0
0	0	0	0	0	0	0	0	0	0	0	0	0	0	0	0	0	0	0	0	0
0	0	0	0	0	0	0	0	0	0	0	0	0	0	0	0	0	0	0	0	0
0	0	0	0	0	0	0	0	0	0	0	0	0	0	0	0	0	0	0	0	0
0	0	0	0	0	0	0	0	0	0	0	0	0	0	0	0	0	0	0	0	0
0	0	0	0	0	0	0	0	0	0	0	0	0	0	0	0	0	0	0	0	0
0	0	0	0	0	0	0	0	0	0	0	0	0	0	0	0	0	0	0	0	0
0	0	0	0	0	0	0	0	0	0	0	0	0	0	0	0	0	0	0	0	0
0	0	0	0	0	0	0	0	0	0	0	0	0	0	0	0	0	0	0	0	0
0	0	0	0	0	0	0	0	0	0	0	0	0	0	0	0	0	0	0	0	0
0	0	0	0	0	0	0	0	0	0	0	0	0	0	0	0	0	0	0	0	0
0	0	0	0	0	0	0	0	0	0	0	0	0	0	0	0	0	0	0	0	0
0	0	0	0	0	0	0	0	0	0	0	0	0	0	0	0	0	0	0	0	0
0	0	0	0	0	0	0	0	0	0	0	0	0	0	0	0	0	0	0	0	0
0	0	0	0	0	0	0	0	0	0	0	0	0	0	0	0	0	0	0	0	0
0	0	0	0	0	0	0	0	0	0	0	0	0	0	0	0	0	0	0	0	0
0	0	0	0	0	0	0	0	0	0	0	0	0	0	0	0	0	0	0	0	0
0	0	0	0	0	0	0	0	0	0	0	0	0	0	0	0	0	0	0	0	0
0	0	0	0	0.01	0	0	0	0	0	0	0	0	0	0	0	0	0	0	0	0
0	0	0	0	0	0	0	0	0	0	0	0	0	0	0	0	0	0	0	0	0
0	0	0	0	0	0	0	0	0	0	0	0	0	0	0	0	0	0	0	0	0
0	0	0	0	0	0	0	0	0	0	0	0	0	0	0	0	0	0	0	0	0
0	0	0	0	0	0	0	0	0	0	0	0	0	0	0	0	0	0	0	0	0
0	0	0	0	0	0	0	0	0	0	0	0	0	0	0	0	0	0	0	0	0
0	0	0	0	0	0	0	0	0	0	0	0	0	0	0	0	0	0	0	0	0
0	0	0	0	0	0	0	0	0	0	0	0	0	0	0	0	0	0	0	0	0
0	0	0	0	0	0	0	0	0	0	0	0	0	0	0	0	0	0	0	0	0
0	0	0	0	0	0	0	0	0	0	0	0	0	0	0	0	0	0	0	0	0
0	0	0	0	0	0	0	0	0	0	0	0	0	0	0	0	0	0	0	0	0
0	0	0	0	0	0	0	0	0	0	0	0	0	0	0	0	0	0	0	0	0
0	0	0	0	0	0	0	0	0	0	0	0	0	0	0	0	0	0	0	0	0

0	0	0	0	0	0	0	0	0	0	0	0	0	0	0	0	0	0	0	0	0
0	0	0	0	0	0	0	0	0	0	0	0	0	0	0	0	0	0	0	0	0
0	0	0	0	0	0	0	0	0	0	0	0	0	0	0	0	0	0	0	0	0
0	0	0	0	0	0	0	0	0	0	0	0	0	0	0	0	0	0	0	0	0
0	0	0	0	0	0	0	0	0	0	0	0	0	0	0	0	0	0	0	0	0
0	0	0	0	0	0	0	0	0	0	0	0	0	0	0	0	0	0	0	0	0
0	0	0	0	0	0	0	0	0	0	0	0	0	0	0	0	0	0	0	0	0
0	0	0	0	0	0	0	0	0	0	0	0	0	0	0	0	0	0	0	0	0
0	0	0	0	0	0	0	0	0	0	0	0	0	0	0	0	0	0	0	0	0
0	0	0	0	0	0	0	0	0	0	0	0	0	0	0	0	0	0	0	0	0
0	0	0	0	0	0	0	0	0	0	0	0	0	0	0	0	0	0	0	0	0
0	0	0	0	0	0	0	0	0	0	0	0	0	0	0	0	0	0	0	0	0
0	0	0	0	0	0	0	0	0	0	0	0	0	0	0	0	0	0	0	0	0
0	0	0	0	0	0	0	0	0	0	0	0	0	0	0	0	0	0	0	0	0
0	0	0	0	0	0	0	0	0	0	0	0	0	0	0	0	0	0	0	0	0
0	0	0	0	0	0	0	0	0	0	0	0	0	0	0	0	0	0	0	0	0
0	0	0	0	0	0	0	0	0	0	0	0	0	0	0	0	0	0	0	0	0
0	0	0	0	0	0	0	0	0	0	0	0	0	0	0	0	0	0	0	0	0
0	0	0	0	0	0	0	0	0	0	0	0	0	0	0	0	0	0	0	0	0
0	0	0	0	0	0	0	0	0	0	0	0	0	0	0	0	0	0	0	0	0
0	0	0	0	0	0	0	0	0	0	0	0	0	0	0	0	0	0	0	0	0
0	0	0	0	0	0	0	0	0	0	0	0	0	0	0	0	0	0	0	0	0
0	0	0	0	0	0.01	0	0	0	0	0	0	0	0	0	0	0	0	0	0	0
0	0	0	0	0	0	0	0	0	0	0	0	0	0	0	0	0	0	0	0	0
0	0	0	0	0	0	0	0	0	0	0	0	0	0	0	0	0	0	0	0	0
0	0	0	0	0	0	0	0	0	0	0	0	0	0	0	0	0	0	0	0	0
0	0	0	0	0	0	0	0	0	0	0	0	0	0	0	0	0	0	0	0	0
0	0	0	0	0	0	0	0	0	0	0	0	0	0	0	0	0	0	0	0	0
0	0	0	0	0	0	0	0	0	0	0	0	0	0	0	0	0	0	0	0	0
0	0	0	0	0	0	0	0	0	0	0	0	0	0	0	0	0	0	0	0	0
0	0	0	0	0	0	0	0	0	0	0	0	0	0	0	0	0	0	0	0	0
0	0	0	0	0	0	0	0	0	0	0	0	0	0	0	0	0	0	0	0	0
0	0	0	0	0	0	0	0	0	0	0	0	0	0	0	0	0	0	0	0	0
0	0	0	0	0	0	0	0	0	0	0	0	0	0	0	0	0	0	0	0	0
0	0	0	0	0	0	0	0	0	0	0	0	0	0	0	0	0	0	0	0	0
0	0	0	0	0	0	0	0	0	0	0	0	0	0	0	0	0	0	0	0	0

0	0	0	0	0	0	0	0	0	0	0	0	0	0	0	0	0	0	0	0	0
0	0	0	0	0	0	0	0	0	0	0	0	0	0	0	0	0	0	0	0	0
0	0	0	0	0	0	0	0	0	0	0	0	0	0	0	0	0	0	0	0	0
0	0	0	0	0	0	0	0	0	0	0	0	0	0	0	0	0	0	0	0	0
0	0	0	0	0	0	0	0	0	0	0	0	0	0	0	0	0	0	0	0	0
0	0	0	0	0	0	0	0	0	0	0	0	0	0	0	0	0	0	0	0	0
0	0	0	0	0	0	0	0	0	0	0	0	0	0	0	0	0	0	0	0	0
0	0	0	0	0	0	0	0	0	0	0	0	0	0	0	0	0	0	0	0	0
0	0	0	0	0	0	0	0	0	0	0	0	0	0	0	0	0	0	0	0	0
0	0	0	0	0	0	0	0	0	0	0	0	0	0	0	0	0	0	0	0	0
0	0	0	0	0	0	0	0	0	0	0	0	0	0	0	0	0	0	0	0	0
0	0	0	0	0	0	0	0	0	0	0	0	0	0	0	0	0	0	0	0	0
0	0	0	0	0	0	0	0	0	0	0	0	0	0	0	0	0	0	0	0	0
0	0	0	0	0	0	0	0	0	0	0	0	0	0	0	0	0	0	0	0	0
0	0	0	0	0	0	0	0	0	0	0	0	0	0	0	0	0	0	0	0	0
0	0	0	0	0	0	0	0	0	0	0	0	0	0	0	0	0	0	0	0	0
0	0	0	0	0	0	0	0	0	0	0	0	0	0	0	0	0	0	0	0	0
0	0	0	0	0	0	0	0	0	0	0	0	0	0	0	0	0	0	0	0	0
0	0	0	0	0	0	0	0	0	0	0	0	0	0	0	0	0	0	0	0	0
0	0	0	0	0	0	0	0	0	0	0	0	0	0	0	0	0	0	0	0	0
0	0	0	0	0	0	0	0	0	0	0	0	0	0	0	0	0	0	0	0	0
0	0	0	0	0	0	0	0	0	0	0	0	0	0	0	0	0	0	0	0	0
0	0	0	0	0	0	0	0	0	0	0	0	0	0	0	0	0	0	0	0	0
0	0	0	0	0	0	0	0	0	0	0	0	0	0	0	0	0	0	0	0	0
0	0	0	0	0	0	0	0	0	0	0	0	0	0	0	0	0	0	0	0	0
0	0	0	0	0	0	0	0	0	0	0	0	0	0	0	0	0	0	0	0	0
0	0	0	0	0	0	0	0	0	0	0	0	0	0	0	0	0	0	0	0	0
0	0	0	0	0	0	0	0	0	0	0	0	0	0	0	0	0	0	0	0	0
0	0	0	0	0	0	0	0	0	0	0	0	0	0	0	0	0	0	0	0	0
0	0	0	0	0	0	0	0	0	0	0	0	0	0	0	0	0	0	0	0	0
0	0	0	0	0	0	0	0	0	0	0	0	0	0	0	0	0	0	0	0	0
0	0	0	0	0	0	0	0	0	0	0	0	0	0	0	0	0	0	0	0	0
0	0	0	0	0	0	0	0	0	0	0	0	0	0	0	0	0	0	0	0	0
0	0	0	0	0	0	0	0	0	0	0	0	0	0	0	0	0	0	0	0	0
0	0	0	0	0	0	0	0	0	0	0	0	0	0	0	0	0	0	0	0	0
0	0	0	0	0	0	0	0	0	0	0	0	0	0	0	0	0	0	0	0	0

0	0	0	0	0	0	0	0	0	0	0	0	0	0	0	0	0	0	0	0	0
0	0	0	0	0	0	0	0	0	0	0	0	0	0	0	0	0	0	0	0	0
0	0	0	0	0	0	0	0	0	0	0	0	0	0	0	0	0	0	0	0	0
0	0	0	0	0	0	0	0	0	0	0	0	0	0	0	0	0	0	0	0	0
0	0	0	0	0	0	0	0	0	0	0	0	0	0	0	0	0	0	0	0	0
0	0	0	0	0	0	0	0	0	0	0	0	0	0	0	0	0	0	0	0	0
0	0	0	0	0	0	0	0	0	0	0	0	0	0	0	0	0	0	0	0	0
0	0	0	0	0	0	0	0	0	0	0	0	0	0	0	0	0	0	0	0	0
0	0	0	0	0	0	0	0	0	0	0	0	0	0	0	0	0	0	0	0	0
0	0	0	0	0	0	0	0	0	0	0	0	0	0	0	0	0	0	0	0	0
0	0	0	0	0	0	0	0	0	0	0	0	0	0	0	0	0	0	0	0	0
0	0	0	0	0	0	0	0	0	0	0	0	0	0	0	0	0	0	0	0	0
0	0	0	0	0	0	0	0	0	0	0	0	0	0	0	0	0	0	0	0	0
0	0	0	0	0	0	0	0	0	0	0	0	0	0	0	0	0	0	0	0	0
0	0	0	0	0	0	0	0	0	0	0	0	0	0	0	0	0	0	0	0	0
0	0	0	0	0	0	0	0	0	0	0	0	0	0	0	0	0	0	0	0	0
0	0	0	0	0	0	0	0	0	0	0	0	0	0	0	0	0	0	0	0	0
0	0	0	0	0	0	0	0	0	0	0	0	0	0	0	0	0	0	0	0	0
0	0	0	0	0	0	0	0	0	0	0	0	0	0	0	0	0	0	0	0	0
0	0	0	0	0	0	0	0	0	0	0	0	0	0	0	0	0	0	0	0	0
0	0	0	0	0	0	0	0	0	0	0	0	0	0	0	0	0	0	0	0	0
0	0	0	0	0	0	0	0	0	0	0	0	0	0	0	0	0	0	0	0	0
0	0	0	0	0	0	0	0	0	0	0	0	0	0	0	0	0	0	0	0	0
0	0	0	0	0	0	0	0	0	0	0	0	0	0	0	0	0	0	0	0	0
0	0	0	0	0	0	0	0	0	0	0	0	0	0	0	0	0	0	0	0	0
0	0	0	0	0	0	0	0	0	0	0	0	0	0	0	0	0	0	0	0	0
0	0	0	0	0	0	0	0	0	0	0	0	0	0	0	0	0	0	0	0	0
0	0	0	0	0	0	0	0	0	0	0	0	0	0	0	0	0	0	0	0	0
0	0	0	0	0	0	0	0	0	0	0	0	0	0	0	0	0	0	0	0	0
0	0	0	0	0	0	0	0	0	0	0	0	0	0	0	0	0	0	0	0	0
0	0	0	0	0	0	0	0	0	0	0	0	0	0	0	0	0	0	0	0	0
0	0	0	0	0	0	0	0	0	0	0	0	0	0	0	0	0	0	0	0	0
0	0	0	0	0	0	0	0	0	0	0	0	0	0	0	0	0	0	0	0	0
0	0	0	0	0	0	0	0	0	0	0	0	0	0	0	0	0	0	0	0	0
0	0	0	0	0	0	0	0	0	0	0	0	0	0	0	0	0	0	0	0	0
0	0	0	0	0	0	0	0	0	0	0	0	0	0	0	0	0	0	0	0	0

0	0	0	0	0	0	0	0	0	0	0	0	0	0	0	0	0	0	0	0	0
0	0	0	0	0	0	0	0	0	0	0	0	0	0	0	0	0	0	0	0	0
0	0	0	0	0	0	0	0	0	0	0	0	0	0	0	0	0	0	0	0	0
0	0	0	0	0	0	0	0	0	0	0	0	0	0	0	0	0	0	0	0	0
0	0	0	0	0	0	0	0	0	0	0	0	0	0	0	0	0	0	0	0	0
0	0	0	0	0	0	0	0	0	0	0	0	0	0	0	0	0	0	0	0	0
0	0	0	0	0	0	0	0	0	0	0	0	0	0	0	0	0	0	0	0	0
0	0	0	0	0	0	0	0	0	0	0	0	0	0	0	0	0	0	0	0	0
0	0	0	0	0	0	0	0	0	0	0	0	0	0	0	0	0	0	0	0	0
0	0	0	0	0	0	0.01	0	0	0	0	0	0	0	0	0	0	0	0	0	0
0	0	0	0	0	0	0	0	0	0	0	0	0	0	0	0	0	0	0	0	0
0	0	0	0	0	0	0	0	0	0	0	0	0	0	0	0	0	0	0	0	0
0	0	0	0	0	0	0	0	0	0	0	0	0	0	0	0	0	0	0	0	0
0	0	0	0	0	0	0	0.01	0	0	0	0	0	0	0	0	0	0	0	0	0
0	0	0	0	0	0	0	0	0	0	0	0	0	0	0	0	0	0	0	0	0
0	0	0	0	0	0	0	0	0	0	0	0	0	0	0	0	0	0	0	0	0
0	0	0	0	0	0	0	0	0	0	0	0	0	0	0	0	0	0	0	0	0
0	0	0	0	0	0	0	0	0	0	0	0	0	0	0	0	0	0	0	0	0
0	0	0	0	0	0	0	0	0	0	0	0	0	0	0	0	0	0	0	0	0
0	0	0	0	0	0	0	0	0	0	0	0	0	0	0	0	0	0	0	0	0
0	0	0	0	0	0	0	0	0	0	0	0	0	0	0	0	0	0	0	0	0
0	0	0	0	0	0	0	0	0	0	0	0	0	0	0	0	0	0	0	0	0
0	0	0	0	0	0	0	0	0	0	0	0	0	0	0	0	0	0	0	0	0
0	0	0	0	0	0	0	0	0	0	0	0	0	0	0	0	0	0	0	0	0
0	0	0	0	0	0	0	0	0	0	0	0	0	0	0	0	0	0	0	0	0
0	0	0	0	0	0	0	0	0	0	0	0	0	0	0	0	0	0	0	0	0
0	0	0	0	0	0	0	0	0	0	0	0	0	0	0	0	0	0	0	0	0
0	0	0	0	0	0	0	0	0	0	0	0	0	0	0	0	0	0	0	0	0
0	0	0	0	0	0	0	0	0	0	0	0	0	0	0	0	0	0	0	0	0
0	0	0	0	0	0	0	0	0	0	0	0	0	0	0	0	0	0	0	0	0
0	0	0	0	0	0	0	0	0	0	0	0	0	0	0	0	0	0	0	0	0
0	0	0	0	0	0	0	0	0	0	0	0	0	0	0	0	0	0	0	0	0
0	0	0	0	0	0	0	0	0	0	0	0	0	0	0	0	0	0	0	0	0
0	0	0	0	0	0	0	0	0	0	0	0	0	0	0	0	0	0	0	0	0.01
0	0	0	0	0	0	0	0	0	0	0	0	0	0	0	0	0	0	0	0	0
0	0	0	0	0	0	0	0	0	0	0	0	0	0	0	0	0	0	0	0	0.01

0	0	0	0	0	0	0	0	0	0	0	0	0	0	0	0	0	0	0	0	0.02
0	0	0	0	0	0	0	0	0	0	0	0	0	0	0	0	0	0	0	0	0
0	0	0	0	0	0	0	0	0	0	0	0	0	0	0	0	0	0	0	0	0
0	0	0	0	0	0	0	0	0	0	0	0	0	0	0	0	0	0	0	0	0.01
0	0	0.2	0	0	0	0	0	0	0	0	0	0.01	0	0	0	0	0	0	0	0
0.01	0.03	0	0.01	0	0	0.01	0.01	0.01	0.02	0	0.01	0.02	0.01	0	0	0.01	0	0.03	0	0
0	0	0	0	0	0.01	0	0	0	0	0	0	0	0	0	0	0	0	0	0	0
0	0	0	0.03	0.01	0	0	0	0.01	0.02	0	0	0.01	0	0	0	0	0	0	0.01	0
0	0	0	0	0	0	0	0	0	0	0	0	0	0	0	0	0	0	0	0	0
0	0	0	0	0	0.01	0	0	0	0	0	0	0	0	0	0	0	0	0	0	0
0	0	0	0	0	0	0	0.01	0.01	0	0	0	0	0	0	0	0	0	0	0	0
0	0.04	0.02	0.01	0.01	0	0	0	0.01	0.02	0	0	0	0	0	0	0	0	0	0	0.03
0.1	0	0.01	0	0.01	0	0	0	0	0	0	0	0.01	0	0	0	0	0	0	0	0.01

miR_Ev	miR_Fil	miR_Fc	miR_Fc	miR_Fr	miR_Gl	miR_Gl	miR_Gr	miR_He	miR_He	miR_He	miR_He	miR_He	miR_He	miR_Hi	miR_Hc	miR_Hc	miR_Hc	miR_Hc	miR_HS	miR_Ki
0.01	0.02	0.03	0.05	0.03	0.01	0.12	0.01	0.06	0	0	0.01	0.01	0	0	0	0.03	0.02	0.03	0.05	0
0	0.02	0.01	0.01	0.02	0.05	0.13	0	0.04	0	0	0	0	0	0.01	0	0	0.01	0	0.03	0
0	0	0	0	0.01	0	0.01	0	0.02	0.01	0	0	0	0	0	0	0	0	0	0.01	0
0	0	0	0	0	0	0	0	0	0	0	0	0	0	0	0	0.01	0	0	0.01	0
0.01	0	0	0	0	0	0	0	0	0	0	0	0	0	0	0	0	0	0	0	0
0.01	0.02	0.01	0.03	0	0	0.02	0	0.02	0	0	0	0	0	0	0.01	0.02	0.01	0	0.04	0
0	0.01	0	0.01	0	0	0	0	0.01	0	0	0	0	0	0	0.01	0.01	0	0.01	0.01	0
0	0.01	0	0.01	0	0	0.02	0	0.01	0	0	0	0	0	0	0	0	0.02	0	0	0
0	0	0	0	0	0	0	0	0.02	0	0	0	0	0	0	0	0	0	0	0	0
0	0	0	0	0	0.01	0.01	0	0	0	0	0	0	0	0	0	0	0	0	0	0
0	0	0	0	0	0	0	0	0	0	0	0	0	0	0	0	0	0	0	0	0
0	0	0	0	0	0	0	0	0	0	0	0	0	0	0	0	0	0	0	0	0
0	0	0	0	0	0	0	0.01	0	0.05	0.01	0.01	0.01	0	0	0	0	0	0	0	0
0.01	0	0	0	0	0	0	0	0	0.01	0	0.01	0	0	0	0	0	0.01	0.01	0	0
0	0	0	0	0	0	0	0	0	0	0	0	0	0	0	0	0	0	0	0	0
0	0	0	0	0	0	0	0	0	0	0	0	0	0	0	0	0	0	0	0	0
0.01	0	0	0	0	0	0	0	0	0	0.02	0.01	0.02	0.01	0	0.01	0	0.01	0.01	0	0.01
0	0	0	0	0	0	0	0	0	0.01	0	0	0	0	0	0	0	0	0	0	0
0	0	0	0	0	0	0	0	0	0	0	0	0	0	0	0	0	0	0	0	0.02
0	0	0	0	0	0	0	0	0	0	0	0	0	0	0	0	0	0	0	0	0
0	0	0	0	0	0	0	0	0	0.15	0.22	0	0	0.03	0	0	0	0	0	0	0
0	0	0	0	0.17	0	0	0	0	0	0	0	0	0	0.28	0	0	0	0	0	0
0	0	0	0	0	0	0	0	0	0	0	0	0	0	0	0	0	0	0	0	0
0.01	0.01	0	0	0	0	0	0	0	0	0	0	0	0	0	0	0	0	0	0	0
0	0.01	0	0	0.07	0.01	0.03	0	0.01	0	0	0	0	0	0.02	0	0	0	0	0	0
0	0.01	0.07	0.06	0.06	0	0	0	0.43	0	0	0	0	0.02	0.09	0	0	0	0	0.16	0
0	0	0	0	0	0	0	0	0	0	0	0	0	0	0	0	0	0	0	0	0
0	0	0	0	0	0	0	0	0	0	0	0	0	0	0	0	0	0	0	0	0
0	0	0	0	0.02	0	0	0	0	0	0.01	0	0	0	0.02	0	0	0	0	0	0
0	0	0	0	0.02	0	0	0	0	0	0	0	0	0	0.02	0	0	0	0	0	0
0	0	0	0	0	0	0	0	0	0	0	0	0	0	0	0	0	0	0	0	0
0	0	0	0	0	0	0	0	0	0	0	0	0	0	0	0	0.01	0	0	0	0
0	0	0	0	0	0	0	0	0	0	0	0	0	0	0	0	0	0	0	0	0
0.01	0.01	0	0	0	0.01	0	0	0	0.07	0.05	0.01	0	0.01	0	0	0	0	0	0.01	0
0	0	0	0	0	0	0	0	0	0	0.01	0	0	0	0	0	0	0	0	0	0

0	0	0	0	0	0	0	0	0	0	0	0	0	0	0.01	0	0	0	0	0	0
0	0	0	0	0	0	0	0	0.01	0	0	0	0	0	0	0	0	0	0	0	0
0	0	0	0	0	0	0	0	0	0	0	0	0	0	0	0	0	0	0	0	0
0	0	0	0	0	0	0	0	0	0	0	0	0	0	0	0	0	0	0	0	0
0	0	0	0	0	0	0	0	0	0	0	0	0	0	0	0	0	0	0	0	0
0	0	0	0	0	0	0	0	0	0	0	0.01	0.01	0	0	0	0	0	0	0	0
0.01	0.01	0	0	0	0	0	0	0	0	0	0	0	0	0	0	0	0	0	0	0
0	0	0	0	0	0	0	0	0	0	0	0	0	0	0	0	0	0	0	0	0
0	0	0	0	0	0	0	0	0	0	0	0	0	0	0	0	0	0	0	0	0
0	0	0	0	0	0	0	0	0	0	0	0	0	0	0	0	0	0	0	0	0
0	0	0	0	0	0	0	0	0	0	0	0	0	0	0	0	0	0	0	0	0
0	0	0	0.01	0	0	0	0	0	0	0	0	0	0	0	0	0	0	0	0	0
0	0.01	0.01	0.01	0	0.02	0.01	0.01	0	0	0.01	0.03	0.02	0.02	0.01	0.01	0.01	0.01	0.01	0.01	0.01
0	0	0	0	0	0	0.01	0	0	0	0	0	0	0	0	0	0	0	0	0	0
0	0	0.09	0.13	0	0	0	0.13	0	0	0	0	0	0	0	0.08	0.06	0.06	0.05	0.11	0
0	0	0.23	0.28	0	0	0.01	0.42	0	0	0	0	0	0	0	0.13	0.09	0.09	0.07	0.09	0
0.15	0.02	0.03	0.01	0.02	0	0.01	0.03	0.12	0	0	0	0	0	0.03	0	0	0	0	0.01	0
0	0	0	0	0	0	0	0	0	0	0	0	0	0	0	0	0	0	0	0	0
0.01	0	0	0	0	0	0	0	0	0	0	0	0	0	0	0	0	0	0	0	0
0	0.01	0.01	0.02	0	0	0	0	0	0	0.02	0.01	0	0	0	0	0.01	0	0.07	0	0
0	0	0	0	0	0	0	0	0	0	0	0	0	0	0	0	0	0	0	0	0
0	0	0	0	0	0	0	0	0	0	0	0	0	0	0	0	0	0	0	0	0
0	0	0	0	0	0	0	0	0	0	0	0	0	0	0	0	0	0	0	0	0
0	0	0	0	0	0	0	0	0	0	0	0	0	0	0	0	0	0	0	0	0
0	0	0	0	0	0	0	0	0	0	0.01	0	0	0	0	0	0	0	0	0	0
0	0	0	0	0	0	0	0	0	0	0	0	0	0	0	0	0	0	0	0	0
0	0	0	0	0	0	0	0	0	0	0	0	0	0	0	0	0	0	0	0	0
0	0	0.01	0.01	0	0	0	0	0	0	0	0	0	0	0	0	0	0	0	0	0
0	0	0	0	0	0	0	0	0	0.02	0	0	0	0	0	0	0	0	0	0	0
0.01	0	0	0	0	0	0	0	0	0	0.02	0.01	0.01	0	0	0	0	0	0	0	0
0	0.01	0	0	0	0	0	0	0	0	0	0	0	0	0	0	0	0	0	0	0
0	0	0	0	0	0	0	0	0	0	0	0	0	0	0.01	0	0	0	0	0	0
0	0	0	0	0	0	0	0	0	0	0	0	0	0	0	0	0	0	0	0	0
0	0	0	0.01	0	0	0	0	0	0	0	0	0	0	0	0.09	0.09	0.05	0.1	0.01	0
0.03	0.01	0.05	0.01	0.01	0.04	0.03	0.02	0	0.02	0.01	0.02	0.01	0.04	0.01	0.01	0.02	0.03	0.02	0.03	0.17
0	0	0	0	0	0	0.01	0	0	0.03	0	0	0	0	0	0	0.01	0	0.02	0	0.01

0.05	0.1	0.14	0.07	0.05	0.08	0.11	0.06	0.05	0.03	0.05	0.03	0.02	0.1	0.04	0.09	0.16	0.11	0.12	0.08	0.12
0.01	0	0	0	0	0	0	0	0	0	0.01	0.02	0.01	0.01	0	0	0.02	0.01	0.02	0.02	0.07
0.01	0	0	0.01	0.06	0.01	0	0	0	0	0.01	0	0	0	0.02	0	0	0	0	0.02	0
0	0	0	0	0.01	0	0	0	0	0.02	0	0	0	0	0.01	0	0	0	0	0	0
0	0	0	0	0	0	0	0	0	0	0	0	0	0	0	0	0	0	0	0	0
0	0	0	0	0	0	0	0	0	0	0	0	0	0	0	0	0	0	0	0	0
0	0	0	0	0	0	0	0	0	0	0	0	0	0	0	0	0	0	0	0	0
0	0	0	0	0	0	0	0	0	0	0	0	0	0	0	0	0	0	0	0	0
0	0	0	0	0	0	0	0	0	0	0	0	0	0	0	0	0	0	0	0	0
0	0	0	0	0	0	0	0	0	0	0	0	0	0	0	0	0	0	0	0	0.01
0	0	0	0	0	0	0	0	0	0	0.01	0	0	0.01	0	0	0.01	0.01	0	0	0.01
0	0	0	0	0	0	0	0	0	0	0	0	0	0	0	0	0	0	0	0	0
0	0	0	0	0	0	0	0	0	0	0	0	0	0	0	0	0	0	0	0	0
0	0	0	0	0	0	0	0	0	0	0	0	0	0	0	0	0	0	0	0	0
0.01	0	0	0	0	0	0	0	0	0.02	0	0.02	0.01	0.01	0	0.01	0.01	0.01	0.02	0	0.14
0	0	0	0	0	0	0	0	0	0	0	0	0	0	0	0	0	0	0	0	0
0	0	0	0	0	0	0	0	0	0	0	0	0	0	0	0	0	0	0	0	0
0	0	0	0	0	0	0	0	0	0	0	0	0	0	0	0	0	0	0	0	0
0	0.01	0.01	0	0	0	0	0	0	0	0.03	0.02	0.01	0.01	0	0	0.01	0.01	0.01	0	0.02
0	0	0	0	0	0	0	0	0	0.03	0.03	0.02	0	0.01	0	0	0	0	0	0	0
0	0	0	0	0	0	0	0	0	0	0	0	0	0.01	0	0	0	0	0	0	0
0	0	0	0	0	0	0	0	0	0	0	0	0	0	0	0	0	0	0	0	0
0	0	0	0	0	0	0	0	0	0	0	0	0	0	0	0	0	0	0	0	0
0	0	0	0	0	0	0	0	0	0.05	0.02	0.04	0.02	0.02	0	0	0	0	0	0	0
0	0	0	0	0	0	0	0	0	0	0	0	0	0	0	0.02	0	0.02	0	0	0
0	0	0	0	0	0	0	0	0	0	0	0	0	0	0	0	0	0	0	0	0.05
0	0.01	0	0	0	0	0	0	0	0	0	0	0	0	0	0	0	0	0	0.01	0.02
0	0	0	0	0	0	0	0	0	0.02	0	0	0	0	0	0	0	0	0	0	0
0	0	0	0	0	0	0	0	0	0	0	0	0	0	0	0	0	0	0	0	0
0	0	0	0	0	0	0	0	0	0	0	0	0	0	0	0	0	0	0	0	0
0	0	0	0	0	0	0	0	0	0	0	0	0	0	0	0	0	0	0	0	0
0	0	0	0	0	0	0	0	0	0	0	0	0	0	0	0	0	0	0	0	0
0	0	0	0	0	0	0	0	0	0	0	0	0	0	0	0	0	0	0	0	0
0.01	0	0	0	0	0	0	0	0	0	0.01	0.01	0	0.02	0	0	0.01	0.01	0.01	0.01	0.02
0.02	0	0	0	0	0	0	0	0	0.02	0.01	0.01	0.01	0.01	0	0.01	0.01	0.01	0.01	0.01	0.11
0	0	0	0	0	0	0	0	0	0	0	0	0	0	0	0	0	0	0	0	0

0	0	0	0	0	0	0	0	0	0	0	0	0	0	0	0	0	0	0	0	0
0	0	0	0	0	0	0	0	0	0	0	0	0	0	0	0	0	0	0	0	0
0	0	0	0	0	0	0	0	0	0	0	0	0	0	0	0	0	0	0	0	0
0	0	0	0	0	0	0	0	0	0	0	0	0	0	0	0	0	0	0	0	0
0	0	0	0	0	0	0	0	0	0	0	0	0	0	0	0	0	0	0	0	0
0	0	0	0	0	0	0	0	0	0	0	0	0	0	0	0	0	0	0	0	0
0	0	0	0	0	0	0	0	0	0	0	0	0	0	0	0	0	0	0	0	0
0	0	0	0	0	0	0	0	0	0	0	0	0	0	0	0	0	0	0	0	0
0	0	0	0	0	0	0	0	0	0	0	0	0	0	0	0	0	0	0	0	0
0.01	0	0	0	0	0	0	0	0	0.02	0.01	0.02	0.01	0.01	0	0.01	0.01	0.01	0.01	0.01	0.02
0	0	0	0	0	0	0	0	0	0	0	0	0	0	0	0	0	0	0	0	0
0.06	0.07	0.03	0.07	0	0.1	0.11	0.01	0	0.13	0.09	0.23	0.33	0.12	0	0.1	0.03	0.09	0.12	0.02	0
0	0	0	0	0	0	0.01	0	0	0	0	0	0	0	0	0	0	0.01	0.01	0	0
0	0	0	0	0	0	0	0	0	0	0	0	0	0	0	0	0	0	0	0	0
0	0	0	0	0	0	0	0	0	0	0	0	0	0	0	0	0	0	0	0	0
0	0	0	0	0	0	0	0	0	0	0	0	0	0	0	0	0	0	0	0	0
0	0	0	0	0	0	0	0	0	0.01	0	0	0	0	0	0	0	0	0	0	0
0	0	0	0	0	0	0	0	0	0	0	0	0	0	0	0	0.02	0	0	0	0
0	0	0	0	0	0	0	0	0	0	0	0	0	0	0	0	0.01	0	0	0	0
0	0	0	0	0	0	0	0	0	0	0	0	0	0	0	0	0.01	0	0	0	0
0	0	0	0	0	0	0	0	0	0	0	0	0	0	0	0	0	0	0	0	0
0	0	0	0	0	0	0	0	0	0	0	0	0	0	0	0	0	0	0	0	0
0	0	0	0	0	0	0	0	0	0	0	0	0	0	0	0	0	0	0	0	0
0.01	0.04	0	0.01	0.01	0.02	0.02	0.01	0.01	0.07	0.03	0.01	0.02	0.04	0.01	0	0	0	0	0	0
0	0	0	0	0	0	0	0	0	0	0	0	0	0	0	0	0	0	0	0	0
0	0	0	0	0	0	0	0	0	0	0	0	0	0	0	0	0	0	0	0	0
0	0	0	0	0	0	0	0	0	0	0	0	0	0	0	0	0	0	0	0	0
0	0.02	0	0	0	0	0	0	0	0	0	0	0	0	0	0	0	0	0	0	0.02
0	0.01	0	0	0	0	0	0	0	0	0	0	0	0	0	0	0	0	0	0	0.01
0	0	0	0	0	0	0	0.09	0	0	0	0	0	0	0	0	0	0	0	0.01	0
0	0	0	0	0	0	0	0	0	0	0	0	0	0	0	0	0	0	0	0	0
0	0.03	0	0	0	0.01	0.03	0.01	0	0	0	0	0	0.01	0	0	0	0	0.01	0	0
0	0	0	0	0.01	0	0	0	0.01	0	0.02	0.01	0.01	0	0	0	0	0	0	0	0
0.01	0.06	0	0	0.01	0.03	0.02	0.01	0	0.02	0.03	0.03	0.02	0.03	0.01	0.01	0.03	0.02	0.03	0.01	0.01
0	0	0	0	0	0	0	0	0	0	0.02	0	0.01	0	0	0	0.01	0	0.01	0	0
0.01	0.01	0.02	0.01	0.02	0	0.01	0.01	0.01	0.03	0.03	0.02	0.03	0.01	0.01	0	0	0	0.01	0.02	0

0.01	0.01	0.01	0	0	0	0	0.01	0	0.02	0.02	0.02	0.02	0	0	0	0	0.01	0	0.01	0
0	0.09	0.01	0.01	0	0.05	0.09	0.03	0.01	0.01	0.01	0.01	0.02	0.05	0.01	0.01	0.02	0.02	0.05	0.02	0
0.01	0.02	0	0	0.01	0.01	0	0	0.02	0.01	0.03	0.03	0.03	0.01	0.01	0	0.01	0	0	0	0
0	0	0	0	0	0	0	0	0	0	0	0	0	0	0	0	0	0	0	0	0
0	0.01	0	0.01	0	0.01	0	0	0	0	0	0.01	0.01	0.01	0	0	0	0	0	0	0
0	0	0	0	0	0	0	0	0	0	0	0	0	0	0	0	0	0	0	0	0
0	0	0	0	0	0	0	0	0	0	0	0	0	0	0	0	0	0	0	0	0
0	0	0	0	0	0	0	0	0	0	0	0	0	0	0	0	0	0	0	0	0
0	0	0	0	0	0	0	0	0	0	0	0	0	0	0	0	0	0	0	0	0
0	0	0	0	0	0	0	0	0	0	0	0	0	0	0	0	0	0	0	0	0
0	0.02	0.02	0.02	0.02	0.03	0.05	0	0.01	0	0	0.01	0.03	0.01	0.01	0.01	0.01	0.02	0	0	0
0	0.06	0.08	0.05	0.02	0.02	0.05	0.01	0	0	0	0.05	0.05	0.06	0.04	0.15	0.05	0.13	0.01	0	0.01
0	0	0.01	0.01	0	0	0	0	0.01	0	0	0	0	0	0	0	0	0	0	0	0
0	0	0	0	0	0	0	0	0	0	0	0	0	0	0	0	0	0	0	0	0
0	0	0	0	0	0	0	0	0	0	0.01	0	0	0	0	0	0	0	0	0	0
0	0	0	0	0	0	0	0	0	0	0	0	0	0	0	0	0	0	0	0	0
0	0	0	0	0	0	0	0	0	0	0	0	0	0	0	0	0	0	0	0	0
0	0	0	0	0	0	0	0	0	0	0	0	0	0	0	0	0	0	0	0	0
0	0	0	0	0	0	0	0	0	0	0	0	0	0	0	0	0	0	0	0	0
0	0	0	0	0	0	0	0	0	0	0	0	0	0	0	0	0	0	0	0	0
0.01	0.01	0	0	0	0.02	0	0	0.01	0	0	0	0	0.01	0.01	0	0	0	0.01	0	0.01
0	0	0	0	0	0	0	0	0	0	0	0	0	0	0	0	0	0	0	0	0
0	0	0.01	0	0	0	0	0	0.01	0	0	0	0	0	0	0	0	0	0	0	0
0.01	0.01	0.01	0	0	0.02	0	0	0.03	0	0.02	0.01	0.02	0.01	0.01	0.01	0.01	0.01	0	0.01	0.02
0	0.01	0.04	0.02	0	0.01	0.01	0.01	0.01	0	0.01	0.02	0.02	0.02	0.01	0.01	0.01	0.01	0.01	0.01	0.01
0	0.03	0	0	0	0	0	0	0	0	0	0	0	0	0	0	0	0	0	0	0
0.01	0.01	0.01	0	0	0.01	0	0.01	0	0.02	0.01	0.07	0.04	0.04	0	0	0.01	0	0	0	0.01
0	0	0	0	0	0	0	0	0	0	0.01	0	0	0	0	0.01	0	0	0	0.01	0
0	0	0	0	0	0	0	0	0	0	0	0	0	0	0	0	0	0	0	0	0
0	0	0	0	0	0	0	0	0	0	0	0	0	0	0	0	0	0	0	0	0
0	0	0	0	0	0	0	0	0	0	0	0	0	0	0	0	0	0	0	0	0.01
0	0	0	0	0	0	0	0	0	0	0	0	0	0	0	0.01	0	0.01	0	0	0
0	0	0	0	0	0	0	0	0	0	0	0	0	0	0	0	0	0	0	0	0
0	0	0	0	0	0	0	0	0	0	0	0	0	0	0	0	0	0	0	0	0
0	0	0	0	0	0	0	0	0	0	0	0	0	0	0	0	0	0	0	0	0
0	0	0	0	0	0	0	0	0	0	0	0	0	0	0	0	0	0	0	0	0

0	0	0	0	0	0	0	0	0	0	0	0	0	0	0	0	0	0	0	0	0
0	0	0	0	0	0	0	0	0	0	0	0	0	0	0	0	0	0	0	0	0
0	0	0	0	0	0	0	0	0	0	0	0	0	0	0	0	0	0	0	0	0
0	0	0	0	0	0	0	0	0	0	0	0	0	0	0	0	0	0	0	0	0
0	0	0	0	0	0	0	0	0	0	0	0	0	0	0	0	0	0	0	0	0
0	0	0	0	0	0	0	0	0	0	0	0	0	0	0	0	0	0	0	0	0
0	0	0	0	0	0	0	0	0	0	0	0	0	0	0	0	0	0	0	0	0
0	0	0	0	0	0	0	0	0	0	0	0	0	0	0	0	0	0	0	0	0
0	0	0	0	0	0	0	0	0	0	0	0	0	0	0	0	0	0	0	0	0
0	0	0	0	0	0	0	0	0	0	0	0	0	0	0	0	0	0	0	0	0
0	0	0	0	0	0	0	0	0	0	0	0	0	0	0	0	0	0	0	0	0
0	0	0	0	0	0	0	0	0	0	0	0	0	0	0	0	0	0	0	0	0.01
0	0	0	0	0	0	0	0	0	0	0	0	0	0	0	0	0	0	0	0	0
0	0	0	0	0	0	0	0	0	0	0	0	0	0	0	0	0	0	0	0	0.01
0	0	0	0	0	0	0	0	0	0	0	0	0	0	0	0	0	0	0	0	0
0	0	0	0	0	0	0	0	0	0	0	0	0	0	0	0	0	0	0	0	0
0	0	0	0	0	0	0	0	0	0	0	0	0	0	0	0	0	0	0	0	0
0	0	0	0	0	0	0	0	0	0	0	0	0	0	0	0	0	0	0	0	0
0	0	0	0	0	0	0	0	0	0	0	0	0	0	0	0	0	0	0	0	0
0	0	0	0	0	0	0.01	0	0	0	0	0	0	0	0	0	0	0	0	0	0
0	0	0	0	0	0	0	0	0	0	0	0	0	0	0	0	0	0	0	0	0
0	0	0	0	0	0	0	0	0	0	0	0	0	0	0	0	0	0	0	0	0
0	0	0	0	0	0	0	0	0	0	0	0	0	0	0	0	0	0	0	0	0
0	0	0	0	0	0	0	0	0	0	0	0	0	0	0	0	0	0	0	0	0
0	0	0	0	0	0	0	0	0	0	0	0	0	0	0	0	0	0	0	0	0
0	0	0	0	0	0	0	0	0	0	0	0	0	0	0	0	0	0	0	0	0
0	0	0	0	0	0	0	0	0	0	0	0	0	0	0	0	0	0	0	0	0
0	0	0	0	0	0	0	0	0	0	0	0	0	0	0	0	0	0	0	0.01	0
0	0	0	0	0	0	0	0	0	0	0	0	0	0	0	0	0	0	0	0	0
0	0	0	0	0	0	0	0	0	0	0	0	0	0	0	0	0	0	0	0	0
0	0.01	0	0	0	0	0	0	0	0	0	0	0	0	0	0	0	0	0	0	0
0	0	0	0	0	0	0	0	0	0	0	0	0	0	0	0	0	0	0	0	0
0	0	0	0	0	0	0	0	0	0	0	0	0	0	0	0	0	0	0	0	0
0	0	0	0	0	0	0	0	0	0	0	0	0	0	0	0	0	0	0	0	0
0	0	0	0	0	0	0	0	0	0	0	0	0	0	0	0	0	0	0	0	0
0	0	0	0	0	0	0	0	0	0	0	0	0	0	0	0	0	0	0	0	0

0	0	0	0	0	0	0	0	0	0	0	0	0	0	0	0	0	0	0	0	0
0	0	0	0	0	0	0	0	0	0	0	0	0	0	0	0	0	0	0	0	0
0.01	0	0	0	0	0	0	0	0	0.03	0	0.01	0	0.01	0	0	0	0	0	0	0
0.01	0	0	0	0	0.01	0	0	0	0	0	0.01	0.01	0.02	0	0	0	0	0	0	0
0	0	0	0	0	0	0	0	0	0	0	0	0	0	0	0	0	0	0	0	0
0	0	0	0	0	0	0	0	0	0	0	0	0	0	0	0	0	0	0	0	0
0	0	0	0	0	0	0	0	0	0	0	0	0	0	0	0	0	0	0	0	0
0.01	0	0	0	0	0	0	0	0	0	0	0	0	0	0	0	0	0	0	0	0
0	0	0	0	0	0	0	0	0	0	0	0	0	0	0	0	0	0	0	0	0
0	0.01	0	0	0	0	0	0	0	0	0	0	0	0	0	0	0	0	0	0	0
0	0	0	0	0	0	0	0	0	0	0	0	0	0	0	0	0	0	0	0	0
0	0	0	0	0	0	0	0	0	0	0	0	0	0	0	0	0	0	0	0	0
0	0	0	0	0	0	0	0	0	0	0	0	0	0	0	0	0	0	0	0	0
0	0	0	0	0	0	0	0	0	0	0	0	0	0	0	0	0	0	0	0	0
0	0	0	0	0	0	0	0	0	0	0	0	0	0	0	0	0	0	0	0	0
0	0	0	0	0	0	0	0	0	0	0	0	0	0	0	0	0	0	0	0	0
0	0	0	0	0	0	0	0	0	0	0	0	0	0	0	0	0	0	0	0	0
0	0	0	0	0	0	0	0	0	0	0	0	0	0	0	0	0	0	0	0	0
0	0	0	0	0	0	0	0	0	0	0	0	0	0	0	0	0	0	0	0	0
0	0	0	0	0	0	0	0	0	0	0	0	0	0	0	0	0	0	0	0	0
0	0	0	0	0	0	0	0	0	0	0	0	0	0	0	0	0	0	0	0	0
0	0	0	0	0	0	0	0	0	0	0	0	0	0	0	0	0	0	0	0	0
0	0	0	0	0	0	0	0	0	0	0	0	0	0	0	0	0	0	0	0	0
0	0	0	0	0	0	0	0	0	0	0	0	0	0	0	0	0	0	0	0	0
0	0	0	0.01	0	0	0	0	0	0	0	0	0	0	0	0	0	0	0	0	0
0.04	0	0	0	0	0.01	0	0.01	0	0.02	0	0	0	0.03	0	0	0	0	0	0.02	0
0	0	0	0	0	0	0	0	0	0	0	0.01	0.01	0.02	0	0.01	0.01	0.02	0.01	0	0
0	0	0	0	0	0	0	0	0	0	0	0	0	0	0	0	0	0	0	0	0
0	0	0	0	0	0	0	0	0	0	0	0	0	0	0	0	0	0	0	0	0
0	0	0	0	0	0	0	0	0	0	0	0	0	0	0	0	0	0	0	0	0
0	0	0	0	0	0	0	0	0	0	0	0	0	0	0	0	0	0	0	0	0
0	0	0	0	0	0	0	0	0	0	0	0	0	0	0	0	0	0	0	0	0
0	0	0	0	0	0	0	0	0	0	0	0	0	0	0	0	0	0	0	0	0
0	0	0	0	0	0	0	0	0	0	0	0	0	0	0	0	0	0	0	0	0
0	0	0	0	0	0	0	0	0	0	0	0	0	0	0	0	0	0	0	0	0
0	0	0	0	0	0	0	0	0	0	0	0	0	0	0	0	0	0	0	0	0

0	0	0	0	0	0	0	0	0	0	0	0	0	0	0	0	0	0	0	0	0
0	0	0	0	0.01	0	0	0	0.02	0	0	0	0	0	0	0	0	0	0	0	0
0	0	0	0	0	0	0	0	0	0	0	0	0	0	0	0	0	0	0	0	0
0	0	0	0	0	0	0	0	0	0	0	0	0	0	0	0	0	0	0	0	0
0	0	0	0	0	0	0	0	0	0	0	0	0	0	0	0	0	0	0	0	0
0	0	0	0	0	0	0	0	0	0	0	0.01	0	0	0	0	0	0	0	0	0
0	0	0	0	0	0	0	0	0	0	0	0	0	0	0	0	0	0	0	0	0
0.01	0	0	0	0	0	0	0	0	0	0	0	0	0	0	0	0	0	0	0	0
0.02	0	0	0	0	0	0	0	0	0	0	0	0	0	0	0	0	0	0	0	0
0	0	0	0	0	0	0	0	0	0	0	0	0	0	0	0	0	0	0	0	0
0	0	0	0	0	0	0	0	0	0	0	0	0	0	0	0	0	0	0	0	0
0	0	0	0	0	0	0	0	0	0	0	0	0	0	0	0	0	0	0	0	0
0	0	0	0	0	0	0	0	0	0	0	0	0	0	0	0	0	0	0	0	0
0	0	0	0	0	0	0	0	0	0	0	0	0	0	0	0	0	0	0	0	0
0	0	0	0	0	0	0	0	0	0	0	0	0	0	0	0	0	0	0	0	0
0	0	0	0	0	0	0	0	0	0	0	0	0	0	0	0	0	0	0	0	0
0	0	0	0	0	0	0	0	0	0	0	0	0	0	0.01	0	0	0	0	0	0
0	0	0	0	0	0	0	0	0	0	0	0	0	0	0	0	0	0	0	0	0
0	0	0	0	0	0	0	0	0	0	0	0	0	0	0	0	0	0	0	0	0
0	0	0	0	0	0	0	0	0	0	0	0	0	0	0	0	0	0	0	0	0
0	0	0	0	0	0	0	0	0	0	0	0	0	0	0	0	0	0	0	0	0
0	0	0	0	0	0	0	0	0	0	0	0	0	0	0	0	0	0	0	0	0
0	0	0	0	0	0	0	0	0	0	0	0	0	0	0	0	0	0	0	0	0
0	0	0	0	0	0	0	0	0	0	0	0	0	0	0	0	0	0	0	0	0
0	0	0	0	0	0	0	0	0	0	0	0	0	0	0	0	0	0	0	0	0
0	0	0	0	0	0	0	0	0	0	0	0	0	0	0	0	0	0	0	0	0
0	0	0	0	0	0	0	0	0	0	0	0	0	0	0	0.01	0	0	0	0	0
0	0	0	0	0	0	0	0	0	0	0	0	0	0	0	0	0	0	0	0	0
0	0	0	0	0	0	0	0	0	0	0	0	0	0	0	0	0	0	0	0	0
0	0	0	0	0	0	0	0	0	0	0	0	0	0	0	0	0	0	0	0	0
0	0	0	0	0	0	0	0	0	0	0	0	0	0	0	0	0	0	0	0	0
0	0	0	0	0	0	0	0	0	0	0	0	0	0	0	0	0	0	0	0	0
0	0	0	0	0	0	0	0	0	0	0	0	0	0	0	0	0	0	0	0	0
0	0	0	0	0	0	0	0	0	0	0	0	0	0	0	0	0	0	0	0	0
0.02	0	0	0	0	0	0.01	0	0	0	0	0	0	0	0	0	0	0	0	0	0
0	0	0	0	0	0	0	0	0	0	0	0	0	0	0	0	0	0	0	0	0

0	0	0	0	0	0	0	0	0	0	0	0	0	0	0	0	0	0	0	0	0
0	0	0	0	0	0	0	0	0	0	0	0	0	0	0	0	0	0	0	0	0
0	0	0	0	0	0	0	0	0	0	0	0	0	0	0	0	0	0	0	0	0
0	0	0	0	0	0	0	0	0	0	0	0	0	0	0	0	0	0	0	0	0
0	0	0	0	0	0	0	0	0	0	0	0	0	0	0	0	0	0	0	0	0
0	0	0	0	0	0	0	0	0	0	0	0	0	0	0	0	0	0	0	0	0
0	0	0	0	0	0	0	0	0	0	0	0	0	0	0	0	0	0	0	0	0
0	0	0	0	0	0	0	0	0	0	0	0	0	0	0	0	0	0	0	0	0
0	0	0	0	0	0	0	0	0	0	0	0	0	0	0	0	0	0	0	0	0
0	0	0	0	0	0	0	0	0	0	0	0	0	0	0	0	0	0	0	0	0
0	0	0	0	0	0	0	0	0	0	0	0	0	0	0	0	0	0	0	0	0
0	0	0	0	0	0	0	0	0	0	0	0	0	0	0	0	0	0	0	0	0
0	0	0	0	0	0	0	0	0	0	0	0	0	0	0	0	0	0	0	0	0
0	0	0	0	0	0	0	0	0	0	0	0	0	0	0	0	0	0	0	0	0
0	0	0	0	0	0	0	0	0	0	0	0	0	0	0	0	0	0	0	0	0
0	0	0	0	0	0	0	0	0	0	0	0	0	0	0	0	0	0	0	0	0
0	0	0	0	0	0	0	0	0	0	0	0	0	0	0	0	0	0	0	0	0
0	0	0	0	0	0	0	0	0	0	0	0	0	0	0	0	0	0	0	0	0
0	0	0	0	0	0	0	0	0	0	0	0	0	0	0	0	0	0	0	0	0
0	0	0	0	0	0	0	0	0	0	0	0	0	0	0	0	0	0	0	0	0
0	0	0	0	0	0	0	0	0	0	0	0	0	0	0	0	0	0	0	0	0
0	0	0	0	0	0	0	0	0	0	0	0	0	0	0	0	0	0	0	0	0
0	0	0	0	0	0	0	0	0	0	0	0	0	0	0	0	0	0	0	0	0
0	0	0	0	0	0	0	0	0	0	0	0	0	0	0	0	0	0	0	0	0
0	0	0	0	0	0	0	0	0	0	0	0	0	0	0	0	0	0	0	0	0
0	0	0	0	0	0	0	0	0	0	0	0	0	0	0	0	0	0	0	0	0
0	0	0	0	0	0	0	0	0	0	0	0	0	0	0	0	0	0	0	0	0
0	0	0	0	0	0	0	0	0	0	0	0	0	0	0	0	0	0	0	0	0
0	0	0	0	0	0	0	0	0	0	0	0	0	0	0	0	0	0	0	0	0
0	0	0	0	0	0	0	0	0	0	0	0	0	0	0	0	0	0	0	0	0
0	0	0	0	0	0	0	0	0	0	0	0	0	0	0	0	0	0	0	0	0
0	0	0	0	0	0	0	0	0	0	0	0	0	0	0	0	0	0	0	0	0
0	0	0	0	0	0	0	0	0	0	0	0	0	0	0	0	0	0	0	0	0
0	0	0	0	0	0	0	0	0	0	0	0	0	0	0	0	0	0	0	0	0
0	0	0	0	0	0	0	0	0	0	0	0	0	0	0	0	0	0	0	0	0
0	0	0	0	0	0	0	0	0	0	0	0	0	0	0	0	0	0	0	0	0

0	0	0	0	0	0	0	0	0	0	0	0	0	0	0	0	0	0	0	0	0
0	0	0	0	0	0	0	0	0	0	0	0	0	0	0	0	0	0	0	0	0
0	0	0	0	0	0	0	0	0	0	0	0	0	0	0	0	0	0	0	0	0
0	0	0	0	0	0	0	0	0	0	0	0	0	0	0	0	0	0	0	0	0
0	0	0	0	0	0	0	0	0	0	0	0	0	0	0	0	0	0	0	0	0
0	0	0	0	0	0	0	0	0	0	0	0	0	0	0	0	0	0	0	0	0
0	0	0	0	0	0	0	0	0	0	0	0	0	0	0	0	0	0	0	0	0
0	0	0	0	0	0	0	0	0	0	0	0	0	0	0	0	0	0	0	0	0
0	0	0	0	0	0	0	0	0	0	0	0	0	0	0	0	0	0	0	0	0
0	0	0	0	0	0	0	0	0	0	0	0	0	0	0	0	0	0	0	0	0
0	0	0	0	0	0	0	0	0	0	0	0	0	0	0	0	0	0	0	0	0
0	0	0	0	0	0	0	0	0	0	0	0	0	0	0	0	0	0	0	0	0
0	0	0	0	0	0	0	0	0	0	0	0	0	0	0	0	0	0	0	0	0
0	0	0	0	0	0	0	0	0	0	0	0	0	0	0	0	0	0	0	0	0
0	0	0	0	0	0	0	0	0	0	0	0	0	0	0	0	0	0	0	0	0
0	0	0	0	0	0	0	0	0	0	0	0	0	0	0	0	0	0	0	0	0
0	0	0	0	0	0	0	0	0	0.02	0	0	0	0	0	0	0	0	0	0	0
0	0	0	0	0	0	0	0	0	0	0	0	0	0	0	0	0	0	0	0	0
0	0	0	0	0	0	0	0	0	0	0	0	0	0	0	0	0	0	0	0	0
0	0	0	0	0	0	0	0	0	0	0	0	0	0	0	0	0	0	0	0	0
0	0	0	0	0	0	0	0	0	0	0	0	0	0	0	0	0	0	0	0	0
0	0	0	0	0	0	0	0	0	0	0	0	0	0	0	0	0	0	0	0	0
0	0	0	0	0	0	0	0	0	0	0	0	0	0	0	0	0	0	0	0	0
0	0	0	0	0	0	0	0	0	0	0	0	0	0	0	0	0	0	0	0	0
0	0	0	0	0	0	0	0	0	0	0	0	0	0	0	0	0	0	0	0	0
0	0	0	0	0	0	0	0	0	0	0	0	0	0	0	0	0	0	0	0	0
0	0	0	0	0	0	0	0	0	0	0	0	0	0	0	0	0	0	0	0	0
0	0	0	0	0	0	0	0	0	0	0	0	0	0	0	0	0	0	0	0	0
0	0	0	0	0	0	0	0	0	0	0	0	0	0	0	0	0	0	0	0	0
0	0	0	0	0	0	0	0	0	0	0	0	0	0	0	0	0	0	0	0	0
0	0	0	0	0	0	0	0	0	0	0	0	0	0	0	0	0	0	0	0	0
0	0	0	0	0	0	0	0	0	0	0	0	0	0	0	0	0	0	0	0	0
0	0	0	0	0	0	0	0	0	0	0	0	0	0	0	0	0	0	0	0	0
0	0	0	0	0	0	0	0	0	0	0	0	0	0	0	0	0	0	0	0	0
0	0	0	0	0	0	0	0	0	0	0	0	0	0	0	0	0	0	0	0	0
0	0	0	0	0	0	0	0	0	0	0	0	0	0	0	0	0	0	0	0	0

0	0	0	0	0	0	0	0	0	0	0	0	0	0	0	0	0	0	0	0	0
0	0	0	0	0	0	0	0	0	0	0	0	0	0	0	0	0	0	0	0	0
0	0	0	0	0	0	0	0	0	0	0	0	0	0	0	0	0	0	0	0	0
0	0	0	0	0	0	0	0	0	0	0	0	0	0	0	0	0	0	0	0	0
0	0	0	0	0	0	0	0	0	0	0	0	0	0	0	0	0	0	0	0	0
0	0	0	0	0	0	0	0	0	0	0	0	0	0	0	0	0	0	0	0	0
0	0	0	0	0	0	0	0	0	0	0	0	0	0	0	0	0	0	0	0	0
0	0	0	0	0	0	0	0	0	0	0	0	0	0	0	0	0	0	0	0	0
0	0	0	0	0	0	0	0	0	0	0	0	0	0	0	0	0	0	0	0	0
0	0	0	0	0	0	0	0	0	0	0	0	0	0	0	0	0	0	0	0	0
0	0	0	0	0	0	0	0	0	0	0	0	0	0	0	0	0	0	0	0	0
0	0	0	0	0	0	0	0	0	0	0	0	0	0	0	0	0	0	0	0	0
0	0	0	0	0	0	0	0	0	0	0	0	0	0	0	0	0	0	0	0	0
0	0	0	0	0	0	0	0	0	0	0	0	0	0	0	0	0	0	0	0	0
0	0	0	0	0	0	0	0	0	0	0	0	0	0	0	0	0	0	0	0	0
0	0	0	0	0	0	0	0	0	0	0	0	0	0	0	0	0	0	0	0	0
0	0	0	0	0	0	0	0	0	0	0	0	0	0	0	0	0	0	0	0	0
0	0	0	0	0	0	0	0	0	0	0	0	0	0	0	0	0	0	0	0	0
0	0	0	0	0	0	0	0	0	0	0	0	0	0	0	0	0	0	0	0	0
0	0	0	0	0	0	0	0	0	0	0	0	0	0	0	0	0	0	0	0	0
0	0	0	0	0	0	0	0	0	0	0	0	0	0	0	0	0	0	0	0	0
0	0	0	0	0	0	0	0	0	0	0	0	0	0	0	0	0	0	0	0	0
0	0	0	0	0	0	0	0	0	0	0	0	0	0	0	0	0	0	0	0	0
0	0	0	0	0	0	0	0	0	0	0	0	0	0	0	0	0	0	0	0	0
0	0	0	0	0	0	0	0	0	0	0	0	0	0	0	0	0	0	0	0	0
0	0	0	0	0	0	0	0	0	0	0	0	0	0	0	0	0	0	0	0	0
0	0	0	0	0	0	0	0	0	0	0	0	0	0	0	0	0	0	0	0	0
0	0	0	0	0	0	0	0	0	0	0	0	0	0	0	0	0	0	0	0	0
0	0	0	0	0	0	0	0	0	0	0	0	0	0	0	0	0	0	0	0	0
0	0	0	0	0	0	0	0	0	0	0	0	0	0	0	0	0	0	0	0	0
0	0	0	0	0	0	0	0	0	0	0	0	0	0	0	0	0	0	0	0	0
0	0	0	0	0	0	0	0	0	0	0	0	0	0	0	0	0	0	0	0	0
0	0	0	0	0	0	0	0	0	0	0	0	0	0	0	0	0	0	0	0	0
0	0	0	0	0	0	0	0	0	0	0	0	0	0	0	0	0	0	0	0	0
0	0	0	0	0	0	0	0	0	0	0	0	0	0	0	0	0	0	0	0	0
0	0	0	0	0	0	0	0	0	0	0	0	0	0	0	0	0	0	0	0	0
0	0	0	0	0	0	0	0	0	0	0	0	0	0	0	0	0	0	0	0	0

0	0	0	0	0	0	0	0	0	0	0	0	0	0	0	0	0	0	0	0	0
0	0	0	0	0	0	0	0	0	0	0	0	0	0	0	0	0	0	0	0	0
0	0	0	0	0	0	0	0	0	0	0	0	0	0	0	0	0	0	0	0	0
0	0	0	0	0	0	0	0	0	0	0	0	0	0	0	0	0	0	0	0	0
0	0	0	0	0	0	0	0	0	0	0	0	0	0	0	0	0	0	0	0	0
0	0	0	0	0	0	0	0	0	0	0	0	0	0	0	0	0	0	0	0	0
0	0	0	0	0	0	0	0	0	0	0	0	0	0	0	0	0	0	0	0	0
0	0	0	0	0	0	0	0	0	0	0	0	0	0	0	0	0	0	0	0	0
0	0	0	0	0	0	0	0	0	0	0	0	0	0	0	0	0	0	0	0	0
0	0	0	0	0	0	0	0	0	0	0	0	0	0	0	0	0	0	0	0	0
0	0	0	0	0	0	0	0	0	0	0	0	0	0	0	0	0	0	0	0	0
0	0	0	0	0	0	0	0	0	0	0	0	0	0	0	0	0	0	0	0	0
0	0	0	0	0	0	0	0	0	0	0	0	0	0	0	0	0	0	0	0	0
0	0	0	0	0	0	0	0	0	0	0	0	0	0	0	0	0	0	0	0	0
0	0	0	0	0	0	0	0	0	0	0	0	0	0	0	0	0	0	0	0	0
0	0	0	0	0	0	0	0	0	0	0	0	0	0	0	0	0	0	0	0	0
0	0	0	0	0	0	0	0	0	0	0	0	0	0	0	0	0	0	0	0	0
0	0	0	0	0	0	0	0	0	0	0	0	0	0	0	0	0	0	0	0	0
0	0	0	0	0	0	0	0	0	0	0	0	0	0	0	0	0	0	0	0	0
0	0	0	0	0	0	0	0	0	0	0	0	0	0	0	0	0	0	0	0	0
0	0	0	0	0	0	0	0	0	0	0	0	0	0	0	0	0	0	0	0	0
0	0	0	0	0	0	0	0	0	0	0	0	0	0	0	0	0	0	0	0	0
0	0	0	0	0	0	0	0	0	0	0	0	0	0	0	0	0	0	0	0	0
0	0	0	0	0	0	0	0	0	0	0	0	0	0	0	0	0	0	0	0	0
0	0	0	0	0	0	0	0	0	0	0	0	0	0	0	0	0	0	0	0	0
0	0	0	0	0	0	0	0	0	0	0	0	0	0	0	0	0	0	0	0	0
0	0	0	0	0	0	0	0	0	0	0	0	0	0	0	0	0	0	0	0	0
0	0	0	0	0	0	0	0	0	0	0	0	0	0	0	0	0	0	0	0	0
0	0	0	0	0	0	0	0	0	0	0	0	0	0	0	0	0	0	0	0	0
0	0	0	0	0	0	0	0	0	0	0	0	0	0	0	0	0	0	0	0	0.01
0	0	0	0	0	0	0	0	0	0	0	0	0	0	0	0	0	0	0	0	0
0	0	0	0	0	0	0	0	0	0	0	0	0	0	0	0	0	0	0	0	0
0	0	0	0	0	0	0	0	0	0	0	0	0	0	0	0	0	0	0	0	0
0	0	0	0	0	0	0	0	0	0	0	0	0	0	0	0	0	0	0	0	0
0	0	0	0	0	0	0	0	0	0	0	0	0	0	0	0	0	0	0	0	0
0	0	0	0	0	0	0	0	0	0	0	0	0	0	0	0	0	0	0	0	0

0	0	0	0	0	0	0	0	0	0	0	0	0	0	0	0	0	0	0	0	0
0	0	0	0	0	0	0	0	0	0	0	0	0	0	0	0	0	0	0	0	0
0	0	0	0	0	0	0	0	0	0	0	0	0	0	0	0	0	0	0	0	0
0	0	0	0	0	0	0	0	0	0	0	0	0	0	0	0	0	0	0	0	0
0	0	0	0	0	0	0	0	0	0	0	0	0	0	0	0	0	0	0	0	0
0	0	0	0	0	0	0	0	0	0	0	0	0	0	0	0	0	0	0	0	0
0	0	0	0	0	0	0	0	0	0	0	0	0	0	0	0	0	0	0	0	0
0	0	0	0	0	0	0	0	0	0	0	0	0	0	0	0	0	0	0	0	0
0	0	0	0	0	0	0	0	0	0	0	0	0	0	0	0	0	0	0	0	0
0	0	0	0	0	0	0	0	0	0	0	0	0	0	0	0	0	0	0	0	0
0	0	0	0	0	0	0	0	0	0	0	0	0	0	0	0	0	0	0	0	0
0	0	0	0	0	0	0	0	0	0	0	0	0	0	0	0	0	0	0	0	0
0	0	0	0	0	0	0	0	0	0	0	0	0	0	0	0	0	0	0	0	0
0	0	0	0	0	0	0	0	0	0	0	0	0	0	0	0	0	0	0	0	0
0	0	0	0	0	0	0	0	0	0	0	0	0	0	0	0	0	0	0	0	0
0	0	0	0	0	0	0	0	0	0	0	0	0	0	0	0	0	0	0	0	0
0	0	0	0	0	0	0	0	0	0	0	0	0	0	0	0	0	0	0	0	0
0	0	0	0	0	0	0	0	0	0	0	0	0	0	0	0	0	0	0	0	0
0	0	0	0	0	0	0	0	0	0	0	0	0	0	0	0	0	0	0	0	0
0	0	0	0	0	0	0	0	0	0	0	0	0	0	0	0	0	0	0	0	0
0	0	0	0	0	0	0	0	0	0	0	0	0	0	0	0	0	0	0	0	0
0	0	0	0	0	0	0	0	0	0	0	0	0	0	0	0	0	0	0	0	0
0	0	0	0	0	0	0	0	0	0	0	0	0	0	0	0	0	0	0	0	0
0	0	0	0	0	0	0	0	0	0	0	0	0	0	0	0	0	0	0	0	0
0	0	0	0	0	0	0	0	0	0	0	0	0	0	0	0	0	0	0	0	0
0	0	0	0	0	0	0	0	0	0	0	0	0	0	0	0	0	0	0	0	0
0	0	0	0	0	0	0	0	0	0	0	0	0	0	0	0	0	0	0	0	0
0	0	0	0	0	0	0	0	0	0	0	0	0	0	0	0	0	0	0	0	0
0	0	0	0	0	0	0	0	0	0	0	0	0	0	0	0	0	0	0	0	0
0	0	0	0	0	0	0	0	0	0	0	0	0	0	0	0	0	0	0	0	0
0	0	0	0	0	0	0	0	0	0	0	0	0	0	0	0	0	0	0	0	0
0	0	0	0	0	0	0	0	0	0	0	0	0	0	0	0	0	0	0	0	0
0	0	0	0	0	0	0	0	0	0	0	0	0	0	0	0	0	0	0	0	0
0	0	0	0	0	0	0	0	0	0	0	0	0	0	0	0	0	0	0	0	0
0	0	0	0	0	0	0	0	0	0	0	0	0	0	0	0	0	0	0	0	0
0	0	0	0	0	0	0	0	0	0	0	0	0	0	0	0	0	0	0	0	0

0	0	0	0	0	0	0	0	0	0	0	0	0	0	0	0	0	0	0	0	0
0	0	0	0	0	0	0	0	0	0	0	0	0	0	0	0	0	0	0	0	0
0	0	0	0	0	0	0	0	0	0	0	0	0	0	0	0	0	0	0	0	0
0	0	0	0	0	0	0	0	0	0	0	0	0	0	0	0	0	0	0	0	0
0	0	0	0	0	0	0	0	0	0	0	0	0	0	0	0	0	0	0	0	0
0	0	0	0	0	0	0	0	0	0	0	0	0	0	0	0	0	0	0	0	0
0	0	0	0	0	0	0	0	0	0	0	0	0	0	0	0	0	0	0	0	0
0	0	0	0	0	0	0	0	0	0	0	0	0	0	0	0	0	0	0	0	0
0	0	0	0	0	0	0	0	0	0	0	0	0	0	0	0	0	0	0	0	0
0	0	0	0	0	0	0	0	0	0	0	0	0	0	0	0	0	0	0	0	0
0	0	0	0	0	0	0	0	0	0	0	0	0	0	0	0	0	0	0	0	0
0	0	0	0	0	0	0	0	0	0	0	0	0	0	0	0	0	0	0	0	0
0	0	0	0	0	0	0	0	0	0	0	0	0	0	0	0	0	0	0	0	0
0	0	0	0	0	0	0	0	0	0	0	0	0	0	0	0	0	0	0	0	0
0	0	0	0	0	0	0.01	0	0	0	0	0	0	0	0	0	0	0	0	0	0
0	0.01	0	0	0	0	0	0	0	0	0.01	0	0	0	0	0	0	0	0	0	0
0	0	0	0	0	0	0	0	0	0	0	0	0	0	0	0	0	0	0	0	0
0	0	0	0	0	0	0	0	0	0	0	0	0	0	0	0	0	0	0	0	0
0	0	0	0	0	0	0	0	0	0	0	0	0	0	0	0	0	0	0	0	0
0	0	0	0	0	0	0	0	0	0	0	0	0	0	0	0	0	0	0	0	0
0	0	0	0	0	0	0	0	0	0	0	0	0	0	0	0	0	0	0	0	0
0	0	0	0	0	0	0	0	0	0	0	0	0	0	0	0	0	0	0	0	0
0	0	0	0	0	0	0	0	0	0	0	0	0	0	0	0	0	0	0	0	0
0	0	0	0	0	0	0	0	0	0	0	0	0	0	0	0	0	0	0	0	0
0	0	0	0	0	0	0	0	0	0	0	0	0	0	0	0	0	0	0	0	0
0	0	0	0	0	0	0	0	0	0	0	0	0	0	0	0	0	0	0	0	0
0	0	0	0	0	0	0	0	0	0	0	0	0	0	0	0	0	0	0	0	0
0	0	0	0	0	0	0	0	0	0	0	0	0	0	0	0	0	0	0	0	0
0	0	0	0	0	0	0	0	0	0	0	0	0	0	0	0	0	0	0	0	0
0	0	0	0	0	0	0	0	0	0	0	0	0	0	0	0	0	0	0	0	0
0	0	0	0	0	0	0	0	0	0	0	0	0	0	0	0	0	0	0	0	0
0	0	0	0	0	0	0	0	0	0	0	0	0	0	0	0	0	0	0	0	0
0	0	0	0	0	0	0	0	0	0	0	0	0	0	0	0	0	0	0	0	0
0	0	0	0	0	0	0	0	0	0	0	0	0	0	0	0	0	0	0	0	0
0	0	0	0	0	0	0	0	0	0	0	0	0	0	0	0	0	0	0	0	0
0	0	0	0	0	0	0	0	0	0	0	0	0	0	0	0	0	0	0	0	0

0	0	0	0	0	0	0	0	0	0	0	0	0	0	0	0	0	0	0	0	0
0	0	0	0	0	0	0	0	0	0	0	0	0	0	0	0	0	0	0	0	0
0	0	0	0	0	0	0	0	0	0	0	0	0	0	0	0	0	0	0	0	0
0	0	0	0	0	0	0	0	0	0	0	0	0	0	0	0	0	0	0	0	0
0.14	0	0	0	0.2	0.26	0.01	0	0	0	0	0	0	0.01	0.15	0.03	0.03	0.04	0	0	0
0.01	0	0	0	0	0	0.01	0	0	0.03	0.02	0.01	0.01	0.01	0	0.01	0.04	0.01	0.02	0.03	0.02
0	0	0	0	0	0	0	0	0	0	0	0	0	0	0	0	0	0	0	0	0
0.01	0	0	0	0	0	0	0	0	0	0	0.01	0.02	0.01	0	0.02	0.01	0.01	0.01	0	0.02
0	0	0	0	0	0	0	0	0	0	0	0	0	0	0	0	0	0	0	0	0
0	0	0	0	0	0	0	0	0	0	0	0	0.01	0.01	0	0	0	0	0	0	0
0	0	0	0	0	0	0	0	0	0.01	0	0	0	0	0	0	0	0	0	0	0
0	0	0	0	0.03	0.03	0	0	0	0	0	0	0	0	0.01	0	0	0	0	0	0.02
0.04	0	0	0	0.01	0.01	0	0	0	0	0	0.01	0	0.01	0.01	0	0	0	0.01	0	0

miR_Ki	miR_Ki	miR_Ki	miR_Li	miR_Lu	miR_Lu	miR_Lu	miR_Lu	miR_M	miR_M	miR_M	miR_M	miR_M	miR_M	miR_Mi	miR_M	miR_M	miR_Ne	miR_Ne	miR_Ne	miR_Ne
0	0.01	0.01	0.02	0.05	0.06	0.04	0.08	0.01	0.02	0.01	0.06	0.05	0.03	0.04	0.02	0.02	0	0	0.09	0
0	0	0	0.01	0.02	0.01	0.01	0.05	0.01	0	0	0.03	0.03	0.02	0.05	0.01	0.02	0	0.01	0.04	0.02
0	0	0	0.01	0	0	0	0	0	0	0.01	0	0	0	0.01	0	0	0	0	0.01	0
0	0	0	0	0.01	0	0	0.01	0	0	0	0	0	0	0	0	0	0	0	0	0
0	0	0	0	0.01	0.01	0.01	0.02	0	0	0	0	0	0.01	0.01	0	0	0	0	0	0
0.01	0	0.01	0	0.04	0.04	0.04	0.08	0.05	0.04	0.05	0.02	0.01	0.01	0	0.01	0.01	0	0	0.04	0
0	0	0	0	0	0	0	0	0	0.01	0	0.01	0.01	0	0	0.01	0	0	0	0.01	0
0	0	0	0	0.03	0.03	0.04	0.03	0	0	0.01	0.01	0.01	0.02	0	0	0.01	0	0	0.01	0
0	0	0	0	0	0	0	0	0	0	0	0	0	0	0	0	0	0	0	0	0
0	0	0	0	0	0	0	0	0	0	0	0	0	0.01	0.01	0	0	0	0	0	0
0	0	0	0	0	0	0	0	0	0	0	0	0	0	0	0	0	0	0	0	0
0	0	0	0	0	0	0	0	0	0	0	0	0	0	0	0	0	0	0	0	0
0	0.01	0	0	0	0	0	0	0.01	0	0	0.01	0.01	0	0	0	0.01	0	0.01	0.01	0.01
0.01	0	0	0	0	0	0	0	0	0	0	0	0	0	0	0.01	0	0	0	0.01	0
0	0	0	0	0	0	0	0	0	0	0	0	0	0	0	0	0	0	0	0	0
0	0	0	0	0	0	0	0	0	0	0	0	0	0	0	0	0	0	0	0.01	0
0.01	0.01	0.02	0	0	0	0	0	0	0	0	0	0	0	0	0	0	0.01	0	0.03	0.01
0	0	0	0	0	0	0	0	0	0	0	0	0	0	0	0	0	0	0	0	0
0.02	0.03	0.03	0	0	0	0	0	0	0	0	0	0	0	0	0	0	0	0	0	0
0.01	0	0.01	0	0	0	0	0	0	0	0	0	0	0	0	0	0	0	0	0.02	0.02
0	0	0	0.72	0	0	0	0	0	0	0	0	0	0.01	0	0	0	0	0	0	0
0	0	0	0	0	0	0	0	0	0	0	0	0	0.01	0.1	0	0	0.09	0.19	0.04	0.14
0	0	0	0	0	0	0	0	0	0	0	0	0	0	0	0	0	0	0	0	0
0	0	0	0	0	0	0	0	0	0	0	0	0	0	0.01	0	0	0	0	0	0.01
0	0	0	0.01	0	0	0	0	0	0	0	0	0	0.02	0.06	0	0	0.01	0.01	0.01	0.01
0	0.01	0	0.06	0	0	0.01	0.01	0	0	0	0.03	0.06	0.01	0.06	0	0.04	0	0.01	0	0.03
0	0	0	0	0	0	0	0	0	0	0	0	0	0	0	0	0	0	0	0	0
0	0	0	0	0	0	0	0	0	0	0	0	0	0	0	0	0	0	0	0	0
0	0	0	0	0	0	0	0	0	0	0	0	0	0	0.01	0	0	0	0	0	0
0	0	0	0	0	0	0	0	0	0	0	0	0	0	0.01	0	0	0	0	0	0
0	0	0	0	0	0	0	0	0	0	0	0	0	0	0	0	0	0	0	0	0
0	0	0	0	0	0	0	0	0	0	0	0	0	0	0	0	0	0	0	0	0
0	0	0	0	0	0	0	0	0	0	0	0	0	0	0	0	0	0	0	0	0
0	0	0	0	0	0.01	0.01	0.01	0	0	0	0	0	0.02	0	0	0	0.02	0	0.01	0.02
0	0	0	0	0	0	0	0	0	0	0	0	0	0	0	0	0	0	0	0	0.01

0	0	0	0	0	0	0	0	0	0	0	0	0	0	0	0	0	0	0	0	0
0	0	0	0	0	0	0	0	0	0	0	0	0	0	0	0	0	0	0	0	0
0	0	0	0	0	0	0	0	0	0	0	0	0	0	0	0	0	0	0	0	0
0	0	0	0	0	0	0	0	0	0	0	0	0	0	0	0	0	0.01	0	0	0
0	0	0	0	0	0	0	0	0	0	0	0	0	0	0	0	0	0	0	0	0
0	0	0	0	0	0	0	0	0	0	0	0	0	0	0	0	0	0	0	0	0
0	0	0	0	0	0	0	0	0	0	0	0	0	0.01	0	0	0	0.05	0	0	0
0	0	0	0	0	0	0	0	0	0	0	0	0	0	0	0	0	0	0.02	0.02	0.02
0	0	0	0	0	0	0	0	0	0	0	0	0	0	0	0	0	0	0.01	0.01	0.01
0	0	0	0	0	0	0	0	0	0	0	0	0	0	0	0	0	0	0	0	0
0	0	0	0	0	0	0	0	0	0	0	0	0	0	0	0	0	0	0	0	0
0	0	0	0	0	0	0	0	0	0	0	0.01	0	0	0	0	0.01	0	0	0	0
0	0.01	0.01	0	0.02	0.01	0.02	0.01	0.01	0.01	0.01	0.01	0.01	0.01	0	0.01	0.01	0.01	0.01	0.01	0.02
0	0	0	0	0	0	0	0	0	0	0	0	0.01	0	0	0	0	0	0	0	0
0	0.01	0	0	0	0	0	0	0.17	0.25	0.19	0.1	0.13	0	0	0.11	0.1	0	0	0	0
0.01	0.07	0	0	0	0	0	0	0.08	0.06	0.07	0.29	0.21	0	0	0.16	0.3	0	0	0	0
0	0	0	0.01	0	0	0	0	0	0	0	0.02	0.02	0.03	0.03	0	0.01	0.02	0.03	0.01	0.02
0	0	0	0	0	0	0	0	0	0	0	0	0	0	0	0	0	0	0	0	0
0	0	0	0	0	0	0	0	0	0	0	0	0	0	0	0	0	0	0	0	0
0	0	0	0	0	0	0	0	0.05	0	0	0.02	0.01	0.03	0	0	0	0	0	0	0
0	0	0	0	0	0	0	0	0	0	0	0	0	0	0	0	0	0	0	0	0
0	0	0	0	0	0	0	0	0	0	0	0	0	0	0	0	0	0	0	0	0
0	0	0	0	0	0	0	0	0	0	0	0	0	0	0	0	0	0	0	0	0
0	0	0	0	0	0	0	0	0	0	0	0	0	0	0	0	0	0	0	0	0
0	0	0	0	0	0	0	0	0	0	0	0	0	0	0	0	0	0	0	0	0
0	0	0	0	0	0	0	0	0	0	0	0	0	0	0	0	0	0	0	0	0
0	0	0	0	0	0	0	0	0	0	0	0	0	0	0	0	0	0	0	0	0
0	0	0	0	0	0	0	0	0	0	0	0.01	0.02	0	0	0	0.01	0	0	0	0
0	0	0	0	0	0	0	0	0	0	0	0	0	0	0	0	0	0	0	0	0
0.01	0	0.01	0	0.01	0	0.01	0	0	0	0	0	0	0	0	0	0	0	0	0	0
0	0	0	0	0	0	0	0	0	0	0	0	0	0	0	0	0	0	0	0	0
0	0	0	0	0	0	0	0	0	0	0	0	0	0	0	0	0	0	0	0	0
0	0	0	0	0	0	0	0	0	0	0	0	0	0	0	0	0	0	0	0	0
0	0	0	0	0	0	0	0	0.07	0	0.16	0.01	0	0.01	0	0	0.01	0	0	0	0
0.05	0.08	0.04	0	0.01	0.01	0.02	0.02	0.02	0.03	0.03	0.03	0.02	0.02	0.01	0.1	0.02	0.05	0.01	0.03	0.03
0.01	0.02	0.01	0	0.01	0.01	0	0.01	0.01	0	0.01	0	0	0.01	0	0.01	0	0	0	0	0

0.12	0.1	0.07	0.03	0.05	0.07	0.05	0.07	0.07	0.08	0.09	0.11	0.1	0.03	0.06	0.18	0.08	0.16	0.02	0.07	0.08
0.04	0.04	0.02	0	0.01	0.01	0.01	0	0.01	0.03	0.01	0	0	0.01	0	0	0	0.02	0	0.01	0.01
0	0	0	0.01	0	0	0	0	0.01	0	0	0.01	0.01	0.01	0.08	0.01	0	0.01	0	0	0.01
0	0	0	0	0	0	0	0	0.01	0	0	0	0	0	0	0	0	0	0	0	0
0	0	0	0	0	0	0	0	0	0	0	0	0	0	0	0	0	0	0	0	0
0	0	0	0	0	0	0	0	0	0	0	0	0	0	0	0	0	0	0	0	0
0	0.01	0.01	0	0	0	0.01	0.01	0	0	0	0	0	0.01	0	0	0	0	0	0	0
0	0	0	0	0	0	0.01	0	0	0	0	0	0	0	0	0	0	0	0	0	0
0	0	0	0	0	0	0	0	0	0	0	0	0	0	0	0	0	0	0	0	0
0	0	0	0	0	0	0	0	0	0	0	0	0	0	0	0	0	0	0	0	0
0.01	0.01	0.02	0	0.01	0	0.01	0	0	0	0	0	0	0.02	0	0	0	0	0	0.01	0
0	0	0	0	0	0	0	0	0	0	0	0	0	0	0	0	0	0	0	0	0
0	0	0	0	0	0	0	0	0	0	0	0	0	0	0	0	0	0	0	0	0
0	0	0	0	0	0	0	0	0	0	0	0	0	0	0	0	0	0	0	0	0
0.03	0.05	0.05	0	0	0	0	0	0	0.01	0	0	0	0	0	0	0	0	0.01	0.01	0.01
0	0	0	0	0	0	0	0	0	0	0	0	0	0	0	0	0	0	0	0	0
0	0	0	0	0	0	0	0	0	0	0	0	0	0	0	0	0	0	0	0	0
0	0	0	0	0	0	0	0	0	0	0	0	0	0	0	0	0	0	0	0	0
0.01	0.01	0.01	0	0.01	0.01	0.01	0.01	0.01	0	0.01	0	0	0	0	0.01	0	0	0	0.01	0.01
0	0	0	0	0.01	0.01	0.01	0.01	0	0	0	0	0	0	0	0	0	0	0	0	0
0	0	0	0	0	0	0	0	0	0	0	0	0	0	0	0	0	0	0	0	0
0	0	0	0	0	0	0	0	0	0	0	0	0	0	0	0	0	0	0	0	0
0	0	0	0	0	0	0	0	0	0	0	0	0	0	0	0	0	0	0	0	0
0	0	0	0.01	0	0.01	0.01	0.01	0	0	0	0	0	0	0	0	0	0	0	0	0
0	0	0	0	0	0	0	0	0	0	0	0	0	0	0	0	0	0	0	0	0
0	0.01	0.01	0	0	0	0	0	0	0	0	0	0	0	0	0	0	0	0	0	0
0.04	0.09	0.05	0	0	0	0	0	0	0	0	0	0	0	0	0	0	0	0	0	0
0	0	0	0	0	0	0	0	0	0	0	0	0	0	0	0	0	0	0	0	0
0	0	0	0	0	0	0	0	0	0	0	0	0	0	0	0	0	0	0	0	0
0	0	0	0	0	0	0	0	0	0	0	0	0	0	0	0	0	0	0	0	0
0	0	0	0	0	0	0	0	0	0	0	0	0	0.01	0	0	0	0	0	0.01	0
0	0	0	0	0	0	0	0	0	0	0	0	0	0	0	0	0	0	0	0	0
0	0	0	0	0	0	0	0	0	0	0	0	0	0	0	0	0	0	0	0	0
0.03	0.06	0.09	0	0.01	0.01	0.01	0.01	0.02	0.08	0.01	0	0	0	0	0.01	0	0.01	0	0.01	0.01
0.04	0.04	0.1	0	0.01	0.01	0.01	0	0.05	0.17	0.03	0	0	0	0	0.01	0	0.01	0.01	0.01	0.01
0	0	0	0	0	0	0	0	0	0	0	0	0	0	0	0	0	0	0	0	0

0	0	0	0	0	0	0	0	0	0	0	0	0	0	0	0	0	0	0	0	0
0	0	0	0	0	0	0	0	0.01	0	0	0	0	0	0	0	0	0	0	0	0
0	0	0	0	0	0	0	0	0	0	0	0	0	0	0	0	0	0	0	0	0
0	0	0	0	0	0	0	0	0	0	0	0	0	0	0	0	0	0	0	0	0
0	0	0	0	0	0	0	0	0	0	0	0	0	0	0	0	0	0	0	0	0
0	0	0	0	0	0	0	0	0	0	0	0	0	0	0	0	0	0	0	0	0
0	0	0	0	0	0	0	0	0	0	0	0	0	0.01	0	0	0	0	0	0	0
0	0	0	0	0	0	0	0	0	0	0	0	0	0	0	0	0	0	0	0	0
0	0	0	0	0	0	0	0	0	0	0	0	0	0	0	0	0	0	0	0	0
0.04	0.03	0.04	0	0.01	0	0.01	0	0.01	0.06	0.01	0	0	0	0	0	0	0	0.01	0.01	0
0	0	0	0	0	0	0	0	0	0	0	0	0	0	0	0	0	0	0	0	0
0.01	0	0	0	0.24	0.23	0.26	0.19	0.09	0.01	0.06	0.02	0.06	0.1	0	0.06	0.04	0	0	0.01	0
0	0	0	0	0	0	0	0	0	0	0	0	0	0	0	0	0	0	0	0	0
0	0	0	0	0	0	0	0	0	0	0	0	0	0	0	0	0	0	0	0	0
0	0	0	0	0	0	0	0	0	0	0	0	0	0	0	0	0	0	0	0	0
0	0	0	0	0	0	0	0	0	0	0	0	0	0.01	0	0	0	0	0	0.01	0.01
0	0	0	0	0	0	0	0	0	0	0	0	0	0	0	0	0	0	0	0	0
0	0	0	0	0	0	0	0	0	0	0	0	0	0	0	0	0	0	0	0	0
0	0	0	0	0	0	0	0	0	0	0	0	0	0	0	0	0	0	0	0	0
0	0	0	0	0	0	0	0	0	0	0	0	0	0	0	0	0	0	0	0	0
0	0.01	0.01	0	0	0	0	0	0	0	0	0	0	0	0	0	0	0.01	0	0.03	0
0	0	0	0	0	0	0	0	0	0	0	0	0	0	0	0	0	0	0	0	0
0	0	0	0	0	0	0	0	0	0	0	0	0	0	0	0	0	0	0	0	0
0	0.01	0.01	0.01	0.01	0.01	0.01	0	0	0	0	0	0.01	0.02	0.01	0.01	0.01	0	0	0	0.01
0	0	0	0	0	0	0	0	0	0	0	0	0	0	0	0	0	0	0	0	0
0	0	0	0	0	0	0	0	0	0	0	0	0	0	0	0	0	0	0	0	0
0	0	0	0	0	0	0	0	0	0	0	0	0	0	0	0	0	0	0	0	0
0.01	0.01	0.01	0	0	0	0	0	0	0	0	0	0	0	0	0	0	0	0	0	0
0	0	0	0	0	0	0	0	0	0	0	0	0	0	0	0	0	0	0	0	0
0	0	0	0	0	0	0	0	0	0	0	0	0	0	0	0.04	0	0	0	0	0
0	0	0	0	0.01	0	0	0	0	0	0	0	0	0	0	0	0	0	0	0	0
0	0	0	0	0.01	0.02	0.01	0.01	0	0	0.01	0	0	0.04	0	0	0	0	0	0	0
0	0	0	0	0.01	0.01	0.01	0.02	0	0	0	0	0	0.01	0.01	0	0	0.01	0	0.02	0
0.01	0.01	0	0	0.03	0.05	0.03	0.03	0.01	0	0.01	0	0	0.06	0.02	0.03	0	0.04	0	0.03	0.03
0.02	0.01	0.01	0	0.01	0.01	0	0	0.01	0	0	0	0	0	0	0	0.01	0	0	0.02	0
0.01	0.01	0	0	0.01	0	0.01	0.01	0	0.01	0.01	0.01	0.01	0	0.02	0.01	0.03	0	0	0.02	0

0.01	0	0	0	0.01	0.01	0.01	0	0.01	0.01	0	0.01	0	0	0	0.01	0.01	0	0	0	0
0	0	0	0	0.03	0.03	0.03	0.02	0.02	0	0.02	0.01	0	0.07	0	0.05	0.01	0.05	0	0.01	0.03
0.01	0.01	0	0	0.03	0.05	0.02	0.04	0	0	0	0	0	0.02	0.01	0	0	0.02	0	0.02	0.01
0	0	0	0	0	0	0	0	0	0	0	0	0	0.01	0	0	0	0	0	0	0
0	0.01	0	0	0.01	0	0.01	0	0	0	0	0.01	0	0.01	0	0	0	0.01	0	0	0
0	0	0	0	0	0	0	0	0	0	0	0	0	0	0	0	0	0	0	0	0
0	0	0	0	0	0	0	0	0	0	0	0	0	0	0	0	0	0	0	0	0
0	0	0	0	0	0	0	0	0	0	0	0	0	0	0	0	0	0	0	0	0
0	0	0	0	0	0	0	0	0	0	0	0	0	0	0	0	0	0	0	0	0
0	0	0	0	0	0	0	0	0	0	0	0	0	0	0	0	0	0	0	0	0
0	0	0	0	0.01	0.01	0.01	0.01	0.01	0	0	0.02	0.02	0.01	0.02	0	0.02	0	0	0	0
0.01	0	0.01	0	0.04	0.02	0.02	0.02	0.01	0.01	0.02	0.05	0.07	0.05	0.01	0.01	0.06	0	0	0	0
0	0	0.01	0	0	0	0	0	0	0	0	0.01	0	0	0	0	0.01	0	0	0	0
0	0	0	0	0	0	0	0	0	0	0	0	0	0	0	0	0	0	0	0	0
0	0	0	0	0	0	0	0	0	0	0	0	0	0	0	0	0	0	0	0	0
0	0	0.01	0	0	0	0	0	0	0	0	0	0	0	0	0	0	0.01	0	0	0.01
0	0	0	0	0	0	0	0	0	0	0	0	0	0	0	0	0	0	0	0	0
0	0	0	0	0	0	0	0	0	0	0	0	0	0	0	0	0	0	0	0	0
0	0	0	0	0	0	0	0	0	0	0	0	0	0	0	0	0	0	0	0	0
0	0	0	0	0	0	0	0	0	0	0	0	0	0	0	0	0	0	0	0	0
0	0.01	0	0	0.01	0.01	0.01	0.01	0	0	0	0	0	0.02	0.01	0	0	0	0	0	0.01
0	0	0	0	0	0	0	0	0	0	0	0	0	0	0	0	0	0	0	0	0
0.01	0	0	0	0	0	0	0	0.01	0	0	0	0	0	0	0	0	0	0	0	0
0.01	0.01	0	0	0	0.01	0	0	0.01	0	0	0	0.01	0	0.01	0.01	0.02	0.01	0.03	0.01	0.02
0.01	0.01	0.01	0	0.01	0	0.01	0.01	0.03	0.01	0.02	0.02	0.01	0.01	0	0.01	0.02	0	0.01	0.01	0.01
0	0	0	0	0.01	0.01	0.02	0.01	0	0	0	0	0	0	0	0	0	0	0	0	0
0.02	0.04	0.02	0	0.02	0.01	0.01	0.02	0	0	0	0	0	0.02	0	0.01	0.01	0.01	0.02	0.01	0.01
0	0.01	0	0	0	0	0.01	0	0	0	0	0	0	0	0	0	0	0	0.03	0	0.01
0	0	0	0	0	0	0	0	0	0	0	0	0	0	0	0	0	0	0	0	0
0	0	0	0	0	0	0	0	0	0	0	0	0	0	0	0	0	0	0	0	0
0	0	0	0	0	0	0	0	0	0	0	0	0	0	0	0	0	0	0.01	0	0
0.01	0	0	0	0	0	0	0	0	0	0	0	0	0	0	0	0	0	0	0	0
0	0	0	0	0	0	0	0	0	0	0	0	0	0	0	0	0	0	0	0	0
0	0	0	0	0	0	0	0	0	0	0	0	0	0	0	0	0	0	0	0	0
0	0	0	0	0	0	0	0	0	0	0	0	0	0	0	0	0	0	0	0	0
0	0	0	0	0	0	0	0	0	0	0	0	0	0	0	0	0	0	0	0	0

0	0	0	0	0	0	0	0	0	0	0	0	0	0	0	0	0	0	0	0	0
0	0	0	0	0	0	0	0	0	0	0	0	0	0	0	0	0	0	0	0	0
0	0	0	0	0	0	0	0	0	0	0	0	0	0	0	0	0	0	0	0	0
0	0	0	0	0	0	0	0	0	0	0	0	0	0	0	0	0	0	0	0	0
0	0	0	0	0	0	0	0	0	0	0	0	0	0	0	0	0	0	0	0	0
0	0	0	0	0	0	0	0	0	0	0	0	0	0	0	0	0	0	0	0	0
0	0	0	0	0	0	0	0	0	0	0	0	0	0	0	0	0	0	0	0	0
0	0	0	0	0	0	0	0	0	0	0	0	0	0	0	0	0	0	0	0	0
0	0	0	0	0	0	0	0	0	0	0	0	0	0	0	0	0	0	0	0	0
0	0	0	0	0	0	0	0	0	0	0	0	0	0	0	0	0	0	0	0	0
0	0.01	0	0	0	0	0	0	0	0	0	0	0	0	0	0	0	0	0	0	0
0	0	0	0	0	0	0	0	0.01	0	0	0	0	0	0	0	0	0	0	0	0
0	0	0	0	0	0	0	0	0	0	0	0	0	0	0	0	0	0	0	0	0
0	0.01	0	0	0	0	0	0	0	0	0	0	0	0	0	0	0	0	0	0	0
0	0	0	0	0	0	0	0	0	0	0	0	0	0	0	0	0	0	0	0.01	0
0	0	0	0	0	0	0	0	0	0	0	0	0	0	0	0	0	0	0	0	0
0	0	0	0	0	0	0	0	0	0	0	0	0	0	0	0	0	0	0	0	0
0	0	0	0	0	0	0	0	0	0	0	0	0	0	0	0	0	0	0	0	0
0	0	0	0	0	0	0	0	0	0	0	0	0	0	0	0	0	0	0	0	0
0	0	0	0	0	0	0	0	0	0	0	0	0	0	0	0	0	0	0	0	0
0	0	0	0	0	0	0	0	0	0	0	0	0	0	0	0	0	0	0	0	0
0	0	0	0	0	0	0	0	0	0	0	0	0	0	0	0	0	0	0	0	0
0	0	0	0	0	0	0	0	0	0	0	0	0	0	0	0	0	0	0	0	0
0	0	0	0	0	0	0	0	0	0	0	0	0	0	0	0	0	0	0	0	0
0	0	0	0	0	0	0	0	0	0	0	0	0	0	0	0	0	0	0	0	0
0	0	0	0	0	0	0	0	0	0	0	0	0	0	0	0	0	0	0	0	0
0	0	0	0	0	0	0	0	0	0	0	0	0	0	0	0	0	0	0	0	0
0	0	0	0	0	0	0	0	0	0	0	0	0	0	0	0	0	0	0	0	0
0	0	0	0	0	0	0	0	0	0	0	0	0	0	0	0	0	0	0	0	0
0	0	0	0	0	0	0	0	0	0	0	0	0	0	0	0	0	0	0	0	0
0	0	0	0	0	0	0	0	0	0	0	0	0	0.01	0	0	0	0	0	0	0
0	0	0	0	0	0	0	0	0	0	0	0	0	0	0	0	0	0	0	0	0
0	0	0	0	0	0	0	0	0	0	0	0	0	0	0	0	0	0	0	0	0
0	0	0	0	0	0	0	0	0	0	0	0	0	0	0	0	0	0	0	0	0
0	0	0	0	0	0	0	0	0	0	0	0	0	0	0	0	0	0	0	0	0
0	0	0	0	0	0	0	0	0	0	0	0	0	0	0	0	0	0	0	0	0

0	0	0	0	0	0	0	0	0	0	0	0	0	0	0	0	0	0	0	0	0
0	0	0	0	0	0	0	0	0	0	0	0	0	0	0	0	0	0	0	0	0
0.01	0	0	0	0	0.01	0.01	0.01	0	0	0	0	0	0	0	0	0	0	0	0	0.01
0	0	0	0	0.01	0.01	0.01	0.01	0	0	0	0	0	0	0	0	0	0	0.01	0	0
0	0	0	0	0	0	0	0	0	0	0	0	0	0	0	0	0	0	0	0	0
0	0	0	0	0	0	0	0	0	0	0	0	0	0	0	0	0	0	0	0	0
0	0	0	0	0	0	0	0	0	0	0	0	0	0	0	0	0	0	0	0	0
0	0	0	0	0	0	0	0	0	0	0	0	0	0	0	0	0	0.02	0	0	0
0.01	0	0.02	0	0	0	0	0	0.01	0	0	0	0	0	0	0	0	0	0	0	0
0	0	0	0	0	0	0	0	0	0	0	0	0	0	0	0	0	0.05	0	0	0
0	0	0	0	0	0	0	0	0	0	0	0	0	0	0	0	0	0	0	0	0
0	0	0	0	0	0	0	0	0	0	0	0	0	0	0	0	0	0.01	0	0	0
0	0	0	0	0	0	0	0	0	0	0	0	0	0	0	0	0	0	0	0	0
0	0	0	0	0	0	0	0	0	0	0	0	0	0	0	0	0	0	0	0	0
0	0	0	0	0	0	0	0	0	0	0	0	0	0	0	0	0	0	0	0	0
0	0	0	0	0	0	0	0	0	0	0	0	0	0	0	0	0	0	0	0	0
0	0	0	0	0	0	0	0	0	0	0	0	0	0	0	0	0	0	0	0	0
0	0	0	0	0	0	0	0	0	0	0	0	0	0	0	0	0	0	0	0	0
0	0	0	0	0	0	0	0	0	0	0	0	0	0	0	0	0	0	0	0	0
0	0	0	0	0	0	0	0	0	0	0	0	0	0	0	0	0	0.01	0	0	0
0	0	0	0	0	0	0	0	0	0	0	0	0	0	0	0	0	0	0	0	0
0	0	0	0	0	0	0	0	0	0	0	0	0	0	0	0	0	0	0	0	0
0	0	0	0	0	0	0	0	0	0	0	0	0	0	0	0	0	0	0	0	0
0	0	0	0	0	0	0	0	0.01	0	0	0	0	0	0	0	0	0	0.01	0	0
0	0	0	0	0	0	0	0	0	0	0	0	0	0.01	0	0	0	0	0	0.01	0
0.04	0.03	0.04	0	0.01	0.02	0.03	0.01	0	0	0.01	0	0	0.01	0	0	0	0.02	0	0	0
0.01	0.01	0	0	0	0	0	0	0	0	0	0	0	0	0	0.01	0	0	0	0	0.02
0	0	0	0	0	0	0	0	0	0	0	0	0	0	0	0	0	0	0	0	0
0	0	0	0	0	0	0	0	0	0	0	0	0	0	0	0	0	0	0	0	0
0	0	0	0	0	0	0	0	0	0	0	0	0	0	0	0	0	0	0	0	0
0	0	0	0	0	0	0	0	0	0	0	0	0	0	0	0	0	0	0	0	0
0	0	0	0	0	0	0	0	0	0	0	0	0	0	0	0	0	0	0	0	0
0	0	0	0	0	0	0	0	0	0	0	0	0	0	0	0	0	0	0	0	0
0	0	0	0	0	0	0	0	0	0	0	0	0	0	0	0	0	0	0	0	0
0	0	0	0	0	0	0	0	0	0	0	0	0	0	0	0	0	0	0	0	0
0	0	0	0	0	0	0	0	0	0	0	0	0	0	0	0	0	0	0	0	0

0	0	0	0	0	0	0	0	0	0	0	0	0	0	0	0	0	0	0	0	0
0	0	0	0.01	0	0	0	0	0	0	0	0	0	0	0.01	0	0	0	0	0	0
0	0	0	0	0	0	0	0	0	0	0	0	0	0	0	0	0	0	0	0	0
0	0	0	0	0	0	0	0	0	0	0	0	0	0	0	0	0	0	0	0	0
0	0	0	0	0	0	0	0	0	0	0	0	0	0	0	0	0	0	0	0	0
0	0	0	0	0	0	0	0	0	0	0	0	0	0	0	0	0	0	0	0	0
0	0	0	0	0	0	0	0	0	0	0	0	0	0	0	0	0	0	0	0	0
0	0	0	0	0	0	0	0	0	0	0	0	0	0	0	0	0	0	0	0	0
0	0	0	0	0	0	0	0	0	0	0	0	0	0	0	0	0	0	0	0	0
0	0	0	0	0	0	0	0	0	0	0	0	0	0	0	0	0	0	0	0	0
0	0	0	0	0	0	0	0	0	0	0	0	0	0	0	0	0	0	0	0	0
0	0	0	0	0	0	0	0	0	0	0	0	0	0	0	0	0	0	0	0	0
0	0	0	0	0	0	0	0	0	0	0	0	0	0	0	0	0	0	0	0	0
0	0	0	0	0	0	0	0	0	0	0	0	0	0	0	0	0	0	0	0	0
0	0	0	0	0	0	0	0	0	0	0	0	0	0	0	0	0	0	0	0	0
0	0	0	0	0	0	0	0	0	0	0	0	0	0	0	0	0	0	0	0	0
0	0	0	0	0	0	0	0	0	0	0	0	0	0	0	0	0	0	0.01	0	0
0	0	0	0	0	0	0	0	0	0	0	0	0	0	0	0	0	0	0	0	0
0	0	0	0	0	0	0	0	0	0	0	0	0	0	0	0	0	0.01	0	0.01	0
0	0	0	0	0	0	0	0	0	0	0	0	0	0	0	0	0	0	0	0	0
0	0	0	0	0	0	0	0	0	0	0	0	0	0	0	0	0	0	0	0	0
0	0	0	0	0	0	0	0	0	0	0	0	0	0	0	0	0	0	0	0	0
0	0	0	0	0	0	0	0	0	0	0	0	0	0	0	0	0	0.01	0	0	0
0	0	0	0	0	0	0	0	0	0	0	0	0	0	0	0	0	0	0	0	0
0	0	0	0	0	0	0	0	0	0	0	0	0	0	0	0	0	0	0	0	0
0	0	0	0	0	0	0	0	0	0	0	0	0	0	0	0	0	0	0	0	0
0	0	0	0	0	0	0	0	0	0	0	0	0	0	0	0	0	0	0	0	0
0	0	0	0	0	0	0	0	0	0	0	0	0	0	0	0	0	0	0	0	0
0	0	0	0	0	0	0	0	0	0	0	0	0	0	0	0	0	0	0	0	0
0	0	0	0	0	0	0	0	0	0	0	0	0	0	0	0	0	0	0	0	0
0	0	0	0	0	0	0	0	0	0	0	0	0	0	0	0	0	0	0	0	0
0	0	0	0	0	0	0	0	0	0	0	0	0	0	0	0	0	0	0	0	0
0	0	0	0	0	0	0	0	0	0	0	0	0	0	0	0	0	0	0	0	0
0	0	0	0	0	0	0	0	0	0	0	0	0	0	0	0	0	0	0	0	0
0.01	0	0	0	0	0	0	0	0	0	0	0	0	0	0	0	0	0.01	0	0	0
0	0	0	0	0	0	0	0	0	0	0	0	0	0	0	0	0	0	0	0	0

0	0	0	0	0	0	0	0	0	0	0	0	0	0	0	0	0	0	0	0	0
0	0	0	0	0	0	0	0	0	0	0	0	0	0	0	0	0	0	0	0	0
0	0	0	0	0	0	0	0	0	0	0	0	0	0	0	0	0	0	0	0	0
0	0	0	0	0	0	0	0	0	0	0	0	0	0	0	0	0	0	0	0	0
0	0	0	0	0	0	0	0	0	0	0	0	0	0	0	0	0	0	0	0	0
0	0	0	0	0	0	0	0	0	0	0	0	0	0	0	0	0	0	0	0	0
0	0	0	0	0	0	0	0	0	0	0	0	0	0	0	0	0	0	0	0	0
0	0	0	0	0	0	0	0	0	0	0	0	0	0	0	0	0	0	0	0	0
0	0	0	0	0	0	0	0	0	0	0	0	0	0	0	0	0	0	0	0	0
0	0	0	0	0	0	0	0	0	0	0	0	0	0	0	0	0	0	0	0	0
0	0	0	0	0	0	0	0	0	0	0	0	0	0	0	0	0	0	0	0	0
0	0	0	0	0	0	0	0	0	0	0	0	0	0	0	0	0	0	0	0	0
0	0	0	0	0	0	0	0	0	0	0	0	0	0	0	0	0	0	0	0	0
0	0	0	0	0	0	0	0	0	0	0	0	0	0	0	0	0	0	0	0	0
0	0	0	0	0	0	0	0	0	0	0	0	0	0	0	0	0	0	0	0	0
0	0	0	0	0	0	0	0	0	0	0	0	0	0	0	0	0	0	0	0	0
0	0	0	0	0	0	0	0	0	0	0	0	0	0	0	0	0	0	0	0	0
0	0	0	0	0	0	0	0	0	0	0	0	0	0	0	0	0	0	0	0	0
0	0	0	0	0	0	0	0	0	0	0	0	0	0	0	0	0	0	0	0	0
0	0	0	0	0	0	0	0	0	0	0	0	0	0	0	0	0	0	0	0	0
0	0	0	0	0	0	0	0	0	0	0	0	0	0	0	0	0	0	0	0	0
0	0	0	0	0	0	0	0	0	0	0	0	0	0	0	0	0	0	0	0	0
0	0	0	0	0	0	0	0	0	0	0	0	0	0	0	0	0	0	0	0	0
0	0	0	0	0	0	0	0	0	0	0	0	0	0	0	0	0	0	0	0	0
0	0	0	0	0	0	0	0	0	0	0	0	0	0	0	0	0	0	0	0	0
0	0	0	0	0	0	0	0	0	0	0	0	0	0	0	0	0	0	0	0	0
0	0	0	0	0	0	0	0	0	0	0	0	0	0	0	0	0	0	0	0	0
0	0	0	0	0	0	0	0	0	0	0	0	0	0	0	0	0	0	0	0	0
0	0	0	0	0	0	0	0	0	0	0	0	0	0	0	0	0	0	0	0	0
0	0	0	0	0	0	0	0	0	0	0	0	0	0	0	0	0	0	0	0	0
0	0	0	0	0	0	0	0	0	0	0	0	0	0	0	0	0	0	0	0	0
0	0	0	0	0	0	0	0	0	0	0	0	0	0	0	0	0	0	0	0	0
0	0	0	0	0	0	0	0	0	0	0	0	0	0	0	0	0	0	0	0	0
0	0	0	0	0	0	0	0	0	0	0	0	0	0	0	0	0	0	0	0	0
0	0	0	0	0	0	0	0	0	0	0	0	0	0	0	0	0	0	0	0	0
0	0	0	0	0	0	0	0	0	0	0	0	0	0	0	0	0	0	0	0	0

0	0	0	0	0	0	0	0	0	0	0	0	0	0	0	0	0	0	0	0	0
0	0	0	0	0	0	0	0	0	0	0	0	0	0	0	0	0	0	0	0	0
0	0	0	0	0	0	0	0	0	0	0	0	0	0	0	0	0	0	0	0	0
0	0	0	0	0	0	0	0	0	0	0	0	0	0	0	0	0	0	0	0	0
0	0	0	0	0	0	0	0	0	0	0	0	0	0	0	0	0	0	0	0	0
0	0	0	0	0	0	0	0	0	0	0	0	0	0	0	0	0	0	0	0	0
0	0	0	0	0	0	0	0	0	0	0	0	0	0	0	0	0	0	0	0	0
0	0	0	0	0	0	0	0	0	0	0	0	0	0	0	0	0	0	0	0	0
0	0	0	0	0	0	0	0	0	0	0	0	0	0	0	0	0	0	0	0	0
0	0	0	0	0	0	0	0	0	0	0	0	0	0	0	0	0	0	0	0	0
0	0	0	0	0	0	0	0	0	0	0	0	0	0	0	0	0	0	0	0	0
0	0	0	0	0	0	0	0	0	0	0	0	0	0	0	0	0	0	0	0	0
0	0	0	0	0	0	0	0	0	0	0	0	0	0	0	0	0	0	0	0	0
0	0	0	0	0	0	0	0	0	0	0	0	0	0	0	0	0	0	0	0	0
0	0	0	0	0	0	0	0	0	0	0	0	0	0	0	0	0	0	0	0	0
0	0	0	0	0	0	0	0	0	0	0	0	0	0	0	0	0	0	0	0	0
0	0	0	0	0	0	0	0	0	0	0	0	0	0	0	0	0	0	0	0	0
0	0	0	0	0	0	0	0	0	0	0	0	0	0	0	0	0	0	0	0	0
0	0	0	0	0	0	0	0	0	0	0	0	0	0	0	0	0	0	0	0	0
0	0	0	0	0	0	0	0	0	0	0	0	0	0	0	0	0	0	0	0	0
0	0	0	0	0	0	0	0	0	0	0	0	0	0	0	0	0	0	0	0	0
0	0	0	0	0	0	0	0	0	0	0	0	0	0	0	0	0	0	0	0	0
0	0	0	0	0	0	0	0	0	0	0	0	0	0	0	0	0	0	0	0	0
0	0	0	0	0	0	0	0	0	0	0	0	0	0	0	0	0	0	0	0	0
0	0	0	0	0	0	0	0	0	0	0	0	0	0	0	0	0	0	0	0	0
0	0	0	0	0	0	0	0	0	0	0	0	0	0	0	0	0	0	0	0	0
0	0	0	0	0	0	0	0	0	0	0	0	0	0	0	0	0	0	0	0	0
0	0	0	0	0	0	0	0	0	0	0	0	0	0	0	0	0	0	0	0	0
0	0	0	0	0	0	0	0	0	0	0	0	0	0	0	0	0	0	0	0	0
0	0	0	0	0	0	0	0	0	0	0	0	0	0	0	0	0	0	0	0	0
0	0	0	0	0	0	0	0	0	0	0	0	0	0	0	0	0	0	0	0	0
0	0	0	0	0	0	0	0	0	0	0	0	0	0	0	0	0	0	0	0	0
0	0	0	0	0	0	0	0	0	0	0	0	0	0	0	0	0	0	0	0	0
0	0	0	0	0	0	0	0	0	0	0	0	0	0	0	0	0	0	0	0	0
0	0	0	0	0	0	0	0	0	0	0	0	0	0	0	0	0	0	0	0	0
0	0	0	0	0	0	0	0	0	0	0	0	0	0	0	0	0	0	0	0	0

0	0	0	0	0	0	0	0	0	0	0	0	0	0	0	0	0	0	0	0	0
0	0	0	0	0	0	0	0	0	0	0	0	0	0	0	0	0	0	0	0	0
0	0	0	0	0	0	0	0	0	0	0	0	0	0	0	0	0	0	0	0	0
0	0	0	0	0	0	0	0	0	0	0	0	0	0	0	0	0	0	0	0	0
0	0	0	0	0	0	0	0	0	0	0	0	0	0	0	0	0	0	0	0	0
0	0	0	0	0	0	0	0	0	0	0	0	0	0	0	0	0	0	0	0	0
0	0	0	0	0	0	0	0	0	0	0	0	0	0	0	0	0	0	0	0	0
0	0	0	0	0	0	0	0	0	0	0	0	0	0	0	0	0	0	0	0	0
0	0	0	0	0	0	0	0	0	0	0	0	0	0	0	0	0	0	0	0	0
0	0	0	0	0	0	0	0	0	0	0	0	0	0	0	0	0	0	0	0	0
0	0	0	0	0	0	0	0	0	0	0	0	0	0	0	0	0	0	0	0	0
0	0	0	0	0	0	0	0	0	0	0	0	0	0	0	0	0	0	0	0	0
0	0	0	0	0	0	0	0	0	0	0	0	0	0	0	0	0	0	0	0	0
0	0	0	0	0	0	0	0	0	0	0	0	0	0	0	0	0	0	0	0	0
0	0	0	0	0	0	0	0	0	0	0	0	0	0	0	0	0	0	0	0	0
0	0	0	0	0	0	0	0	0	0	0	0	0	0	0	0	0	0	0	0	0
0	0	0	0	0	0	0	0	0	0	0	0	0	0	0	0	0	0	0	0	0
0	0	0	0	0	0	0	0	0	0	0	0	0	0	0	0	0	0	0	0	0
0	0	0	0	0	0	0	0	0	0	0	0	0	0	0	0	0	0	0	0	0
0	0	0	0	0	0	0	0	0	0	0	0	0	0	0	0	0	0	0	0	0
0	0	0	0	0	0	0	0	0	0	0	0	0	0	0	0	0	0	0	0	0
0	0	0	0	0	0	0	0	0	0	0	0	0	0	0	0	0	0	0	0	0
0	0	0	0	0	0	0	0	0	0	0	0	0	0	0	0	0	0	0	0	0
0	0	0	0	0	0	0	0	0	0	0	0	0	0	0	0	0	0	0	0	0
0	0	0	0	0	0	0	0	0	0	0	0	0	0	0	0	0	0	0	0	0
0	0	0	0	0	0	0	0	0	0	0	0	0	0	0	0	0	0	0	0	0
0	0	0	0	0	0	0	0	0	0	0	0	0	0	0	0	0	0	0	0	0
0	0	0	0	0	0	0	0	0	0	0	0	0	0	0	0	0	0	0	0	0
0	0	0	0	0	0	0	0	0	0	0	0	0	0	0	0	0	0	0	0	0
0	0	0	0	0	0	0	0	0	0	0	0	0	0	0	0	0	0	0	0	0
0	0	0	0	0	0	0	0	0	0	0	0	0	0	0	0	0	0	0	0	0
0	0	0	0	0	0	0	0	0	0	0	0	0	0	0	0	0	0	0	0	0
0	0	0	0	0	0	0	0	0	0	0	0	0	0	0	0	0	0	0	0	0
0	0	0	0	0	0	0	0	0	0	0	0	0	0	0	0	0	0	0	0	0
0	0	0	0	0	0	0	0	0	0	0	0	0	0	0	0	0	0	0	0	0
0	0	0	0	0	0	0	0	0	0	0	0	0	0	0	0	0	0	0	0	0

0	0	0	0	0	0	0	0	0	0	0	0	0	0	0	0	0	0	0	0	0
0	0	0	0	0	0	0	0	0	0	0	0	0	0	0	0	0	0	0	0	0
0	0	0	0	0	0	0	0	0	0	0	0	0	0	0	0	0	0	0	0	0
0	0	0	0	0	0	0	0	0	0	0	0	0	0	0	0	0	0	0	0	0
0	0	0	0	0	0	0	0	0	0	0	0	0	0	0	0	0	0	0	0	0
0	0	0	0	0	0	0	0	0	0	0	0	0	0	0	0	0	0	0	0	0
0	0	0	0	0	0	0	0	0	0	0	0	0	0	0	0	0	0	0	0	0
0	0	0	0	0	0	0	0	0	0	0	0	0	0	0	0	0	0	0	0	0
0	0	0	0	0	0	0	0	0	0	0	0	0	0	0	0	0	0	0	0	0
0	0	0	0	0	0	0	0	0	0	0	0	0	0	0	0	0	0	0	0	0
0	0	0	0	0	0	0	0	0	0	0	0	0	0	0	0	0	0	0	0	0
0	0	0	0	0	0	0	0	0	0	0	0	0	0	0	0	0	0	0	0	0
0	0	0	0	0	0	0	0	0	0	0	0	0	0	0	0	0	0	0	0	0
0	0	0	0	0	0	0	0	0	0	0	0	0	0	0	0	0	0	0	0	0
0	0	0	0	0	0	0	0	0	0	0	0	0	0	0	0	0	0	0	0	0
0	0	0	0	0	0	0	0	0	0	0	0	0	0	0	0	0	0	0	0	0
0	0	0	0	0	0	0	0	0	0	0	0	0	0	0	0	0	0	0	0	0
0	0	0	0	0	0	0	0	0	0	0	0	0	0	0	0	0	0	0	0	0
0	0	0	0	0	0	0	0	0	0	0	0	0	0	0	0	0	0	0	0	0
0	0	0	0	0	0	0	0	0	0	0	0	0	0	0	0	0	0	0	0	0
0	0	0	0	0	0	0	0	0	0	0	0	0	0	0	0	0	0	0	0	0
0	0	0	0	0	0	0	0	0	0	0	0	0	0	0	0	0	0	0	0	0
0	0	0	0	0	0	0	0	0	0	0	0	0	0	0	0	0	0	0	0	0
0	0	0	0	0	0	0	0	0	0	0	0	0	0	0	0	0	0	0	0	0
0	0	0	0	0	0	0	0	0	0	0	0	0	0	0	0	0	0	0	0	0
0	0	0	0	0	0	0	0	0	0	0	0	0	0	0	0	0	0	0	0	0
0	0	0	0	0	0	0	0	0	0	0	0	0	0	0	0	0	0	0	0	0
0	0	0	0	0	0	0	0	0	0	0	0	0	0	0	0	0	0	0	0	0
0	0	0	0	0	0	0	0	0	0	0	0	0	0	0	0	0	0	0	0	0
0	0	0	0	0	0	0	0	0	0	0	0	0	0	0	0	0	0	0	0	0
0	0	0	0	0	0	0	0	0	0	0	0	0	0	0	0	0	0	0	0	0
0	0	0	0	0	0	0	0	0	0	0	0	0	0	0	0	0	0	0	0	0
0	0	0	0	0	0	0	0	0	0	0	0	0	0	0	0	0	0	0	0	0
0	0	0	0	0	0	0	0	0	0	0	0	0	0	0	0	0	0	0	0	0
0	0	0	0	0	0	0	0	0	0	0	0	0	0	0	0	0	0	0	0	0
0	0	0	0	0	0	0	0	0	0	0	0	0	0	0	0	0	0	0	0	0

0	0	0	0	0	0	0	0	0	0	0	0	0	0	0	0	0	0	0	0	0
0	0	0	0	0	0	0	0	0	0	0	0	0	0	0	0	0	0	0	0	0
0	0	0	0	0	0	0	0	0	0	0	0	0	0	0	0	0	0	0	0	0
0	0	0	0	0	0	0	0	0	0	0	0	0	0	0	0	0	0	0	0	0
0	0	0	0	0	0	0	0	0	0	0	0	0	0	0	0	0	0	0	0	0
0	0	0	0	0	0	0	0	0	0	0	0	0	0	0	0	0	0	0	0	0
0	0	0	0	0	0	0	0	0	0	0	0	0	0	0	0	0	0	0	0	0
0	0	0	0	0	0	0	0	0	0	0	0	0	0	0	0	0	0	0	0	0
0	0	0	0	0	0	0	0	0	0	0	0	0	0	0	0	0	0	0	0	0
0	0	0	0	0	0	0	0	0	0	0	0	0	0	0	0	0	0	0	0	0
0	0	0	0	0	0	0	0	0	0	0	0	0	0	0	0	0	0	0	0	0
0	0	0	0	0	0	0	0	0	0	0	0	0	0	0	0	0	0	0	0	0
0	0	0	0	0	0	0	0	0	0	0	0	0	0	0	0	0	0	0	0	0
0	0	0	0	0	0	0	0	0	0	0	0	0	0	0	0	0	0	0	0	0
0	0	0	0	0	0	0	0	0	0	0	0	0	0	0	0	0	0	0	0	0
0	0	0	0	0	0	0	0	0	0	0	0	0	0	0	0	0	0	0	0	0
0	0	0	0	0	0	0	0	0	0	0	0	0	0	0	0	0	0	0	0	0
0	0	0	0	0	0	0	0	0	0	0	0	0	0	0	0	0	0	0	0	0
0	0	0	0	0	0	0	0	0	0	0	0	0	0	0	0	0	0	0	0	0
0	0	0	0	0	0	0	0	0	0	0	0	0	0	0	0	0	0	0	0	0
0	0	0	0	0	0	0	0	0	0	0	0	0	0	0	0	0	0	0	0	0
0	0	0	0	0	0	0	0	0	0	0	0	0	0	0	0	0	0	0	0	0
0	0	0	0	0	0	0	0	0	0	0	0	0	0	0	0	0	0	0	0	0
0	0	0	0	0	0	0	0	0	0	0	0	0	0	0	0	0	0	0	0	0
0	0	0	0	0	0	0	0	0	0	0	0	0	0	0	0	0	0	0	0	0
0	0	0	0	0	0	0	0	0	0	0	0	0	0	0	0	0	0	0	0	0
0	0	0	0	0	0	0	0	0	0	0	0	0	0	0	0	0	0	0	0	0
0	0	0	0	0	0	0	0	0	0	0	0	0	0	0	0	0	0	0	0	0
0	0	0	0	0	0	0	0	0	0	0	0	0	0	0	0	0	0	0	0	0
0	0	0	0	0	0	0	0	0	0	0	0	0	0	0	0	0	0	0	0	0
0	0	0	0	0	0	0	0	0	0	0	0	0	0	0	0	0	0	0	0	0
0	0.01	0	0	0	0	0	0	0	0	0	0	0	0	0	0	0	0	0	0	0
0	0	0	0	0	0	0	0	0	0	0	0	0	0	0	0	0	0	0	0	0
0	0	0	0	0	0	0	0	0	0	0	0	0	0	0	0	0	0	0	0	0
0	0	0	0	0	0	0	0	0	0	0	0	0	0	0	0	0	0	0	0	0
0	0	0	0	0	0	0	0	0	0	0	0	0	0	0	0	0	0	0	0	0

0	0	0	0	0	0	0	0	0	0	0	0	0	0	0	0	0	0	0	0	0
0	0	0	0	0	0	0	0	0	0	0	0	0	0	0	0	0	0	0	0	0
0	0	0	0	0	0	0	0	0	0	0	0	0	0	0	0	0	0	0	0	0
0	0	0	0	0	0	0	0	0	0	0	0	0	0	0	0	0	0	0	0	0
0	0	0	0	0	0	0	0	0	0	0	0	0	0	0	0	0	0	0	0	0
0	0	0	0	0	0	0	0	0	0	0	0	0	0	0	0	0	0	0	0	0
0	0	0	0	0	0	0	0	0	0	0	0	0	0	0	0	0	0	0	0	0
0	0	0	0	0	0	0	0	0	0	0	0	0	0	0	0	0	0	0	0	0
0	0	0	0	0	0	0	0	0	0	0	0	0	0	0	0	0	0	0	0	0
0	0	0	0	0	0	0	0	0	0	0	0	0	0	0	0	0	0	0	0	0
0	0	0	0	0	0	0	0	0	0	0	0	0	0	0	0	0	0	0	0	0
0	0	0	0	0	0	0	0	0	0	0	0	0	0	0	0	0	0	0	0	0
0	0	0	0	0	0	0	0	0	0	0	0	0	0	0	0	0	0	0	0	0
0	0	0.02	0	0.01	0	0.01	0	0.01	0	0	0	0	0	0	0	0	0	0	0.01	0
0	0	0	0	0	0	0	0	0	0	0	0	0	0	0	0	0	0.01	0.02	0.01	0.02
0	0	0	0	0	0	0	0	0	0	0	0	0	0	0.01	0	0	0	0.02	0	0.01
0	0	0	0	0	0	0	0	0	0	0	0	0	0	0	0	0	0	0	0	0
0	0	0	0	0	0	0	0	0	0	0	0	0	0	0	0	0	0	0	0	0
0	0	0	0	0	0	0	0	0	0	0	0	0	0	0	0	0	0	0	0	0
0	0	0	0	0	0	0	0	0	0	0	0	0	0	0	0	0	0	0	0	0
0	0	0	0	0	0	0	0	0	0	0	0	0	0	0	0	0	0	0	0	0
0	0	0	0	0	0	0	0	0	0	0	0	0	0	0	0	0	0	0	0	0
0	0	0	0	0	0	0	0	0	0	0	0	0	0	0	0	0	0	0	0	0
0	0	0	0	0	0	0	0	0	0	0	0	0	0	0	0	0	0	0	0	0.01
0	0	0	0	0	0	0	0	0	0	0	0	0	0	0	0	0	0	0	0	0
0	0	0	0	0	0	0	0	0	0	0	0	0	0	0	0	0	0	0	0	0
0	0	0	0	0	0	0	0	0	0	0	0	0	0	0	0	0	0	0	0	0
0	0	0	0	0	0	0	0	0	0	0	0	0	0	0	0	0	0	0	0	0
0	0	0	0	0	0	0	0	0	0	0	0	0	0	0	0	0	0	0	0	0
0	0	0	0	0	0	0	0	0	0	0	0	0	0	0	0	0	0	0.01	0	0
0	0	0	0	0	0	0	0	0	0	0	0	0	0	0	0	0	0	0	0	0
0	0	0	0	0	0	0	0	0	0	0	0	0	0	0	0	0	0	0	0	0
0	0	0	0	0	0	0	0	0	0	0	0	0	0	0	0	0	0	0	0	0
0	0	0	0	0	0	0	0	0	0	0	0	0	0	0	0	0	0	0	0	0
0	0	0	0	0	0	0	0	0	0	0	0	0	0	0	0	0	0	0	0	0
0	0	0	0	0	0	0	0	0	0	0	0	0	0	0	0	0	0	0	0	0

0	0	0	0	0	0	0	0	0	0	0	0	0	0	0	0	0	0	0	0	0
0	0	0	0	0	0	0	0	0	0	0	0	0	0	0	0	0	0	0	0	0
0	0	0	0	0	0	0	0	0	0	0	0	0	0	0	0	0	0	0	0	0
0	0	0	0	0	0	0	0	0	0	0	0	0	0	0	0	0	0	0	0	0
0.01	0	0	0	0	0	0	0	0	0	0	0	0	0	0.14	0	0	0.09	0.21	0.05	0.1
0.07	0.02	0.05	0	0.01	0.01	0.01	0.01	0.02	0.04	0.03	0.01	0.01	0.01	0	0.01	0	0	0.01	0.02	0.01
0	0	0	0	0	0	0	0	0	0	0	0	0	0	0	0	0	0	0	0	0
0.03	0.02	0	0	0	0	0.01	0.01	0	0	0	0	0	0.01	0	0.01	0	0	0.01	0.01	0.02
0	0	0	0	0	0	0	0	0	0	0	0	0	0	0	0	0	0	0	0	0
0.01	0	0	0	0	0.01	0	0	0	0	0	0	0	0	0	0	0	0	0	0	0
0	0	0	0	0	0	0.01	0.01	0	0	0	0	0	0	0	0	0	0	0	0	0
0.01	0	0	0.01	0	0	0	0	0	0	0.03	0	0	0	0.04	0	0	0.01	0.05	0.02	0.03
0.01	0	0.01	0	0.01	0.01	0.01	0.01	0	0	0	0	0	0.02	0.02	0	0	0.01	0.04	0.01	0.03

miR_N	miR_O	miR_O	miR_O	miR_O	miR_Pa	miR_Pi	miR_Pl	miR_Pl	miR_P	miR_P	miR_Pr	miR_re	miR_re	miR_re	miR_S	miR_S	miR_Sp	miR_T-	miR_T-	miR_T-
0.02	0.13	0.03	0.04	0.08	0.03	0.03	0.01	0	0.04	0.04	0.03	0.03	0.12	0.02	0	0	0.05	0.04	0.02	0.09
0.01	0.03	0	0.01	0.1	0.04	0.03	0	0	0.01	0.02	0.07	0.06	0.14	0.05	0	0	0.1	0.02	0	0.04
0	0	0	0	0.02	0	0.01	0	0	0	0	0.05	0	0.01	0	0	0	0.02	0	0	0
0	0.02	0	0	0	0	0	0	0	0	0	0	0	0	0	0	0	0.01	0.01	0	0
0	0.02	0.01	0.01	0	0.01	0	0	0	0	0	0	0	0.01	0	0	0	0	0	0	0
0.01	0.02	0.04	0.04	0	0.01	0	0	0	0.04	0.08	0.01	0.01	0.04	0	0	0	0	0.02	0.03	0.03
0	0	0	0	0	0	0	0	0	0	0	0.01	0	0	0	0	0	0	0	0.01	0.01
0.01	0	0	0.01	0	0.01	0	0	0	0.01	0.01	0	0	0.01	0	0	0	0	0	0	0
0	0	0	0	0	0	0	0	0	0	0	0	0	0	0	0	0	0	0	0	0
0	0	0.01	0.01	0.02	0	0	0.01	0	0	0.02	0	0	0	0	0	0	0.01	0	0	0
0	0	0	0	0	0	0	0	0	0	0	0	0	0	0	0	0	0	0	0	0
0	0	0	0	0	0	0	0	0	0	0.02	0	0	0	0	0.01	0	0	0	0	0
0.01	0	0	0	0	0	0	0	0	0	0	0	0	0	0	0	0	0	0	0.01	0
0	0	0	0	0	0	0	0	0	0	0	0	0	0	0	0	0.01	0	0	0	0
0	0	0	0	0	0	0	0	0	0	0	0	0	0	0	0	0	0	0	0	0
0	0	0	0	0	0	0	0	0	0	0	0	0	0	0	0	0	0	0	0	0
0	0	0	0.01	0	0	0	0	0	0	0	0	0	0	0	0.01	0.01	0	0.01	0.01	0
0	0	0	0	0	0	0	0	0	0	0	0	0	0	0	0	0	0	0	0	0
0	0	0	0	0	0	0	0	0	0.01	0.01	0	0	0	0	0	0	0	0	0	0.02
0	0	0	0	0	0	0	0	0	0	0	0	0	0	0	0	0	0	0	0	0
0	0	0	0	0	0	0	0	0	0	0	0	0	0	0	0	0	0	0	0	0
0	0	0	0	0	0	0	0	0	0	0	0	0	0	0	0	0	0	0	0	0
0	0	0	0	0	0	0	0	0	0	0	0	0	0	0	0	0	0	0	0	0
0	0	0	0	0.01	0.01	0.01	0	0	0	0	0	0	0	0	0	0	0.01	0	0	0
0	0	0	0	0.12	0	0.04	0.01	0	0	0.01	0.02	0.02	0.02	0.01	0	0	0.03	0	0	0
0	0	0	0	0.05	0.04	0.05	0.15	0	0	0	0.02	0	0	0	0	0	0.1	0	0.01	0
0	0	0	0	0	0	0	0	0	0	0	0	0	0	0	0	0	0	0	0	0
0	0	0	0	0	0	0	0	0	0	0	0	0	0	0	0	0	0	0	0	0
0	0	0	0	0	0	0	0	0	0	0	0	0	0	0	0	0.01	0	0	0.01	0
0	0	0	0	0	0	0	0	0	0	0	0	0	0	0	0	0.01	0	0	0.01	0
0	0	0	0	0	0	0	0	0	0	0	0	0	0	0	0	0	0	0	0	0
0	0	0	0	0	0	0	0	0	0	0	0	0	0	0	0	0	0	0	0	0
0	0	0	0	0	0	0	0	0	0	0	0	0	0	0	0	0	0	0	0	0
0	0.03	0.01	0.01	0.01	0	0	0.01	0	0	0.01	0	0.01	0.01	0.02	0.01	0.02	0	0	0.02	0
0	0.03	0	0	0	0	0	0	0	0	0	0	0	0	0	0	0	0	0	0	0

0	0	0	0	0	0	0.01	0	0	0	0	0	0	0	0	0	0	0	0	0	0
0	0	0	0	0	0	0	0	0	0	0	0	0	0	0	0	0	0	0	0	0
0	0	0	0	0	0	0	0	0	0	0	0	0	0	0	0	0	0	0	0	0
0	0	0	0	0	0	0	0	0	0	0	0	0.01	0	0.01	0	0	0	0	0	0
0	0	0	0	0	0	0	0	0	0	0	0	0	0	0	0	0.01	0	0	0	0
0	0	0.01	0	0	0	0	0	0	0	0	0	0	0	0	0	0	0	0	0	0
0	0	0	0	0	0	0	0	0	0.01	0	0	0.01	0	0.01	0	0	0	0	0	0
0	0	0	0	0	0	0	0	0	0	0	0	0	0	0	0	0	0	0	0	0
0	0	0	0	0	0	0	0	0	0	0	0	0	0	0	0.01	0	0	0	0	0
0	0	0	0	0	0	0	0	0	0	0	0	0	0	0	0	0	0	0	0	0
0	0	0	0	0	0	0	0	0	0	0	0	0	0	0	0	0	0	0	0	0
0	0	0	0	0.01	0	0	0	0	0	0	0	0	0	0	0	0	0.01	0	0	0
0.01	0	0.02	0.02	0.01	0	0	0.01	0	0	0.01	0	0.01	0.01	0.03	0	0.01	0.01	0.01	0.01	0
0	0	0	0	0	0.06	0.01	0.01	0	0	0	0	0	0	0	0	0	0	0	0	0
0.13	0	0	0	0	0	0	0	0.27	0	0.01	0	0	0	0	0	0	0	0.1	0.13	0.14
0.39	0	0	0	0	0	0	0	0.13	0	0	0	0	0	0	0	0	0	0.22	0.22	0.16
0	0.03	0.01	0	0.14	0.03	0.02	0.11	0	0	0	0.29	0.07	0.03	0.11	0	0	0.12	0	0	0
0	0	0	0	0	0	0	0	0	0	0	0	0	0	0	0	0	0	0	0	0
0	0	0	0	0.01	0	0	0	0	0	0	0.05	0	0	0	0	0	0.01	0	0	0
0	0	0	0	0	0	0	0	0	0.19	0.03	0	0	0	0	0	0	0.02	0	0.02	0.01
0	0	0	0	0	0	0	0	0	0	0	0	0	0	0	0	0	0	0	0	0
0	0	0	0	0	0	0	0	0	0	0	0	0	0	0	0	0	0	0	0	0
0	0	0	0	0	0	0	0	0	0	0	0	0	0	0	0	0	0	0	0	0
0	0	0	0	0	0	0	0	0	0	0	0	0	0	0	0	0	0	0	0	0
0	0	0	0	0	0	0	0	0.04	0	0	0	0	0	0	0	0	0	0	0	0
0	0	0	0	0	0	0	0	0	0	0	0	0	0	0	0	0	0	0	0	0
0	0	0	0	0	0	0	0	0	0	0	0	0	0	0	0	0.01	0	0	0	0
0.05	0	0	0	0	0	0	0	0	0	0	0	0	0	0	0	0	0.01	0	0.01	0
0	0	0	0.01	0	0	0	0	0	0	0	0	0	0	0	0	0.01	0	0	0	0
0	0	0.01	0.01	0	0.01	0	0	0	0	0.01	0	0	0.01	0	0	0	0	0	0	0
0	0	0	0	0	0	0	0	0	0	0	0	0	0	0	0	0	0	0	0	0
0	0	0	0	0	0	0	0	0	0	0	0	0	0	0	0	0.01	0	0	0	0
0	0	0	0	0	0	0	0	0	0	0	0	0	0	0	0	0	0	0	0	0
0	0	0	0	0	0	0	0	0	0	0	0	0	0	0	0	0	0	0	0.01	0
0.02	0	0.02	0.01	0	0.02	0.02	0	0.01	0.01	0.03	0	0.02	0.02	0.02	0.02	0.02	0	0.1	0.08	0.09
0	0.02	0.01	0.01	0	0	0	0	0.03	0	0	0	0	0	0	0	0.01	0	0	0	0

0.06	0.1	0.11	0.1	0.05	0.07	0.12	0.04	0.11	0.04	0.04	0.06	0.1	0.07	0.02	0.04	0.04	0.03	0.21	0.11	0.12
0	0	0.01	0.01	0	0	0	0	0	0	0	0	0	0	0	0.01	0.01	0	0.01	0.01	0.01
0.01	0	0	0	0	0	0.01	0	0	0.01	0.01	0	0	0	0.01	0	0	0.01	0.01	0.05	0.05
0	0	0	0	0	0	0	0	0	0	0	0	0	0	0	0	0	0	0.01	0.01	0.02
0	0	0	0	0	0	0	0	0	0	0	0	0	0	0	0	0	0	0	0	0
0	0	0	0	0	0	0	0	0	0	0	0	0	0	0	0	0	0	0	0	0
0	0	0	0	0	0	0.01	0	0	0.01	0	0	0	0	0	0.01	0.01	0	0	0	0
0	0	0	0	0	0	0	0	0	0	0	0	0	0	0	0	0	0	0	0	0
0	0	0	0	0	0	0	0	0	0	0	0	0	0	0	0	0	0	0	0	0
0	0	0	0	0	0	0	0	0	0	0	0	0	0	0	0	0	0	0	0	0
0	0.02	0.01	0.01	0	0	0	0	0	0	0	0	0	0.01	0.01	0	0.01	0	0	0	0
0	0	0	0	0	0	0	0	0	0	0	0	0	0	0	0	0	0	0	0	0
0	0	0	0	0	0	0	0	0	0	0	0	0	0	0	0	0	0	0	0	0
0	0	0	0	0	0	0	0	0	0	0	0	0	0	0	0	0	0	0	0	0
0	0	0	0.01	0	0	0	0	0	0	0	0	0	0	0	0.06	0.01	0	0.01	0	0
0	0	0	0	0	0	0	0	0	0	0	0	0	0	0	0.01	0.01	0	0	0	0
0	0	0	0	0	0	0	0	0	0	0	0	0	0	0	0	0	0	0	0	0
0	0	0	0	0	0	0	0	0	0	0	0	0	0	0	0	0	0	0	0	0
0	0	0	0	0	0.01	0	0	0.01	0.01	0.01	0	0.01	0.02	0.01	0.04	0	0.01	0.03	0	0
0	0	0	0	0	0	0	0	0	0	0	0	0	0	0	0	0	0	0	0	0
0	0	0	0	0	0	0	0	0	0	0	0	0	0	0	0	0	0	0	0	0
0	0	0	0	0	0	0	0	0	0	0	0	0	0	0	0	0	0	0	0	0
0	0	0	0	0	0	0	0	0	0	0	0	0	0	0	0	0	0	0	0	0
0	0	0	0	0	0	0	0	0	0	0	0	0	0	0	0	0	0	0	0	0
0	0	0	0	0.01	0	0.01	0	0	0	0	0.01	0	0	0	0	0	0	0	0	0
0	0	0.01	0.01	0	0	0	0	0	0	0	0	0	0	0	0	0	0	0	0	0
0	0	0	0	0	0	0	0	0	0	0.01	0	0	0	0	0	0	0	0	0.02	0.01
0	0.02	0	0	0	0	0	0	0	0	0	0	0	0	0	0	0	0	0	0	0
0	0.02	0	0	0	0	0	0	0	0	0	0	0	0	0	0	0	0	0	0	0
0	0	0	0	0.01	0	0	0	0	0	0	0.01	0	0	0	0	0	0	0	0	0
0	0	0	0	0	0	0	0	0	0	0	0	0	0	0	0	0	0	0	0	0
0	0	0	0	0	0	0	0	0	0	0	0	0	0	0	0	0	0	0	0	0
0	0	0	0	0	0	0	0	0	0	0	0	0	0	0.01	0	0	0.01	0	0	0
0	0	0.01	0.01	0	0	0	0	0	0	0.01	0	0	0	0	0.01	0.02	0	0.01	0.01	0.01
0	0	0.01	0.01	0	0	0	0	0	0	0.01	0	0	0	0	0.01	0.05	0	0	0.01	0
0	0	0	0	0	0.02	0	0	0	0	0.01	0	0	0	0	0	0	0	0	0	0

0	0	0	0	0	0.01	0	0	0	0	0.01	0	0	0	0	0	0	0	0	0	0
0	0	0	0	0	0.04	0.03	0.02	0	0	0	0.02	0	0	0	0	0	0.03	0	0	0
0	0	0	0	0	0	0	0	0	0	0	0	0	0	0	0	0	0	0	0	0
0	0	0	0	0	0	0	0	0	0	0	0	0	0	0	0	0	0	0	0	0
0	0	0	0	0	0	0	0	0	0	0	0	0	0	0	0	0	0	0	0	0
0	0	0	0	0	0	0	0	0	0	0	0.01	0	0	0	0	0	0.02	0	0	0
0	0	0	0	0	0	0	0	0	0	0	0	0	0	0	0	0	0	0	0	0
0	0	0	0	0	0	0	0	0	0	0	0	0	0	0	0	0	0	0	0	0
0	0	0	0	0	0	0	0	0	0	0	0	0	0	0	0	0	0	0	0	0
0	0	0.01	0.01	0	0	0	0	0.02	0	0	0	0	0	0	0.01	0	0	0.01	0.01	0.01
0	0	0	0	0	0	0	0	0	0	0	0	0	0	0	0.01	0.01	0	0	0	0
0.02	0.18	0.22	0.21	0	0.03	0	0.01	0.12	0.16	0.15	0.02	0.06	0.04	0.05	0	0.01	0.01	0	0.02	0.02
0	0	0	0	0	0	0	0	0	0	0	0	0	0	0.01	0	0	0	0	0	0
0	0	0	0	0	0	0	0	0	0	0	0	0	0	0	0	0	0	0	0	0
0	0	0	0	0	0	0	0	0	0	0	0	0	0	0	0	0	0	0	0	0
0	0	0	0	0.01	0	0	0	0	0	0	0.01	0	0	0	0	0	0.01	0	0	0
0	0	0	0	0	0	0	0	0	0	0	0	0	0	0	0	0	0	0	0	0
0	0	0	0	0	0	0	0	0	0	0	0	0	0	0	0	0	0	0	0	0
0	0	0	0	0	0	0	0	0	0	0	0	0	0	0	0	0	0	0	0	0
0	0	0	0	0	0	0	0	0	0	0	0	0	0	0	0	0	0	0	0	0
0	0	0	0	0	0	0	0	0	0	0	0	0	0	0	0	0	0	0	0	0
0	0	0	0	0	0	0	0	0	0	0	0	0	0	0	0	0	0	0	0	0
0	0	0	0	0	0	0	0	0	0	0	0	0	0	0	0	0	0	0	0	0
0	0.02	0.01	0.01	0.01	0.01	0.02	0.02	0	0.04	0.04	0.02	0.05	0.03	0.09	0	0.01	0.02	0	0	0
0	0	0	0	0	0	0	0	0	0	0	0	0	0	0	0	0	0	0	0	0
0	0	0	0	0	0	0	0	0	0	0	0	0	0	0	0	0	0	0	0	0
0	0	0	0	0	0	0	0	0	0	0	0	0	0	0	0	0	0	0	0	0
0	0	0.01	0.02	0	0	0	0	0	0.01	0.01	0	0	0	0	0	0.01	0	0	0	0
0	0	0	0	0	0	0	0	0	0.01	0	0.01	0	0	0	0.01	0	0	0	0	0
0	0	0	0	0	0	0	0	0	0	0	0	0	0	0	0	0	0	0	0	0
0	0	0	0	0	0	0	0	0	0	0	0	0	0	0	0	0	0	0	0	0
0	0.02	0.02	0.01	0.01	0.01	0	0.01	0	0.01	0.01	0.01	0	0.01	0.01	0	0	0.02	0	0	0
0	0.02	0	0	0	0.01	0.01	0	0	0.01	0.01	0.02	0	0	0	0	0	0.01	0	0	0
0	0.05	0.02	0.03	0.01	0.03	0.02	0.05	0	0.02	0.03	0.01	0.02	0.02	0.01	0	0	0.03	0	0	0
0	0	0.01	0.01	0	0	0	0	0	0	0	0	0	0	0	0	0	0	0.01	0	0
0.01	0.1	0.01	0.01	0.03	0.03	0.03	0.01	0	0.01	0.01	0.03	0	0.01	0	0	0.01	0.03	0	0.01	0.02

0.01	0	0.01	0	0.01	0	0.01	0	0	0	0.01	0	0	0.01	0	0	0.01	0.01	0	0	0.01
0.01	0.03	0.05	0.04	0.01	0.02	0	0.03	0.02	0.03	0.02	0.01	0.1	0.05	0.07	0	0	0	0	0	0
0	0.02	0.01	0	0.01	0.03	0.01	0.01	0	0.01	0	0.02	0.01	0.01	0.01	0	0	0.02	0	0	0
0	0	0	0	0	0	0	0	0	0.01	0.01	0	0	0	0	0	0.01	0	0	0	0
0	0	0.01	0.01	0	0	0	0	0	0.02	0.02	0	0.01	0.01	0.02	0	0	0	0	0	0
0	0	0	0	0	0	0	0	0	0	0	0	0	0	0	0.01	0	0	0	0	0
0	0	0	0	0	0	0	0	0	0	0	0	0	0	0	0	0	0	0	0	0
0	0	0	0	0	0	0	0	0	0	0	0	0	0	0	0	0	0	0	0	0
0	0	0	0	0	0	0	0	0	0	0	0	0	0	0	0	0	0	0	0	0
0	0	0	0	0	0	0	0	0	0	0	0	0	0	0	0	0	0	0	0	0
0.02	0	0.02	0.02	0.02	0.01	0.02	0	0.02	0.01	0.02	0.03	0.01	0.01	0.01	0	0	0.01	0	0	0
0.09	0	0.02	0.02	0	0.05	0.05	0.01	0.02	0.01	0.03	0	0.07	0.02	0.04	0	0	0	0.04	0.01	0
0.02	0	0	0	0	0.01	0.01	0	0	0	0	0	0	0	0	0	0	0	0.01	0	0
0	0	0	0	0	0	0	0	0	0	0	0	0	0	0	0	0	0	0	0	0
0	0	0.01	0	0	0	0	0	0	0	0	0	0	0	0	0	0	0	0	0	0
0	0	0	0	0	0	0	0	0	0	0	0	0	0	0	0	0	0	0.01	0	0
0	0	0	0	0	0	0	0	0	0	0	0	0	0	0	0.21	0.13	0	0	0	0
0	0	0	0	0	0	0	0	0	0	0	0	0	0	0	0.15	0.05	0	0	0	0
0	0	0	0	0	0	0	0	0	0	0	0	0	0	0	0.05	0.14	0	0	0	0
0	0	0	0	0	0	0	0	0	0	0	0	0	0	0	0.04	0.04	0	0	0	0
0	0	0	0	0	0.02	0	0.02	0	0.04	0.02	0.01	0	0	0	0	0	0	0	0	0
0	0	0.01	0.01	0	0	0	0	0	0	0	0	0	0	0	0	0	0	0	0	0
0	0.02	0	0.01	0	0.01	0	0	0	0.01	0.01	0.01	0	0	0	0	0.01	0	0	0	0
0	0	0.01	0.01	0.01	0.02	0.03	0.06	0	0.01	0.01	0.01	0	0	0	0	0.01	0.01	0	0.01	0
0.02	0	0.01	0.01	0	0.01	0	0	0	0.01	0.01	0	0	0	0	0	0.02	0	0	0.03	0.02
0	0	0.01	0	0	0	0	0	0	0.01	0	0	0	0	0	0	0	0	0	0	0
0.01	0	0	0	0	0	0	0	0.02	0	0.01	0	0	0	0.01	0	0	0	0.02	0.01	0.01
0	0	0.01	0.01	0.01	0	0	0	0	0	0	0	0	0	0	0	0	0.01	0	0	0
0	0	0	0	0	0	0	0	0	0	0	0	0	0	0	0	0	0	0	0	0
0	0	0	0	0	0	0	0	0	0	0	0	0	0	0	0	0	0	0	0	0
0	0	0	0	0	0	0	0	0	0	0	0	0	0	0	0.04	0	0	0	0	0
0	0	0	0	0	0	0	0	0	0	0	0	0	0	0	0	0	0	0	0	0
0	0	0	0	0	0	0	0	0	0	0	0	0	0	0	0	0	0	0	0	0
0	0	0	0	0	0	0	0	0	0	0	0	0	0	0	0	0	0	0	0	0
0	0	0	0	0	0	0	0	0	0	0	0	0	0	0	0	0.01	0	0	0	0
0	0	0	0	0	0	0	0	0	0	0	0	0	0	0	0	0	0	0	0	0

0	0	0	0	0	0	0	0	0	0	0	0	0	0	0	0	0	0	0	0	0
0	0	0	0	0	0	0	0	0	0	0	0	0	0	0	0	0	0	0	0	0
0	0	0	0	0	0	0	0	0	0	0	0	0	0	0	0	0	0	0	0	0
0	0	0	0	0	0	0	0	0	0	0	0	0	0	0	0	0	0	0	0	0
0	0	0	0	0	0	0	0	0	0	0	0	0	0	0	0	0	0	0	0	0
0	0	0	0	0	0	0	0	0	0	0	0	0	0	0	0	0	0	0	0	0
0	0	0	0	0	0	0	0	0	0	0	0	0	0	0	0	0	0	0	0	0
0	0	0	0	0	0	0	0	0	0	0	0	0	0	0	0	0	0	0	0	0
0	0	0	0	0	0	0	0	0	0	0	0	0	0	0	0	0	0	0	0	0
0	0	0	0	0	0	0	0	0	0	0	0	0	0	0	0	0	0	0	0	0
0	0	0	0	0	0	0	0	0	0	0	0	0	0	0	0	0	0	0	0	0
0	0	0	0	0	0	0	0	0	0	0	0	0	0	0	0	0	0	0	0	0
0	0	0	0	0	0	0	0	0	0	0	0	0	0	0	0.01	0	0	0	0	0
0	0	0	0	0	0	0	0	0	0	0	0	0	0	0	0	0	0	0	0	0
0	0	0	0	0	0	0	0	0	0	0	0	0	0	0	0	0	0	0	0	0
0	0	0	0	0	0	0	0	0	0	0	0	0	0	0	0	0	0	0	0	0
0	0	0	0	0	0	0	0	0	0	0	0	0	0	0	0	0	0	0	0	0
0	0	0	0	0	0	0	0	0	0	0	0	0	0	0	0	0	0	0	0	0
0	0	0	0	0	0	0	0	0	0	0	0	0	0	0	0	0	0	0	0	0
0	0	0	0.01	0	0	0	0	0	0	0	0	0	0	0	0	0	0	0	0	0
0	0	0	0	0	0	0	0	0	0	0	0	0	0	0	0	0	0	0	0	0
0	0	0	0	0	0	0	0	0	0	0	0	0	0	0	0	0	0.01	0	0	0
0	0	0	0	0	0	0	0	0	0	0	0	0	0	0	0	0	0	0	0	0
0	0	0	0	0	0	0	0	0	0	0	0	0	0	0	0	0	0	0	0	0
0	0	0	0	0	0	0	0	0	0	0	0	0	0	0	0	0	0	0	0	0
0	0	0	0	0	0	0	0	0	0	0	0	0	0	0	0	0	0	0	0	0
0	0	0	0	0	0	0	0	0	0	0	0	0	0	0	0	0	0	0	0	0
0	0	0	0	0	0	0	0	0	0	0	0	0	0	0	0	0.01	0	0	0	0
0	0	0	0	0	0	0	0	0	0	0	0	0	0	0	0	0	0	0	0	0
0	0	0	0	0	0	0	0	0	0	0	0	0	0	0	0.1	0.04	0	0	0	0
0	0	0	0	0	0.01	0.01	0.01	0	0.01	0	0	0.01	0.03	0.03	0	0	0	0	0	0
0	0	0	0	0	0	0	0	0	0	0	0	0	0	0	0	0	0	0	0	0
0	0	0	0	0	0	0	0	0	0	0	0	0	0	0	0	0	0	0	0	0
0	0	0	0	0	0	0	0	0	0	0	0	0	0	0	0	0	0	0	0	0
0	0	0	0	0	0	0	0	0	0	0	0	0	0	0	0	0	0	0	0	0
0	0	0	0	0	0	0	0	0	0	0	0	0	0	0	0	0	0	0	0	0

0	0	0	0	0	0	0	0	0	0	0	0	0	0	0	0.01	0.01	0	0	0	0
0	0	0	0	0	0	0	0	0	0	0	0	0	0	0	0.02	0.02	0	0	0	0
0	0	0	0	0	0	0	0	0	0	0	0	0.01	0.01	0	0	0	0	0	0	0
0	0	0	0	0	0	0	0	0	0	0.01	0	0	0	0	0.01	0	0	0	0	0
0	0	0	0	0	0.08	0.03	0	0	0	0	0	0	0	0	0	0	0	0	0	0
0	0	0	0	0	0	0	0	0	0	0	0	0	0.01	0	0	0	0	0	0	0
0	0	0	0	0	0	0	0	0	0	0	0	0	0	0	0	0	0	0	0	0
0	0	0	0	0	0	0	0	0	0	0	0	0	0.01	0.01	0	0	0	0	0	0
0	0	0	0	0	0	0	0	0	0	0	0	0	0	0	0	0.01	0	0	0	0
0	0	0	0	0	0	0	0	0	0	0	0	0	0	0	0	0	0	0	0	0
0	0	0	0	0	0	0	0	0	0	0	0	0	0	0	0	0	0	0	0	0
0	0	0	0	0	0	0	0	0	0	0	0	0	0	0	0	0	0	0	0	0
0	0	0	0	0	0	0	0	0	0	0	0	0	0	0	0	0	0	0	0	0
0	0	0	0	0	0	0	0	0	0	0	0	0	0	0	0	0	0	0	0	0
0	0	0	0	0	0	0	0	0	0	0	0	0	0	0	0	0	0	0	0	0
0	0	0	0	0	0	0	0	0	0	0	0	0	0	0	0	0	0	0	0	0
0	0	0	0	0	0	0	0	0	0	0	0	0	0	0	0	0	0	0	0	0
0	0	0	0	0	0	0	0	0	0	0	0	0	0	0	0	0	0	0	0	0
0	0	0	0	0	0	0	0	0	0	0	0	0	0	0	0	0	0	0	0	0
0	0	0	0	0	0	0	0	0	0	0	0	0	0	0	0	0	0	0	0	0
0	0	0	0	0	0	0	0	0	0	0	0	0	0	0	0	0	0	0	0	0
0	0	0	0	0	0	0	0	0	0	0	0	0	0	0	0	0	0	0	0	0
0	0	0	0	0	0	0	0	0	0	0	0	0	0	0	0	0	0	0	0	0
0	0	0	0	0	0	0	0	0	0.01	0	0	0	0	0	0.01	0	0	0	0	0
0	0.02	0	0	0	0	0	0	0	0	0.01	0	0	0	0	0	0	0.01	0	0	0
0	0	0	0	0.02	0	0.02	0.08	0	0	0.01	0	0.12	0.09	0.1	0	0	0	0	0	0
0	0	0	0	0	0	0	0	0.01	0	0	0	0	0	0	0	0	0	0	0	0
0	0	0	0	0	0	0	0	0	0	0	0	0	0	0	0	0	0	0	0	0
0	0	0	0	0	0	0	0	0	0	0	0	0	0	0	0	0	0	0	0	0
0	0	0	0	0	0	0	0	0	0	0	0	0	0	0	0	0	0	0	0	0
0	0	0	0	0	0	0	0	0	0	0	0	0	0	0	0	0	0	0	0	0
0	0	0	0	0	0	0	0	0	0	0	0	0	0	0	0	0	0	0	0	0
0	0	0	0	0	0	0	0	0	0	0	0	0	0	0	0	0	0	0	0	0
0	0	0	0	0	0	0	0	0	0	0	0	0	0	0	0	0	0	0	0	0
0	0	0	0	0	0	0	0	0	0	0	0	0	0	0	0	0	0	0	0	0
0	0	0	0	0	0	0	0	0	0	0	0	0	0	0	0	0	0	0	0	0

0	0	0	0	0	0	0	0	0	0	0	0	0	0	0	0	0	0	0	0	0
0	0	0	0	0.01	0	0.01	0.01	0	0	0	0	0	0	0	0	0	0	0	0	0
0	0	0	0	0	0	0	0	0	0	0	0	0	0	0	0	0	0	0	0	0
0	0	0	0	0	0	0	0	0	0	0	0	0	0	0	0	0	0	0	0	0
0	0	0.01	0	0	0	0	0	0	0	0	0	0	0	0	0	0	0	0	0	0
0	0	0	0	0	0	0	0	0	0	0	0	0	0	0	0	0	0	0	0	0
0	0	0	0	0	0	0	0	0	0	0	0	0	0	0	0	0	0	0	0	0
0	0	0	0	0	0	0	0	0	0	0	0	0	0	0	0	0	0	0	0	0
0	0	0	0	0	0	0	0	0	0	0	0	0	0	0	0	0	0	0	0	0
0	0	0	0	0	0	0	0	0	0	0	0	0	0	0	0	0	0	0	0	0
0	0	0	0	0	0	0	0	0	0	0	0	0	0	0	0	0	0	0	0	0
0	0	0	0	0	0	0	0	0	0	0	0	0	0	0	0	0	0	0	0	0
0	0	0	0	0	0	0	0	0	0	0	0	0	0	0	0	0	0	0	0	0
0	0.02	0	0	0	0	0	0	0	0	0	0	0	0	0	0	0	0	0	0	0
0	0	0	0	0	0	0	0	0	0	0	0	0	0	0	0	0	0	0	0	0
0	0	0	0	0	0	0	0	0	0	0	0	0	0	0	0	0	0	0	0	0
0	0	0	0	0	0	0	0	0	0	0	0	0	0	0	0	0.01	0	0	0	0
0	0	0	0	0	0	0	0	0	0	0	0	0	0	0	0	0	0	0	0	0
0	0	0	0	0	0	0	0	0	0	0	0	0	0	0	0	0	0	0	0	0
0	0	0	0	0	0	0	0	0	0	0	0	0	0	0	0	0	0	0	0	0
0	0	0	0	0	0	0	0	0	0	0	0	0	0	0	0	0	0	0	0	0
0	0	0	0	0	0	0	0	0	0	0	0	0	0	0	0	0	0	0	0	0
0	0	0	0	0	0	0	0	0	0	0	0	0	0	0	0	0	0	0	0	0
0	0	0	0	0	0	0	0	0	0	0	0	0	0	0	0	0	0	0	0	0
0	0	0	0	0	0	0	0	0	0	0	0	0	0	0	0	0	0	0	0	0
0	0	0	0	0	0	0	0	0	0	0	0	0	0	0	0	0	0	0	0	0
0	0	0	0	0	0	0	0	0	0	0	0	0	0	0	0	0	0	0	0	0
0	0	0	0	0	0	0	0	0	0	0	0	0	0	0	0	0	0	0	0	0
0	0	0	0	0	0	0	0	0	0	0	0	0	0	0	0	0	0	0	0	0
0	0	0	0	0	0	0	0	0	0	0	0	0	0	0	0	0	0	0	0	0
0	0	0	0	0	0	0	0	0	0	0	0	0	0	0	0	0	0	0	0	0
0	0	0	0	0	0	0	0	0	0	0	0	0	0	0	0	0	0	0	0	0
0	0	0	0	0	0	0	0	0	0	0	0	0	0	0	0	0	0	0	0	0
0	0	0	0	0	0	0	0	0	0	0	0	0	0	0	0	0	0	0	0	0
0	0	0	0	0	0	0	0	0	0	0	0	0.01	0	0.01	0	0	0	0	0	0
0	0	0	0	0	0	0	0	0	0	0	0	0	0	0	0	0	0	0	0	0

0	0	0	0	0	0	0	0	0	0	0	0	0	0	0	0	0	0	0	0	0
0	0	0	0	0	0	0	0	0	0	0	0	0	0	0	0	0	0	0	0	0
0	0	0	0	0	0	0	0	0	0	0	0	0	0	0	0	0	0	0	0	0
0	0	0	0	0	0	0	0	0	0	0	0	0	0	0	0	0	0	0	0	0
0	0	0	0	0	0	0	0	0	0	0	0	0	0	0	0	0	0	0	0	0
0	0	0	0	0	0	0	0	0	0	0	0	0	0	0	0	0	0	0	0	0
0	0	0	0	0	0	0	0	0	0	0	0	0	0	0	0	0	0	0	0	0
0	0	0	0	0	0	0	0	0	0	0	0	0	0	0	0	0	0	0	0	0
0	0	0	0	0	0	0	0	0	0	0	0	0	0	0	0	0	0	0	0	0
0	0	0	0	0	0	0	0	0	0	0	0	0	0	0	0	0	0	0	0	0
0	0	0	0	0	0	0	0	0	0	0	0	0	0	0	0	0	0	0	0	0
0	0	0	0	0	0	0	0	0	0	0	0	0	0	0	0	0	0	0	0	0
0	0	0	0	0	0	0	0	0	0	0	0	0	0	0	0	0	0	0	0	0
0	0	0	0	0	0	0	0	0	0	0	0	0	0	0	0	0	0	0	0	0
0	0	0	0	0	0	0	0.01	0	0	0	0	0	0	0	0	0	0	0	0	0
0	0	0	0	0	0	0	0.01	0	0	0	0	0	0	0	0	0	0	0	0	0
0	0	0	0	0	0	0	0.02	0	0	0	0	0	0	0	0	0	0	0	0	0
0	0	0	0	0	0	0	0.03	0	0	0	0	0	0	0	0	0	0	0	0	0
0	0	0	0	0	0	0	0.01	0	0	0	0	0	0	0	0	0	0	0	0	0
0	0	0	0	0	0	0	0	0	0	0	0	0	0	0	0	0	0	0	0	0
0	0	0	0	0	0	0	0	0	0	0	0	0	0	0	0	0	0	0	0	0
0	0	0	0	0	0	0	0.03	0	0	0	0	0	0	0	0	0	0	0	0	0
0	0	0	0	0	0	0	0.01	0	0	0	0	0	0	0	0	0.01	0	0	0	0
0	0	0	0	0	0	0	0	0	0	0	0	0	0	0	0	0	0	0	0	0
0	0	0	0	0	0	0	0.01	0	0	0	0	0	0	0	0	0	0	0	0	0
0	0	0	0	0	0	0	0.01	0	0	0	0	0	0	0	0	0	0	0	0	0
0	0	0	0	0	0	0	0	0	0	0	0	0	0	0	0	0	0	0	0	0
0	0	0	0	0	0	0	0	0	0	0	0	0	0	0	0	0	0	0	0	0
0	0	0	0	0	0	0	0	0	0	0	0	0	0	0	0	0	0	0	0	0
0	0	0	0	0	0	0	0	0	0	0	0	0	0	0	0	0	0	0	0	0
0	0	0	0	0	0	0	0.01	0	0	0	0	0	0	0	0	0	0	0	0	0
0	0	0	0	0	0	0	0	0	0	0	0	0	0	0	0	0	0	0	0	0
0	0	0	0	0	0	0	0	0	0	0	0	0	0	0	0	0	0	0	0	0
0	0	0	0	0	0	0	0	0	0	0	0	0	0	0	0	0	0	0	0	0
0	0	0	0	0	0	0	0	0	0	0	0	0	0	0	0	0	0	0	0	0
0	0	0	0	0	0	0	0	0	0	0	0	0	0	0	0	0	0	0	0	0

0	0	0	0	0	0	0	0	0	0	0	0	0	0	0	0	0	0	0	0	0
0	0	0	0	0	0	0	0	0	0	0	0	0	0	0	0	0	0	0	0	0
0	0	0	0	0	0	0	0	0	0	0	0	0	0	0	0	0	0	0	0	0
0	0	0	0	0	0	0	0	0	0	0	0	0	0	0	0	0	0	0	0	0
0	0	0	0	0	0	0	0	0	0	0	0	0	0	0	0	0	0	0	0	0
0	0	0	0	0	0	0	0	0	0	0	0	0	0	0	0	0	0	0	0	0
0	0	0	0	0	0	0	0.01	0	0	0	0	0	0	0	0	0	0	0	0	0
0	0	0	0	0	0	0	0	0	0	0	0	0	0	0	0	0	0	0	0	0
0	0	0	0	0	0	0	0	0	0	0	0	0	0	0	0	0	0	0	0	0
0	0	0	0	0	0	0	0.01	0	0	0	0	0	0	0	0	0	0	0	0	0
0	0	0	0	0	0	0	0.01	0	0	0	0	0	0	0	0	0	0	0	0	0
0	0	0	0	0	0	0	0	0	0	0	0	0	0	0	0	0	0	0	0	0
0	0	0	0	0	0	0	0	0	0	0	0	0	0	0	0	0	0	0	0	0
0	0	0	0	0	0	0	0.01	0	0	0	0	0	0	0	0	0	0	0	0	0
0	0	0	0	0	0	0	0	0	0	0	0	0	0	0	0	0	0	0	0	0
0	0	0	0	0	0	0	0	0	0	0	0	0	0	0	0	0	0	0	0	0
0	0	0	0	0	0	0	0	0	0	0	0	0	0	0	0	0	0	0	0	0
0	0	0	0	0	0	0	0	0	0	0	0	0	0	0	0	0	0	0	0	0
0	0	0	0	0	0	0	0	0	0	0	0	0	0	0	0	0	0	0	0	0
0	0	0	0	0	0	0	0	0	0	0	0	0	0	0	0	0	0	0	0	0
0	0	0	0	0	0	0	0	0	0	0	0	0	0	0	0	0	0	0	0	0
0	0	0	0	0	0	0	0	0	0	0	0	0	0	0	0	0	0	0	0	0
0	0	0	0	0	0	0	0	0	0	0	0	0	0	0	0	0	0	0	0	0
0	0	0	0	0	0	0	0	0	0	0	0	0	0	0	0	0	0	0	0	0
0	0	0	0	0	0	0	0	0	0	0	0	0	0	0	0	0	0	0	0	0
0	0	0	0	0	0	0	0	0	0	0	0	0	0	0	0	0	0	0	0	0
0	0	0	0	0	0	0	0	0	0	0	0	0	0	0	0	0	0	0	0	0
0	0	0	0	0	0	0	0	0	0	0	0	0	0	0	0	0	0	0	0	0
0	0	0	0	0	0	0	0	0	0	0	0	0	0	0	0	0	0	0	0	0
0	0	0	0	0	0	0	0	0	0	0	0	0	0	0.01	0	0	0	0	0	0
0	0	0	0	0	0	0	0	0	0	0	0	0	0	0	0	0	0	0	0	0
0	0	0	0	0	0	0	0	0	0	0	0	0	0	0	0	0	0	0	0	0
0	0	0	0	0	0	0	0	0	0	0	0	0	0	0	0	0	0	0	0	0
0	0	0	0	0	0	0	0	0	0	0	0	0	0	0	0	0	0	0	0	0
0	0	0	0	0	0	0	0	0	0	0	0	0	0	0	0	0	0	0	0	0
0	0	0	0	0	0	0	0	0	0	0	0	0	0	0	0	0	0	0	0	0

0	0	0	0	0	0	0	0	0	0	0	0	0	0	0	0	0	0	0	0	0
0	0	0	0	0	0	0	0	0	0	0	0	0	0	0	0	0	0	0	0	0
0	0	0	0	0	0	0	0	0	0	0	0	0	0	0	0	0	0	0	0	0
0	0	0	0	0	0	0	0	0	0	0	0	0	0	0	0	0	0	0	0	0
0	0	0	0	0	0	0	0	0	0	0	0	0	0	0	0	0	0	0	0	0
0	0	0	0	0	0	0	0	0	0	0	0	0	0	0	0	0	0	0	0	0
0	0	0	0	0	0	0	0	0	0	0	0	0	0	0	0	0	0	0	0	0
0	0	0	0	0	0	0	0	0	0	0	0	0	0	0	0	0	0	0	0	0
0	0	0	0	0	0	0	0	0	0	0	0	0	0	0	0	0	0	0	0	0
0	0	0	0	0	0	0	0	0	0	0	0	0	0	0	0	0	0	0	0	0
0	0	0	0	0	0	0	0	0	0	0	0	0	0	0	0	0	0	0	0	0
0	0	0	0	0	0	0	0	0	0	0	0	0	0	0	0	0	0	0	0	0
0	0	0	0	0	0	0	0	0	0	0	0	0	0	0	0	0	0	0	0	0
0	0	0	0	0	0	0	0	0	0	0	0	0	0	0	0	0	0	0	0	0
0	0	0	0	0	0	0	0	0	0	0	0	0	0	0	0	0	0	0	0	0
0	0	0	0	0	0	0	0	0	0	0	0	0	0	0	0	0	0	0	0	0
0	0	0	0	0	0	0	0	0	0	0	0	0	0	0	0	0	0	0	0	0
0	0	0	0	0	0	0	0	0	0	0	0	0	0	0	0	0	0	0	0	0
0	0	0	0	0	0	0	0	0	0	0	0	0	0	0	0	0	0	0	0	0
0	0	0	0	0	0	0	0	0	0	0	0	0	0	0	0	0	0	0	0	0
0	0	0	0	0	0	0	0	0	0	0	0	0	0	0	0	0	0	0	0	0
0	0	0	0	0.01	0	0	0	0	0	0	0	0	0	0	0	0	0	0	0	0
0	0	0	0	0	0	0	0	0	0	0	0	0	0	0	0	0	0	0	0	0
0	0	0	0	0	0	0	0	0	0	0	0	0	0	0	0	0	0	0	0	0
0	0	0	0	0	0	0	0	0	0	0	0	0	0	0	0	0	0	0	0	0
0	0	0	0	0	0	0	0	0	0	0	0	0	0	0	0	0	0	0	0	0
0	0	0	0	0	0	0	0	0	0	0	0	0	0	0	0	0	0	0	0	0
0	0	0	0	0	0	0	0	0	0	0	0	0	0	0	0	0	0	0	0	0
0	0	0	0	0	0	0	0	0	0	0	0	0	0	0	0	0	0	0	0	0
0	0	0	0	0	0	0	0	0	0	0	0	0	0	0	0	0	0	0	0	0
0	0	0	0	0	0	0	0	0	0	0	0	0	0	0	0	0	0	0	0	0
0	0	0	0	0	0	0	0	0	0	0	0	0	0	0	0	0	0	0	0	0
0	0	0	0	0	0	0	0	0	0	0	0	0	0	0	0	0	0	0	0	0
0	0	0	0	0	0	0	0	0	0	0	0	0	0	0	0	0	0	0	0	0
0	0	0	0	0	0	0	0	0	0	0	0	0	0	0	0	0	0	0	0	0
0	0	0	0	0	0	0	0	0	0	0	0	0	0	0	0	0	0	0	0	0

0	0	0	0	0	0	0	0	0	0	0	0	0	0	0	0	0	0	0	0	0
0	0	0	0	0	0	0	0	0	0	0	0	0	0	0	0	0	0	0	0	0
0	0	0	0	0	0	0	0	0	0	0	0	0	0	0	0	0	0	0	0	0
0	0	0	0	0	0	0	0	0	0	0	0	0	0	0	0	0	0	0	0	0
0	0	0	0	0	0	0	0	0	0	0	0	0	0	0	0	0	0	0	0	0
0	0	0	0	0	0	0	0	0	0	0	0	0	0	0	0	0	0	0	0	0
0	0	0	0	0	0	0	0	0	0	0	0	0	0	0	0	0	0	0	0	0
0	0	0	0	0	0	0	0	0	0	0	0	0	0	0	0	0	0	0	0	0
0	0	0	0	0	0	0	0	0	0	0	0	0	0	0	0	0	0	0	0	0
0	0	0	0	0	0	0	0	0	0	0	0	0	0	0	0	0	0	0	0	0
0	0	0	0	0	0	0	0	0	0	0	0	0	0	0	0	0	0	0	0	0
0	0	0	0	0	0	0	0	0	0	0	0	0	0	0	0	0	0	0	0	0
0	0	0	0	0	0	0	0	0	0	0	0	0	0	0	0	0	0	0	0	0
0	0	0	0	0	0	0	0	0	0	0	0	0	0	0	0	0	0	0	0	0
0	0	0	0	0	0	0	0	0	0	0	0	0	0	0	0	0	0	0	0	0
0	0	0	0	0	0	0	0	0	0	0	0	0	0	0	0	0	0	0	0	0
0	0	0	0	0	0	0	0	0	0	0	0	0	0	0	0	0	0	0	0	0
0	0	0	0	0	0	0	0	0	0	0	0	0	0	0	0	0	0	0	0	0
0	0	0	0	0	0	0	0	0	0	0	0	0	0	0	0	0	0	0	0	0
0	0	0	0	0	0	0	0	0	0	0	0	0	0	0	0	0	0	0	0	0
0	0	0	0	0	0	0	0	0	0	0	0	0	0	0	0	0	0	0	0	0
0	0	0	0	0	0	0	0	0	0	0	0	0	0	0	0	0	0	0	0	0
0	0	0	0	0	0	0	0	0	0	0	0	0	0	0	0	0	0	0	0	0
0	0	0	0	0	0	0	0	0	0	0	0	0	0	0	0	0	0	0	0	0
0	0	0	0	0	0	0	0	0	0	0	0	0	0	0	0	0	0	0	0	0
0	0	0	0	0	0	0	0	0	0	0	0	0	0	0	0	0	0	0	0	0
0	0	0	0	0	0	0	0	0	0	0	0	0	0	0	0	0	0	0	0	0
0	0	0	0	0	0	0	0	0	0	0	0	0	0	0	0	0	0	0	0	0
0	0	0	0	0	0	0	0	0	0	0	0	0	0	0	0	0	0	0	0	0
0	0	0	0	0	0	0	0	0	0	0	0	0	0	0	0	0	0	0	0	0
0	0	0	0	0	0	0	0	0	0	0	0	0	0	0	0	0	0	0	0	0
0	0	0	0	0	0	0	0	0	0	0	0	0	0	0	0	0	0	0	0	0
0	0	0	0	0	0	0	0	0	0	0	0	0	0	0	0	0	0	0	0	0
0	0	0	0	0	0	0	0	0	0	0	0	0	0	0	0	0	0	0	0	0
0	0	0	0	0	0	0	0	0	0	0	0	0	0	0	0	0	0	0	0	0
0	0	0	0	0	0	0	0	0	0	0	0	0	0	0	0	0	0	0	0	0

0	0	0	0	0	0	0	0	0	0	0	0	0	0	0	0	0	0	0	0	0
0	0	0	0	0	0	0	0	0	0	0	0	0	0	0	0	0	0	0	0	0
0	0	0	0	0	0	0	0	0	0	0	0	0	0	0	0	0	0	0	0	0
0	0	0	0	0	0	0	0	0	0	0	0	0	0	0	0.01	0	0	0	0	0
0	0	0	0	0	0	0	0	0	0	0	0	0	0	0	0	0	0	0	0	0
0	0	0	0	0	0	0	0	0	0	0	0	0	0	0	0	0	0	0	0	0
0	0	0	0	0	0	0	0	0	0	0	0	0	0	0	0	0	0	0	0	0
0	0	0	0	0	0	0	0	0	0	0	0	0	0	0	0	0	0	0	0	0
0	0	0	0	0	0	0	0	0	0	0	0	0	0	0	0	0	0	0	0	0
0	0	0	0	0	0	0	0	0	0	0	0	0	0	0	0	0	0	0	0	0
0	0	0	0	0	0	0	0	0	0	0	0	0	0	0	0	0	0	0	0	0
0	0	0	0	0	0	0	0	0	0	0	0	0	0	0	0	0	0	0	0	0
0	0	0	0	0	0	0	0	0	0	0	0	0	0	0	0	0	0	0	0	0
0	0	0	0	0	0	0	0	0	0	0	0	0	0	0	0	0	0	0	0	0
0	0	0	0	0	0	0	0	0	0	0	0	0	0	0	0	0	0	0	0	0
0	0	0	0	0	0	0	0	0	0	0	0	0	0	0	0	0	0	0	0	0
0	0	0	0	0	0	0	0	0	0	0	0	0	0	0	0	0	0	0	0	0
0	0	0	0	0	0	0	0	0	0	0	0	0	0	0	0	0	0	0	0	0
0	0	0	0	0	0	0	0	0	0	0	0	0	0	0	0	0	0	0	0	0
0	0	0	0	0	0	0	0	0	0	0	0	0	0	0	0	0	0	0	0	0
0	0	0	0	0	0	0	0	0	0	0	0	0	0	0	0	0	0	0	0	0
0	0	0	0	0	0	0	0	0	0	0	0	0	0	0	0	0	0	0	0	0
0	0	0	0	0	0	0	0	0	0	0	0	0	0	0	0	0	0	0	0	0
0	0	0	0	0	0	0	0	0	0	0	0	0	0	0	0	0	0	0	0	0
0	0	0	0	0	0	0	0	0	0	0	0	0	0	0	0	0	0	0	0	0
0	0	0	0	0	0	0	0	0	0	0	0	0	0	0	0	0	0	0	0	0
0	0	0	0	0	0	0	0	0	0	0	0	0	0	0	0	0	0	0	0	0
0	0	0	0	0	0	0	0	0	0	0	0	0	0	0	0	0	0	0	0	0
0	0	0	0	0	0	0	0	0	0	0	0	0	0	0	0	0	0	0	0	0
0	0	0	0	0	0	0	0	0	0	0	0	0	0	0	0	0	0	0	0	0
0	0	0	0	0	0	0	0	0	0	0	0	0	0	0	0	0	0	0	0	0
0	0	0	0	0	0	0	0	0	0	0	0	0	0	0	0	0	0	0	0	0
0	0	0	0	0	0	0	0	0	0	0	0	0	0	0	0	0	0	0	0	0
0	0	0	0	0	0	0	0	0	0	0	0	0	0	0	0	0	0	0	0	0
0	0	0	0	0	0	0	0	0	0	0	0	0	0	0	0	0	0	0	0	0
0	0	0	0	0	0	0	0	0	0	0	0	0	0	0	0	0	0	0	0	0

0	0	0	0	0	0	0	0	0	0	0	0	0	0	0	0	0	0	0	0	0
0	0	0	0	0	0	0	0	0	0	0	0	0	0	0	0	0	0	0	0	0
0	0	0	0	0	0	0	0	0	0	0	0	0	0	0	0	0	0	0	0	0
0	0	0	0	0	0	0	0	0	0	0	0	0	0	0	0	0	0	0	0	0
0	0	0	0	0	0	0	0	0	0	0	0	0	0	0	0	0	0	0	0	0
0	0	0	0	0	0	0	0	0	0	0	0	0	0	0	0	0	0	0	0	0
0	0	0	0	0	0	0	0	0	0	0	0	0	0	0	0	0	0	0	0	0
0	0	0	0	0	0	0	0	0	0	0	0	0	0	0	0	0	0	0	0	0
0	0	0	0	0	0	0	0	0	0	0	0	0	0	0	0	0	0	0	0	0
0	0	0	0	0	0	0	0	0	0	0	0	0	0	0	0	0	0	0	0	0
0	0	0	0	0	0	0	0	0	0	0	0	0	0	0	0	0	0	0	0	0
0	0	0	0	0	0	0	0	0	0	0	0	0	0	0	0	0	0	0	0	0
0	0	0	0	0	0	0	0	0	0	0	0	0	0	0	0	0	0	0	0	0
0	0	0.01	0.02	0	0.04	0.12	0	0	0	0	0	0	0	0	0	0	0	0	0	0
0	0	0	0	0	0	0	0	0	0.01	0	0	0	0	0	0	0.01	0	0	0	0
0	0	0	0	0	0	0	0	0	0	0	0	0	0	0	0	0	0	0	0	0
0	0	0	0	0	0	0	0	0	0	0	0	0	0	0	0	0	0	0	0	0
0	0	0	0	0	0	0	0	0	0	0	0	0	0	0	0	0	0	0	0	0
0	0	0	0	0	0	0	0	0	0	0	0	0	0	0	0	0	0	0	0	0
0	0	0	0	0	0	0	0	0	0	0	0	0	0	0	0	0	0	0	0	0
0	0	0	0	0	0	0	0	0	0	0	0	0	0	0	0	0	0	0	0	0
0	0	0	0	0	0	0	0	0	0	0	0	0	0	0	0	0	0	0	0	0
0	0	0	0	0	0	0	0	0	0	0	0	0	0	0	0	0	0	0	0	0
0	0	0	0	0	0	0	0	0	0	0	0	0	0	0	0	0	0	0	0	0
0	0	0	0	0	0	0	0	0	0	0	0	0	0	0	0	0	0	0	0	0
0	0	0	0	0	0	0	0	0	0	0	0	0	0	0	0	0	0	0	0	0
0	0	0	0	0	0	0	0	0	0	0	0	0	0	0	0	0	0	0	0	0
0	0	0	0	0	0	0	0	0	0	0	0	0	0	0	0	0	0	0	0	0
0	0	0	0	0	0	0	0	0	0	0	0	0	0	0	0	0	0	0	0	0
0	0.02	0	0	0	0	0	0	0	0	0	0	0	0	0	0	0	0	0	0	0
0	0	0	0	0	0	0	0	0	0	0	0	0	0	0	0	0	0	0	0	0
0	0	0	0	0	0	0	0	0	0	0	0	0	0	0	0	0	0	0	0	0
0	0	0	0	0	0	0	0	0	0	0	0	0	0	0	0	0	0	0	0	0
0	0	0	0	0	0	0	0	0	0	0	0	0	0	0	0	0	0	0	0	0
0	0	0	0	0	0	0	0	0	0	0	0	0	0	0	0	0	0	0	0	0
0	0	0	0	0	0	0	0	0	0	0	0	0	0	0	0	0	0	0	0	0

0	0	0	0	0	0	0	0	0	0	0	0	0	0	0	0	0	0	0	0	0
0	0	0	0	0	0	0	0	0	0	0	0	0	0	0	0	0	0	0	0	0
0	0	0	0	0	0	0	0	0	0	0	0	0	0	0	0	0	0	0	0	0
0	0	0	0	0	0	0	0	0	0	0	0	0	0	0	0	0	0	0	0	0
0	0	0	0	0	0	0	0	0.01	0	0	0	0	0	0	0	0.01	0	0.01	0	0
0	0	0.01	0.01	0.01	0	0	0	0.02	0	0.01	0	0	0	0	0	0	0.01	0.02	0.01	0.01
0	0	0	0	0	0	0	0	0	0	0	0	0	0	0	0	0	0	0	0	0
0	0.02	0.01	0.01	0	0	0	0	0.02	0	0	0	0	0	0	0.01	0	0	0.01	0	0
0	0	0	0	0	0	0	0	0	0	0	0	0	0	0	0	0	0	0	0	0
0	0	0	0	0	0.01	0.01	0	0.02	0	0	0	0	0	0	0	0.01	0	0.01	0	0
0	0	0	0	0	0	0	0	0	0	0	0	0	0	0	0	0	0	0	0	0
0	0	0	0	0.05	0	0.05	0	0	0.01	0.01	0.03	0	0	0	0	0	0.02	0	0	0
0	0.02	0.02	0.01	0.02	0.01	0.01	0.01	0	0.01	0.01	0.01	0.01	0.01	0.01	0	0.01	0.02	0	0	0

miR_T-	miR_T-	miR_T-	miR_T-	miR_T-	miR_T-	miR_T-	miR_T-	miR_T-	miR_T-	miR_T-	miR_T-	miR_T-	miR_T-	miR_T-	miR_T-	miR_T-	miR_Te	miR_Te	miR_Te	miR_Te
0.01	0.02	0.02	0.01	0.05	0.01	0.04	0.03	0.03	0.03	0.04	0	0.01	0.01	0.01	0.04	0.01	0	0	0	0.04
0.04	0.01	0.04	0.05	0.02	0.02	0.01	0.01	0.02	0.01	0.01	0	0.01	0.01	0	0.02	0	0	0	0	0.09
0.02	0	0.02	0.03	0.01	0.01	0	0	0	0	0	0	0.02	0.01	0	0	0	0	0	0	0.02
0.01	0	0	0	0	0	0	0	0.01	0.01	0.01	0	0	0	0	0	0	0	0	0	0
0	0	0	0	0	0	0	0	0	0	0	0	0	0	0	0	0	0	0	0	0
0	0	0	0	0.01	0.01	0.01	0.01	0.03	0.02	0.03	0	0.01	0	0	0.03	0	0	0	0	0.01
0	0	0	0	0	0	0.01	0.01	0.01	0.01	0	0	0	0	0.01	0.01	0.01	0	0	0	0
0.01	0	0.01	0	0	0	0.01	0	0.01	0.01	0.02	0	0.01	0	0	0.01	0	0	0	0	0
0	0	0	0	0	0	0	0	0	0	0	0	0	0	0	0	0	0	0	0	0
0	0	0	0	0	0	0	0	0	0	0	0	0	0	0	0	0	0	0	0	0
0	0	0	0	0	0	0	0	0	0	0	0	0	0	0	0	0	0	0	0	0
0	0	0	0	0	0	0	0	0	0	0	0	0	0	0	0	0	0	0	0	0
0	0	0	0	0	0.01	0.02	0.01	0	0	0.01	0	0	0	0.01	0.01	0	0	0	0	0
0	0	0	0.01	0	0	0	0	0	0	0	0	0	0	0	0	0	0	0.01	0	0
0	0	0	0	0	0	0	0	0	0	0	0	0	0	0	0	0	0	0	0	0
0	0	0	0	0	0	0	0	0	0	0.01	0	0	0	0	0	0	0	0	0.01	0
0	0	0.01	0	0	0.01	0	0	0.01	0.01	0.01	0.01	0	0	0	0	0.01	0	0	0.01	0
0	0	0	0	0	0	0	0	0	0	0	0	0	0	0	0	0	0	0	0	0
0	0	0	0	0	0	0	0	0	0	0	0	0	0	0	0	0	0	0	0	0
0	0	0	0	0	0	0	0	0	0	0	0	0	0	0	0	0	0	0	0	0
0.05	0	0.04	0.05	0	0	0	0	0	0	0	0	0	0	0	0	0.06	0	0	0	0
0	0	0	0	0	0	0	0	0	0	0	0.01	0	0	0	0	0	0.01	0.01	0	0
0	0	0	0	0	0	0	0	0	0	0	0	0	0	0	0	0	0	0	0	0
0	0	0.01	0.01	0	0	0	0	0	0	0	0	0	0	0	0	0	0	0	0	0
0.02	0	0.02	0.02	0	0.01	0	0	0	0	0	0	0	0	0	0	0	0	0	0	0.03
0.01	0.05	0.01	0	0	0.02	0	0	0.02	0.01	0.02	0.04	0	0	0	0	0	0.01	0	0	0.13
0	0	0	0	0	0	0	0	0	0	0	0	0	0	0	0	0	0	0	0	0
0	0	0	0	0	0	0	0	0	0	0	0	0	0	0	0	0	0	0	0	0
0	0	0	0	0	0	0	0	0	0	0	0	0	0	0	0	0	0	0	0	0
0	0	0	0	0	0	0	0	0	0	0	0	0	0	0	0	0	0	0	0	0
0	0	0	0	0	0	0	0	0	0	0	0	0	0	0	0	0	0	0	0	0
0	0	0	0	0	0	0	0	0	0	0	0	0	0	0	0	0	0	0	0	0
0	0	0	0	0	0	0	0	0	0	0	0	0	0	0	0	0	0	0	0	0
0.02	0.01	0.02	0.02	0.01	0.01	0	0	0	0	0	0	0	0	0	0	0	0.04	0.03	0.03	0
0	0	0	0	0	0	0	0	0	0	0	0	0	0	0	0	0	0.01	0	0	0

0	0	0	0	0	0	0	0	0	0	0	0	0	0	0	0	0	0	0	0	0
0	0	0	0	0	0	0	0	0	0	0	0	0	0	0	0	0	0	0	0	0
0	0	0	0	0	0	0	0	0	0	0	0	0	0	0	0	0	0	0	0	0
0	0	0	0	0	0	0	0	0	0	0	0	0	0	0	0	0	0	0	0	0
0	0	0	0	0	0	0	0	0	0	0	0	0	0	0	0	0	0	0	0	0
0	0	0	0	0	0.01	0	0	0	0	0	0	0	0	0	0	0	0.02	0	0	0
0	0	0	0	0	0	0	0	0	0	0	0	0	0	0	0	0	0	0	0	0
0	0	0	0	0	0	0	0	0	0	0	0	0	0	0	0	0	0	0	0	0
0	0	0	0	0	0	0	0	0	0	0	0	0	0	0	0	0	0	0	0	0
0	0	0	0	0	0	0	0	0	0	0	0	0.01	0	0	0	0	0	0	0	0
0	0	0	0	0	0	0	0	0	0	0	0	0	0	0	0	0	0	0	0	0
0	0	0	0	0	0	0.01	0.01	0	0	0	0.03	0	0	0.01	0	0	0	0	0	0
0.01	0.02	0.01	0	0	0.01	0.01	0.01	0	0	0.01	0.01	0.02	0	0.01	0.01	0.01	0.01	0	0	0.01
0.01	0	0.01	0.01	0	0	0	0	0	0	0	0	0	0	0	0	0	0	0.07	0	0
0.02	0.13	0.03	0	0.08	0.03	0.16	0.12	0.08	0.1	0.2	0.13	0.17	0.12	0.16	0.15	0.19	0	0	0	0
0.04	0.19	0.14	0	0.28	0.08	0.26	0.33	0.2	0.16	0.17	0.17	0.36	0.54	0.41	0.23	0.4	0	0	0	0
0.01	0	0.01	0.02	0	0	0	0	0	0	0	0	0	0	0	0	0.01	0	0	0	0.2
0	0.07	0	0	0.01	0.1	0	0	0	0	0	0.01	0	0	0	0	0	0	0	0	0
0	0	0	0	0	0	0	0	0	0	0	0	0	0	0	0	0	0	0	0	0.01
0	0	0	0	0	0	0	0.02	0	0	0.01	0	0.01	0	0	0.01	0	0	0	0	0
0	0	0	0	0	0	0	0	0	0	0	0	0	0	0	0	0	0	0	0	0
0	0	0	0	0	0	0	0	0	0	0	0	0	0	0	0	0	0	0	0	0
0	0	0	0	0	0	0	0	0	0	0	0	0	0	0	0	0	0	0	0	0
0	0	0	0	0	0	0	0	0	0	0	0	0	0	0	0	0	0	0	0	0
0	0	0	0	0	0	0	0	0	0	0	0	0	0	0	0	0	0	0	0	0
0	0	0	0	0	0	0	0	0	0	0	0	0	0	0	0	0	0	0	0	0
0	0	0	0	0	0	0	0	0	0	0	0	0	0	0	0	0	0	0	0	0
0	0	0	0	0	0	0.11	0.03	0	0	0	0	0.03	0.03	0.07	0.05	0.02	0	0	0	0
0	0	0	0	0	0	0	0	0	0	0	0	0	0	0	0	0	0	0	0	0
0	0	0	0	0	0	0	0	0	0	0	0	0	0	0	0	0	0	0	0.01	0
0	0	0	0	0	0	0	0	0	0	0	0	0	0	0	0	0	0	0	0	0
0	0	0	0	0	0	0	0	0	0	0	0	0	0	0	0	0	0	0	0	0
0	0	0	0	0	0	0	0	0	0	0	0	0	0	0	0	0	0	0	0	0
0	0	0	0	0	0	0	0	0	0.01	0.01	0	0.01	0	0	0	0	0	0	0	0
0.09	0.1	0.06	0.07	0.11	0.04	0.01	0.02	0.07	0.08	0.12	0	0.03	0.02	0.01	0.01	0	0.02	0.01	0.04	0.01
0.01	0	0.01	0.01	0	0	0	0	0.01	0.01	0.01	0	0	0.01	0	0	0	0	0.01	0.01	0

0.22	0.27	0.14	0.16	0.08	0.05	0.03	0.03	0.14	0.17	0.1	0.19	0.07	0.08	0.07	0.04	0.05	0.1	0.05	0.08	0.09
0	0	0.01	0	0.01	0.02	0	0	0.01	0.02	0.01	0	0	0.01	0	0	0	0.03	0.02	0.01	0
0	0	0	0	0.03	0	0.01	0	0.07	0.06	0.02	0.01	0	0	0.01	0	0	0	0	0	0.01
0	0	0	0	0	0	0	0	0.01	0	0.01	0	0	0	0	0	0	0	0	0	0
0	0	0	0	0	0	0	0	0	0	0	0	0.01	0	0	0	0	0	0	0	0
0	0	0	0	0	0	0	0	0	0	0	0	0	0	0	0	0	0	0	0	0
0	0	0	0	0	0	0	0	0	0	0.01	0	0	0	0	0	0	0	0.01	0	0
0	0	0	0	0	0	0	0	0	0	0	0	0	0	0	0	0	0	0	0	0
0	0	0	0	0	0	0	0	0	0	0	0	0	0	0	0	0	0	0.01	0	0
0	0	0	0	0	0	0	0	0	0	0	0	0	0	0	0	0	0	0	0	0
0	0	0	0	0	0	0	0	0	0	0	0	0	0	0.01	0	0	0.02	0.01	0	0
0	0	0	0	0	0	0	0	0	0	0	0	0	0	0	0	0	0	0	0	0
0	0	0	0	0	0	0	0	0	0	0	0	0	0	0	0	0	0	0	0	0
0	0	0	0	0	0	0	0	0	0	0	0	0	0	0	0	0	0	0	0	0
0	0.01	0	0	0	0	0	0	0.01	0.01	0	0	0	0	0	0	0	0.01	0.01	0.01	0
0	0	0	0	0	0	0	0	0	0	0.02	0	0	0	0	0	0	0	0	0	0
0	0	0	0	0	0	0	0	0	0	0	0	0	0	0	0	0	0	0	0	0
0	0	0	0	0	0	0	0	0	0	0	0	0	0	0	0	0	0	0	0	0
0.02	0	0.01	0.02	0.01	0.02	0	0.01	0.01	0.01	0	0.01	0	0	0	0.01	0	0	0	0	0
0	0	0	0	0	0	0	0	0	0	0	0	0	0	0	0	0	0	0	0	0
0	0	0	0	0	0	0	0	0	0	0	0	0	0	0	0	0	0	0	0	0
0	0	0	0	0	0	0	0	0	0	0	0	0	0	0	0	0	0	0	0	0
0	0	0	0	0	0	0	0	0	0	0	0	0	0	0	0	0	0	0	0	0
0	0	0	0	0	0.02	0	0	0	0	0	0	0	0	0	0	0	0	0	0	0
0	0	0.01	0	0	0	0	0	0	0	0	0	0	0	0	0	0	0	0	0	0.01
0	0	0	0	0	0	0	0	0	0	0	0	0	0	0	0	0	0	0	0	0
0	0	0	0	0	0	0	0	0	0	0	0	0	0	0	0	0	0.01	0	0	0
0	0	0	0	0	0	0	0	0	0	0	0	0	0	0	0	0	0	0	0	0
0	0	0	0	0	0	0	0	0	0	0	0	0	0	0	0	0	0	0	0	0
0	0	0	0	0	0	0	0	0	0	0	0	0	0	0	0	0	0	0	0	0
0	0	0	0	0	0	0	0	0	0	0	0	0	0	0	0	0	0	0	0	0
0	0	0	0	0	0	0	0	0	0	0	0	0	0	0	0	0	0	0	0	0
0	0	0	0	0	0	0	0	0	0	0	0	0	0	0	0	0	0	0	0	0
0	0	0	0	0	0	0	0	0.01	0.01	0	0	0	0	0	0	0	0	0.01	0.01	0
0	0	0	0	0.01	0.01	0	0.01	0.01	0.02	0.02	0	0	0	0	0	0.02	0	0.03	0.02	0
0	0	0	0	0	0	0	0	0	0	0	0	0	0	0	0	0	0	0	0	0

0	0	0	0	0	0	0	0	0	0	0	0	0	0	0	0	0	0	0	0	0
0	0	0	0	0	0	0	0	0	0	0	0	0	0	0	0	0	0	0.03	0	0
0	0	0	0	0	0	0	0	0	0	0	0	0	0	0	0	0	0	0	0	0.02
0	0	0	0	0	0	0	0	0	0	0	0	0	0	0	0	0	0	0	0	0
0	0	0	0	0	0	0	0	0	0	0	0	0	0	0	0	0	0	0	0	0
0	0	0	0	0	0	0	0	0	0	0	0	0	0	0	0	0	0	0.01	0	0
0	0	0	0	0	0	0	0	0	0	0	0	0	0	0	0	0	0	0	0	0
0	0	0	0	0	0	0	0	0	0	0	0	0	0	0	0	0	0	0	0	0
0	0	0	0	0	0	0	0	0	0	0	0	0	0	0	0	0	0	0	0	0
0	0.01	0	0	0	0	0	0	0.01	0.01	0.01	0	0	0	0	0	0	0	0.01	0.01	0
0	0	0	0	0	0	0	0	0	0	0.01	0	0	0	0	0	0	0	0	0	0
0.02	0.01	0.01	0.02	0.04	0.14	0.04	0.04	0	0.01	0	0.04	0.04	0.01	0.02	0.03	0	0	0.09	0.04	0
0	0	0	0	0	0	0	0	0.02	0.01	0	0	0	0	0	0	0	0	0	0	0
0	0	0	0	0	0	0	0	0	0	0	0	0	0	0	0	0	0	0	0	0
0	0	0	0	0	0	0	0	0	0	0	0	0	0	0	0	0	0	0	0	0
0	0	0	0	0	0	0	0	0	0	0	0	0	0	0	0	0	0	0	0	0.01
0	0	0	0	0	0	0	0	0	0	0	0	0	0	0	0	0	0	0	0	0
0	0	0	0	0	0	0	0	0	0	0	0	0	0	0	0	0	0	0	0	0
0	0	0	0	0	0	0	0	0	0	0	0	0	0	0	0	0	0	0	0	0
0	0	0	0	0	0	0	0	0	0	0	0	0	0	0	0	0	0	0	0	0
0	0	0	0	0	0	0	0	0	0	0	0	0	0	0	0	0	0	0	0	0
0	0	0	0	0	0	0	0	0	0	0	0	0	0	0	0	0	0	0	0	0
0	0	0	0	0	0	0	0	0	0	0	0	0	0	0	0	0	0	0	0	0
0.02	0	0.02	0.03	0.01	0.01	0	0.01	0	0	0	0	0	0	0.01	0	0	0	0.02	0	0.02
0	0	0	0	0	0	0	0	0	0	0	0	0	0	0	0	0	0	0	0	0
0	0	0	0	0	0	0	0	0	0	0	0	0	0	0	0	0	0	0	0	0
0	0	0	0	0	0	0	0	0	0	0	0	0	0	0	0	0	0	0	0	0
0	0	0	0	0	0	0	0	0	0	0	0	0	0	0	0	0	0	0	0	0
0	0	0	0	0	0	0	0.01	0	0	0	0	0	0	0	0	0	0	0	0	0
0	0	0	0	0	0	0	0	0	0	0.02	0	0	0	0	0	0	0	0	0	0
0	0	0	0	0	0	0	0	0	0	0	0	0	0	0	0	0	0	0	0	0
0.01	0	0.01	0.01	0	0	0	0.01	0	0	0	0	0	0	0	0	0	0	0	0	0.01
0.01	0	0	0.01	0	0	0	0	0	0	0	0	0	0	0	0	0	0	0	0	0.01
0.02	0.01	0.03	0.04	0	0.02	0	0.01	0	0	0	0	0	0	0.01	0	0.01	0.01	0.01	0.01	0.01
0.01	0	0.01	0.01	0	0.01	0	0	0.01	0.01	0.02	0	0	0	0	0	0	0	0	0	0
0	0.02	0	0	0.01	0.01	0.02	0.03	0	0	0	0	0.01	0.01	0.02	0.04	0	0	0.01	0.01	0.01

0	0	0	0	0.01	0.01	0.01	0	0	0	0.01	0	0	0	0.01	0.02	0	0	0	0	0
0.03	0.01	0.06	0.05	0.01	0.01	0	0	0.01	0	0.01	0.01	0	0	0.02	0.01	0.02	0.01	0.01	0.01	0.01
0.02	0	0.01	0.03	0	0.01	0	0	0	0	0	0.03	0	0.01	0	0	0	0.01	0.01	0	0.01
0	0	0	0	0	0	0	0	0	0	0	0	0	0	0	0	0	0	0	0	0
0	0	0	0	0	0.01	0	0	0	0	0	0	0	0	0	0	0.01	0.01	0	0	0
0	0	0	0	0	0	0	0	0	0	0	0	0	0	0	0	0	0	0	0	0
0	0	0	0	0	0	0	0	0	0	0	0	0	0	0	0	0	0	0	0	0
0	0	0	0	0	0	0	0	0	0	0	0	0	0	0	0	0	0	0	0	0
0	0	0	0	0	0	0	0	0	0	0	0	0	0	0	0	0	0	0	0	0
0	0	0	0	0	0	0	0	0	0	0	0	0	0	0	0	0	0	0	0	0
0	0	0.01	0	0	0	0.03	0.03	0	0	0	0.04	0	0.01	0.02	0.03	0.01	0	0	0	0.01
0.01	0	0	0.02	0.01	0.03	0.07	0.08	0.01	0.01	0.02	0.08	0.02	0.02	0.05	0.06	0.04	0	0.01	0.01	0
0	0	0	0	0	0	0	0.01	0	0	0	0	0	0	0.01	0	0.01	0	0	0	0
0	0	0	0	0	0	0	0	0	0	0	0	0	0	0	0	0	0	0	0	0
0	0	0	0	0	0	0	0	0	0	0	0	0	0	0	0	0	0	0	0	0
0	0	0	0	0	0	0	0	0	0	0.01	0	0	0	0	0	0	0	0	0	0
0	0	0	0	0	0	0	0	0	0	0	0	0	0	0	0	0	0.17	0.1	0.15	0
0	0	0	0	0	0	0	0	0	0	0	0	0	0	0	0	0	0.04	0.07	0.1	0
0	0	0	0	0	0	0	0	0	0	0	0	0	0	0	0	0	0.11	0.03	0.04	0
0	0	0	0	0	0	0	0	0	0	0	0	0	0	0	0	0	0.02	0.03	0.07	0
0.01	0	0	0.01	0	0	0	0	0	0	0	0	0	0	0	0	0	0	0.01	0	0
0	0.01	0	0	0	0	0	0	0	0	0	0	0	0	0	0	0	0	0	0	0
0.01	0	0	0	0	0	0	0	0	0	0	0	0	0	0	0	0	0	0	0	0
0.01	0	0.01	0.01	0.02	0.01	0	0.01	0.01	0.01	0	0	0.03	0.01	0.01	0.01	0.01	0	0	0	0.01
0.01	0	0	0.01	0.02	0.01	0.01	0.04	0.01	0.01	0.01	0.03	0.05	0.02	0.02	0.02	0.04	0.02	0.01	0	0
0	0	0	0	0	0	0	0	0	0	0	0	0	0	0	0	0	0	0	0	0
0.01	0	0	0	0.03	0.03	0.01	0	0.01	0.01	0.02	0.04	0.01	0	0.01	0	0	0.02	0.01	0.02	0
0	0	0	0	0	0	0	0	0	0	0	0	0	0	0	0.01	0	0	0	0	0
0	0	0	0	0	0	0	0	0	0	0	0	0	0	0	0	0	0	0	0	0
0	0	0	0	0	0	0	0	0	0	0	0	0	0	0	0	0	0	0	0	0
0	0	0	0	0	0	0	0	0	0	0	0	0	0	0	0	0	0	0	0	0
0	0	0	0	0	0.01	0	0	0	0	0	0	0	0	0	0	0	0	0	0	0
0	0	0	0	0	0	0	0	0	0	0	0	0	0	0	0	0	0	0	0	0
0	0	0	0	0	0	0	0	0	0	0	0	0	0	0	0	0	0	0	0	0
0	0	0	0	0	0	0	0	0	0	0	0	0	0	0	0	0	0	0	0	0
0	0	0	0	0	0	0	0	0	0	0	0	0	0	0	0	0	0	0	0	0

0	0	0	0	0	0	0	0	0	0	0	0	0	0	0	0	0	0	0	0	0
0	0	0	0	0	0	0	0	0	0	0	0	0	0	0	0	0	0	0	0	0
0	0	0	0	0	0	0	0	0	0	0	0	0	0	0	0	0	0	0	0	0
0	0	0	0	0	0	0	0	0	0	0	0	0	0	0	0	0	0	0	0	0
0	0	0	0	0	0	0	0	0	0	0	0	0	0	0	0	0	0	0	0	0
0	0	0	0	0	0	0	0	0	0	0	0	0	0	0	0	0	0	0	0	0
0	0	0	0	0	0	0	0	0	0	0	0	0	0	0	0	0	0	0	0	0
0	0	0	0	0	0	0	0	0	0	0	0	0	0	0	0	0	0	0	0	0
0	0	0	0	0	0	0	0	0	0	0	0	0	0	0	0	0	0	0	0	0
0	0	0	0	0	0	0	0	0	0	0	0	0	0	0	0	0	0	0	0	0
0	0	0	0	0	0	0	0	0	0	0	0	0	0	0	0	0	0	0	0	0
0	0	0	0	0	0	0	0	0	0	0	0	0	0	0	0	0	0	0	0	0
0	0.01	0	0	0	0	0	0	0	0	0.01	0	0	0	0	0	0	0	0	0	0
0	0	0	0	0	0	0	0	0	0	0	0	0	0	0	0	0	0	0	0	0
0	0	0	0	0	0	0	0	0	0	0	0	0	0	0	0	0	0	0	0	0
0	0	0	0	0	0	0	0	0	0	0	0	0.01	0	0	0.01	0	0	0	0	0
0	0	0	0	0	0	0	0	0	0	0	0	0	0	0	0.01	0	0	0	0	0
0	0	0	0	0	0	0	0	0	0	0	0	0	0	0	0	0	0	0	0	0
0	0	0	0	0	0	0	0	0	0	0	0	0	0	0	0	0	0	0	0	0
0	0	0.01	0	0	0	0	0	0	0	0	0	0	0	0	0	0	0	0	0	0
0	0	0	0	0	0	0	0	0	0	0	0	0	0	0	0	0	0	0	0	0
0	0	0	0	0	0	0	0	0	0	0	0	0	0	0	0	0	0	0	0	0
0	0	0	0	0	0	0	0	0	0	0	0	0	0	0	0	0	0	0	0	0
0	0	0	0	0	0	0	0	0	0	0	0	0	0	0	0	0	0	0	0	0
0	0	0	0	0	0	0	0	0	0	0	0	0	0	0	0	0	0	0	0	0
0	0	0	0	0	0	0	0	0	0	0	0	0	0	0	0	0	0	0	0	0
0	0	0	0	0	0	0	0	0	0	0	0	0	0	0	0	0	0	0	0	0
0	0	0	0	0	0	0	0	0	0	0.02	0	0	0	0	0	0	0	0	0	0
0	0	0	0	0	0	0	0	0	0	0	0	0	0	0	0	0	0	0	0	0
0	0	0	0	0	0	0	0	0	0	0	0	0	0	0	0	0	0.09	0.04	0.11	0
0	0	0	0	0	0	0	0	0	0	0	0	0	0	0	0	0	0	0	0	0
0	0	0	0	0	0	0	0	0	0	0	0	0	0	0	0	0	0	0	0	0
0	0	0	0	0	0	0	0	0	0	0	0	0	0	0	0	0	0	0	0	0
0	0	0	0	0	0	0	0	0	0	0	0	0	0	0	0	0	0	0	0	0
0	0	0	0	0	0	0	0	0	0	0	0	0	0	0	0	0	0	0.01	0	0
0	0	0	0	0	0	0	0	0	0	0	0	0	0	0	0	0	0	0.02	0	0

0	0	0	0	0	0	0	0	0	0	0	0	0	0	0	0	0	0	0.03	0	0
0	0	0	0	0	0	0	0	0	0	0	0	0	0	0	0	0	0	0.03	0	0
0	0	0	0	0	0	0	0	0	0	0.01	0.04	0	0	0	0	0	0.01	0	0	0
0	0	0	0	0	0	0	0	0	0	0	0	0	0	0	0	0	0	0	0	0
0	0	0	0	0	0	0	0	0	0	0	0	0	0	0	0	0	0	0	0	0
0	0	0	0	0	0	0	0	0	0	0	0	0	0	0	0	0	0	0	0	0
0	0	0	0	0	0	0	0	0	0	0	0	0	0	0	0	0	0	0	0	0
0	0	0	0	0	0	0	0	0	0	0	0	0	0	0	0	0	0	0	0	0
0	0	0.01	0	0	0	0	0	0	0	0	0	0	0	0	0	0	0	0	0	0
0	0	0.01	0	0	0	0	0	0	0	0	0	0	0	0	0	0	0	0	0	0
0	0	0	0	0	0	0	0	0	0	0	0	0	0	0	0	0	0	0	0	0
0	0	0	0	0	0	0	0	0	0	0	0	0	0	0	0	0	0	0	0	0
0	0	0	0	0	0	0	0	0	0	0	0	0	0	0	0	0	0	0	0	0
0	0	0	0	0	0	0	0	0	0	0	0	0	0	0	0	0	0	0	0	0
0	0	0	0	0	0	0	0	0	0	0	0	0	0	0	0	0	0	0	0	0
0	0	0	0	0	0	0	0	0	0	0	0	0	0	0	0	0	0	0	0	0
0	0	0	0	0	0	0	0	0	0	0	0	0	0	0	0	0	0	0	0	0
0	0	0	0	0	0	0	0	0	0	0	0	0	0	0	0	0	0	0	0	0
0	0	0	0	0	0	0	0	0	0	0	0	0	0	0	0	0	0	0	0	0
0	0	0	0	0	0	0	0	0	0	0	0	0	0	0	0	0	0	0	0	0
0	0	0	0	0	0	0	0	0	0	0	0	0	0	0	0	0	0	0	0	0
0	0	0	0	0	0	0	0	0	0	0	0	0	0	0	0	0	0	0	0	0
0	0	0	0	0	0	0	0	0	0	0	0	0	0	0	0	0	0	0	0	0
0	0	0	0	0	0	0	0	0	0	0	0.01	0	0	0	0	0	0	0	0	0
0	0	0	0	0	0.01	0	0.01	0	0	0	0	0	0	0	0.01	0	0	0	0	0
0	0	0	0	0	0	0	0	0.01	0.01	0.01	0	0	0	0	0	0	0	0.01	0.05	0.03
0	0	0	0	0	0.01	0	0	0	0	0	0	0.01	0	0	0	0	0	0	0	0
0	0	0	0	0	0	0	0	0	0	0	0	0	0	0	0	0	0	0	0	0
0	0	0	0	0	0	0	0	0	0	0	0	0	0	0	0	0	0	0	0	0
0	0	0	0	0	0	0	0	0	0	0	0	0	0	0	0	0	0	0	0	0
0	0	0	0	0	0	0	0	0	0	0	0	0	0	0	0	0	0	0	0	0
0	0	0	0	0	0	0	0	0	0	0	0	0	0	0	0	0	0	0	0	0
0	0	0	0	0	0	0	0	0	0	0	0	0	0	0	0	0	0	0	0	0
0	0	0	0	0	0	0	0	0	0	0	0	0	0	0	0	0	0	0	0	0
0	0	0	0	0	0	0	0	0	0	0	0	0	0	0	0	0	0	0	0	0
0	0	0	0	0	0	0	0	0	0	0	0	0	0	0	0	0	0	0	0	0

0	0	0	0	0	0	0	0	0	0	0	0	0	0	0	0	0	0	0	0	0
0	0	0	0	0	0.01	0	0	0	0	0	0	0	0	0	0	0	0	0	0	0
0	0	0	0	0	0	0	0	0	0	0	0	0	0	0	0	0	0	0	0	0
0	0	0	0	0	0	0	0	0	0	0	0	0	0	0	0	0	0	0	0	0
0	0	0	0	0	0	0	0	0	0	0	0	0	0	0	0	0	0	0	0	0
0	0	0	0	0	0	0	0	0	0	0	0	0	0	0	0	0	0	0	0	0
0	0	0	0	0	0	0	0	0	0	0	0	0	0	0	0	0	0	0	0	0
0	0	0	0	0	0	0	0	0	0	0	0	0	0	0	0	0	0	0	0	0
0	0	0	0	0	0	0	0	0	0	0	0	0	0	0	0	0	0	0	0	0
0	0	0	0	0	0	0	0	0	0	0	0	0	0	0	0	0	0	0	0	0
0	0	0	0	0	0	0	0	0	0	0	0	0	0	0	0	0	0	0	0	0
0	0	0	0	0	0	0	0	0	0	0	0	0	0	0	0	0	0	0	0	0
0	0	0	0	0	0	0	0	0	0	0	0	0	0	0	0	0	0	0	0	0
0	0	0	0	0	0	0	0	0	0	0	0	0	0	0	0	0	0	0	0	0
0	0	0	0	0	0	0	0	0	0	0	0	0	0	0	0	0	0	0	0	0
0	0	0	0	0	0	0	0	0	0	0	0	0	0	0	0	0	0	0	0	0
0	0	0	0	0	0	0	0	0	0	0	0	0	0	0	0	0	0	0	0	0
0	0	0	0	0	0	0	0	0	0	0	0	0	0	0	0	0	0	0	0	0
0	0	0	0	0	0	0	0	0	0	0	0	0	0	0	0	0	0	0	0	0
0	0	0	0	0	0	0	0	0	0	0	0	0	0	0	0	0	0	0	0	0
0	0	0	0	0	0	0	0	0	0	0	0	0	0	0	0	0	0	0	0	0
0	0	0	0	0	0	0	0	0	0	0	0	0	0	0	0	0	0	0	0	0
0	0	0	0	0	0	0	0	0	0	0	0	0	0	0	0	0	0	0	0	0
0	0	0	0	0	0	0	0	0	0	0	0	0	0	0	0	0	0	0	0	0
0	0	0	0	0	0	0	0	0	0	0	0	0	0	0	0	0	0	0	0	0
0	0	0	0	0	0	0	0	0	0	0	0	0	0	0	0	0	0	0	0	0
0	0	0	0	0	0	0	0	0	0	0	0	0	0	0	0	0	0	0	0	0
0	0	0	0	0	0	0	0	0	0	0	0	0	0	0	0	0	0	0	0	0
0	0	0	0	0	0	0	0	0	0	0	0	0	0	0	0	0	0	0	0	0
0	0	0	0	0	0	0	0	0	0	0	0	0	0	0	0	0	0	0	0	0
0	0	0	0	0	0	0	0	0	0	0	0	0	0	0	0	0	0	0	0	0
0	0	0	0	0	0	0	0	0	0	0	0	0	0	0	0	0	0	0	0	0
0	0	0	0	0	0	0	0	0	0	0	0	0	0	0	0	0	0	0	0	0
0	0	0	0	0	0	0	0	0	0	0.01	0	0	0	0	0	0	0	0	0	0
0	0	0	0	0	0	0	0	0	0	0	0	0	0	0	0	0	0	0	0	0.01
0	0	0	0	0	0	0	0	0	0	0	0	0	0	0	0	0	0	0	0	0

0	0	0	0	0	0	0	0	0	0	0	0	0	0	0	0	0	0	0	0	0
0	0	0	0	0	0	0	0	0	0	0	0	0	0	0	0	0	0	0	0	0
0	0	0	0	0	0	0	0	0	0	0	0	0	0	0	0	0	0	0	0	0
0	0	0	0	0	0	0	0	0	0	0	0	0	0	0	0	0	0	0	0	0
0	0	0	0	0	0	0	0	0	0	0	0	0	0	0	0	0	0	0	0	0
0	0	0	0	0	0	0	0	0	0	0	0	0	0	0	0	0	0	0	0	0
0	0	0	0	0	0	0	0	0	0	0	0	0	0	0	0	0	0	0	0	0.01
0	0	0	0	0	0	0	0	0	0	0	0	0	0	0	0	0	0	0	0	0
0	0	0	0	0	0	0	0	0	0	0	0	0	0	0	0	0	0	0	0	0
0	0	0	0	0	0	0	0	0	0	0	0	0	0	0	0	0	0	0	0	0
0	0	0	0	0	0	0	0	0	0	0	0	0	0	0	0	0	0	0	0	0
0	0	0	0	0	0	0	0	0	0	0	0	0	0	0	0	0	0	0	0	0
0	0	0	0	0	0	0	0	0	0	0	0	0	0	0	0	0	0	0	0	0.01
0	0	0	0	0	0	0	0	0	0	0	0	0	0	0	0	0	0	0	0	0
0	0	0	0	0	0	0	0	0	0	0	0	0	0	0	0	0	0	0	0	0
0	0	0	0	0	0	0	0	0	0	0	0	0	0	0	0	0	0	0	0	0
0	0	0	0	0	0	0	0	0	0	0	0	0	0	0	0	0	0	0	0	0
0	0	0	0	0	0	0	0	0	0	0	0	0	0	0	0	0	0	0	0	0
0	0	0	0	0	0	0	0	0	0	0	0	0	0	0	0	0	0	0	0	0
0	0	0	0	0	0	0	0	0	0	0	0	0	0	0	0	0	0	0	0	0
0	0	0	0	0	0	0	0	0	0	0	0	0	0	0	0	0	0	0	0	0
0	0	0	0	0	0	0	0	0	0	0	0	0	0	0	0	0	0	0	0	0
0	0	0	0	0	0	0	0	0	0	0	0	0	0	0	0	0	0	0	0	0
0	0	0	0	0	0	0	0	0	0	0	0	0	0	0	0	0	0	0	0	0
0	0	0	0	0	0	0	0	0	0	0	0	0	0	0	0	0	0	0	0	0
0	0	0	0	0	0	0	0	0	0	0	0	0	0	0	0	0	0	0	0	0
0	0	0	0	0	0	0	0	0	0	0	0	0	0	0	0	0	0	0	0	0
0	0	0	0	0	0	0	0	0	0	0	0	0	0	0	0	0	0	0	0	0
0	0	0	0	0	0	0	0	0	0	0	0	0	0	0	0	0	0	0	0	0
0	0	0	0	0	0	0	0	0	0	0	0	0	0	0	0	0	0	0	0	0
0	0	0	0	0	0	0	0	0	0	0	0	0	0	0	0	0	0	0	0	0
0	0	0	0	0	0	0	0	0	0	0	0	0	0	0	0	0	0	0	0	0
0	0	0	0	0	0	0	0	0	0	0	0	0	0	0	0	0	0	0	0	0
0	0	0	0	0	0	0	0	0	0	0	0	0	0	0	0	0	0	0	0	0
0	0	0	0	0	0	0	0	0	0	0	0	0	0	0	0	0	0	0	0	0
0	0	0	0	0	0	0	0	0	0	0	0	0	0	0	0	0	0	0	0	0

0	0	0	0	0	0	0	0	0	0	0	0	0	0	0	0	0	0	0	0	0
0	0	0	0	0	0	0	0	0	0	0	0	0	0	0	0	0	0	0	0	0
0	0	0	0	0	0	0	0	0	0	0	0	0	0	0	0	0	0	0	0	0
0	0	0	0	0	0	0	0	0	0	0	0	0	0	0	0	0	0	0	0	0
0	0	0	0	0	0	0	0	0	0	0	0	0	0	0	0	0	0	0	0	0
0	0	0	0	0	0	0	0	0	0	0	0	0	0	0	0	0	0	0	0	0
0	0	0	0	0	0	0	0	0	0	0	0	0	0	0	0	0	0	0	0	0
0	0	0	0	0	0	0	0	0	0	0	0	0	0	0	0	0	0	0	0	0
0	0	0	0	0	0	0	0	0	0	0	0	0	0	0	0	0	0	0	0	0
0	0	0	0	0	0	0	0	0	0	0	0	0	0	0	0	0	0	0	0	0
0	0	0	0	0	0	0	0	0	0	0	0	0	0	0	0	0	0	0	0	0
0	0	0	0	0	0	0	0	0	0	0	0	0	0	0	0	0	0	0	0	0
0	0	0	0	0	0	0	0	0	0	0	0	0	0	0	0	0	0	0	0	0
0	0	0	0	0	0	0	0	0	0	0	0	0	0	0	0	0	0	0	0	0
0	0	0	0	0	0	0	0	0	0	0	0	0	0	0	0	0	0	0	0	0
0	0	0	0	0	0	0	0	0	0	0	0	0	0	0	0	0	0	0	0	0
0	0	0	0	0	0	0	0	0	0	0	0	0	0	0	0	0	0	0	0	0
0	0	0	0	0	0	0	0	0	0	0	0	0	0	0	0	0	0	0	0	0
0	0	0	0	0	0	0	0	0	0	0	0	0	0	0	0	0	0	0	0	0
0	0	0	0	0	0	0	0	0	0	0	0	0	0	0	0	0	0	0	0	0
0	0	0	0	0	0	0	0	0	0	0	0	0	0	0	0	0	0	0	0	0
0	0	0	0	0	0	0	0	0	0	0	0	0	0	0	0	0	0	0	0	0
0	0	0	0	0	0	0	0	0	0	0	0	0	0	0	0	0	0	0	0	0
0	0	0	0	0	0	0	0	0	0	0	0	0	0	0	0	0	0	0	0	0
0	0	0	0	0	0	0	0	0	0	0	0	0	0	0	0	0	0	0	0	0
0	0	0	0	0	0	0	0	0	0	0	0	0	0	0	0	0	0	0	0	0
0	0	0	0	0	0	0	0	0	0	0	0	0	0	0	0	0	0	0	0	0
0	0	0	0	0	0	0	0	0	0	0	0	0	0	0	0	0	0	0	0	0
0	0	0	0	0	0	0	0	0	0	0	0	0	0	0	0	0	0	0	0	0
0	0	0	0	0	0	0	0	0	0	0	0	0	0	0	0	0	0	0	0	0
0	0	0	0	0	0	0	0	0	0	0	0	0	0	0	0	0	0	0	0	0
0	0	0	0	0	0	0	0	0	0	0	0	0	0	0	0	0	0	0	0	0
0	0	0	0	0	0	0	0	0	0	0	0	0	0	0	0	0	0	0	0	0
0	0	0	0	0	0	0	0	0	0	0	0	0	0	0	0	0	0	0	0	0
0	0	0	0	0	0	0	0	0	0	0	0	0	0	0	0	0	0	0	0	0
0	0	0	0	0	0	0	0	0	0	0	0	0	0	0	0	0	0	0	0	0

0	0	0	0	0	0	0	0	0	0	0	0	0	0	0	0	0	0	0	0	0
0	0	0	0	0	0	0	0	0	0	0	0	0	0	0	0	0	0	0	0	0
0	0	0	0	0	0	0	0	0	0	0	0	0	0	0	0	0	0	0	0	0
0	0	0	0	0	0	0	0	0	0	0	0	0	0	0	0	0	0	0	0	0
0	0	0	0	0	0	0	0	0	0	0	0	0	0	0	0	0	0	0	0	0
0	0	0	0	0	0	0	0	0	0	0	0	0	0	0	0	0	0	0	0	0
0	0	0	0	0	0	0	0	0	0	0	0	0	0	0	0	0	0	0	0	0
0	0	0	0	0	0	0	0	0	0	0	0	0	0	0	0	0	0	0	0	0
0	0	0	0	0	0	0	0	0	0	0	0	0	0	0	0	0	0	0	0	0
0	0	0	0	0	0	0	0	0	0	0	0	0	0	0	0	0	0	0	0	0
0	0	0	0	0	0	0	0	0	0	0	0	0	0	0	0	0	0	0	0	0
0	0	0	0	0	0	0	0	0	0	0	0	0	0	0	0	0	0	0	0	0
0	0	0	0	0	0	0	0	0	0	0	0	0	0	0	0	0	0	0	0	0
0	0	0	0	0	0	0	0	0	0	0	0	0	0	0	0	0	0	0	0	0
0	0	0	0	0	0	0	0	0	0	0	0	0	0	0	0	0	0	0	0	0
0	0	0	0	0	0	0	0	0	0	0	0	0	0	0	0	0	0	0	0	0
0	0	0	0	0	0	0	0	0	0	0	0	0	0	0	0	0	0	0	0	0
0	0	0	0	0	0	0	0	0	0	0	0	0	0	0	0	0	0	0	0	0
0	0	0	0	0	0	0	0	0	0	0	0	0	0	0	0	0	0	0	0	0
0	0	0	0	0	0	0	0	0	0	0	0	0	0	0	0	0	0	0	0	0
0	0	0	0	0	0	0	0	0	0	0	0	0	0	0	0	0	0	0	0	0
0	0	0	0	0	0	0	0	0	0	0	0	0	0	0	0	0	0	0	0	0.01
0	0	0	0	0	0	0	0	0	0	0	0	0	0	0	0	0	0	0	0	0
0	0	0	0	0	0	0	0	0	0	0	0	0	0	0	0	0	0	0	0	0
0	0	0	0	0	0	0	0	0	0	0	0	0	0	0	0	0	0	0	0	0
0	0	0	0	0	0	0	0	0	0	0	0	0	0	0	0	0	0	0	0	0
0	0	0	0	0	0	0	0	0	0	0	0	0	0	0	0	0	0	0	0	0
0	0	0	0	0	0	0	0	0	0	0	0	0	0	0	0	0	0	0	0	0
0	0	0	0	0	0	0	0	0	0	0	0	0	0	0	0	0	0	0	0	0
0	0	0	0	0	0	0	0	0	0	0	0	0	0	0	0	0	0	0	0	0
0	0	0	0	0	0	0	0	0	0	0	0	0	0	0	0	0	0	0	0	0
0	0	0	0	0	0	0	0	0	0	0	0	0	0	0	0	0	0	0	0	0
0	0	0	0	0	0	0	0	0	0	0	0	0	0	0	0	0	0	0	0	0
0	0	0	0	0	0	0	0	0	0	0	0	0	0	0	0	0	0	0	0	0
0	0	0	0	0	0	0	0	0	0	0	0	0	0	0	0	0	0	0	0	0
0	0	0	0	0	0	0	0	0	0	0	0	0	0	0	0	0	0	0	0	0

0	0	0	0	0	0	0	0	0	0	0	0	0	0	0	0	0	0	0	0	0
0	0	0	0	0	0	0	0	0	0	0	0	0	0	0	0	0	0	0	0	0
0	0	0	0	0	0	0	0	0	0	0	0	0	0	0	0	0	0	0	0	0
0	0	0	0	0	0	0	0	0	0	0	0	0	0	0	0	0	0	0	0	0
0	0	0	0	0	0	0	0	0	0	0	0	0	0	0	0	0	0	0	0	0
0	0	0	0	0	0	0	0	0	0	0	0	0	0	0	0	0	0	0	0	0
0	0	0	0	0	0	0	0	0	0	0	0	0	0	0	0	0	0	0	0	0
0	0	0	0	0	0	0	0	0	0	0	0	0	0	0	0	0	0	0	0	0
0	0	0	0	0	0	0	0	0	0	0	0	0	0	0	0	0	0	0	0	0
0	0	0	0	0	0	0	0	0	0	0	0	0	0	0	0	0	0	0	0	0
0	0	0	0	0	0	0	0	0	0	0	0	0	0	0	0	0	0	0	0	0
0	0	0	0	0	0	0	0	0	0	0	0	0	0	0	0	0	0	0	0	0
0	0	0	0	0	0	0	0	0	0	0	0	0	0	0	0	0	0	0	0	0
0	0	0	0	0	0	0	0	0	0	0	0	0	0	0	0	0	0	0	0	0
0	0	0	0	0	0	0	0	0	0	0	0	0	0	0	0	0	0	0	0	0
0	0	0	0	0	0	0	0	0	0	0	0	0	0	0	0	0	0	0	0	0
0	0	0	0	0	0	0	0	0	0	0	0	0	0	0	0	0	0	0	0	0
0	0	0	0	0	0	0	0	0	0	0	0	0	0	0	0	0	0	0	0	0
0	0	0	0	0	0	0	0	0	0	0	0	0	0	0	0	0	0	0	0	0
0	0	0	0	0	0	0	0	0	0	0	0	0	0	0	0	0	0	0	0	0
0	0	0	0	0	0	0	0	0	0	0	0	0	0	0	0	0	0	0	0	0
0	0	0	0	0	0	0	0	0	0	0	0	0	0	0	0	0	0	0	0	0
0	0	0	0	0	0	0	0	0	0	0	0	0	0	0	0	0	0	0	0	0
0	0	0	0	0	0	0	0	0	0	0	0	0	0	0	0	0	0	0	0	0
0	0	0	0	0	0	0	0	0	0	0	0	0	0	0	0	0	0	0	0	0
0	0	0	0	0	0	0	0	0	0	0	0	0	0	0	0	0	0	0	0	0
0	0	0	0	0	0	0	0	0	0	0	0	0	0	0	0	0	0	0	0	0
0	0	0	0	0	0	0	0	0	0	0	0	0	0	0	0	0	0	0	0	0
0	0	0	0	0	0	0	0	0	0	0	0	0	0	0	0	0	0	0	0	0
0	0	0	0	0	0	0	0	0	0	0	0	0	0	0	0	0	0	0	0	0
0	0	0	0	0	0	0	0	0	0	0	0	0	0	0	0	0	0	0	0	0
0	0	0	0	0	0	0	0	0	0	0	0	0	0	0	0	0	0	0	0	0
0	0	0	0	0	0	0	0	0	0	0	0	0	0	0	0	0	0	0	0	0
0	0	0	0	0	0	0	0	0	0	0	0	0	0	0	0	0	0	0	0	0
0	0	0	0	0	0	0	0	0	0	0	0	0	0	0	0	0	0	0	0	0
0	0	0	0	0	0	0	0	0	0	0	0	0	0	0	0	0	0	0	0	0

0	0	0	0	0	0	0	0	0	0	0	0	0	0	0	0	0	0	0	0	0
0	0	0	0	0	0	0	0	0	0	0	0	0	0	0	0	0	0	0	0	0
0	0	0	0	0	0	0	0	0	0	0	0	0	0	0	0	0	0	0	0	0
0	0	0	0	0	0	0	0	0	0	0	0	0	0	0	0	0	0	0	0	0
0	0	0	0	0	0	0	0	0	0	0	0	0	0	0	0	0	0	0	0	0
0	0	0	0	0	0	0	0	0	0	0	0	0	0	0	0	0	0	0	0	0
0	0	0	0	0	0	0	0	0	0	0	0	0	0	0	0	0	0	0	0	0
0	0	0	0	0	0	0	0	0	0	0	0	0	0	0	0	0	0	0	0	0
0	0	0	0	0	0	0	0	0	0	0	0	0	0	0	0	0	0	0	0	0
0	0	0	0	0	0	0	0	0	0	0	0	0	0	0	0	0	0	0	0	0
0	0	0	0	0	0	0	0	0	0	0	0	0	0	0	0	0	0	0	0	0
0	0	0	0	0	0	0	0	0	0	0	0	0	0	0	0	0	0	0	0	0
0	0	0	0	0	0	0	0	0	0	0	0	0	0	0	0	0	0	0	0	0
0	0	0	0	0	0	0	0	0	0	0	0	0	0	0	0	0	0	0	0	0
0	0	0	0	0	0	0	0	0	0	0	0	0	0	0	0	0	0	0	0	0
0	0	0	0	0	0	0	0	0	0	0	0	0	0	0	0	0	0	0	0	0
0	0	0	0	0	0	0	0	0	0	0	0	0	0	0	0	0	0	0	0	0
0	0	0	0	0	0	0	0	0	0	0	0	0	0	0	0	0	0	0	0	0
0	0	0	0	0	0	0	0	0	0	0	0	0	0	0	0	0	0	0	0	0
0	0	0	0	0	0	0	0	0	0	0	0	0	0	0	0	0	0	0	0	0
0	0	0	0	0	0	0	0	0	0	0	0	0	0	0	0	0	0	0	0	0
0	0	0	0	0	0	0	0	0	0	0	0	0	0	0	0	0	0	0	0	0
0	0	0	0	0	0	0	0	0	0	0	0	0	0	0	0	0	0	0	0	0
0	0	0	0	0	0	0	0	0	0	0	0	0	0	0	0	0	0	0	0	0
0	0	0	0	0	0	0	0	0	0	0	0	0	0	0	0	0	0	0	0	0
0	0	0	0	0	0	0	0	0	0	0	0	0	0	0	0	0	0	0	0	0
0	0	0	0	0	0	0	0	0	0	0	0	0	0	0	0	0	0	0	0	0
0	0	0	0	0	0	0	0	0	0	0	0	0	0	0	0	0	0	0	0	0
0	0	0	0	0	0	0	0	0	0	0	0	0	0	0	0	0	0	0	0	0
0	0	0	0	0	0	0	0	0	0	0	0	0	0	0	0	0	0	0	0	0
0	0	0	0	0	0	0	0	0	0	0	0	0	0	0	0	0	0	0	0	0
0	0	0	0	0	0	0	0	0	0	0	0	0	0	0	0	0	0	0	0	0
0	0	0	0	0	0	0	0	0	0	0	0	0	0	0	0	0	0	0	0	0
0	0	0	0	0	0	0	0	0	0	0	0	0	0	0	0	0	0	0	0	0
0	0	0	0	0	0	0	0	0	0	0	0	0	0	0	0	0	0	0	0	0
0	0	0	0	0	0	0	0	0	0	0	0	0	0	0	0	0	0	0	0	0

0	0	0	0	0	0	0	0	0	0	0	0	0	0	0	0	0	0	0	0	0
0	0	0	0	0	0	0	0	0	0	0	0	0	0	0	0	0	0	0	0	0
0	0	0	0	0	0	0	0	0	0	0	0	0	0	0	0	0	0	0	0	0
0	0	0	0	0	0	0	0	0	0	0	0	0	0	0	0	0	0	0	0	0
0	0	0	0	0	0	0	0	0	0	0	0	0	0	0	0	0	0	0	0	0
0	0	0	0	0	0	0	0	0	0	0	0	0	0	0	0	0	0	0	0	0
0	0	0	0	0	0	0	0	0	0	0	0	0	0	0	0	0	0	0	0	0
0	0	0	0	0	0	0	0	0	0	0	0	0	0	0	0	0	0	0	0	0
0	0	0	0	0	0	0	0	0	0	0	0	0	0	0	0	0	0	0	0	0
0	0	0	0	0	0	0	0	0	0	0	0	0	0	0	0	0	0	0	0	0
0	0	0	0	0	0	0	0	0	0	0	0	0	0	0	0	0	0	0	0	0
0	0	0	0	0	0	0	0	0	0	0	0	0	0	0	0	0	0	0	0	0
0	0	0	0	0	0	0	0	0	0	0	0	0	0	0	0	0	0	0	0	0
0	0	0	0	0	0	0	0	0	0	0.01	0	0	0	0	0	0	0	0.01	0	0
0	0	0	0	0	0	0	0	0	0	0	0	0	0	0	0	0	0.02	0	0.01	0
0	0	0	0.01	0	0	0	0	0	0	0	0	0	0	0	0	0	0	0	0	0
0	0	0	0	0	0	0	0	0	0	0	0	0	0	0	0	0	0	0	0	0
0	0	0	0	0	0	0	0	0	0	0	0	0	0	0	0	0	0	0	0	0
0	0	0	0	0	0	0	0	0	0	0	0	0	0	0	0	0	0	0	0	0
0	0	0	0	0	0	0	0	0	0	0	0	0	0	0	0	0	0	0	0	0
0	0	0	0	0	0	0	0	0	0	0	0	0	0	0	0	0	0	0	0	0
0	0	0	0	0	0	0	0	0	0	0	0	0	0	0	0	0	0	0	0	0
0	0	0	0	0	0	0	0	0	0	0	0	0	0	0	0	0	0	0	0	0
0	0	0	0	0	0	0	0	0	0	0	0	0	0	0	0	0	0	0	0	0
0	0	0	0	0	0	0	0	0	0	0	0	0	0	0	0	0	0	0	0	0
0	0	0	0	0	0	0	0	0	0	0	0	0	0	0	0	0	0	0	0	0
0	0	0	0	0	0	0	0	0	0	0	0	0	0	0	0	0	0	0	0	0
0	0	0	0	0	0	0	0	0	0	0	0	0	0	0	0	0	0	0	0	0
0	0	0	0	0	0	0	0	0	0	0	0	0	0	0	0	0	0	0	0	0
0	0	0	0	0	0	0	0	0	0	0	0	0	0	0	0	0	0	0	0	0
0	0	0	0	0	0	0	0	0	0	0	0	0	0	0	0	0	0	0	0	0
0	0	0	0	0	0	0	0	0	0	0	0	0	0	0	0	0	0	0	0	0
0	0	0	0	0	0	0	0	0	0	0	0	0	0	0	0	0	0	0	0	0
0	0	0	0	0	0	0	0	0	0	0	0	0	0	0	0	0	0	0	0	0
0	0	0	0	0	0	0	0	0	0	0	0	0	0	0	0	0	0	0	0	0
0	0	0	0	0	0	0	0	0	0	0	0	0	0	0	0	0	0	0	0	0

0	0	0	0	0	0	0	0	0	0	0	0	0	0	0	0	0	0	0	0	0
0	0	0	0	0	0	0	0	0	0	0	0	0	0	0	0	0	0	0	0	0
0	0	0	0	0	0	0	0	0	0	0	0	0	0	0	0	0	0	0	0	0
0	0	0	0	0	0	0	0	0	0	0	0	0	0	0	0	0	0	0	0	0
0	0	0	0	0	0	0	0	0	0.01	0.01	0.01	0	0	0	0	0	0.02	0.02	0	0
0	0	0.01	0	0.01	0.01	0	0	0.02	0.02	0	0	0	0.01	0	0.01	0	0	0	0	0
0	0	0	0	0	0	0	0	0	0	0	0	0	0	0	0	0	0	0	0	0
0.01	0	0.01	0.01	0	0.01	0	0	0.01	0.01	0.01	0	0	0.01	0	0	0	0.01	0	0.01	0
0	0	0	0	0	0	0	0	0	0	0	0	0	0	0	0	0	0	0	0	0
0	0	0	0	0	0.01	0	0	0	0.01	0	0	0	0	0	0	0	0	0	0	0
0	0	0	0	0	0	0	0	0	0	0	0	0	0	0	0	0	0	0	0	0
0	0	0	0	0	0	0	0	0	0	0	0.01	0	0	0	0	0	0	0.01	0	0.01
0.11	0	0.09	0.1	0.01	0.02	0	0	0	0	0	0	0	0	0	0	0	0.01	0.01	0	0.01

miR_Th	miR_U	miR_U	miR_U	miR_U	miR_Ut	miR_Stomach
0.08	0.03	0.06	0.07	0.08	0.03	0.01
0.08	0.06	0.07	0.06	0.14	0.11	0.01
0.04	0.01	0	0.01	0.01	0.04	0
0	0	0	0.01	0	0	0
0.01	0	0.01	0.01	0.01	0	0
0.03	0.01	0.02	0.01	0.02	0.01	0.01
0.01	0	0	0	0	0	0.01
0.05	0.02	0	0.01	0.01	0.01	0
0	0	0	0	0	0	0
0.01	0	0.01	0.01	0	0	0
0	0	0	0	0	0	0
0	0	0	0	0.01	0	0
0	0	0	0	0	0	0
0	0	0	0	0	0	0
0	0	0	0	0	0	0
0	0	0	0	0	0	0
0	0	0	0	0	0	0
0	0	0	0	0	0	0
0	0	0	0	0	0	0
0.01	0	0	0	0	0	0
0	0	0	0	0	0	0
0	0	0	0	0	0	0
0	0	0	0	0	0	#N/D
0.01	0	0	0	0	0	0
0.05	0.01	0.02	0.03	0.01	0.02	0
0.06	0	0	0	0	0.04	0
0	0	0	0	0	0	#N/D
0	0	0	0	0	0	0
0	0	0	0	0	0	0
0	0	0	0	0	0	0
0	0	0	0	0	0	0
0	0	0	0	0	0	0
0	0	0	0	0	0	0
0	0.01	0.01	0	0	0.01	0
0	0	0	0	0	0	0

0	0	0	0	0	0	0
0	0	0	0	0	0	0
0	0	0	0	0	0	0
0	0	0	0	0	0	0
0	0	0	0	0	0	0
0	0	0	0	0	0	0
0	0.01	0.01	0.01	0	0	0
0	0	0	0	0	0	0
0.01	0	0	0	0	0	0
0	0	0	0	0	0	0
0	0	0	0	0	0	0
0	0	0	0	0	0	0.01
0	0.01	0.01	0.01	0.03	0	0
0	0	0	0	0	0	0.01
0	0.03	0.01	0.01	0	0	0
0	0.03	0.02	0.02	0	0	0
0.07	0.22	0.19	0.2	0.25	0.31	0.01
0	0	0	0	0	0	0
0.01	0.01	0	0	0	0.07	0.03
0	0.01	0	0	0	0	0
0	0	0	0	0	0	0
0	0	0	0	0	0	0
0	0	0	0	0	0	0
0	0	0	0	0	0	0
0	0	0	0	0	0	0.07
0	0	0	0	0	0	0
0	0	0	0	0	0	0
0	0	0	0	0	0	0
0	0	0	0	0	0	0
0	0	0	0	0	0	0
0	0	0	0	0	0.01	0
0	0	0	0	0	0	0
0	0	0	0	0	0	0
0	0	0	0	0	0	0
0.01	0.03	0.03	0.02	0.02	0	0
0	0	0	0.01	0	0	0

0.04	0.03	0.06	0.04	0.06	0.04	0
0	0	0	0	0	0	0.01
0.01	0.01	0.01	0	0	0	0
0	0	0	0	0	0	0
0	0	0	0	0	0	0
0	0	0	0	0	0	0
0	0	0	0	0	0	0
0	0	0	0	0	0	0
0	0	0	0	0	0	0
0	0	0	0	0	0	0
0	0	0	0	0	0	0
0	0	0	0	0	0	0
0	0	0	0	0	0	0
0	0	0	0	0	0	0
0	0	0	0	0	0	0
0	0	0	0	0	0	0
0	0	0	0	0	0	0
0	0	0	0	0	0	0
0.01	0	0.01	0.01	0.01	0	0.01
0	0	0	0	0	0	0.04
0	0.01	0	0	0	0	0
0	0.01	0	0	0	0	0
0	0	0	0	0	0	0
0	0	0	0	0	0	0
0.01	0	0	0	0	0	0
0	0	0	0	0	0	0
0	0.01	0	0	0	0	0
0	0	0	0	0	0	0
0	0	0	0	0	0	0
0	0	0	0	0	0.01	0
0	0	0	0	0	0.01	0
0	0	0	0	0	0	0
0	0	0	0	0	0	0.01
0	0	0	0	0	0	0
0	0	0	0	0	0	0.02
0	0	0	0	0	0	0.02

0	0	0	0	0	0	0.01
0.04	0	0	0	0	0	0.01
0	0	0	0	0	0	0
0	0	0	0	0	0	0
0	0	0	0	0	0	0
0	0	0	0	0	0	0
0	0	0	0	0	0	0
0	0	0	0	0	0	0
0	0	0	0	0	0	0
0	0	0	0	0	0	0
0	0	0	0	0	0	0
0.01	0.05	0.05	0.03	0.04	0.02	0.11
0	0	0	0	0	0	0
0	0	0	0	0	0	0
0	0	0	0	0	0	0
0	0	0	0	0	0	0
0	0	0	0	0	0	0
0	0	0	0	0	0	0
0	0	0	0	0	0	0
0	0	0	0	0	0	0
0	0	0	0	0	0	0
0	0	0	0	0	0	0
0	0	0	0	0	0	0
0	0.05	0.03	0.04	0.02	0.02	0
0	0	0	0	0	0	0
0	0	0	0	0	0	0
0	0	0	0	0	0	0
0	0	0	0.01	0	0	0
0	0.01	0.01	0	0	0	0
0	0	0	0	0	0	0.01
0	0	0	0	0	0	0
0.01	0.02	0.02	0.02	0.01	0.01	0.01
0.03	0	0	0	0.01	0.03	0.01
0.01	0.03	0.03	0.05	0.03	0.01	0.06
0.01	0	0	0	0	0	0
0.04	0.01	0.01	0.01	0.01	0.03	0.01

0.01	0.01	0	0	0	0	0
0.01	0.08	0.09	0.1	0.07	0.01	0
0.02	0	0.01	0.01	0	0	0
0	0	0	0	0.01	0	0
0	0.01	0.01	0.01	0.01	0	0
0	0	0	0	0	0	0
0	0	0	0	0	0	0
0	0	0	0	0	0	0
0	0	0	0	0	0	0
0	0	0	0	0	0	0
0.02	0	0.01	0.01	0	0.04	0.06
0	0.01	0.01	0	0	0	0.05
0	0	0	0	0	0	0.11
0	0	0	0	0	0	0
0	0	0	0	0	0	0
0	0	0	0	0	0	0
0	0	0	0	0	0	0
0	0	0	0	0	0	0
0	0	0	0	0	0	0
0	0	0	0	0	0	0
0.01	0	0	0	0.01	0	0
0	0	0	0	0	0	0.01
0.02	0	0	0	0	0	0
0.02	0.01	0.01	0	0.01	0.01	0.01
0	0	0	0	0	0	0.01
0	0	0	0	0	0	0.03
0	0	0	0	0	0	0
0	0	0	0	0	0	0
0	0	0	0	0	0	0
0	0	0	0	0	0	0
0	0	0	0	0	0	0
0	0	0	0	0	0	0
0	0	0	0	0	0	0
0	0	0	0	0	0	0
0	0	0	0	0	0	0
0	0	0	0	0	0	0

0	0	0	0	0	0	0
0	0	0	0	0	0	0
0	0	0	0	0	0	0
0	0	0	0	0	0	0
0	0	0	0	0	0	0
0	0	0	0	0	0	0
0	0	0	0	0	0	0
0	0	0	0	0	0	0
0	0	0	0	0	0	0
0	0	0	0	0	0	0
0	0	0	0	0	0	0
0	0	0	0	0	0	0
0	0	0	0	0	0	0
0	0	0	0	0	0	0
0	0	0	0	0	0	0
0	0.01	0	0	0	0	0
0	0	0	0	0	0	0
0	0	0	0	0	0	0
0	0	0	0	0	0	0
0	0	0	0	0	0	0.01
0	0	0	0	0	0	0
0	0	0	0	0	0	0
0	0	0	0	0	0	0
0	0	0	0	0	0	#N/D
0	0	0	0	0	0	0
0	0	0	0	0	0	#N/D
0	0	0	0	0	0	0
0	0	0	0	0	0	0
0	0	0	0	0	0	0
0	0	0	0	0	0	0
0	0	0.01	0.02	0.02	0	0
0	0	0	0	0	0	0
0	0	0	0	0	0	0
0	0	0	0	0	0	0
0	0	0	0	0	0	0
0	0	0	0	0	0	0

0	0	0	0	0	0	0
0	0	0	0	0	0	0
0	0	0	0	0	0	0
0	0	0	0	0	0	0
0	0	0	0	0	0	0.01
0	0	0	0.01	0	0	0
0	0	0	0	0	0	0
0	0	0	0.01	0	0	0
0	0	0	0	0	0	0.01
0	0	0	0	0	0	0
0	0	0	0	0	0	0
0	0	0	0	0	0	0
0	0	0	0	0	0	0
0	0	0	0	0	0	0
0	0	0	0	0	0	0
0	0	0	0	0	0	0
0	0	0	0	0	0	0
0	0	0	0	0	0	0
0	0	0	0	0	0	0
0	0	0	0	0	0	0
0	0	0	0	0	0	0
0	0	0	0	0	0	0
0	0	0	0	0	0	0
0.01	0	0	0	0	0	#N/D
0	0	0	0	0	0	0
0	0.01	0.01	0	0	0.01	0
0	0	0	0	0	0	0
0	0	0	0	0	0	0
0	0	0	0	0	0	0
0	0	0	0	0	0	0
0	0	0	0	0	0	0
0	0	0	0	0	0	0
0	0	0	0	0	0	0
0	0	0	0	0	0	0
0	0	0	0	0	0	0
0	0	0	0	0	0	0

0	0	0	0	0	0	0
0.02	0	0	0	0	0	0.04
0	0	0	0	0	0	0
0	0	0	0	0	0	0
0	0	0	0	0	0	0
0	0	0	0	0	0	#N/D
0	0	0	0	0	0	0
0	0	0	0	0	0	0
0	0	0	0	0	0	0
0	0	0	0	0	0	0
0	0	0	0	0	0	0
0	0	0	0	0	0	0
0	0	0	0	0	0	0
0	0	0	0	0	0	0
0	0	0	0	0	0	0
0	0	0	0	0	0	0
0	0	0	0	0	0	0
0	0	0	0	0	0	0
0	0	0	0	0	0	0
0	0	0	0	0	0	0
0	0	0	0	0	0	0
0	0	0	0	0	0	0
0	0	0	0	0	0	0
0	0	0	0	0	0	0
0	0	0	0	0	0	0
0	0	0	0	0	0	0
0.01	0	0	0	0	0	0
0	0	0	0	0	0	0
0	0	0	0	0	0	0
0	0	0	0	0	0	0
0	0	0	0	0	0	0
0	0	0	0	0	0	0
0	0	0	0	0	0	0
0	0	0	0	0	0	0
0	0	0	0	0	0	0
0	0	0	0	0	0	0

0	0	0	0	0	0	0
0	0	0	0	0	0	0
0	0	0	0	0	0	0
0	0	0	0	0	0	0
0	0	0	0	0	0	0
0	0	0	0	0	0	0
0	0	0	0	0	0	0
0	0	0	0	0	0	0
0	0	0	0	0	0	0
0	0	0	0	0	0	0
0	0	0	0	0	0	0
0	0	0	0	0	0	0
0	0	0	0	0	0	0
0	0	0	0	0	0	0
0	0	0	0	0	0	0
0	0	0	0	0	0	0
0	0	0	0	0	0	0
0	0	0	0	0	0	0
0	0	0	0	0	0	0
0	0	0	0	0	0	0
0	0	0	0	0	0	0
0	0	0	0	0	0	0
0	0	0	0	0	0	0
0	0	0	0	0	0	0
0	0	0	0	0	0	0
0	0	0	0	0	0	0
0	0	0	0	0	0	0
0	0	0	0	0	0	0
0	0	0	0	0	0	0
0	0	0	0	0	0	0
0	0	0	0	0	0	0
0	0	0	0	0	0	0
0	0	0	0	0	0	0
0	0	0	0	0	0	0
0	0	0	0	0	0	0
0	0	0	0	0	0	0

0	0	0	0	0	0	0
0	0	0	0	0	0	0
0	0	0	0	0	0	0
0	0	0	0	0	0	0
0	0	0	0	0	0	0
0	0	0	0	0	0	0
0	0	0	0	0	0	0
0	0	0	0	0	0	0
0	0	0	0	0	0	0
0	0	0	0	0	0	0
0	0	0	0	0	0	0
0	0	0	0	0	0	0
0	0	0	0	0	0	0
0	0	0	0	0	0	0
0	0	0	0	0	0	0
0	0	0	0	0	0	#N/D
0	0	0	0	0	0	0
0	0	0	0	0	0	0
0	0	0	0	0	0	0
0	0	0	0	0	0	#N/D
0	0	0	0	0	0	0
0	0	0	0	0	0	0
0	0	0	0	0	0	0
0	0	0	0	0	0	0
0	0	0	0	0	0	0
0	0	0	0	0	0	0
0	0	0	0	0	0	0
0	0	0	0	0	0	0
0	0	0	0	0	0	0
0	0	0	0	0	0	0
0	0	0	0	0	0	0
0	0	0	0	0	0	0
0	0	0	0	0	0	0
0	0	0	0	0	0	0
0	0	0	0	0	0	0
0	0	0	0	0	0	0

0	0	0	0	0	0	0
0	0	0	0	0	0	0
0	0	0	0	0	0	0
0	0	0	0	0	0	0
0	0	0	0	0	0	0
0	0	0	0	0	0	0
0	0	0	0	0	0	0
0	0	0	0	0	0	0
0	0	0	0	0	0	0
0	0	0	0	0	0	0
0	0	0	0	0	0	0
0	0	0	0	0	0	0
0	0	0	0	0	0	0
0	0	0	0	0	0	0
0	0	0	0	0	0	0
0	0	0	0	0	0	0
0	0	0	0	0	0	0
0	0	0	0	0	0	0
0	0	0	0	0	0	0
0	0	0	0	0	0	0
0	0	0	0	0	0	0
0	0	0	0	0	0	0
0	0	0	0	0	0	0
0	0	0	0	0	0	0
0	0	0	0	0	0	0
0	0	0	0	0	0	0
0	0	0	0	0	0	0
0	0	0	0	0	0	0
0	0	0	0	0	0	0
0	0	0	0	0	0	0
0	0	0	0	0	0	0
0	0	0	0	0	0	0
0	0	0	0	0	0	0
0	0	0	0	0	0	0
0	0	0	0	0	0	0
0	0	0	0	0	0	0

0	0	0	0	0	0	0
0	0	0	0	0	0	0
0	0	0	0	0	0	0
0	0	0	0	0	0	0
0	0	0	0	0	0	0
0	0	0	0	0	0	0
0	0	0	0	0	0	0
0	0	0	0	0	0	0
0	0	0	0	0	0	0
0	0	0	0	0	0	0
0	0	0	0	0	0	0
0	0	0	0	0	0	0
0	0	0	0	0	0	0
0	0	0	0	0	0	0
0	0	0	0	0	0	0
0	0	0	0	0	0	0
0	0	0	0	0	0	0
0	0	0	0	0	0	0
0	0	0	0	0	0	0
0	0	0	0	0	0	0
0	0	0	0	0	0	0
0	0	0	0	0	0	0
0	0	0	0	0	0	0
0	0	0	0	0	0	0
0	0	0	0	0	0	0
0	0	0	0	0	0	0
0	0	0	0	0	0	0
0	0	0	0	0	0	0
0	0	0	0	0	0	0
0	0	0	0	0	0	0
0	0	0	0	0	0	0
0	0	0	0	0	0	0
0	0	0	0	0	0	0
0	0	0	0	0	0	0
0	0	0	0	0	0	0
0	0	0	0	0	0	0

0	0	0	0	0	0	0
0	0	0	0	0	0	0
0	0	0	0	0	0	0
0	0	0	0	0	0	0
0	0	0	0	0	0	0
0	0	0	0	0	0	0
0	0	0	0	0	0	0
0	0	0	0	0	0	0
0	0	0	0	0	0	0
0	0	0	0	0	0	0
0	0	0	0	0	0	0
0	0	0	0	0	0	0
0	0	0	0	0	0	0
0	0	0	0	0	0	0
0	0	0	0	0	0	0
0	0	0	0	0	0	0
0	0	0	0	0	0	0
0	0	0	0	0	0	0
0	0	0	0	0	0	0
0	0	0	0	0	0	0
0	0	0	0	0	0	0
0	0	0	0	0	0	0
0	0	0	0	0	0	0
0	0	0	0	0	0	0
0	0	0	0	0	0	0
0	0	0	0	0	0	0
0	0	0	0	0	0	0
0	0	0	0	0	0	0
0	0	0	0	0	0	0
0	0	0	0	0	0	0
0	0	0	0	0	0	0
0	0	0	0	0	0	0
0	0	0	0	0	0	0
0	0	0	0	0	0	0
0	0	0	0	0	0	0
0	0	0	0	0	0	0

0	0	0	0	0	0	0
0	0	0	0	0	0	0
0	0	0	0	0	0	0
0	0	0	0	0	0	0
0	0	0	0	0	0	0
0	0	0	0	0	0	0
0	0	0	0	0	0	0
0	0	0	0	0	0	0
0	0	0	0	0	0	0
0	0	0	0	0	0	0
0	0	0	0	0	0	0
0	0	0	0	0	0	0
0	0	0	0	0	0	0
0	0	0	0	0	0	0
0	0	0	0	0	0	0
0	0	0	0	0	0	0
0	0	0	0	0	0	0
0	0	0	0	0	0	0
0	0	0	0	0	0	0
0	0	0	0	0	0	0
0	0	0	0	0	0	0
0	0	0	0	0	0	0
0	0	0	0	0	0	0
0	0	0	0	0	0	0
0	0	0	0	0	0	0
0	0	0	0	0	0	0
0	0	0	0	0	0	0
0	0	0	0	0	0	0
0	0	0	0	0	0	0
0	0	0	0	0	0	0
0	0	0	0	0	0	0
0	0	0	0	0	0	0
0	0	0	0	0	0	0
0	0	0	0	0	0	0
0	0	0	0	0	0	0
0	0	0	0	0	0	0

0	0	0	0	0	0	0
0	0	0	0	0	0	0
0	0	0	0	0	0	0
0	0	0	0	0	0	0
0	0.01	0	0	0	0	0
0	0	0	0	0	0	0.01
0	0	0	0	0	0	0
0	0	0	0	0	0	0.01
0	0	0	0	0	0	0
0	0	0	0	0	0	0
0	0	0	0	0	0	0
0.02	0	0	0	0	0.03	0
0.01	0	0.01	0.01	0	0	0

hsa-21	9079	45620	0.49	10.8	9079	45620	0.109	0	0	0	0
hsa-29c	4481	44413	0.47	10.52	4481	44413	0.106	0	0	0	0
hsa-148a	5811	27265	0.29	6.46	5811	27265	0.065	0	0	0	0
hsa-29a	4311	25271	0.27	5.98	4311	25271	0.060	0	0	0	0
hsa-24	4884	24245	0.26	5.74	4884	24245	0.058	0	0	0	0
hsa-29b	5624	20401	0.22	4.83	5624	20401	0.049	0	0	0	0
hsa-1308	1162	18177	0.19	4.3	1162	18177	0.043	0	0	0	0
hsa-192	3538	16507	0.18	3.91	3538	16507	0.039	0	0	0	0
hsa-451	3667	15064	0.16	3.57	3667	15064	0.036	0	0	0	0
hsa-145	2385	12109	0.13	2.87	2385	12109	0.029	0	0	0	0
hsa-31	3522	11947	0.13	2.83	3522	11947	0.029	0	0	0	0
hsa-200a	2341	9777	0.1	2.32	2341	9777	0.023	0	0	0	0
hsa-19b	1863	8716	0.09	2.06	1863	8716	0.021	0	0	0	0
hsa-26a	2326	6257	0.07	1.48	2326	6257	0.015	0	0	0	0
hsa-200b	1739	5883	0.06	1.39	1739	5883	0.014	0	0	0	0
hsa-let-7b	2487	5326	0.06	1.26	2487	5326	0.013	0	0	0	0
hsa-143	1792	4672	0.05	1.11	1792	4672	0.011	0	0	0	0
hsa-191	1262	4514	0.05	1.07	1262	4514	0.011	0	0	0	0
hsa-let-7f	1619	3972	0.04	0.94	1619	3972	0.009	0	0	0	0
hsa-23b	556	3846	0.04	0.91	556	3846	0.009	0	0	0	0
hsa-378	1640	3753	0.04	0.89	1640	3753	0.009	0	0	0	0
hsa-141	920	3127	0.03	0.74	920	3127	0.007	0	0	0	0
hsa-199b	1294	3127	0.03	0.74	1294	3127	0.007	0	0	0	0
hsa-17	1081	2997	0.03	0.71	1081	2997	0.007	0	0	0	0
hsa-375	1612	2928	0.03	0.69	1612	2928	0.007	0	0	0	0
hsa-93	1214	2748	0.03	0.65	1214	2748	0.007	0	0	0	0
hsa-30b	662	2724	0.03	0.65	662	2724	0.007	0	0	0	0
hsa-223	520	2706	0.03	0.64	520	2706	0.006	0	0	0	0
hsa-let-7g	1157	2654	0.03	0.63	1157	2654	0.006	0	0	0	0
hsa-23a	481	2603	0.03	0.62	481	2603	0.006	0	0	0	0
hsa-let-7a	526	2510	0.03	0.59	526	2510	0.006	0	0	0	0
hsa-34a	1085	2387	0.03	0.57	1085	2387	0.006	0	0	0	0
hsa-200c	978	2237	0.02	0.53	978	2237	0.005	0	0	0	0
hsa-30e	1355	2225	0.02	0.53	1355	2225	0.005	0	0	0	0
hsa-92a	1012	2215	0.02	0.53	1012	2215	0.005	0	0	0	0
hsa-30d	1070	2182	0.02	0.52	1070	2182	0.005	0	0	0	0
hsa-140	947	2163	0.02	0.51	947	2163	0.005	0	0	0	0
hsa-125a	621	2067	0.02	0.49	621	2067	0.005	0	0	0	0
hsa-660	957	1842	0.02	0.44	957	1842	0.004	0	0	0	0
hsa-199a	833	1837	0.02	0.44	833	1837	0.004	0	0	0	0
hsa-19a	559	1629	0.02	0.39	559	1629	0.004	0	0	0	0
hsa-26b	759	1547	0.02	0.37	759	1547	0.004	0	0	0	0
hsa-99a	817	1514	0.02	0.36	817	1514	0.004	0	0	0	0
hsa-15b	934	1504	0.02	0.36	934	1504	0.004	0	0	0	0
hsa-let-7i	756	1428	0.02	0.34	756	1428	0.003	0	0	0	0
hsa-429	552	1398	0.02	0.33	552	1398	0.003	0	0	0	0
hsa-181a	953	1387	0.02	0.33	953	1387	0.003	0	0	0	0
hsa-484	493	1286	0.01	0.31	493	1286	0.003	0	0	0	0
hsa-151	629	1276	0.01	0.3	629	1276	0.003	0	0	0	0
hsa-126	786	1180	0.01	0.28	786	1180	0.003	0	0	0	0
hsa-30c	286	1154	0.01	0.27	286	1154	0.003	0	0	0	0
hsa-195	578	1129	0.01	0.27	578	1129	0.003	0	0	0	0
hsa-574	813	1122	0.01	0.27	813	1122	0.003	0	0	0	0
hsa-27a	518	1102	0.01	0.26	518	1102	0.003	0	0	0	0
hsa-424	490	970	0.01	0.23	490	970	0.002	0	0	0	0
hsa-210	599	967	0.01	0.23	599	967	0.002	0	0	0	0
hsa-125b	425	962	0.01	0.23	425	962	0.002	0	0	0	0
hsa-22	495	943	0.01	0.22	495	943	0.002	0	0	0	0

hsa-221	601	931	0.01	0.22	601	931	0.002	0	0	0	0
hsa-15a	669	925	0.01	0.22	669	925	0.002	0	0	0	0
hsa-1826	509	904	0.01	0.21	509	904	0.002	0	0	0	0
hsa-30a	509	798	0.01	0.19	509	798	0.002	0	0	0	0
hsa-338	400	777	0.01	0.18	400	777	0.002	0	0	0	0
hsa-150	392	756	0.01	0.18	392	756	0.002	0	0	0	0
hsa-20a	384	697	0.01	0.17	384	697	0.002	0	0	0	0
hsa-181b	433	695	0.01	0.17	433	695	0.002	0	0	0	0
hsa-let-7d	374	687	0.01	0.16	374	687	0.002	0	0	0	0
hsa-590	333	667	0.01	0.16	333	667	0.002	0	0	0	0
hsa-203	394	644	0.01	0.15	394	644	0.002	0	0	0	0
hsa-222	394	639	0.01	0.15	394	639	0.002	0	0	0	0
hsa-204	349	609	0.01	0.14	349	609	0.001	0	0	0	0
hsa-25	318	580	0.01	0.14	318	580	0.001	0	0	0	0
hsa-186	346	574	0.01	0.14	346	574	0.001	0	0	0	0
has-7	380	573	0.01	0.14	380	573	0.001	0	0	0	0
hsa-365	192	504	0.01	0.12	192	504	0.001	0	0	0	0
hsa-let-7e	336	481	0.01	0.11	336	481	0.001	0	0	0	0
hsa-28	321	472	0.01	0.11	321	472	0.001	0	0	0	0
hsa-27b	220	452	0.01	0.11	220	452	0.001	0	0	0	0
hsa-532	310	448	0.01	0.11	310	448	0.001	0	0	0	0
hsa-130a	299	445	0.01	0.11	299	445	0.001	0	0	0	0
hsa-574	248	428	0.01	0.1	248	428	0.001	0	0	0	0
hsa-425	257	405	0	0.1	257	405	0.001	0	0	0	0
hsa-16	354	402	0	0.1	354	402	0.001	0	0	0	0
hsa-148b	196	372	0	0.09	196	372	0.001	0	0	0	0
hsa-193a	196	361	0	0.09	196	361	0.001	0	0	0	0
hsa-331	273	359	0	0.09	273	359	0.001	0	0	0	0
hsa-138	215	350	0	0.08	215	350	0.001	0	0	0	0
hsa-let-7c	178	341	0	0.08	178	341	0.001	0	0	0	0
hsa-182	202	310	0	0.07	202	310	0.001	0	0	0	0
hsa-193b	215	307	0	0.07	215	307	0.001	0	0	0	0
hsa-497	183	303	0	0.07	183	303	0.001	0	0	0	0
hsa-423	204	298	0	0.07	204	298	0.001	0	0	0	0
hsa-339	178	294	0	0.07	178	294	0.001	0	0	0	0
hsa-140	213	293	0	0.07	213	293	0.001	0	0	0	0
hsa-142	245	293	0	0.07	245	293	0.001	0	0	0	0
hsa-362	165	293	0	0.07	165	293	0.001	0	0	0	0
hsa-345	256	286	0	0.07	256	286	0.001	0	0	0	0
hsa-146b	188	283	0	0.07	188	283	0.001	0	0	0	0
hsa-361	190	282	0	0.07	190	282	0.001	0	0	0	0
hsa-155	200	254	0	0.06	200	254	0.001	0	0	0	0
hsa-582	151	247	0	0.06	151	247	0.001	0	0	0	0
hsa-146a	198	242	0	0.06	198	242	0.001	0	0	0	0
hsa-374b	205	240	0	0.06	205	240	0.001	0	0	0	0
hsa-20b	151	230	0	0.05	151	230	0.001	0	0	0	0
hsa-142	185	227	0	0.05	185	227	0.001	0	0	0	0
hsa-502	158	226	0	0.05	158	226	0.001	0	0	0	0
hsa-130b	171	215	0	0.05	171	215	0.001	0	0	0	0
hsa-28	141	213	0	0.05	141	213	0.001	0	0	0	0
hsa-342	156	196	0	0.05	156	196	0.000	0	0	0	0
hsa-361	168	192	0	0.05	168	192	0.000	0	0	0	0
hsa-135a	123	186	0	0.04	123	186	0.000	0	0	0	0
hsa-218	106	183	0	0.04	106	183	0.000	0	0	0	0
hsa-194	129	178	0	0.04	129	178	0.000	0	0	0	0
hsa-1259	15	172	0	0.04	15	172	0.000	0	0	0	0
hsa-362	134	169	0	0.04	134	169	0.000	0	0	0	0
hsa-320a	90	151	0	0.04	90	151	0.000	0	0	0	0

hsa-455	117	145	0	0.03	117	145	0.000	0	0	0		0
hsa-505	133	145	0	0.03	133	145	0.000	0	0	0		0
hsa-151	113	141	0	0.03	113	141	0.000	0	0	0		0
hsa-301a	105	137	0	0.03	105	137	0.000	0	0	0		0
hsa-664	87	135	0	0.03	87	135	0.000	0	0	0		0
hsa-326	96	131	0	0.03	96	131	0.000	0	0	0		0
hsa-185	111	130	0	0.03	111	130	0.000	0	0	0		0
hsa-152	116	128	0	0.03	116	128	0.000	0	0	0		0
hsa-363	98	126	0	0.03	98	126	0.000	0	0	0		0
hsa-128	84	125	0	0.03	84	125	0.000	0	0	0		0
hsa-183	98	120	0	0.03	98	120	0.000	0	0	0		0
hsa-188	109	120	0	0.03	109	120	0.000	0	0	0		0
hsa-139	89	118	0	0.03	89	118	0.000	0	0	0		0
hsa-768	69	118	0	0.03	69	118	0.000	0	0	0		0
hsa-598	83	116	0	0.03	83	116	0.000	0	0	0		0
hsa-324	103	113	0	0.03	103	113	0.000	0	0	0		0
hsa-708	94	111	0	0.03	94	111	0.000	0	0	0		0
hsa-132	92	106	0	0.03	92	106	0.000	0	0	0		0
hsa-551b	72	106	0	0.03	72	106	0.000	0	0	0		0
hsa-542	67	103	0	0.02	67	103	0.000	0	0	0		0
hsa-32	68	99	0	0.02	68	99	0.000	0	0	0		0
hsa-136	72	94	0	0.02	72	94	0.000	0	0	0		0
hsa-193a	69	94	0	0.02	69	94	0.000	0	0	0		0
hsa-215	68	93	0	0.02	68	93	0.000	0	0	0		0
hsa-886	38	92	0	0.02	38	92	0.000	0	0	0		0
hsa-92b	78	90	0	0.02	78	90	0.000	0	0	0		0
hsa-33a	74	89	0	0.02	74	89	0.000	0	0	0		0
hsa-18a	67	85	0	0.02	67	85	0.000	0	0	0		0
hsa-768	40	85	0	0.02	40	85	0.000	0	0	0		0
hsa-1248	51	83	0	0.02	51	83	0.000	0	0	0		0
hsa-324	81	83	0	0.02	81	83	0.000	0	0	0		0
hsa-455	67	83	0	0.02	67	83	0.000	0	0	0		0
hsa-135b	57	75	0	0.02	57	75	0.000	0	0	0		0
hsa-340	55	75	0	0.02	55	75	0.000	0	0	0		0
hsa-744	58	75	0	0.02	58	75	0.000	0	0	0		0
hsa-99b	74	74	0	0.02	74	74	0.000	0	0	0		0
hsa-500	57	72	0	0.02	57	72	0.000	0	0	0		0
hsa-376c	54	71	0	0.02	54	71	0.000	0	0	0		0
hsa-501	58	68	0	0.02	58	68	0.000	0	0	0		0
hsa-1280	57	65	0	0.02	57	65	0.000	0	0	0		0
hsa-450a	45	63	0	0.02	45	63	0.000	0	0	0		0
hsa-335	53	61	0	0.01	53	61	0.000	0	0	0		0
hsa-338	53	61	0	0.01	53	61	0.000	0	0	0		0
hsa-886	31	60	0	0.01	31	60	0.000	0	0	0		0
hsa-320d	49	57	0	0.01	49	57	0.000	0	0	0		0
hsa-98	53	56	0	0.01	53	56	0.000	0	0	0		0
hsa-320b	43	54	0	0.01	43	54	0.000	0	0	0		0
hsa-652	53	54	0	0.01	53	54	0.000	0	0	0		0
hsa-1297	35	53	0	0.01	35	53	0.000	0	0	0		0
hsa-495	38	53	0	0.01	38	53	0.000	0	0	0		0
hsa-873	40	53	0	0.01	40	53	0.000	0	0	0		0
hsa-95	43	53	0	0.01	43	53	0.000	0	0	0		0
hsa-887	48	52	0	0.01	48	52	0.000	0	0	0		0
hsa-328	46	49	0	0.01	46	49	0.000	0	0	0		0
hsa-214	48	48	0	0.01	48	48	0.000	0	0	0		0
hsa-503	44	48	0	0.01	44	48	0.000	0	0	0		0
hsa-494	32	44	0	0.01	32	44	0.000	0	0	0		0
hsa-874	32	44	0	0.01	32	44	0.000	0	0	0		0

hsa-181c	42	43	0	0.01	42	43	0.000	0	0	0	0
hsa-487b	34	43	0	0.01	34	43	0.000	0	0	0	0
hsa-532	42	43	0	0.01	42	43	0.000	0	0	0	0
hsa-374a	38	40	0	0.01	38	40	0.000	0	0	0	0
hsa-940	36	39	0	0.01	36	39	0.000	0	0	0	0
hsa-211	28	38	0	0.01	28	38	0.000	0	0	0	0
hsa-423	34	38	0	0.01	34	38	0.000	0	0	0	0
hsa-379	37	37	0	0.01	37	37	0.000	0	0	0	0
hsa-421	37	37	0	0.01	37	37	0.000	0	0	0	0
hsa-96	36	37	0	0.01	36	37	0.000	0	0	0	0
hsa-339	30	32	0	0.01	30	32	0.000	0	0	0	0
hsa-671	32	32	0	0.01	32	32	0.000	0	0	0	0
hsa-1307	31	31	0	0.01	31	31	0.000	0	0	0	0
hsa-33b	28	31	0	0.01	28	31	0.000	0	0	0	0
hsa-342	30	31	0	0.01	30	31	0.000	0	0	0	0
hsa-224	28	30	0	0.01	28	30	0.000	0	0	0	0
hsa-625	26	29	0	0.01	26	29	0.000	0	0	0	0
hsa-769	27	28	0	0.01	27	28	0.000	0	0	0	0
hsa-190	25	27	0	0.01	25	27	0.000	0	0	0	0
hsa-197	25	26	0	0.01	25	26	0.000	0	0	0	0
hsa-337	23	26	0	0.01	23	26	0.000	0	0	0	0
hsa-382	25	26	0	0.01	25	26	0.000	0	0	0	0
hsa-582	23	25	0	0.01	23	25	0.000	0	0	0	0
hsa-766	20	25	0	0.01	20	25	0.000	0	0	0	0
has-9	21	24	0	0.01	21	24	0.000	0	0	0	0
hsa-212	22	22	0	0.01	22	22	0.000	0	0	0	0
hsa-628	20	22	0	0.01	20	22	0.000	0	0	0	0
hsa-127	20	20	0	0.01	20	20	0.000	0	0	0	0
hsa-153	20	20	0	0.01	20	20	0.000	0	0	0	0
hsa-330	19	20	0	0.01	19	20	0.000	0	0	0	0
hsa-1270	15	19	0	0	15	19	0.000	0	0	0	0
hsa-320c	18	19	0	0	18	19	0.000	0	0	0	0
hsa-409	18	19	0	0	18	19	0.000	0	0	0	0
hsa-629	17	19	0	0	17	19	0.000	0	0	0	0
hsa-1246	17	17	0	0	17	17	0.000	0	0	0	0
hsa-1274b	17	17	0	0	17	17	0.000	0	0	0	0
hsa-1301	17	17	0	0	17	17	0.000	0	0	0	0
hsa-18b	16	17	0	0	16	17	0.000	0	0	0	0
hsa-545	14	17	0	0	14	17	0.000	0	0	0	0
hsa-720	16	17	0	0	16	17	0.000	0	0	0	0
hsa-1271	15	16	0	0	15	16	0.000	0	0	0	0
hsa-452	12	16	0	0	12	16	0.000	0	0	0	0
hsa-654	12	16	0	0	12	16	0.000	0	0	0	0
hsa-301b	12	15	0	0	12	15	0.000	0	0	0	0
hsa-337	15	15	0	0	15	15	0.000	0	0	0	0
hsa-149	13	14	0	0	13	14	0.000	0	0	0	0
hsa-181d	14	14	0	0	14	14	0.000	0	0	0	0
hsa-34c	13	13	0	0	13	13	0.000	0	0	0	0
hsa-543	13	13	0	0	13	13	0.000	0	0	0	0
hsa-576	12	13	0	0	12	13	0.000	0	0	0	0
hsa-579	13	13	0	0	13	13	0.000	0	0	0	0
hsa-876	12	13	0	0	12	13	0.000	0	0	0	0
hsa-1285	12	12	0	0	12	12	0.000	0	0	0	0
hsa-486	11	12	0	0	11	12	0.000	0	0	0	0
hsa-642	11	12	0	0	11	12	0.000	0	0	0	0
hsa-1268	10	11	0	0	10	11	0.000	0	0	0	0
hsa-154	11	11	0	0	11	11	0.000	0	0	0	0
hsa-370	11	11	0	0	11	11	0.000	0	0	0	0

hsa-590	7	11	0	0	7	11	0.000	0	0	0	0
hsa-1249	9	10	0	0	9	10	0.000	0	0	0	0
hsa-1287	10	10	0	0	10	10	0.000	0	0	0	0
hsa-381	7	10	0	0	7	10	0.000	0	0	0	0
hsa-296	5	9	0	0	5	9	0.000	0	0	0	0
hsa-542	9	9	0	0	9	9	0.000	0	0	0	0
hsa-671	9	9	0	0	9	9	0.000	0	0	0	0
hsa-1181	8	8	0	0	8	8	0.000	0	0	0	0
hsa-1247	7	8	0	0	7	8	0.000	0	0	0	0
hsa-147b	7	8	0	0	7	8	0.000	0	0	0	0
hsa-330	8	8	0	0	8	8	0.000	0	0	0	0
hsa-627	8	8	0	0	8	8	0.000	0	0	0	0
hsa-876	8	8	0	0	8	8	0.000	0	0	0	0
hsa-1275	7	7	0	0	7	7	0.000	0	0	0	0
hsa-144	7	7	0	0	7	7	0.000	0	0	0	0
hsa-409	7	7	0	0	7	7	0.000	0	0	0	0
hsa-584	7	7	0	0	7	7	0.000	0	0	0	0
hsa-589	7	7	0	0	7	7	0.000	0	0	0	0
hsa-1	6	6	0	0	6	6	0.000	0	0	0	0
hsa-125a	6	6	0	0	6	6	0.000	0	0	0	0
hsa-329	6	6	0	0	6	6	0.000	0	0	0	0
hsa-331	6	6	0	0	6	6	0.000	0	0	0	0
hsa-369	6	6	0	0	6	6	0.000	0	0	0	0
hsa-432	6	6	0	0	6	6	0.000	0	0	0	0
hsa-483	6	6	0	0	6	6	0.000	0	0	0	0
hsa-127	5	5	0	0	5	5	0.000	0	0	0	0
hsa-299	5	5	0	0	5	5	0.000	0	0	0	0
hsa-299	4	5	0	0	4	5	0.000	0	0	0	0
hsa-369	5	5	0	0	5	5	0.000	0	0	0	0
hsa-486	5	5	0	0	5	5	0.000	0	0	0	0
hsa-665	5	5	0	0	5	5	0.000	0	0	0	0
hsa-122	3	4	0	0	3	4	0.000	0	0	0	0
hsa-1229	4	4	0	0	4	4	0.000	0	0	0	0
hsa-491	4	4	0	0	4	4	0.000	0	0	0	0
hsa-499	4	4	0	0	4	4	0.000	0	0	0	0
hsa-615	4	4	0	0	4	4	0.000	0	0	0	0
hsa-641	3	4	0	0	3	4	0.000	0	0	0	0
hsa-656	1	4	0	0	1	4	0.000	0	0	0	0
hsa-769	4	4	0	0	4	4	0.000	0	0	0	0
hsa-877	4	4	0	0	4	4	0.000	0	0	0	0
hsa-1224	3	3	0	0	3	3	0.000	0	0	0	0
hsa-133a	3	3	0	0	3	3	0.000	0	0	0	0
hsa-146b	3	3	0	0	3	3	0.000	0	0	0	0
hsa-1827	3	3	0	0	3	3	0.000	0	0	0	0
hsa-205	3	3	0	0	3	3	0.000	0	0	0	0
hsa-450b	3	3	0	0	3	3	0.000	0	0	0	0
hsa-487a	3	3	0	0	3	3	0.000	0	0	0	0
hsa-493	3	3	0	0	3	3	0.000	0	0	0	0
hsa-511	3	3	0	0	3	3	0.000	0	0	0	0
hsa-550	3	3	0	0	3	3	0.000	0	0	0	0
hsa-570	2	3	0	0	2	3	0.000	0	0	0	0
hsa-942	3	3	0	0	3	3	0.000	0	0	0	0
hsa-1236	2	2	0	0	2	2	0.000	0	0	0	0
hsa-1250	2	2	0	0	2	2	0.000	0	0	0	0
hsa-1274a	2	2	0	0	2	2	0.000	0	0	0	0
hsa-129	2	2	0	0	2	2	0.000	0	0	0	0
hsa-134	2	2	0	0	2	2	0.000	0	0	0	0
hsa-188	2	2	0	0	2	2	0.000	0	0	0	0

hsa-190b	2	2	0	0	2	2	0.000	0	0	0	0
hsa-219	2	2	0	0	2	2	0.000	0	0	0	
hsa-376a	1	2	0	0	1	2	0.000	0	0	0	0
hsa-377	2	2	0	0	2	2	0.000	0	0	0	0
hsa-411	2	2	0	0	2	2	0.000	0	0	0	0
hsa-489	2	2	0	0	2	2	0.000	0	0	0	0
hsa-548b	2	2	0	0	2	2	0.000	0	0	0	0
hsa-577	2	2	0	0	2	2	0.000	0	0	0	0
hsa-923	2	2	0	0	2	2	0.000	0	0	0	0
hsa-941	2	2	0	0	2	2	0.000	0	0	0	0
hsa-1225	1	1	0	0	1	1	0.000	0	0	0	0
hsa-1237	1	1	0	0	1	1	0.000	0	0	0	0
hsa-124	1	1	0	0	1	1	0.000	0	0	0	0
hsa-1254	1	1	0	0	1	1	0.000	0	0	0	0
hsa-1255a	1	1	0	0	1	1	0.000	0	0	0	0
hsa-1262	1	1	0	0	1	1	0.000	0	0	0	0
hsa-1263	1	1	0	0	1	1	0.000	0	0	0	0
hsa-1277	1	1	0	0	1	1	0.000	0	0	0	0
hsa-1284	1	1	0	0	1	1	0.000	0	0	0	0
hsa-129	1	1	0	0	1	1	0.000	0	0	0	0
hsa-1290	1	1	0	0	1	1	0.000	0	0	0	0
hsa-1296	1	1	0	0	1	1	0.000	0	0	0	0
hsa-1306	1	1	0	0	1	1	0.000	0	0	0	0
hsa-139	1	1	0	0	1	1	0.000	0	0	0	0
hsa-323	1	1	0	0	1	1	0.000	0	0	0	0
hsa-346	1	1	0	0	1	1	0.000	0	0	0	0
hsa-34b	1	1	0	0	1	1	0.000	0	0	0	0
hsa-34c	1	1	0	0	1	1	0.000	0	0	0	0
hsa-380	1	1	0	0	1	1	0.000	0	0	0	0
hsa-433	1	1	0	0	1	1	0.000	0	0	0	0
hsa-490	1	1	0	0	1	1	0.000	0	0	0	0
hsa-491	1	1	0	0	1	1	0.000	0	0	0	0
hsa-514	1	1	0	0	1	1	0.000	0	0	0	0
hsa-519d	1	1	0	0	1	1	0.000	0	0	0	0
hsa-548a	1	1	0	0	1	1	0.000	0	0	0	0
hsa-548e	1	1	0	0	1	1	0.000	0	0	0	0
hsa-551a	1	1	0	0	1	1	0.000	0	0	0	0
hsa-561	1	1	0	0	1	1	0.000	0	0	0	0
hsa-592	1	1	0	0	1	1	0.000	0	0	0	0
hsa-595	1	1	0	0	1	1	0.000	0	0	0	0
hsa-597	1	1	0	0	1	1	0.000	0	0	0	0
hsa-636	1	1	0	0	1	1	0.000	0	0	0	0
hsa-650	1	1	0	0	1	1	0.000	0	0	0	0
hsa-663b	1	1	0	0	1	1	0.000	0	0	0	0
hsa-758	1	1	0	0	1	1	0.000	0	0	0	0
hsa-765	1	1	0	0	1	1	0.000	0	0	0	0
hsa-802	1	1	0	0	1	1	0.000	0	0	0	0
hsa-885	1	1	0	0	1	1	0.000	0	0	0	0
hsa-889	1	1	0	0	1	1	0.000	0	0	0	0
hsa-890	1	1	0	0	1	1	0.000	0	0	0	0
hsa-944	1	1	0	0	1	1	0.000	0	0	0	0
hsa-37	37	0	0.01	37	37	0	0.000	0	0	0	target
			4.44	136.04			1				

Ref

target
target
target
target
target
target
target
target
target
target
target
target
target
target
target
target
target
target
target
target
target
target
target
target
target
target
target
target
target
target
target
target
target
target
target
target
target
target
target
target
target
target
target
target
target
target
target
target
target
target
target
target
target
target
target
target
target
target

Ref

target
target
target
target
target
target
target
target
target
target
target
target
target
target
target
target
target
target
target
target
target
target
target
target
target
target
target
target
target
target
target
target
target
target
target
target
target
target
target
target
target
target
target
target
target
target
target
target
target
target
target
target
target
target
target
target
target
target

target
target
target
target
target
target
target
target
target
target
target
target
target
target
target
target
target
target
target
target
target
target
target
target
target
target
target
target
target
target
target
target
target
target
target
target
target
target
target
target
target
target
target
target
target
target
target
target
target
target
target
target
target
target
target
target
target
target

Ref

target
target
target
target
target
target
target
target
target
target
target
target
target
target
target
target
target
target
target
target
target
target
target
target
target
target
target
target
target
target
target
target
target
target
target
target
target
target
target
target
target
target
target
target
target
target
target
target
target
target
target
target
target
target
target
target
target
target

Ref

target
target
target
target
target
target
target
target
target
target
target
target
target
target
target
target
target
target
target
target
target
target
target
target
target
target
target
target
target
target
target
target
target
target
target
target
target
target
target
target
target
target
target
target
target
target
target
target
target
target
target
target
target
target
target
target
target
target

Ref

target

target
target
target
target
target
target
target
target
target
target
target
target
target
target
target
target
target
target
target
target
target
target
target
target
target
target
target
target
target
target
target
target
target
target
target
target
target
target
target
target
target
target
target
target
target
target
target
target
target
no
